# Regional HNSCC metabolomics reveals widespread changes to one-carbon metabolism and S-adenosylmethionine metabolism across tumour core, tumour edge and adjacent non-tumour tissues

**DOI:** 10.1038/s41416-026-03410-4

**Published:** 2026-04-29

**Authors:** Andrew D. Southam, James A. Higginson, Gavin R. Lloyd, Matthew J. Smith, Lauren E. Cruchley-Fuge, Rachel Spruce, Ossama Edbali, Ralf J. M. Weber, Hisham Mehanna, Nikolaos Batis

**Affiliations:** 1 Phenome Centre Birmingham, University of Birmingham, Edgbaston, Birmingham UK; 2 School of Biosciences, College of Life and Environmental Sciences, University of Birmingham, Edgbaston, Birmingham UK; 3 Institute of Head and Neck Studies and Education (InHANSE), Department of Cancer and Genomic Sciences, College of Medicine and Health, University of Birmingham, Birmingham, UK; 4 Department of Metabolism, Digestion and Reproduction, Imperial College London, London, UK; 5 Unit of Integrative Metabolomics, Institute of Environmental Medicine, Karolinska Institutet, Stockholm, Sweden; 6 Department of Biomedical Sciences, School of Immunity, Infection and Inflammation, College of Medicine and Health, University of Birmingham, Birmingham, UK

**Keywords:** Cancer metabolism, Head and neck cancer, Cancer metabolism

## Abstract

**Background:**

Cancer, including head and neck squamous cell carcinoma (HNSCC), induces changes to metabolism that drive the disease. Regional metabolomics allows understanding of metabolic variation across the tumour, including in the tumour core, where hypoxia is likely more pronounced.

**Methods:**

Ultra-high performance liquid chromatography-mass spectrometry metabolomics was applied to regionally distinct patient tissue samples: tumour edge, tumour core and adjacent non-tumour. Statistical, correlation and pathway enrichment analyses were performed.

**Results:**

Markers of hypoxia or pseudohypoxia—lactate, succinate, fumarate, and the lactate:pyruvate ratio—were elevated in both core and edge tumour regions relative to adjacent tissue, with a trend toward stronger changes in the core. One-carbon metabolites were altered in HNSCC, including tumour-associated increases of S-adenosylmethionine (SAM) and SAM metabolites (S-adenosylhomocysteine, polyamines, methylated nucleosides, dimethylarginine, trimethylysine and 1-methylnicotinamide). Histidine, tryptophan, choline and folate appear metabolically connected to one-carbon metabolism in HNSCC: histidine, L-kynurenine (tryptophan metabolite), some purine metabolites (including deoxyguanosine, deoxyinosine) and choline were elevated in tumour tissue; while histidine/SAM, L-kynurenine/deoxyguanosine, L-kynurenine/deoxyinosine and folate/methionine were correlated in tumour tissue only.

**Conclusion:**

Tumour edge and core exhibited one-carbon metabolic changes relative to non-tumour, with the magnitude of change generally greater in the core reflecting location dependent variation of SAM metabolism in HNSCC.

## Introduction

Head and neck squamous cell carcinoma (HNSCC) is an aggressive malignancy that arises from the mucous membranes lining the structures of the upper aerodigestive tract, including oral, pharyngeal and laryngeal subsites [1]. It is strongly associated with tobacco and alcohol use, and with high-risk forms of the human papillomavirus (HPV). HNSCC is the seventh most common malignancy worldwide, and is rising in incidence, partly due to rising rates of HPV infection [1]. Morbidity and mortality associated with HNSCC and its treatment is high, and affected patients have complex needs, yet it remains a relatively underfunded area of research.

Thus, there remains a pressing need to increase the biochemical understanding of HNSCC to enable the development of novel treatments that improve clinical outcome. Here, we focus on biochemistry related to metabolism. Considerable cellular metabolic changes occur during the process of carcinogenesis, including in HNSCC, that could represent potential therapeutic targets [2, 3]. Cancer-specific metabolic changes elevate anabolic processes to drive proliferation and growth, while also maintaining energetic demands [3]. Intra-tumour regional location influences cancer metabolism primarily due to oxygen and nutrient depletion towards the tumour core caused by tumour growth superseding that of the supporting vasculature [4, 5]. Tumour hypoxia is common in HNSCC, where it drives cancer metastasis and drug resistance, and leads to poorer prognosis [6–8].

One-carbon metabolism is perturbed in several cancers [9] and may be influenced by hypoxia [10, 11], however an understanding of one-carbon metabolism in HNSCC is incomplete [12]. One-carbon metabolism encompasses folate metabolism and the methionine cycle [9, 13]. Folate metabolism drives purine and thymidine synthesis and contributes to NADP + /NADPH ratio maintenance [13]. Targeting folate metabolism induces anticancer activity through DNA synthesis disruption [9]. The methionine cycle generates S-adenosylmethionine (SAM) [14], which is used for several processes implicated in cancer including polyamine synthesis and the methylation of DNA, RNA, proteins and some metabolites [14–17]. SAM is elevated in primary tumour tissue and saliva from HNSCC patients [18]. SAM has both pro-cancer [19, 20] and anti-cancer [21] effects, thus the tight regulation of SAM availability appears key in cancer [19, 22, 23].

Here we apply univariate and multivariate statistical analysis to steady-state ultra-high performance liquid chromatography mass spectrometry (UHPLC-MS) analyses of matched samples from three regionally distinct primary sample types from HNSCC patients: [i] non-tumour tissue (5 cm away from the tumour), [ii] tumour edge tissue, [iii] core tumour tissue (from the tumour centre but avoiding any necrotic core). Over-representation analysis is used to indicate which pathways are disrupted and correlation analysis is used to identify changes to relationships between pairs of metabolites, metabolic processes or pathways [24].

## Methods

### Materials

Samples were collected from 22 patients undergoing surgical resection of head and neck cancer. Representative samples were taken from the central core of the tumour (C), from the visible tumour edge (E), and from matched normal mucosa (N) at least 5 cm from the tumour site. All patients gave informed consent to participate in this study (ethical approval 16/NW/0265). Samples were immediately flash frozen and stored at –80 ⁰C. Acetonitrile, methanol, water and propan-2-ol were all LC-MS grade (Fisher Scientific). Chloroform was HPLC grade (Fisher Scientific).

### Experimental design

Polar compounds (metabolites) were measured in core tumour tissue (C), edge tumour tissue (E) and surrounding non-tumour tissue (N). For a total of *n* = 22 patients; *n* = 18 had C, E & N tissue; *n* = 2 had C & N tissue only; *n* = 2 had E & N tissue only.

### Metabolite extraction, process blank preparation, resuspension for ultra-high performance liquid chromatography-mass spectrometry (UHPLC-MS) and quality control (QC) preparation

Polar metabolites were extracted from tissue samples using a biphasic methanol/chloroform/water approach and then dried (Supplementary Information). Process blank samples were prepared in the same way in the absence of tissue, and then all dried samples were resuspended in 3:1 acetonitrile:water, and intrastudy QC samples were prepared by pooling 40 µL from biological samples (Supplementary information). Samples were then maintained at 4 ⁰C.

### UHPLC-MS analysis

Biological samples were analysed by hydrophilic interaction chromatography (HILIC) UHPLC-MS in positive and negative ion modes (Supplementary Information). Quality control (QC) samples were analysed throughout the analytical batch and process blank samples were analysed at the beginning and end of the analytical batch (Supplementary Information). For metabolite annotation, QC samples were analysed by data dependent MS^2^ over five mass ranges (70–120 m/z; 120–170 m/z; 170–220 m/z; 220–270 m/z; 270–1050 m/z), and where necessary, authentic chemical standards were also analysed by the same HILIC UHPLC-MS(/MS) assays (Supplementary Information).

### Raw data processing and statistics

Data processing and analysis is described in detail in the Supplementary Information. Briefly, for the main study, features were retained in the data matrix if they were: present in >90% of QC samples; had a peak intensity relative standard deviation (RSD) < 30% across QC samples; and had a mean QC/extract blank intensity ratio of >20% and present in >50% of the samples (Supplementary Information). Peak intensities were then normalised (Probabilistic Quotient Normalisation), and for multivariate statistics missing value imputation and generalised log scaling was applied (Supplementary Information). Univariate analysis (ANOVA and post hoc testing) and multivariate analysis (PCA and PLS-DA) were applied as detailed in the Supplementary Information.

### Metabolite annotation and pathway analysis

For metabolite annotation and identification, two complementary strategies were employed. First, experimental retention times (RTs) and/or MS/MS spectra were matched against authentic chemical standards. When standard-derived MS/MS spectra were unavailable, experimental MS/MS data were compared to the mzCloud spectral library (mzcloud.org) or other published data (Supplementary Fig. S1; see Supplementary Information for full details). Pathway analysis of all annotated significantly changing metabolites was conducted in MetaboAnalyst 6.0 (https://www.metaboanalyst.ca/; Supplementary Information).

### Correlation analysis

Metabolites related to one-carbon metabolism were annotated/identified within the dataset regardless of their significance status. Spearman’s Rank correlation was applied to the responses of these metabolites separately within either: (a) non-tumour tissue, (b) tumour edge tissue or (c) core tumour tissue. p-values for Spearman Rank correlation were False Discovery corrected (q < 0.05) within each tissue type to account for multiple comparisons.

## Results

### HNSCC tumour and non-tumour tissues are metabolically distinct, with greater metabolic change observed between core tumour/non-tumour samples compared to tumour edge/non-tumour samples

**Fig. 1:**
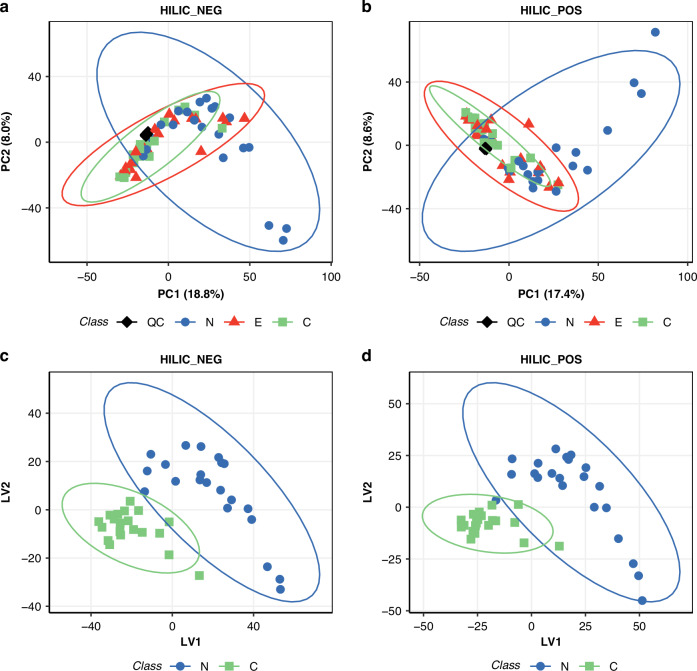
Multivariate analysis of metabolite data across non-tumour and HNSCC tissue phenotypes. Principal components analysis (PCA) of UHPLC-MS metabolite data from non-tumour [N], edge tumour [E], and core tumour [C] tissues (**a**, **b**). Partial least squares discriminant analysis (PLS-DA) of non-tumour [N] and core tumour [C] samples (**c**, **d**). Tenfold cross validation was applied to the PLS-DA (see “Methods” section and Table 1).

More than 18% of the UHPLC-MS data significantly changed across phenotypes (Table 1). The most metabolic variation occurred between non-tumour/core tumour comparisons, followed by non-tumour/tumour edge comparisons, with much less variation between edge/core tumour comparisons (Table 1). Tumour samples have a narrower distribution on the principal components analysis (PCA) scores plot than non-tumour samples (Fig. 1) indicating a more homogeneous metabolome. Partial least squared discriminate analysis (PLS-DA) classified non-tumour and core tumour samples with high accuracy (Table 1 and Fig. 1). 169 significantly changed features were annotated across positive and negative ion datasets, which equated to 49 unique compounds (Supplementary Table S1, Supplementary Fig. S1).

**Table 1:** Numbers of detected features and partial least squares discriminate analysis (PLS-DA) accuracy in the HILIC UHPLC-MS positive and negative ion datasets, including the numbers of significantly changing features across non-tumour, tumour edge and core tumour phenotypes.

Dataset	Number of features after quality filtering	Number of significantly changing features (q < 0.05)^#^	% of significantly changed features in dataset	Post hoc analysis—number of significant features			PLS-DA analysis of non-tumour vs core tumour balanced accuracy (validation set)
				Non-tumour/ tumour edge	Non-tumour/ core tumour	Tumour edge / core tumour	
HILIC positive ion	5718	1248	21.8%	934	1195	79	87% ±9%
HILIC negative ion	7047	1278	18.1%	971	1235	133	84% ±11%

^#^ANOVA with false discovery rate [FDR] < 5% across non-tumour, tumour edge and core tumour samples.

### Pathway mapping of the significantly changed metabolites revealed an overrepresentation of KEGG pathways related to one-carbon, polyamine and cysteine metabolism in HNSCC tumours

**Fig. 2:**
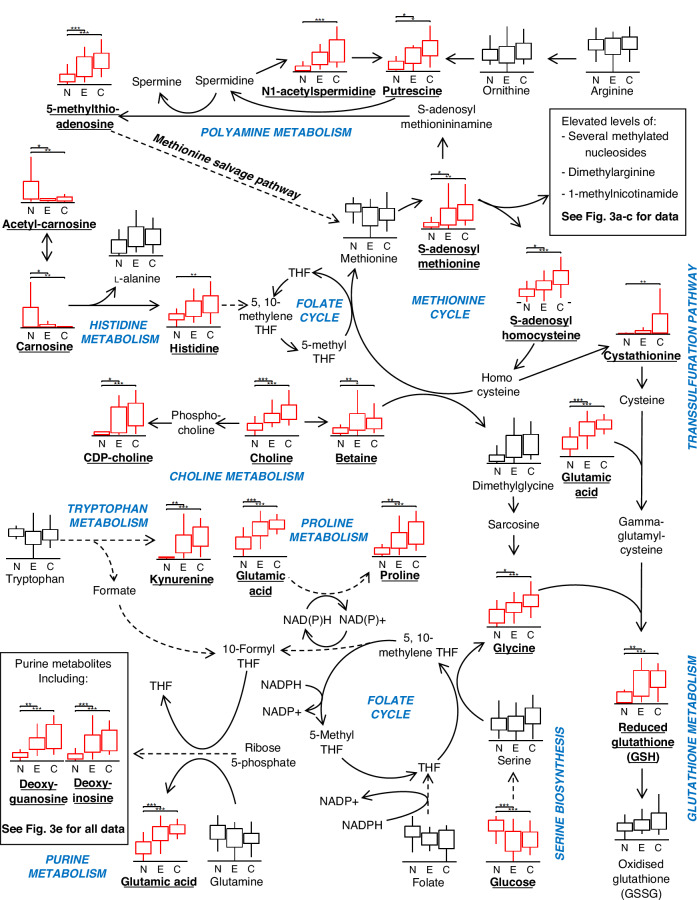
Metabolic pathway mapping of metabolites related to one‑carbon, polyamine and cysteine metabolism in non-tumour and HNSCC tumour tissues. UHPLC-MS metabolite response data for non-tumour [N], edge tumour [E], and core tumour [C] tissues from HNSCC patients transposed on to a metabolic map of processes related to one-carbon, polyamine and cysteine metabolism (based on KEGG pathways in combination with referenced work [9, 13, 25]). Detected metabolites are depicted by a box and whisker plot while non-detected metabolites are depicted by the metabolite name only. Metabolites that have significant intensity changes across classes (ANOVA FDR < 5%) are shown as red box and whisker plots with the metabolite name in **bold-underline**, and posthoc significance is indicated (Tukeys HSD; *p < 0.05, **p < 0.005, ***p < 0.001). Considering box and whisker plots, standard Tukey versions were used with the box covering the lower to upper quartileand the whiskers are 1.5x the interquartile range. Solid arrows depict that a direct metabolic connection exists between metabolites, while dashed arrows depict connections between metabolites include one or more intermediate metabolites. The metabolic map is broken down into smaller metabolic processes—defined in blue italicised capital letters. THF tetrahydrofolate.

**Fig. 3:**
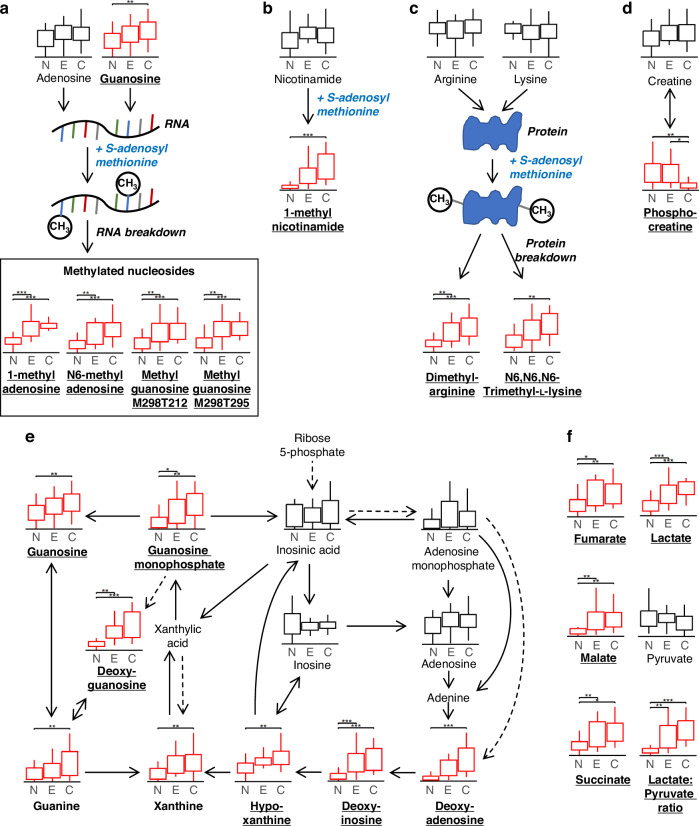
Alterations in methylated metabolites, their unmethylated counterparts, purine metabolites, and hypoxia‑ and energetics‑related compounds in non-tumour and HNSCC tumour tissues. UHPLC-MS metabolite response data for non-tumour [N], edge tumour [E], and core tumour [C] tissues from HNSCC patients transposed on to metabolic map of processes related to (**a**) nucleoside methylation, **b** nicotinamide methylation, **c** arginine and lysine methylation, **d** creatine/phosphocreatine metabolism, **e** purine metabolism and **f** selected metabolites from glycolysis and the TCA cycle that could infer hypoxic/pseudohypoxic changes. Metabolic maps are based on KEGG pathways in combination with referenced work [23, 26–29]. Metabolites that have significant intensity changes across classes (ANOVA FDR < 5%) are shown as red box and whisker plots with the metabolite name in **bold-underline**, and posthoc significance is indicated (Tukeys HSD; *p < 0.05, **p < 0.005, ***p < 0.001). Considering box and whisker plots, standard Tukey versions were used with the box covering the lower to upper quartile and the whiskers are 1.5x the interquartile range. Solid arrows depict that a direct metabolic connection exists between metabolites, while dashed arrows depict connections between metabolites include one or more intermediate metabolites. Note: 2x methyguanosine isomers were identified (**a**)—here the M298T212 and M298T295 refer to the peak identifier number (Supplementary Table S3).

Pathway analysis of the significantly changed annotated metabolites (Supplementary Table S1) indicated enrichment of several pathways including *purine metabolism*, *arginine and proline metabolism*, *histidine metabolism*, *glutathione metabolism*, and *cysteine & methionine metabolism* (*p* < 0.05, see Supplementary Table S2 for full lists). Relative to non-tumour tissue, the coverage of metabolic changes in core and edge tumour were similar (Supplementary Table S2). Manual investigation of the enriched metabolic pathways led us to define the following metabolic processes as altered in HNSCC (see Supplementary Table S2): (i) the methionine cycle, (ii) the folate cycle, (iii) cysteine metabolism and (iv) polyamine metabolism. Metabolites in (or related to) these processes were annotated from our data regardless of significance status (Supplementary Table S3; Supplementary Fig. S1) and their responses across the three phenotypes are displayed within metabolic pathway maps based on the KEGG database (www.genome.jp/kegg/) with input from referenced publications [9, 13, 23, 25–28] (Figs. 2, 3). The remainder of the results section focuses on these metabolites in Supplementary Table S3.

### Considering metabolites related to one-carbon, polyamine and cysteine metabolism, there is a sharp drop in the number of significant metabolic correlations in core tumour tissue compared to tumour edge and non-tumour tissues

**Fig. 4:**
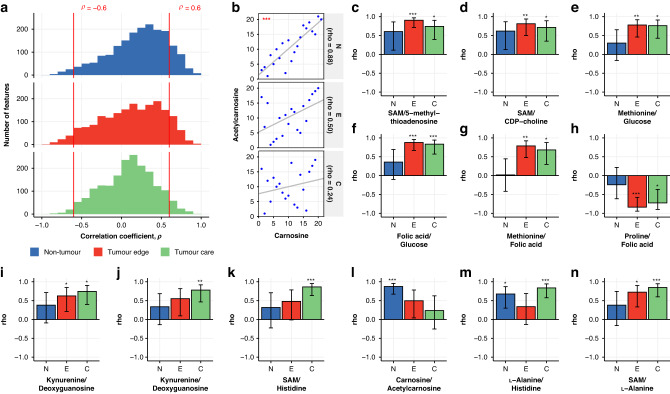
Key correlations between metabolite-pairs related to one‑carbon, polyamine and cysteine metabolism in non-tumour and HNSCC tumour tissues. Spearman’s rank correlation analysis of UHPLC-MS metabolite response data for metabolites related to one-carbon, polyamine and cysteine metabolism (Supplementary Table S3). Note that correlations were calculated *within*, not across, classes (non-tumour [N], edge tumour [E], and core tumour [C] tissues from HNSCC patients). A histogram of all correlation coefficients is shown for each phenotype (**a**), and correlation coefficients for specific metabolite pairs are indicated (**b**–**n**; see also Supplementary Table S4, Supplementary Fig. S2). Error bars represent the upper and lower 95% confidence limits. SAM   S-adenosylmethionine, CDP cytidine diphosphate. *q < 0.05, **q < 0.01, ***q < 0.001 (FDR corrected).

Correlation analysis can identify relationships between pairs of metabolites in each phenotype, with metabolite pairs either: [i] correlated in all phenotypes; [ii] uncorrelated in all phenotypes; or [iii] correlated in some phenotypes but not others. Considering metabolites related to one-carbon, polyamine and cysteine metabolism (Supplementary Table S3), there were many significantly correlated metabolite pairs in non-tumour (19.3% of all tested pairs; q < 0.05), less correlated metabolite pairs in tumour edge (16.5%), and very few in core tumour (3.0%; Supplementary Table S4). The correlation coefficient distribution of all tested metabolite pairs revealed tumour edge (median r = 0.17) as intermediate to non-tumour (median r = 0.26) and core tumour (median r = 0.11, Fig. 4). These data suggest that tumour tissue has higher metabolic dysregulation relative to non-tumour tissue, which is most pronounced in the tumour core. Correlations that remain in core and tumour edge tissue likely indicate important relationships in these phenotypes, with several illustrated and discussed below (Fig. 4; Supplementary Fig. S2).

### S-adenosylmethionine (SAM), S-adenosylhomocysteine (SAH) and metabolic products that require SAM were elevated in HNSCC tumour tissue suggesting an increase in methionine cycle activity and onward SAM metabolism

Methionine cycle metabolites, S-adenosylmethionine (SAM) and S-adenosylhomocysteine (SAH), were increased in tumour edge and core tumour relative to non-tumour (greatest change in core/non-tumour, Fig. 2). Dimethylarginine, trimethyl-L-lysine, 1-methylnicotinamide and several methylated nucleosides (which all require SAM to become methylated, producing SAH in return [23, 27–29]) were elevated in core tumour relative to non-tumour tissue, with the methylated nucleosides and dimethylarginine also elevated in tumour edge relative to non-tumour (Fig. 3a–c). It could not be determined whether dimethylarginine was the symmetric or asymmetric isomer [28] (both have similar retention times, Supplementary Table S3). SAM is also used for polyamine synthesis [16]. Acetylspermidine (polyamine), putrescine (polyamine precursor) and 5-methylthioadenosine (biproduct of polyamine synthesis) were increased in core tumour tissue relative to non-tumour. Putrescine and 5-methylthioadenosine were elevated in tumour edge relative to non-tumour but to a lesser extent (Fig. 2). Detection responses of 5-methylthioadenosine and SAM were positively correlated (and thus potentially in equilibrium [24]) in tumour edge and core tumour tissues (Fig. 4c), suggesting that SAM availability and polyamine synthesis are related in HNSCC tumour tissue.

### Several purine metabolites are elevated in HNSCC tumour tissue suggesting an elevation in purine biosynthesis, a process that requires tetrahydrofolate (THF)-derived one-carbon units

Increased responses of the purine metabolites deoxyadenosine, deoxyinosine, deoxyguanosine, hypoxanthine, xanthine, guanine and guanosine monophosphate (GMP) were observed in core tumour tissue relative to non-tumour tissue (deoxyinosine, deoxyguanosine and GMP were also significantly elevated in tumour edge; Fig. 3e) suggesting that purine synthesis may be elevated in tumour tissue and illustrating an apparent increased demand for THF-based one-carbon units [13].

### Changes to choline, folic acid, histidine, proline, and tryptophan metabolism in HNSCC tumour tissue suggests that they may support one-carbon unit production for purine biosynthesis and S-adenosylmethionine production

Choline and CDP-choline were elevated in core and edge tumour relative to non-tumour samples (Fig. 2). SAM and CDP-choline responses were positively correlated in edge and core tumour tissue (Fig. 4d) suggesting a metabolic link between the *CDP-choline pathway* and *the methionine cycle*. As choline is a precursor for both SAM (via its metabolism into betaine [30], a metabolite also elevated in tumour edge and core tumour tissue, Fig. 2) and CDP-choline, the high choline response in tumour tissue may contribute to elevated SAM and CDP-choline levels (Fig. 2).

Methionine, folic acid and glucose were positively correlated (thus potentially in equilibrium [24]) in edge and core tumour tissues, but not in non-tumour tissue (Fig. 4e–g). These correlations could relate to increases in one-carbon metabolism: folate metabolism (via 5-methyl tetrahydrofolate [5-methyl THF]) in combination with glucose metabolism (via serine biosynthesis and subsequent donation of a one-carbon group from serine) can provide 1-carbon units for methionine (and thus SAM) [13] (Fig. 2). The increase in glycine in tumour edge and core tumour further supports this hypothesis, as glycine is released during serine one-carbon donation to the folate cycle [13] (Fig. 2).

Proline was elevated in tumour edge and core tumour tissues relative to non-tumour samples and negatively correlated with folic acid in edge and core tumour tissues but not in non-tumour tissue (Figs. 2 and 4h). A negative correlation may indicate that one metabolite contributes to the production of the other metabolite and itself becomes consumed. This could indicate that proline synthesis is increased in tumour tissue and is linked to folic acid metabolism via NAD(P)H as previously described (10-Formyl THF production provides NAD(P)H for proline synthesis [25]; Fig. 2).

L-kynurenine and the purine metabolites, deoxyinosine and deoxyguanosine, were elevated in tumour edge and core tumour tissues relative to non-tumour tissue (Fig. 2). L-kynurenine/deoxyinosine and L-kynurenine/deoxyguanosine were positively correlated in core tumour (L-kynurenine/deoxyinosine correlated in tumour edge), but uncorrelated in non-tumour tissue (Fig. 4i–j). These relationships could indicate a link between tryptophan metabolism and one-carbon metabolism given that tryptophan catabolism into L-kynurenine releases formate, which can be used for 10-Formyl-THF production [9, 13] and onward purine synthesis [13] (Fig. 2).

Histidine was elevated in core tumour tissue relative to non-tumour tissue (Fig. 2). Histidine and SAM were positively correlated in core tumour and uncorrelated in tumour edge and non-tumour (Fig. 4k). Histidine can support 1-carbon production [9, 13], providing a rationale for these observations. Carnosine and acetylcarnosine (histidine/L-alanine dipeptides) were depleted in tumour tissue (Fig. 2). Carnosine/acetylcarnosine were correlated in non-tumour tissue but not in tumour tissue, whereas histidine/L-alanine correlation strengthened in the tumour core (Fig. 4b, l, m). This suggests that carnosine is catabolised in tumour tissue (Fig. 2). L-alanine levels were unchanged across phenotypes; however, L-alanine and SAM were correlated in tumour tissue but not non-tumour tissue, similar to histidine/SAM (Fig. 4n). Our findings suggest that histidine released from carnosine catabolism may support one-carbon production in tumour tissue.

### Increased glutathione levels in HNSCC tumour tissue suggests an increase in glutathione synthesis via the methionine cycle and cysteine metabolism

Cystathionine—which is derived from the methionine cycle-intermediate homocysteine [31]—was increased in core tumour tissue relative to non-tumour, consistent with the apparent increase in methionine cycle activity we observe in HNSCC (above, Fig. 2). Cystathionine is a transulfuration pathway precursor for cysteine [31], itself a precursor for glutathione [32]. Reduced glutathione (GSH) and glutathione precursors (glycine and glutamic acid) were increased in tumour edge and core tumour tissues relative to non-tumour, while oxidised glutathione (GSSG) was unchanged across phenotypes (Fig. 2). This suggested that glutathione synthesis is increased in HNSCC, potentially driven by increased methionine cycle and transulfuration pathway activity. The outcome of such an event would enhance cellular reducing capacity.

### Elevations of lactate and TCA cycle intermediates in tumour tissue and depletion of phosphocreatine in the tumour core but not the tumour edge indicates regional differences in metabolic energetics

Lactate, succinate, fumarate, and malate were elevated in edge and core tumour tissues compared to non-tumour (Fig. 3f, Supplementary Table S5). Succinate, fumarate, and malate showed similar increases across edge and core, while lactate and the lactate:pyruvate ratio (LPR) trended higher in the core (LPR edge v core q = 0.088; Fig. 3f, Supplementary Table S5). These patterns suggest hypoxia or pseudohypoxia is present in HNSCC, with greater intensity likely in the core. Phosphocreatine levels were decreased in core tumour tissue relative to both non-tumour and tumour edge (creatine unchanged across phenotypes, Fig. 3d), suggesting that phosphocreatine demand was high in core tumour and that ATP demand relative to ATP production was higher here.

## Discussion

This study reveals that widespread changes to one-carbon metabolism occur in primary HNSCC tumour tissue. The elevated S-adenosylmethionine [SAM] levels we observe in HNSCC—along with an apparent increase in SAM utilisation—is consistent with elevated SAM reported in oral cancer [18]. Greater SAM-related changes in the tumour core compared to the tumour edge may indicate a regional response to oxygen and nutrient stress, with hypoxia known to occur in poorly vascularised HNSCC cores [6, 7]. This interpretation is supported by our data, which show elevated levels of lactate, fumarate, malate and succinate in HNSCC tumour tissue – metabolic signatures of hypoxia or pseudohypoxia [33]. Our observed phosphocreatine decrease in the core, but not the edge, may reflect impaired ATP regeneration under hypoxic conditions [34]. In HNSCC, hypoxia drives tumour-promoting metabolism and the impairment of treatment efficacy [6], highlighting the value of regional metabolic profiling in identifying potential therapeutic targets. Below we explore how our metabolic findings relate to SAM and one-carbon metabolism, and how these may be impacted by local tumour conditions.

Our observed elevation of SAM and SAM metabolic products in HNSCC, suggest a high demand for- and use of one-carbon units (CH_3_). SAM is produced via the methionine cycle from the essential amino acid methionine [13]. Methionine procurement—strongly implicated in cancer [14]—comes via the re-methylation of homocysteine, and/or cellular import from an external source with the latter occurring in HNSCC tumours [35]. Considering the former, our data suggests multiple metabolic sources could be used for methionine re-methylation and one-carbon unit provision. Firstly, choline, a potential one-carbon source for SAM production [30], was increased in our HNSCC tumour tissue data—consistent with published data [36]—and correlated with SAM metabolism. In HNSCC, choline uptake transporters are highly expressed and associated with cell viability and SAM production [37]. Secondly, tetrahydrafolate (THF) metabolism can be used for methionine production [9]. We observed that processes requiring THF metabolic outputs—purine synthesis and proline biosynthesis [13, 25]—appeared to increase in HNSCC, consistent with other cancers [38, 39]. Increased THF metabolism in HNSCC was further supported by observed tumour-specific correlations between methionine and THF precursors: folate (THF precursor) and glucose (which can donate one-carbon units to THF via serine and serine hydroxymethyltransferase SHMT [13]). Hypoxia can increase SHMT2 gene expression [11], while in HNSCC, mitochondrial SHMT2 expression is elevated and associated with poor prognosis [40]. Thirdly, tryptophan can provide one-carbon units to THF metabolism [9, 13], a pathway active in cancer [41]. Tumour-specific L-kynurenine elevation and correlation with purine metabolites in our data, supports potential use of this pathway in the HNSCC. Furthermore, production of indoleamine 2,3-dioxygenase (IDO)—the enzyme that converts tryptophan to L-kynurenine – is enhanced by hypoxia [42] and often upregulated in HNSCC [43]. A fourth potential one-carbon source is histidine, which can contribute one-carbon units to THF metabolism [9, 13]. In our study, histidine was elevated in HNSCC and correlated with SAM, supporting its role as a one-carbon source. Additionally, the depletion of carnosine and acetylcarnosine, loss of their mutual correlation, and gain of histidine/alanine correlation in tumour tissue may suggest that carnosine could provide a histidine source—consistent with known cancer-induced carnosine dipeptidase 2 (CNDP2) catabolism of peptides to support amino acid supply [44]. Following methionine re-methylation, methionine is converted to SAM by methionine adenosyltransferase (MAT) [45], with the MAT2A isoform elevated in several cancers, including nasopharyngeal cancer (a HNSCC subtype) [20, 45]. Hypoxia upregulates MAT2A [10] and elevated MAT2A activity promotes tumour aggressiveness, SAM production [45], and can drive external methionine dependence [20]. MAT2A inhibition has anticancer effects [45] especially under methionine depletion [19] or S-methyl-5’-thioadenosine phosphorylase loss [46] (MTAP; enzyme involved in methionine recycling, which is commonly lost in HNSCC [47]).

Our data suggest that the use of SAM (i) as a methyl donor and (ii) for polyamine synthesis increase in HNSCC. Elevated methylated nucleosides observed in HNSCC tumour tissue—including N6-methyladenosine—could indicate increased RNA methylation and/or increased RNA turnover as seen in cancer patients [26]. N6-methyladenosine regulates mRNA stability, splicing and translation [48] and is linked to cancer progression and metastasis [15]. In HNSCC, increased expression of N6-methyladenosine regulatory genes is associated with poor prognosis [49]. Additionally, N6-methyladenosine tends to increase in hypoxia, leading to stabilisation of mRNA under hypoxic conditions [50]. The increase of methylated amino acids observed in our HNSCC tumour data may result from SAM-dependent methylation of arginine and lysine in proteins, with proteolysis releasing dimethylarginine and trimethyllysine [27, 28]. Histone lysine and arginine methylation contributes to cancer drug resistance [51], while disruption of lysine methylation is a potential anticancer target [52] and lysine methyltransferases can be induced by hypoxia [53]. Arginine methylation is catalysed by protein arginine N-methyltransferase (PRMT) enzymes [54], with PRMT1 and PRMT5 overexpressed in HNSCC and oesophageal SCC where they promote oncogenic signalling and poor prognosis [55, 56]. Combined PRMT1/5 inhibition reduces tumour volume in vivo [57], while PRMT1-mediated epidermal growth factor receptor (EGFR) methylation is linked to resistance against cetuximab [58], the frontline therapy for HNSCC. Additionally, hypoxia can increase PRMT1 and PRMT5 expression [59]. The elevation of 1-methylnicotinamide we observe in our HNSCC data is consistent with published data [18]. 1-methylnicotinamide is produced by nicotinamide N-methyltransferase (NNMT), whose activity is increased in cancer [23] and HNSCC stem cells [60]. NNMT promotes disease progression and is an anticancer target [23]. NNMT activity consumes SAM, which can decrease the “methylation potential” and lead to DNA and histone hypomethylation[23]—potentially relevant given HNSCC’s association with global DNA hypomethylation [61]. The increase in polyamines and their biproduct, 5-methylthioadenosine, we observe in HNSCC suggests elevated polyamine production, consistent with published work [18]. The rise in 5-methylthioadenosine may also reflect MTAP loss, reported in 62% of oral SCC cases [47]. Polyamine synthesis is a therapeutic target in cancers [16], including HNSCC [62] and may negatively regulate SAM metabolic processes, including DNA methylation [63] and 1-carbon unit availability [22]. In hypoxia, polyamine uptake and synthesis increases, and polyamines are able to help cancer cells adapt to hypoxic stress [64].

The cystathionine elevation we observe in HNSCC is consistent with increased methionine cycle activity as it can be derived from homocysteine, a methionine cycle intermediate [31]. Onward cystathionine metabolism can generate cysteine and glutathione via the transulfuration pathway[31, 32], which may help to explain the increased glutathione we observe in HNSCC. The enzyme cystathionine-β-synthase—which converts homocysteine to cystathionine in the transulfuration pathway—is upregulated in cancer [31] and positively regulated by SAM [31]. SAH availability—produced during SAM-dependent methylation– controls the rate of the transulfuration pathway [65], linking our observed increases in cystathionine, glutathione, SAM and SAH. The transfulfuration pathway supports redox balance under nutrient stress in cancer [65] and is an anticancer target [31]. The increase in GSH we observe in HNSCC (while GSSG remains unchanged) indicates an increase in cellular reducing power, which is known to support cancer progression and chemoresistance [32].

In conclusion, SAM, SAH, their metabolic products, and purine metabolites are elevated in HNSCC, particularly in the tumour core, suggesting increased one-carbon production, methionine cycle activity, and downstream SAM metabolism. This potential increase in one-carbon production may involve contributions from THF metabolism, choline, tryptophan and histidine (via carnosine breakdown). Additionally, elevated levels of cystathionine and glutathione in HNSCC suggests an increase of the transulferation pathway downstream of the methionine cycle. Pro-cancer implications of elevated SAM metabolism could include oncogene and oncoprotein expression [15, 56], redox regulation [66], creating a favourable tumour microenvironment [67], drug resistance [58] and immunosuppression [68]. Notably, SAM-related changes were more pronounced in the tumour core, where we also detected elevated lactate, fumarate, malate, and succinate, alongside decreased phosphocreatine—findings consistent with hypoxia or pseudohypoxia and impaired ATP regeneration. Hypoxia is known to upregulate key enzymes in one-carbon and SAM metabolism while promoting polyamine synthesis and N6-methyladenosine RNA methylation, potentially driving the metabolic shifts we observe [10, 11, 50, 53, 64, 68]. The multiple observed SAM metabolic processes that change in HNSCC are metabolically interlinked [19, 22, 23, 46, 57], and it is likely that a balance of these processes is important in the tumour tissue given that SAM has both pro-cancer [19, 20] and anti-cancer [21] effects. Together, these findings highlight SAM metabolism as a metabolically interlinked and potentially targetable vulnerability in HNSCC.

## Supplementary information

Supplementary material - in depth description of all methods used in the paper.

Supplementary Figure S1

Supplementary Figure S2

Supplementary Table S1

Supplementary Table S2

Supplementary Table S3

Supplementary Table S4

Supplementary Table S5

## Data Availability

Data is available at Metabolights (https://www.ebi.ac.uk/metabolights/), study reference MTBLS1905.
