# Supplementary material for: Regional HNSCC metabolomics reveals widespread changes to one-carbon metabolism and S-adenosylmethionine metabolism across tumour core, tumour edge and adjacent non-tumour tissues
Source: Br J Cancer. 2026 Apr 29;135(3):372–81. doi: 10.1038/s41416-026-03410-4 (PMC13372809; doi:10.1038/s41416-026-03410-4)
Supplement: Supplementary file 1 — Supplementary material - in depth description of all methods used in the paper. [file 41416_2026_3410_MOESM1_ESM.docx]

**Regional HNSCC metabolomics reveals widespread changes to one-carbon metabolism and S-adenosylmethionine metabolism across tumour core, tumour edge and adjacent non-tumour tissues – SUPPLEMENTARY INFORMATION**

Andrew D. Southam^1,2*^, James A. Higginson^3,4^, Gavin R. Lloyd^1,2^, Matthew J. Smith^1,5^, Lauren E. Cruchley-Fuge^1,2^, Rachel Spruce^3^, Ossama Edbali^1,2^, Ralf J. M. Weber^1,2^, Hisham Mehanna^3^, Nikolaos Batis^3,6*^

^1^Phenome Centre Birmingham, University of Birmingham, Edgbaston, Birmingham, B15 2TT, UK

^2^School of Biosciences, College of Life and Environmental Sciences, University of Birmingham, Edgbaston, Birmingham, B15 2TT, UK

^3^Institute of Head and Neck Studies and Education (InHANSE), Department of Cancer and Genomic Sciences, College of Medicine and Health, University of Birmingham, B15 2TT, UK

^4^Department of Metabolism, Digestion and Reproduction, Imperial College London, W12 0NN, UK

^5^Unit of Integrative Metabolomics, Institute of Environmental Medicine, Karolinska Institutet, SE-171 77, Stockholm, Sweden

^6^Department of Biomedical Sciences, School of Immunity, Infection and Inflammation, College of Medicine and Health, University of Birmingham, B15 2TT, UK

*email (authors for correspondence): [a.d.southam@bham.ac.uk](mailto:a.d.southam@bham.ac.uk); [n.batis@bham.ac.uk](mailto:n.batis@bham.ac.uk)

**Supplementary Methods**

**Metabolite extraction, process blank preparation, resuspension for UHPLC-MS and QC preparation.** Tissue was extracted using a biphasic methanol/chloroform/water approach. Tissue sample sizes were between 10 – 41.3 mg (with one extra small sample of 6.5 mg). Frozen tissue was homogenised with ice-cold methanol (15 µL/mg wet tissue mass) and ice-cold water (6 µL/mg) using a bead-based homogeniser (Precellys24 and CK14 tubes, Stretton Scientific, UK) for 2 x 10 s bursts of 6400 rpm. The homogenate was transferred to a 2 mL glass vial and ice-cold chloroform (15 µL/mg) and water (7.5 µL/mg) added. Sample was vortexed (60 s), incubated (on ice, 10 min), centrifuged (2,500-g, 10 min, 4°C) and set at room temperature (5 min) to allow biphase partitioning. A fixed volume of the upper biphasic layer containing the extracted polar metabolites was taken from all samples (300 µL – equivalent to 10 mg extracted tissue) except for the small 6.5 mg sample (here 150 µL was taken – equivalent to 5 mg extracted tissue). Samples were dried using a SpeedVac Concentrator (Savant SPD111V230, Thermo Fisher Scientific). Dried polar extracts were resuspended in 100 μL of 3:1 acetonitrile:water (except for the extra small sample, which was resuspended in 50 μL to ensure an equivalent final concentration to the other samples), vortexed (30 s), centrifuged (21,000-rcf, 20 min, 4°C) and 50 μL of the supernatant was aliquoted into a low recovery volume HPLC vial (Chromatography Direct, UK). Vials were set at 4⁰C until and during UHPLC-MS analysis.

**Extract blank and quality control (QC) sample preparation.** Extract blank samples were prepared by the method written above but in the absence of tissue and using solvent volumes equivalent to those used for a 20 mg tissue piece. A pooled QC sample was created by pooling 40 μL of each resuspended sample (except for the extra small sample) after final centrifugation step. The QC pool was vortexed (60 s) and 50 μL of this was aliquoted into multiple low recovery volume HPLC vials. Vials were set at 4⁰C until and during UHPLC-MS analysis.

**UHPLC-MS analysis – main study data collection**. A hydrophilic interaction chromatography (HILIC) based Ultra-High Performance Liquid Chromatography-Mass Spectrometry (UHPLC-MS) assay was applied as used previously^1^ using an Accucore-150-Amide-HILIC column (100 x 2.1 mm, 2.6 μm, Thermo Fisher Scientific, MA, USA). Mobile phase A was 95% acetonitrile/water (10 mM ammonium formate, 0.1% formic acid); mobile phase B was 50% acetonitrile/water (10 mM ammonium formate, 0.1% formic acid); and the gradient was t=0.0, 1% B; t=1.0, 1% B; t=3.0, 15% B; t=6.0, 50% B; t=9.0, 95% B; t=10.0, 95% B; t=10.5, 1% B; t=14.0, 1% B. All changes were linear (curve = 5) and the flow rate was 0.50 mL/min. Column temperature was 35 °C and injection volume was 2 μL. Data were acquired separately in positive and negative ionisation modes (70 – 1050 m/z) with a resolution of 70,000 (FWHM at m/z 200). Ion source parameters: Sheath gas = 53 arbitrary units, Aux gas = 14 arbitrary units, Sweep gas = 3 arbitrary units, Spray Voltage = 3.5kV (positive ion) / 2.7kV (negative ion), Capillary temp. = 269 °C (positive ion) / 320 °C (negative ion), Aux gas heater temp. = 438°C (positive ion) / 320 °C (negative ion). Quality control (QC) samples were analysed as column equilibration samples (the first eight injections). QC samples were recorded throughout the run to check for analytical quality: a further 2 QC samples at the beginning of the run, then a QC analysed every seventh injection and 2 QCs at the end of the analytical batch. Extract blank samples was analysed as the 3^rd^ and last injections of the analytical batch. For the purpose of metabolite annotation, data dependent MS2 in ‘Discovery mode’ was applied to five QC samples over five mass ranges (70 – 120 m/z; 120-170 m/z; 170-220 m/z; 220-270 m/z; 270-1050 m/z) using following settings: MS1 resolution = 35,000, MS2 resolution = 17,500; Isolation width = 3.0 m/z; stepped collision energies = 25, 60, 100 eV (named ‘original data collection’). To improve the detection & metabolite annotation of key metabolites, authentic chemical standards and repeated acquisitions of biological QC samples were collected in both positive and negative ion modes using stepped normalised collision energies (NCE) of 20, 40, 130% (named ‘follow-up data’ collection). Some standard compounds (carnitine, 1-methylnicotinamide, guanine, phosphocholine) were not sufficiently fragmented at NCE20, 40, 130%; so, these compounds – along with biological QC samples – were fragmented with higher energies (NCE40, 80, 130%). The fragmentation energies used for each compound is indicated in Figure S1. In all cases the chromatography was the same.

**UHPLC-MS negative ion analysis of lactate, fumarate, pyruvate, succinate and malate**. The UHPLC-MS method described in the previous section was not optimal for detecting key organic acids that indicate (pseudo)hypoxia and TCA cycle dysregulation – lactate, fumarate, pyruvate, succinate, and malate. To accurately detect these metabolites, we employed a dedicated method specifically optimised for their analysis. Samples were analysed using a Transcend Vanquish Flex Duo LX-2 UHPLC system coupled with a heated electrospray Orbitrap Exploris 120 mass spectrometer (Thermo Fisher Scientific) using an ACQUITY Premier BEH Amide VanGuard FIT Column (1.7µ, 2.1x150mm, Waters). For negative ion analysis, mobile phase A was 10 mM ammonium acetate (taken from a 200 mM stock at pH 9.0; pH adjusted with a 28% ammonium hydroxide aqueous solution, Honeywell) dissolved in acetonitrile/water (95:5 [v/v]) and mobile phase B was 10 mM ammonium acetate (taken from a 200 mM stock at pH 9.0; pH adjusted with ammonium hydroxide) dissolved in acetonitrile/water (50/50 [v/v]). The gradient elution applied was t=0.0, 1% B; t=1.0, 1% B; t=1.1, 15% B; t=4.5, 35% B; t=8.25, 80% B; t=8.5, 95% B; t=10.0, 99% B; t=10.75, 1% B; t=16.0, 1% B. All changes were linear and the flow rate was 0.35 mL/min. Column temperature was 40°C and injection volume was 2μL. Data were acquired in negative ionisation mode only (70 – 700 m/z) with a mass resolution 120,000 (FWHM, *m/z* 200). Ion source parameters: Sheath gas = 35 arbitrary units, Aux gas = 7 arbitrary units, Sweep gas = 0 arbitrary units, Spray Voltage = 2.5kV, Ion transfer tube temp. = 320°C, Vapourizer temp. = 275°C. The software controlling the instruments and data acquisition were: Orbitrap Exploris 120 TuneApplication (v4.3.458.15); Thermo Scientific Xcalibur (v4.7.69.37, 2023), and Aria MX (2020). All data were acquired in profile mode. For metabolite annotation authentic chemical standards and biological QC samples were collected in negative ion modes using stepped normalised collision energies (NCE) of 15, 30, 60%. Some standard compounds were not sufficiently fragmented at NCE15, 30, 60%; so, these compounds – along with biological QC samples – were fragmented with higher energies: lactate – NCE80, 120, 180%; pyruvate – 40, 80, 120%. The fragmentation energies used for each compound is indicated in Figure S1. In all cases the chromatography was the same.

**Raw data processing and filtering using QC samples (main study).** Data was processed as previously reported^1-3^ with the MS^1^ data used for peak area integration measurements and the MS^2^ data used for annotation purposes only. QC data showed slight UHPLC-MS intensity drift, so data was signal corrected^4^. Processing of the MS^1^ data was carried out as follows: vendor format raw data files (.RAW) were converted to the mzML file format using ProteoWizard software^5^. Deconvolution was performed by XCMS software^6^ (version 1.46.0 running in the Galaxy environment^7^) applying min peak width (6); max peak width (30); ppm (14); mzdiff (0.001); bw (0.25); mzwid (0.01); minfrac (0.5). A data matrix of peak areas for metabolite features (m/z-retention time pairs) vs. samples was constructed. Features were retained in the data matrix if they were: present in >90% of QC samples; had a peak intensity relative standard deviation (RSD)<30% across QC samples; and had a mean QC/extract blank intensity ratio of >20% and present in >50% of the samples. Samples were excluded if more than 50% of the features were missing – only one example of this existed – 1x non-tumour (N) sample was removed from the UHPLC-MS HILIC negative ion data set. All signal correction and filtering steps were applied using R version 4.1.1^8^ and the structToolbox package^3, 9^. To evaluate and correct for systematic mass error across the m/z range, 80 known reference features consistently present across the study samples were used. These included sodium formate clusters and endogenous metabolites detected in >80% of samples—such as riboflavin, 9-HODE, guanosine, fructose-6-phosphate, and L-tyrosine—spanning an m/z range of 128 to 1000. A cubic smoothing spline was first fitted to the m/z error as a function of m/z using a randomly selected 50% subset of reference features, to correct for variations in instrument accuracy across the mass range. The model was then validated on the remaining 50% of reference features. The same procedure was repeated for m/z error as a function of injection order to correct for temporal mass drift. After validating both models, the corrections were applied sequentially to the entire dataset (HILIC negative ion data ONLY), including precursor m/z values from LC-MS/MS spectra used for metabolite annotation.

**Raw data processing for the lactate, fumarate, pyruvate, succinate and malate data.** Peak integration was performed using Thermo Fisher Quan Browser, which operates within the Xcalibur software environment (version 4.6.67.17, release date 03/08/2022). The ICIS peak detection algorithm was applied for chromatographic peak identification and integration. Integration parameters were set as follows: smoothing points of 1, baseline window of 500, area noise factor of 5, peak noise factor of 10, peak m/z error of 5 ppm, and a minimum peak height corresponding to a signal-to-noise ratio of 3. The RSD of each of the 5 compounds was calculated across the QC samples and each compound met our quality criteria of QC RSD<30%. Finally, peak areas were normalised to total spectral area.

**Metabolite annotation.** For metabolite annotation and identification, two complementary strategies were employed. First, experimental retention times (RTs) and/or MS/MS spectra were matched against authentic chemical standards. Second, when standard-derived MS/MS spectra were unavailable, experimental MS/MS data were compared to the mzCloud spectral library (mzcloud.org) or other published works. Prior to spectral matching, MS/MS spectra were cleaned to reduce noise and improve reliability^10^. More specifically, fragment peaks with relative intensities below 3% were treated as noise and removed. In each 0.01 Da m/z bin, the weakest peak was also removed. If a spectrum showed minimal variation in its interquartile range or below its median, any peaks falling beneath the 75th percentile or median, respectively, were treated as noise and removed. The method then kept the precursor ion (when present) and the three strongest fragment ions. This adaptive filtering process removes weak signals while keeping the most important ions for accurate spectral matching. Spectral similarity matching was performed using the normalised dot-product cosine (DPC) metric, and annotations with scores ≥0.65 were retained. RT alignment to authentic chemical standards required agreement within ±20 seconds (for either the ‘original’ or ‘follow-up’ data acquisition).

For the annotated compounds in Table S3 (i.e., one-carbon–related metabolites), when multiple ion forms were detected, we report the most abundant ion with the fewest missing values (typically [M+H]^+^ or [M–H]^–^). The metabolite annotation grade, based on the Metabolomics Standards Initiative (MSI), is provided for each compound in the results tables.

Four metabolites with DPC scores slightly below the 0.65 threshold were retained for the following reasons:

1. **Dimethylglycine:** A nearby m/z peak at the same retention time was likely co-isolated and contributed extra fragments, but the experimental MS² still contained the key diagnostic library fragment.
2. **Methylhistamine:** A similar co-isolated peak likely added extra fragments, yet the experimental MS² retained the essential library-matching ions.
3. **CDP-choline:** Despite a methodological difference (stepped vs. single-collision energy), the spectrum visually matched all major library fragments (5 ions) with a DPC of 0.63.
4. **N-acetylcarnosine:** A nearby co-isolated ion likely produced additional fragments, but the defining library fragment was present in the experimental MS².

Additional annotation and quality details are provided in Tables S1, S3, S5, and Figure S1. Figures have been generated using the R package lcmsPlot (<https://github.com/computational-metabolomics/lcmsplot>).

**Statistics and pathway analysis.** Considering the main study data: After quality filtering, the peak tables were prepared for statistical analysis using Probabilistic Quotient Normalisation (PQN; mean QC as reference)^11^. For multivariate analysis the following additional steps were applied: *k*-nearest neighbour imputation (k = 5)^12, 13^; generalised log transform (QC as reference)^14, 15^. Univariate analysis (ANOVA and post-hoc testing) and multivariate analysis (principal components analysis, PCA^16, 17^, and partial least squares discriminant analysis, PLS-DA])^18, 19^ were then applied using the processed peak tables. The PLS-DA model was trained on two-thirds of the data and validated on the remaining on-third, with 10-fold cross validation. All processing step and statistical analysis was conducted using R v4.1.1^8^ and the structToolbox package^3, 9^. Considering box and whisker plots of individual metabolites, standard Tukey versions were used with the box covering the lower to upper quartile, the midline is the median, and the whiskers are 1.5x the interquartile range. Pathway analysis of all annotated significantly changing metabolites was conducted in MetaboAnalyst 6.0 (<https://www.metaboanalyst.ca/>)^20^. A hypergeometric test was used as the enrichment method, relative-betweeness centrality used as the topology measure, and the reference metabolome was the homo sapiens pathway library in KEGG).

Considering the lactate, fumarate, pyruvate, succinate and malate data univariate analysis (ANOVA and Tukey HSD post-hoc testing) was applied to the normalised peak areas. For the lactate/pyruvate ratio data, the ratio was log transformed and tested with a non-parametric test (Kruskal-Wallis and Dunn post hoc).

Note: Statistical tests were justified based on the study design. Parametric one-way ANOVA was used as the primary test because most metabolite variables are expected to follow a normal distribution, and ANOVA provides greater statistical power. To ensure robustness, all metabolites identified as significant by ANOVA were re-tested using the non-parametric Kruskal–Wallis test with FDR correction. Results from both approaches were highly consistent (Supplementary Tables S1 and S3).

**Correlation analysis.** Metabolites related to one-carbon metabolism were annotated/identified within the dataset regardless of their significance status. Spearman’s Rank correlation was applied to the responses of these metabolites separately within either: (a) non-tumour tissue, (b) tumour edge tissue or (c) core tumour tissue. p-values for Spearman Rank correlation were False Discovery corrected (q<0.05) within each tissue type to account for multiple comparisons. Upper and lower 95% confidence limits (defined in plots by error bars) were calculated using a Fisher transform approach (see: <https://stats.stackexchange.com/questions/18887/how-to-calculate-a-confidence-interval-for-spearmans-rank-correlation>).

**References**

(1) Southam, A. D.; Pursell, H.; Frigerio, G.; Jankevics, A.; Weber, R. J. M.; Dunn, W. B. Characterization of Monophasic Solvent-Based Tissue Extractions for the Detection of Polar Metabolites and Lipids Applying Ultrahigh-Performance Liquid Chromatography-Mass Spectrometry Clinical Metabolic Phenotyping Assays. *J Proteome Res* **2021**, *20* (1), 831-840. DOI: 10.1021/acs.jproteome.0c00660 From NLM.

(2) Jankevics, A.; Lloyd, G. R.; Weber, R. J. M. pmp: Peak Matrix Processing and signal batch correction for metabolomics datasets. *Bioconductor* **2025**. DOI: 10.18129/B9.bioc.pmp, R package version 1.20.0, <https://bioconductor.org/packages/pmp>.

(3) Lloyd, G. R.; Jankevics, A.; Weber, R. J. M. struct: an R/Bioconductor-based framework for standardized metabolomics data analysis and beyond. *Bioinformatics* **2021**, *36* (22-23), 5551-5552. DOI: 10.1093/bioinformatics/btaa1031 From NLM.

(4) Kirwan, J. A.; Broadhurst, D. I.; Davidson, R. L.; Viant, M. R. Characterising and correcting batch variation in an automated direct infusion mass spectrometry (DIMS) metabolomics workflow. *Analytical and Bioanalytical Chemistry* **2013**, *405* (15), 5147-5157. DOI: 10.1007/s00216-013-6856-7.

(5) Kessner, D.; Chambers, M.; Burke, R.; Agus, D.; Mallick, P. ProteoWizard: open source software for rapid proteomics tools development. *Bioinformatics* **2008**, *24* (21), 2534-2536. DOI: 10.1093/bioinformatics/btn323 From NLM.

(6) Smith, C. A.; Want, E. J.; O'Maille, G.; Abagyan, R.; Siuzdak, G. XCMS: processing mass spectrometry data for metabolite profiling using nonlinear peak alignment, matching, and identification. *Anal Chem* **2006**, *78* (3), 779-787. DOI: 10.1021/ac051437y From NLM.

(7) Davidson, R. L.; Weber, R. J. M.; Liu, H.; Sharma-Oates, A.; Viant, M. R. Galaxy-M: a Galaxy workflow for processing and analyzing direct infusion and liquid chromatography mass spectrometry-based metabolomics data. *Gigascience* **2016**, *5*, s13742–13016–10115–13748. DOI: 10.1186/s13742-016-0115-8.

(8) Team, R. C. R: A language and environment for statistical computing. R Foundation for Statistical Computing, Vienna, Austria. URL: <https://www.R-project.org/>. **2021**.

(9) Lloyd, G. R.; Weber, R. J. M. structToolbox: Data processing & analysis tools for Metabolomics and other omics. *Bioconductor* **2020**. DOI: 10.18129/B9.bioc.structToolbox.

(10) Dalla Valle, N.; Garcia-Aloy, M.; Robatscher, P.; Franceschi, P.; Oberhuber, M. Improving Spectral Similarity and Molecular Network Reliability through Noise Signal Filtering in MS/MS Spectra. *Analytical Chemistry* **2025**, *97* (29), 15873-15882. DOI: 10.1021/acs.analchem.5c02109.

(11) Dieterle, F.; Ross, A.; Schlotterbeck, G.; Senn, H. Probabilistic quotient normalization as robust method to account for dilution of complex biological mixtures. Application in H-1 NMR metabonomics. *Analytical Chemistry* **2006**, *78* (13), 4281-4290. DOI: 10.1021/ac051632c.

(12) Gromski, P. S.; Xu, Y.; Kotze, H. L.; Correa, E.; Ellis, D. I.; Armitage, E. G.; Turner, M. L.; Goodacre, R. Influence of missing values substitutes on multivariate analysis of metabolomics data. *Metabolites* **2014**, *4* (2), 433-452. DOI: 10.3390/metabo4020433 From NLM.

(13) Troyanskaya, O.; Cantor, M.; Sherlock, G.; Brown, P.; Hastie, T.; Tibshirani, R.; Botstein, D.; Altman, R. B. Missing value estimation methods for DNA microarrays. *Bioinformatics* **2001**, *17* (6), 520-525. DOI: 10.1093/bioinformatics/17.6.520.

(14) Durbin, B. P.; Hardin, J. S.; Hawkins, D. M.; Rocke, D. M. A variance-stabilizing transformation for gene-expression microarray data. *Bioinformatics* **2002**, *18 Suppl 1*, S105-110. DOI: 10.1093/bioinformatics/18.suppl_1.s105 From NLM.

(15) Parsons, H. M.; Ludwig, C.; Gunther, U. L.; Viant, M. R. Improved classification accuracy in 1-and 2-dimensional NMR metabolomics data using the variance stabilising generalised logarithm transformation. *BMC Bioinformatics* **2007**, *8*, 16, Article. DOI: 10.1186/1471-2105-8-234.

(16) Brereton, R. G. Introduction. In *Data Analysis and Chemometrics for Metabolomics*, 2024; pp 1-25.

(17) Jolliffe, I. Principal Component Analysis. In *Wiley StatsRef: Statistics Reference Online*.

(18) Barker, M.; Rayens, W. Partial least squares for discrimination. *Journal of Chemometrics* **2003**, *17* (3), 166-173. DOI: <https://doi.org/10.1002/cem.785>.

(19) Brereton, R. G.; Lloyd, G. R. Partial least squares discriminant analysis: taking the magic away. *Journal of Chemometrics* **2014**, *28* (4), 213-225. DOI: <https://doi.org/10.1002/cem.2609>.

(20) Pang, Z.; Lu, Y.; Zhou, G.; Hui, F.; Xu, L.; Viau, C.; Spigelman, Aliya F.; MacDonald, Patrick E.; Wishart, David S.; Li, S.; et al. MetaboAnalyst 6.0: towards a unified platform for metabolomics data processing, analysis and interpretation. *Nucleic Acids Research* **2024**, *52* (W1), W398-W406. DOI: 10.1093/nar/gkae253 (acccessed 4/22/2025).
