# Supplementary material for: Regional HNSCC metabolomics reveals widespread changes to one-carbon metabolism and S-adenosylmethionine metabolism across tumour core, tumour edge and adjacent non-tumour tissues
Source: Br J Cancer. 2026 Apr 29;135(3):372–81. doi: 10.1038/s41416-026-03410-4 (PMC13372809; doi:10.1038/s41416-026-03410-4)

**Supplementary Figure S1:** Metabolite annotation and identification. Spectral data supporting metabolite identification for metabolites listed in Supplementary Tables S1 and S3. Where available, these include retention time, MS/MS spectra, and matches to our in-house library or online repositories (e.g., mzCloud). For each metabolite, normalised stepped HCD MS/MS energies and $MS^2$ match scores are shown when corresponding data exist. The data-collection type is indicated: ‘original’ refers to acquisition during the primary study, and ‘follow-up’ denotes re-acquisition performed to obtain higher-quality $MS^2$ data when the original data collection was insufficient.

# 1-methyladenosine [M+H]+ | HMDB0003331

Positive ion mode: 282.1197 m/z | Instrument: QE focus

## Chromatogram

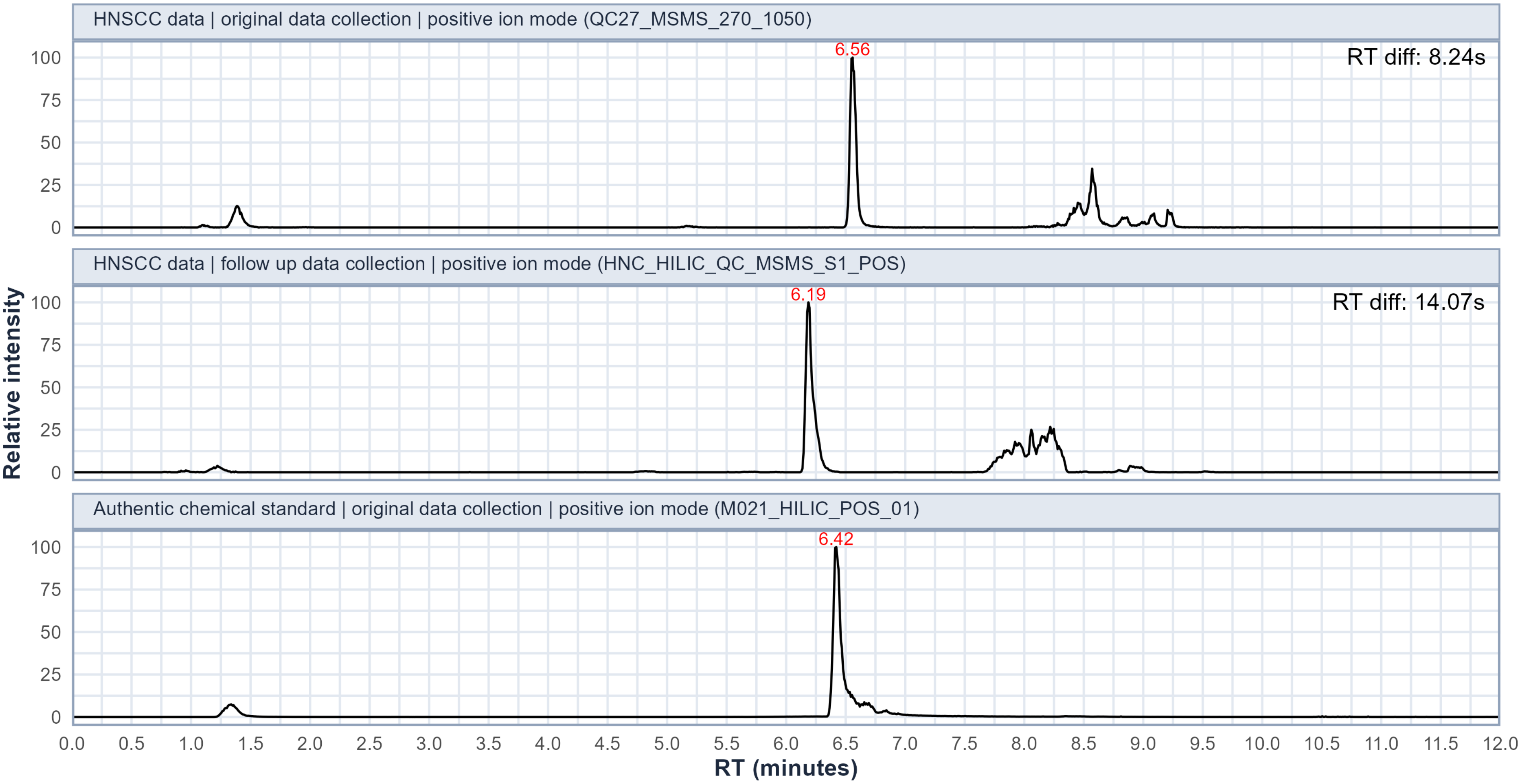


## MS/MS

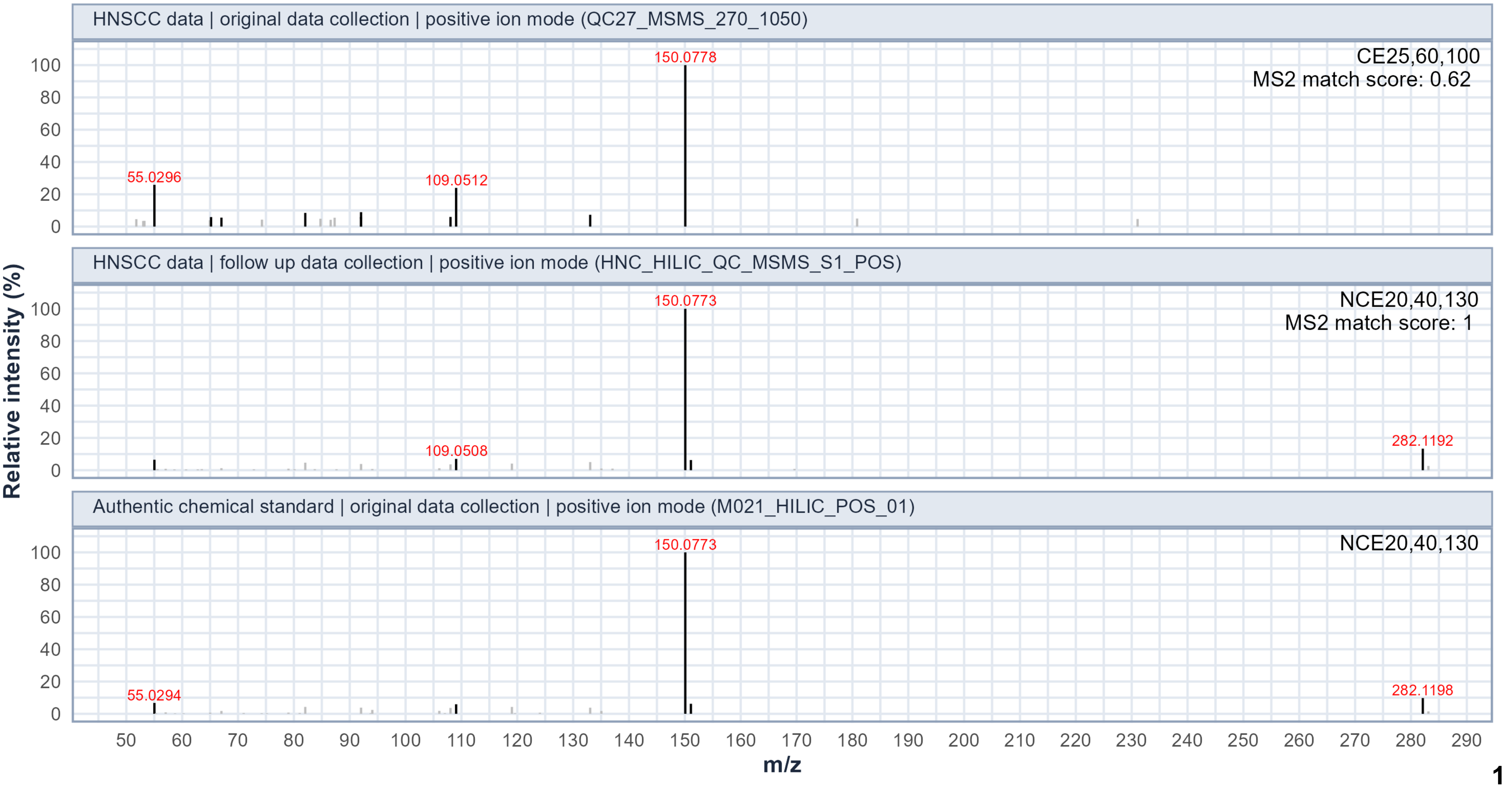

# 1-methylnicotinamide [M-e]+ | HMDB0000699

Positive ion mode: 137.0709 m/z | Instrument: QE focus

## Chromatogram

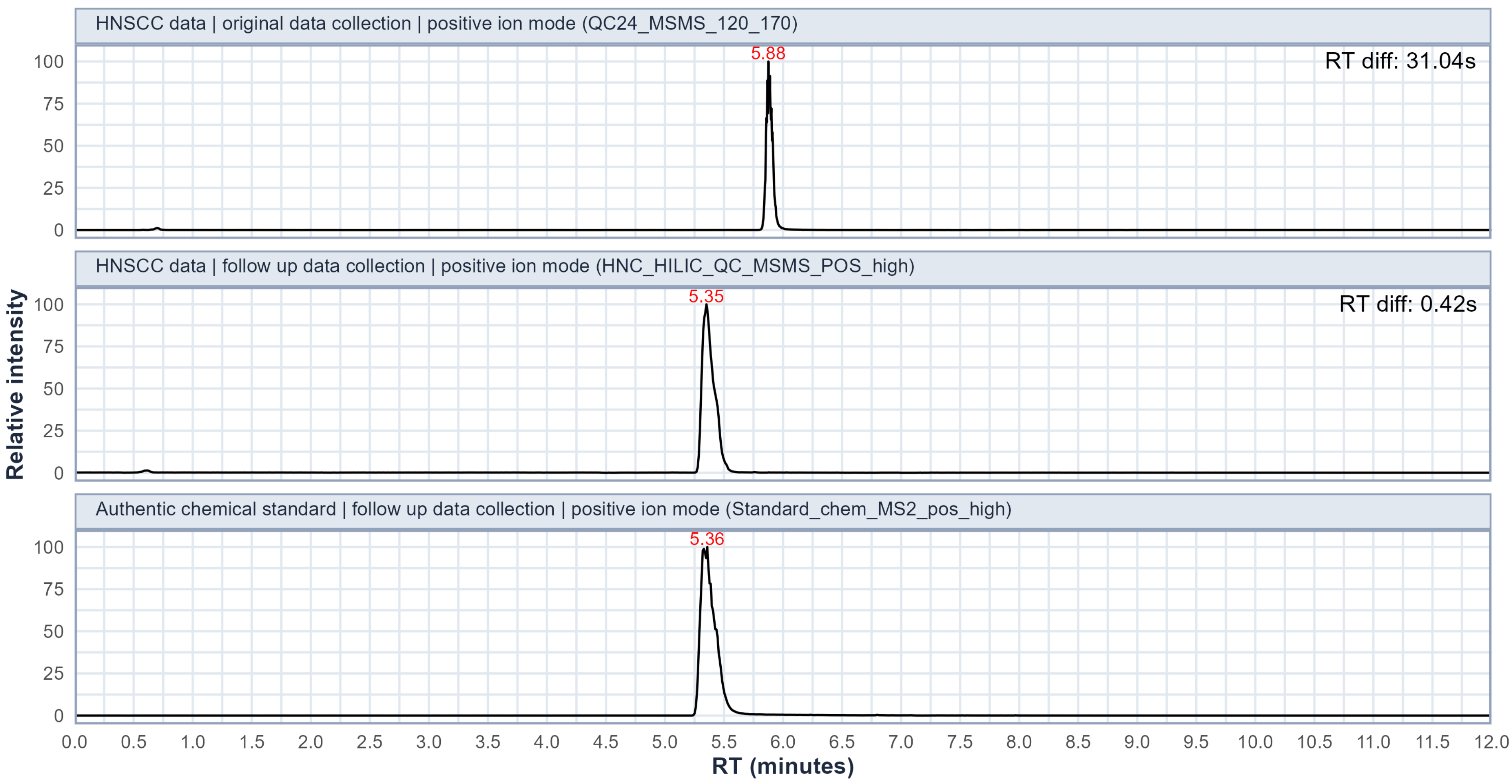


## MS/MS

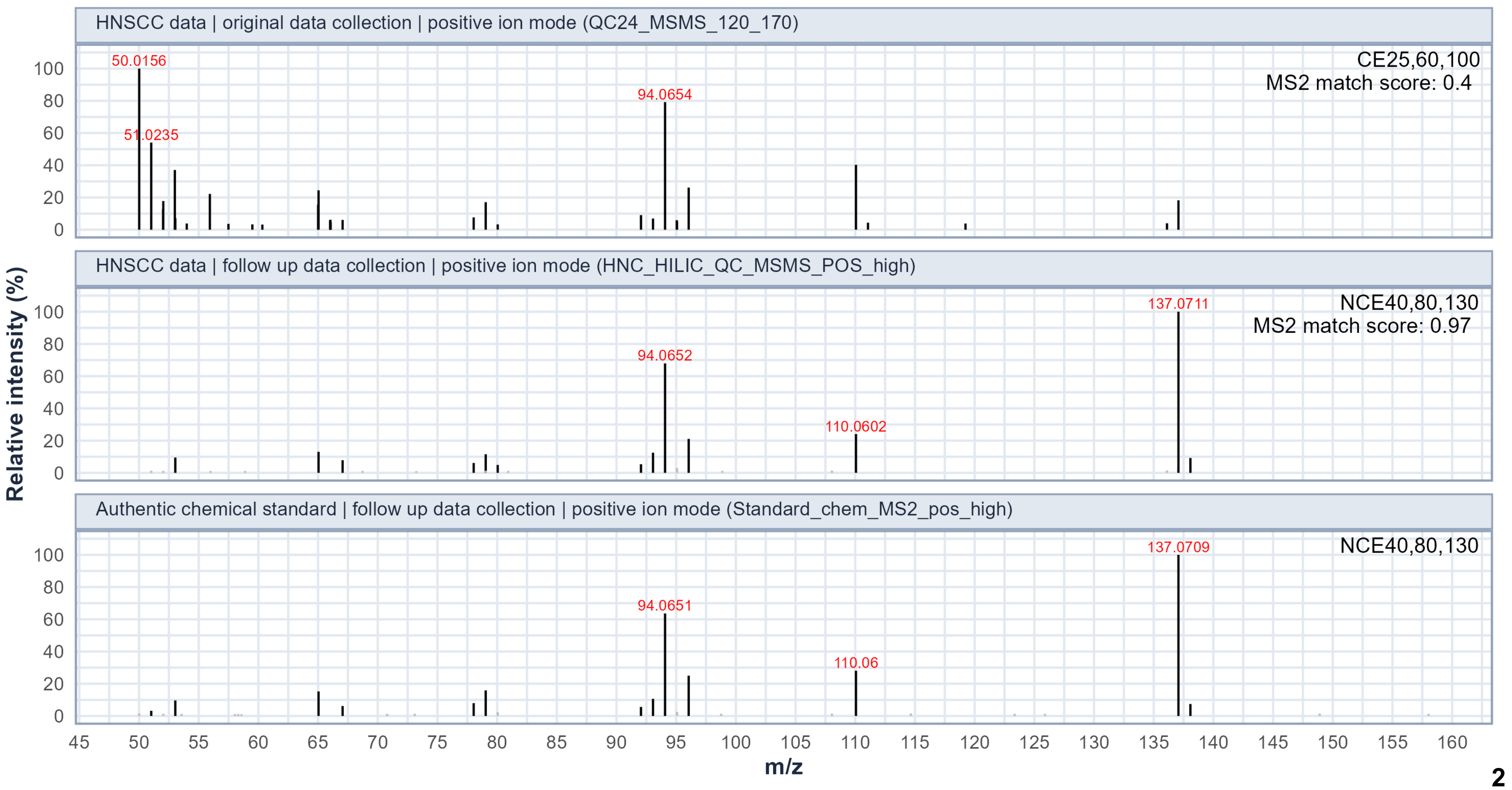

# 5'-Methylthioadenosine [M+H]+ | HMDB0001173

Positive ion mode: 298.0968 m/z | Instrument: QE focus

## Chromatogram

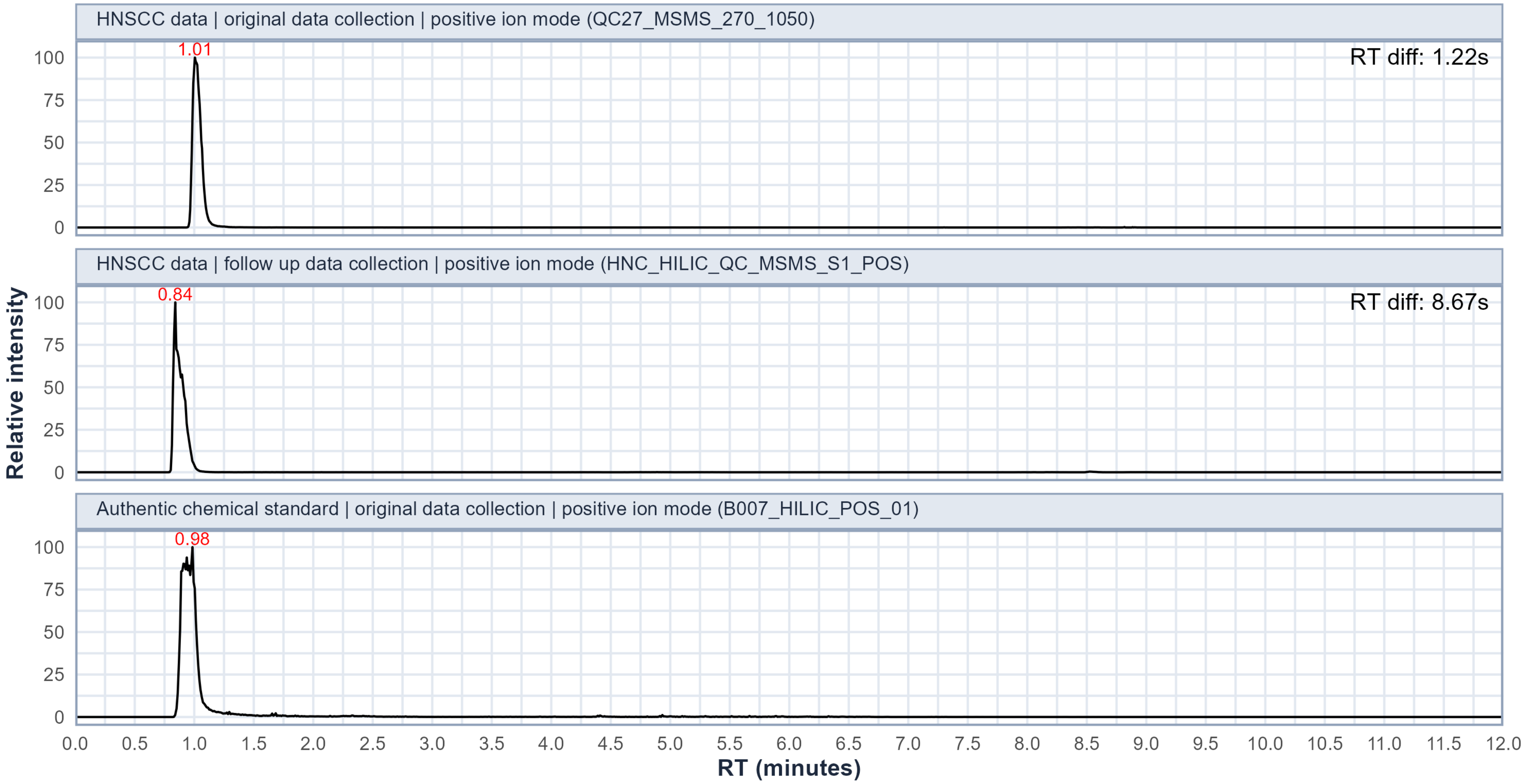


## MS/MS

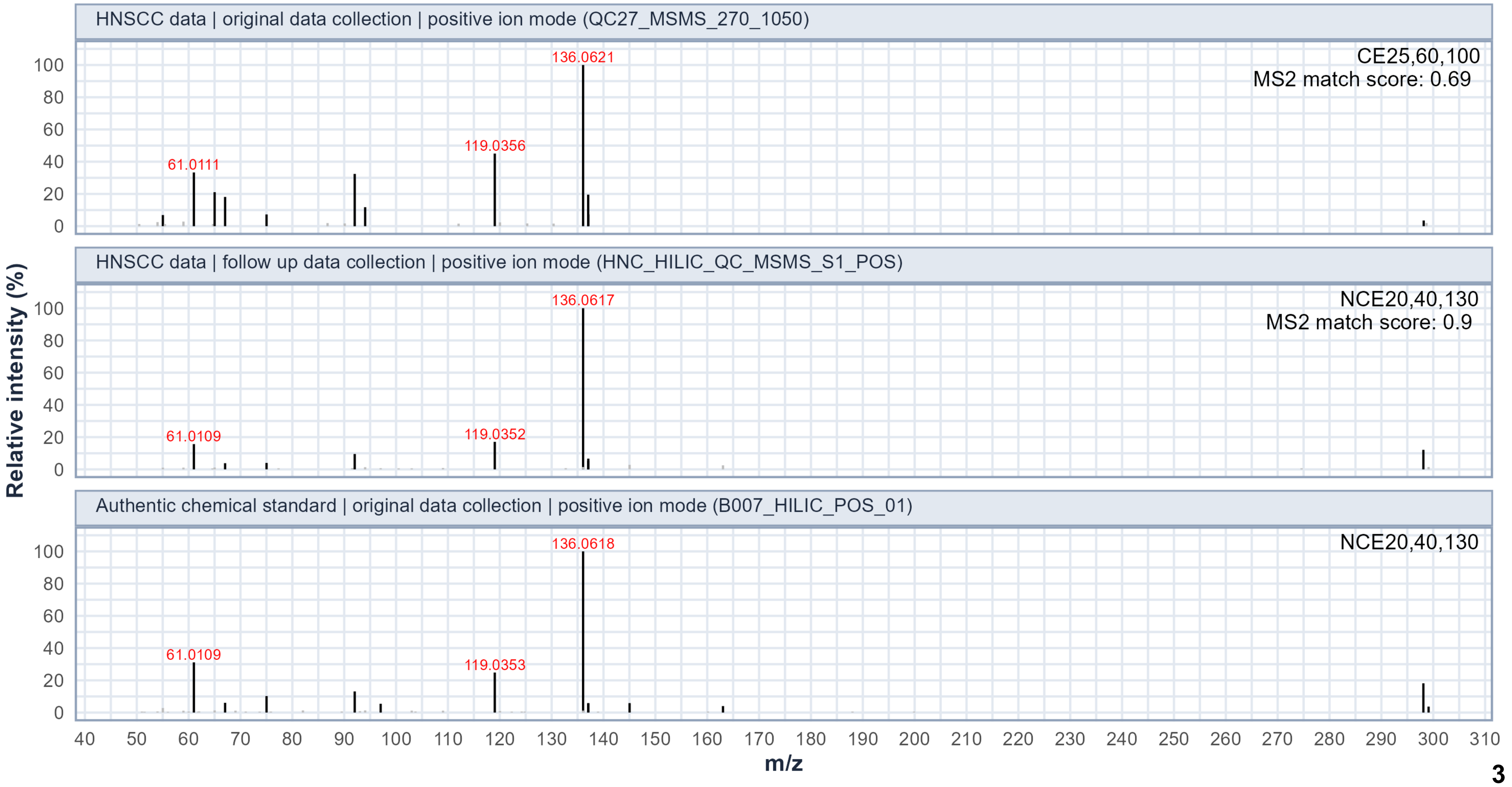

# Adenosine [M+H]+ | HMDB0000050

Positive ion mode: 268.104 m/z | Instrument: QE focus

## Chromatogram

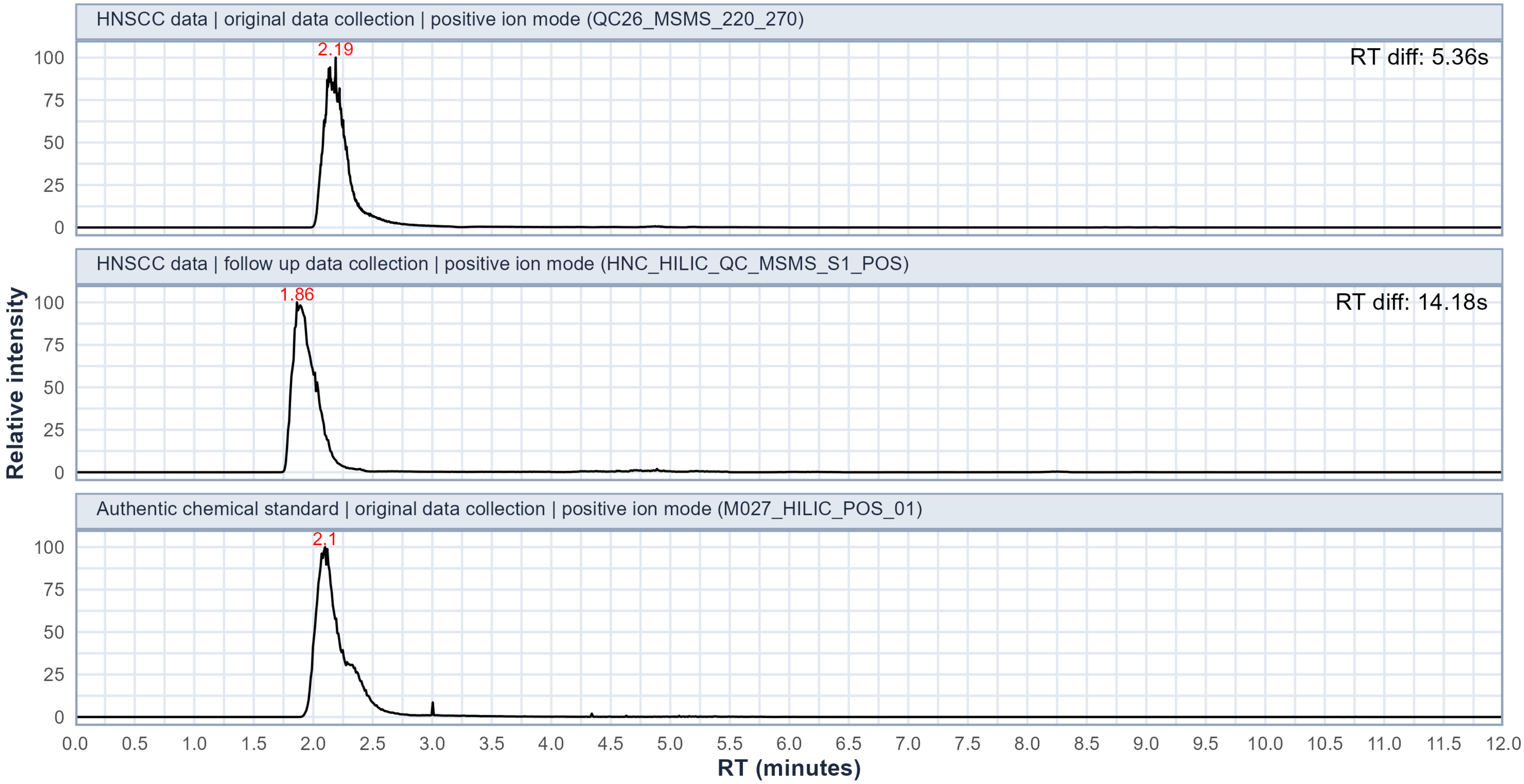


## MS/MS

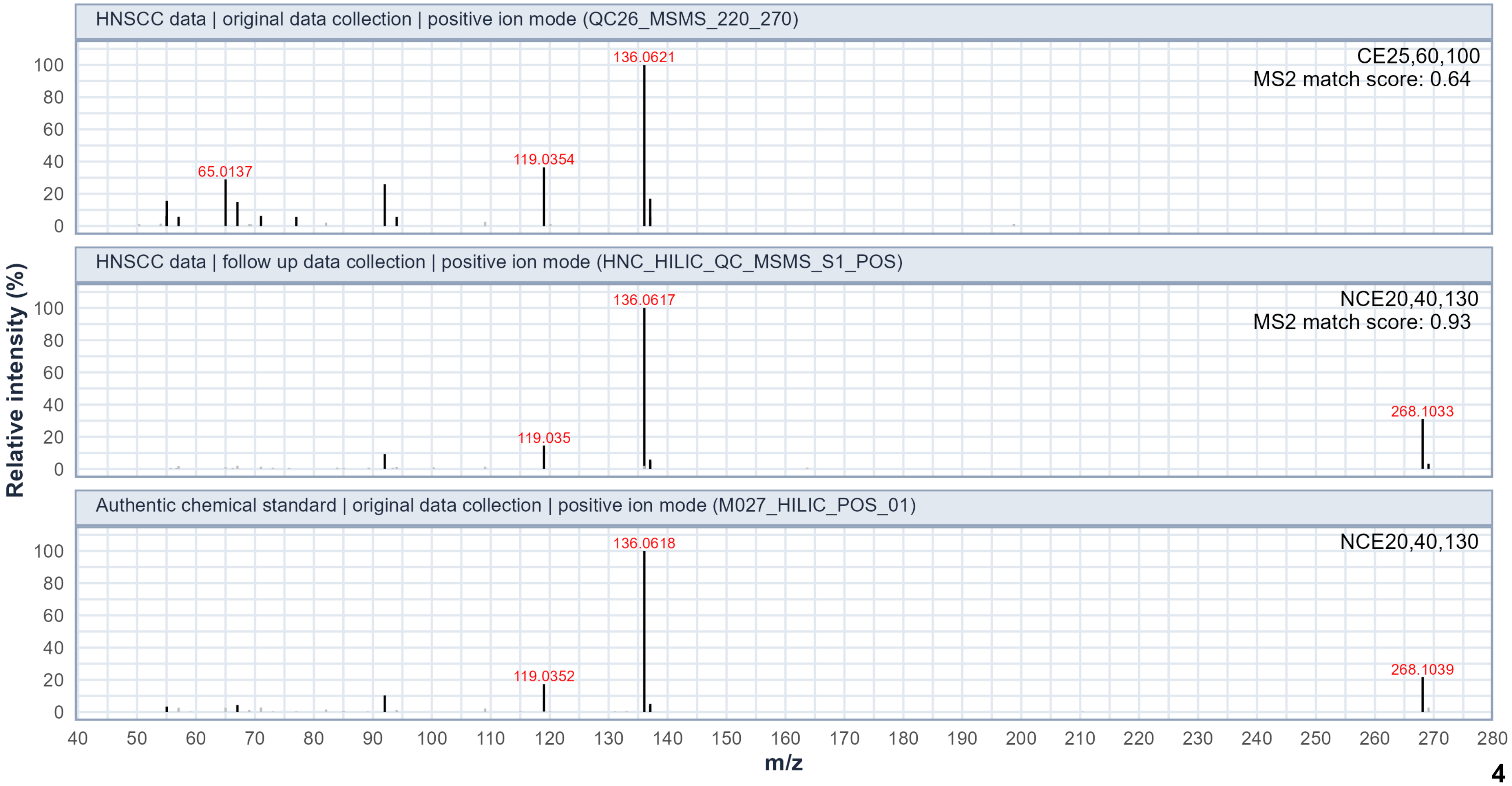

# Adenosine monophosphate, AMP [M-H]- | HMDB0000045

Negative ion mode: 346.0558 m/z | Instrument: QE focus

## Chromatogram

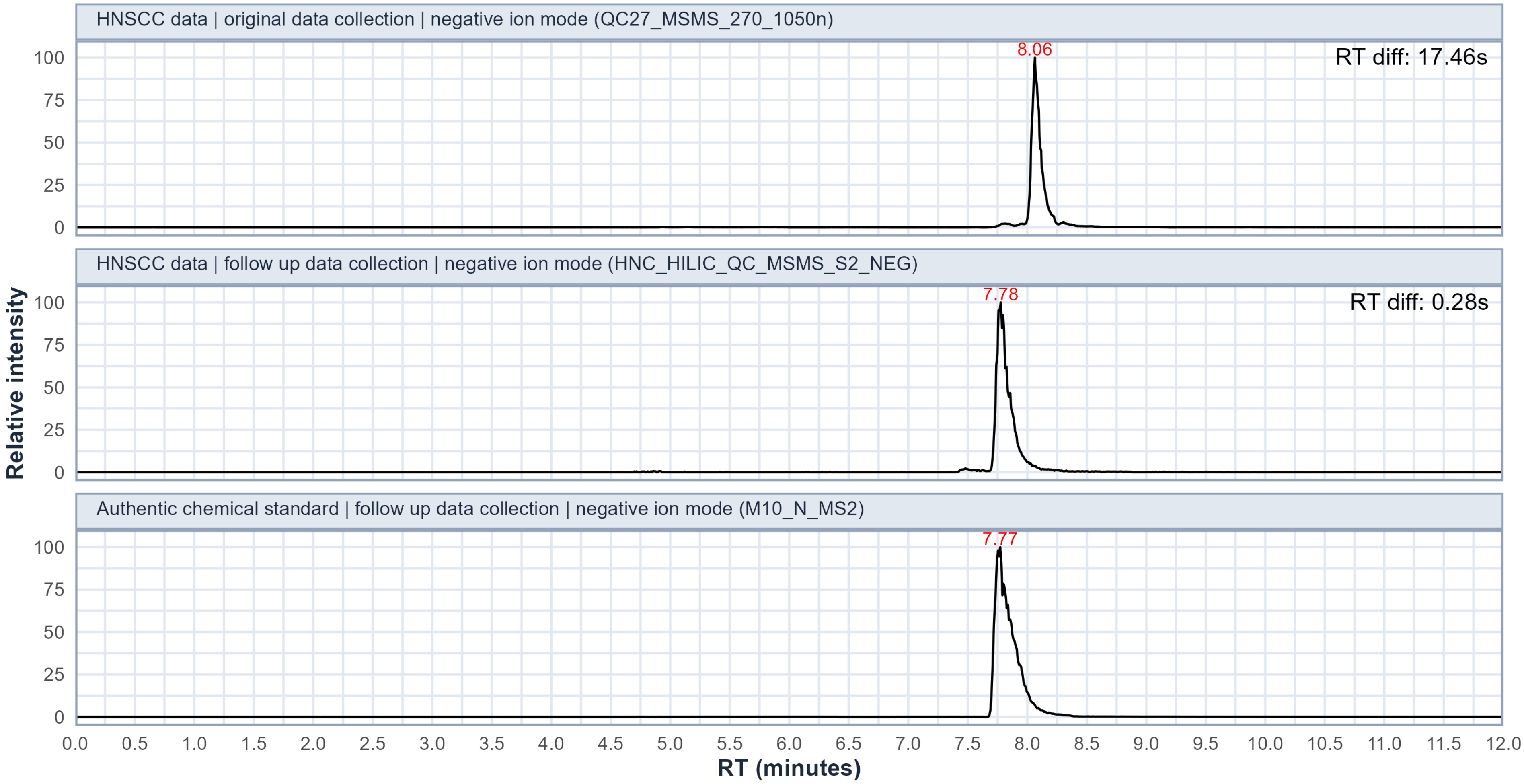


## MS/MS

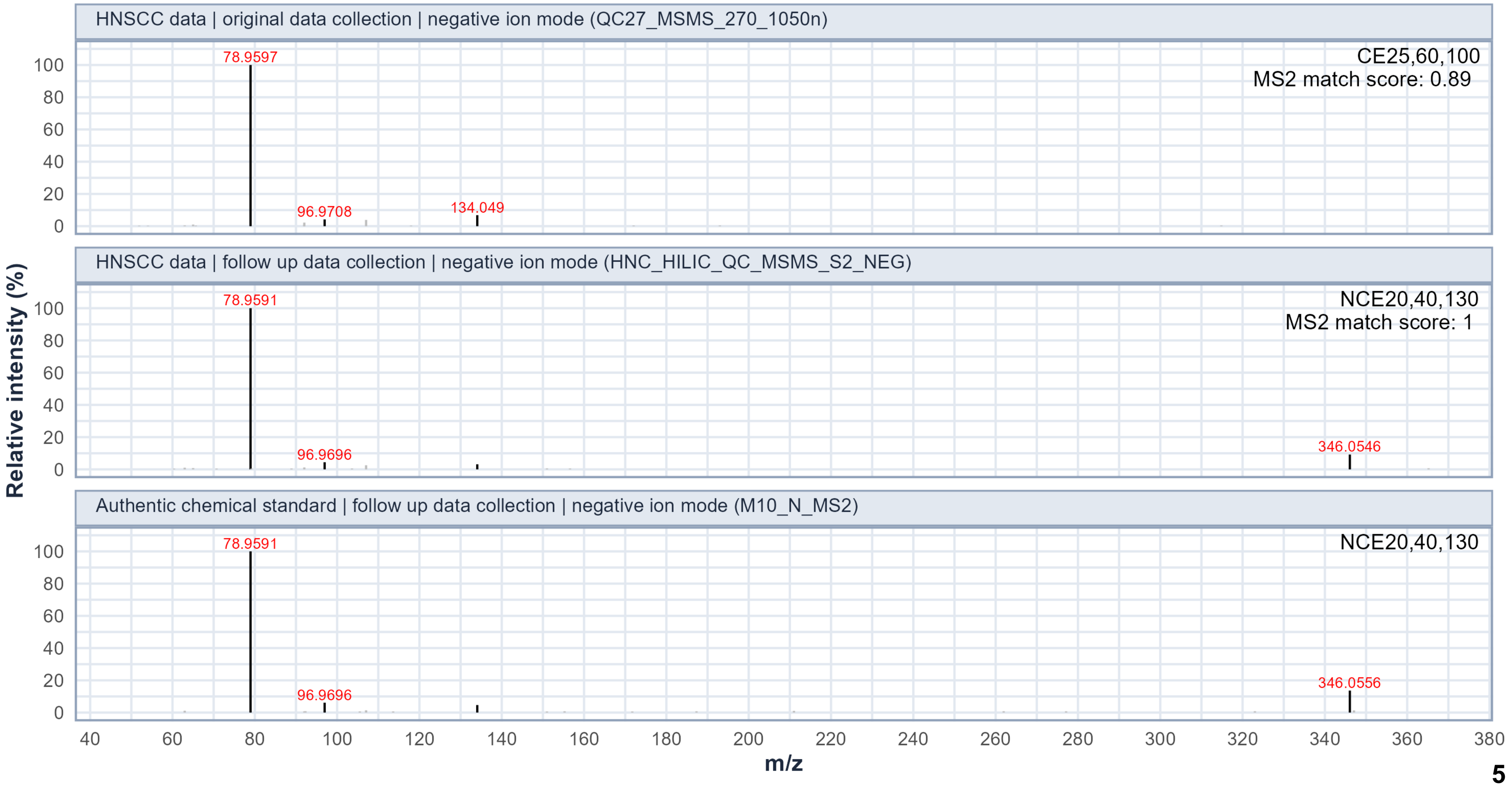

# Allantoin [M-H]- | HMDB0000462

Negative ion mode: 157.0367 m/z | Instrument: QE focus

## Chromatogram

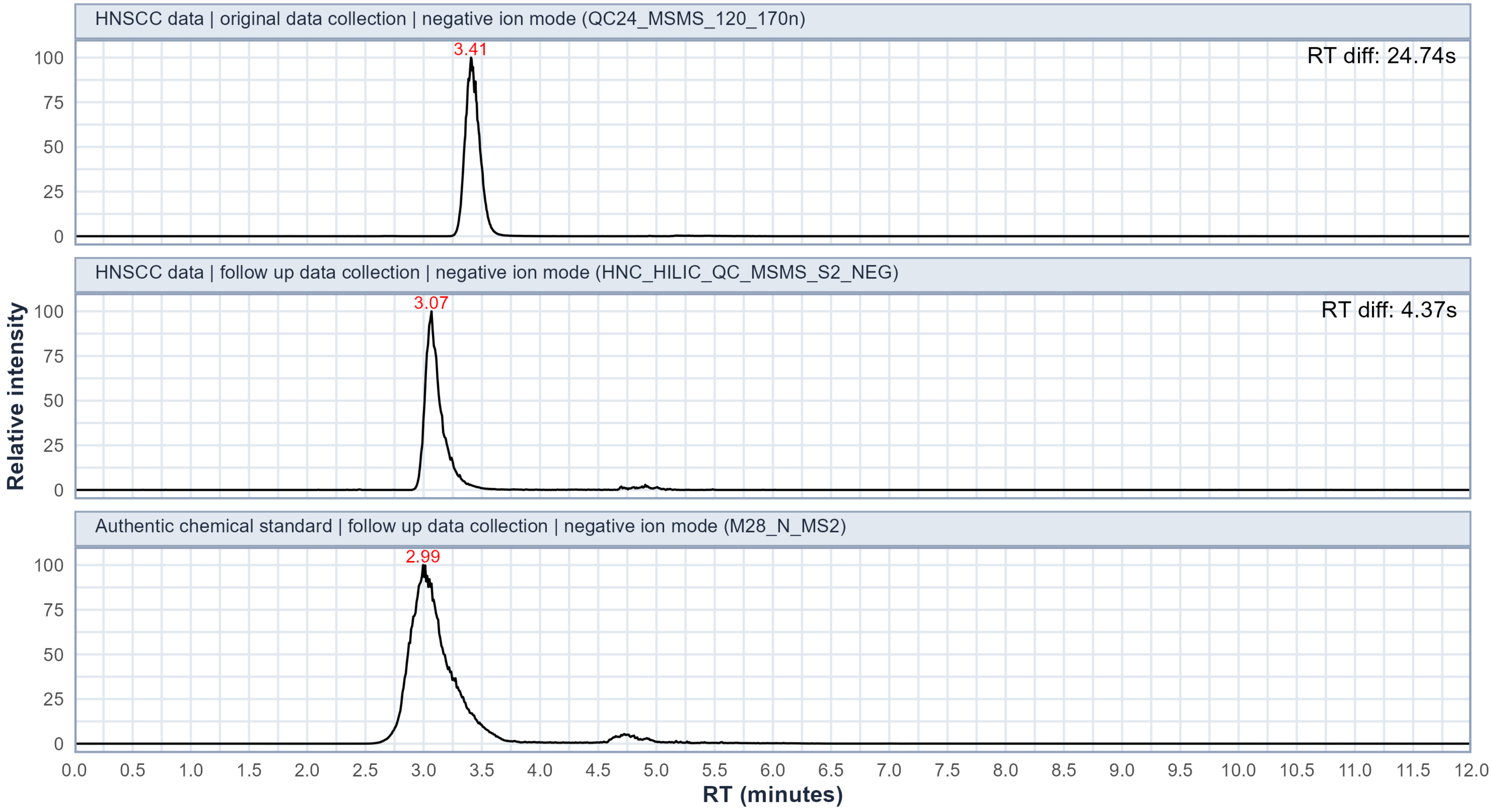


## MS/MS

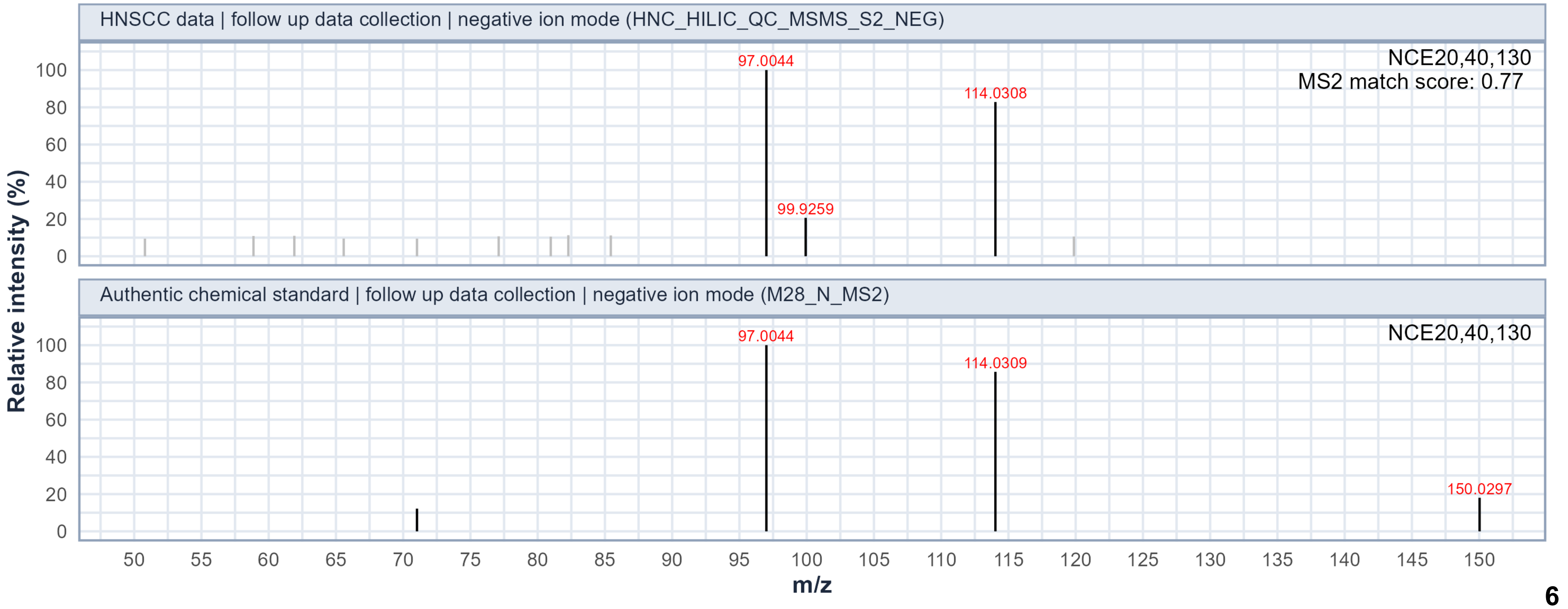

# Betaine [M-e]+ | HMDB0000043

Positive ion mode: 118.0863 m/z | Instrument: QE focus

## Chromatogram

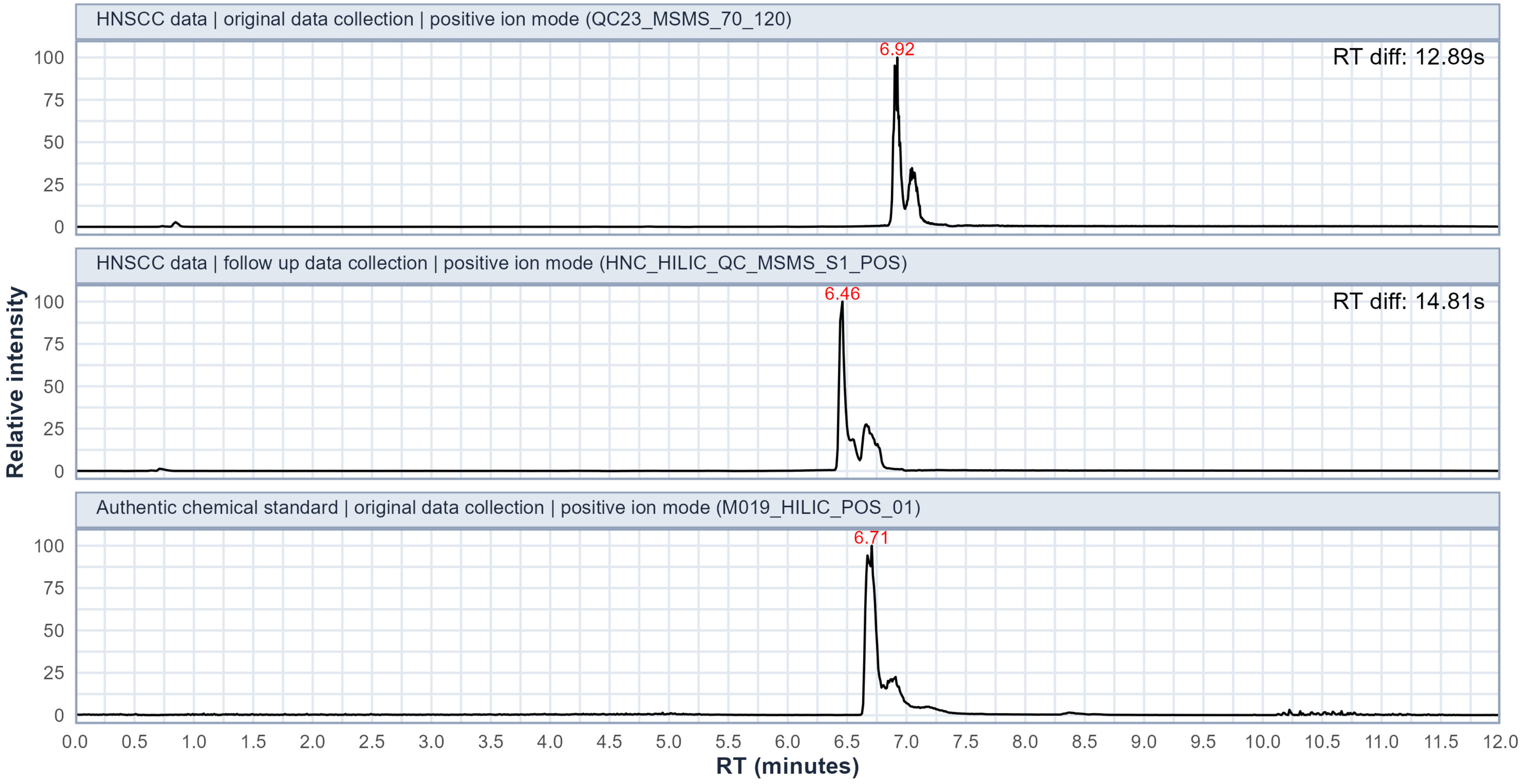


## MS/MS

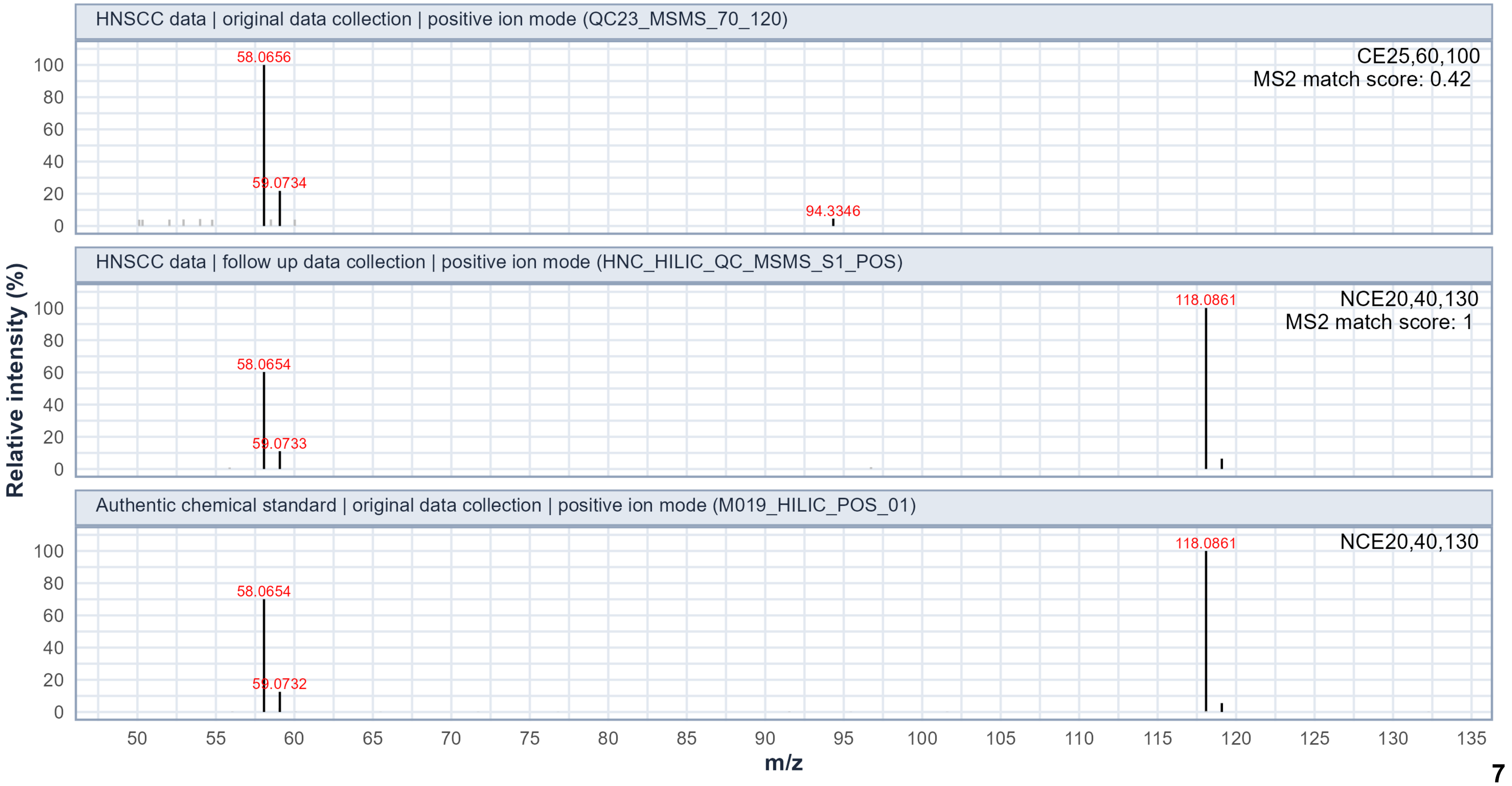

# Carnitine [M+H]+ | HMDB0000062

Positive ion mode: 162.1125 m/z | Instrument: QE focus

## Chromatogram

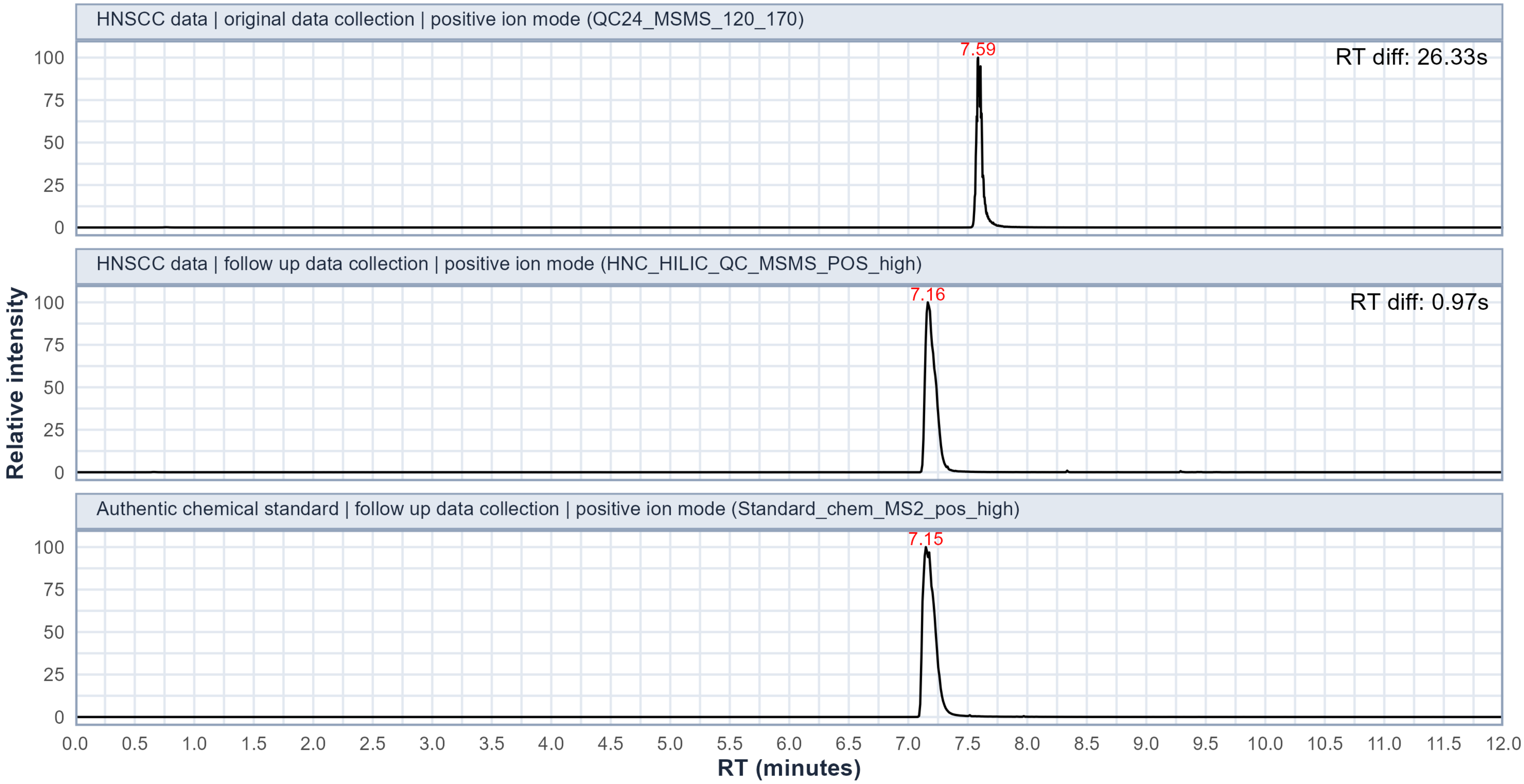


## MS/MS

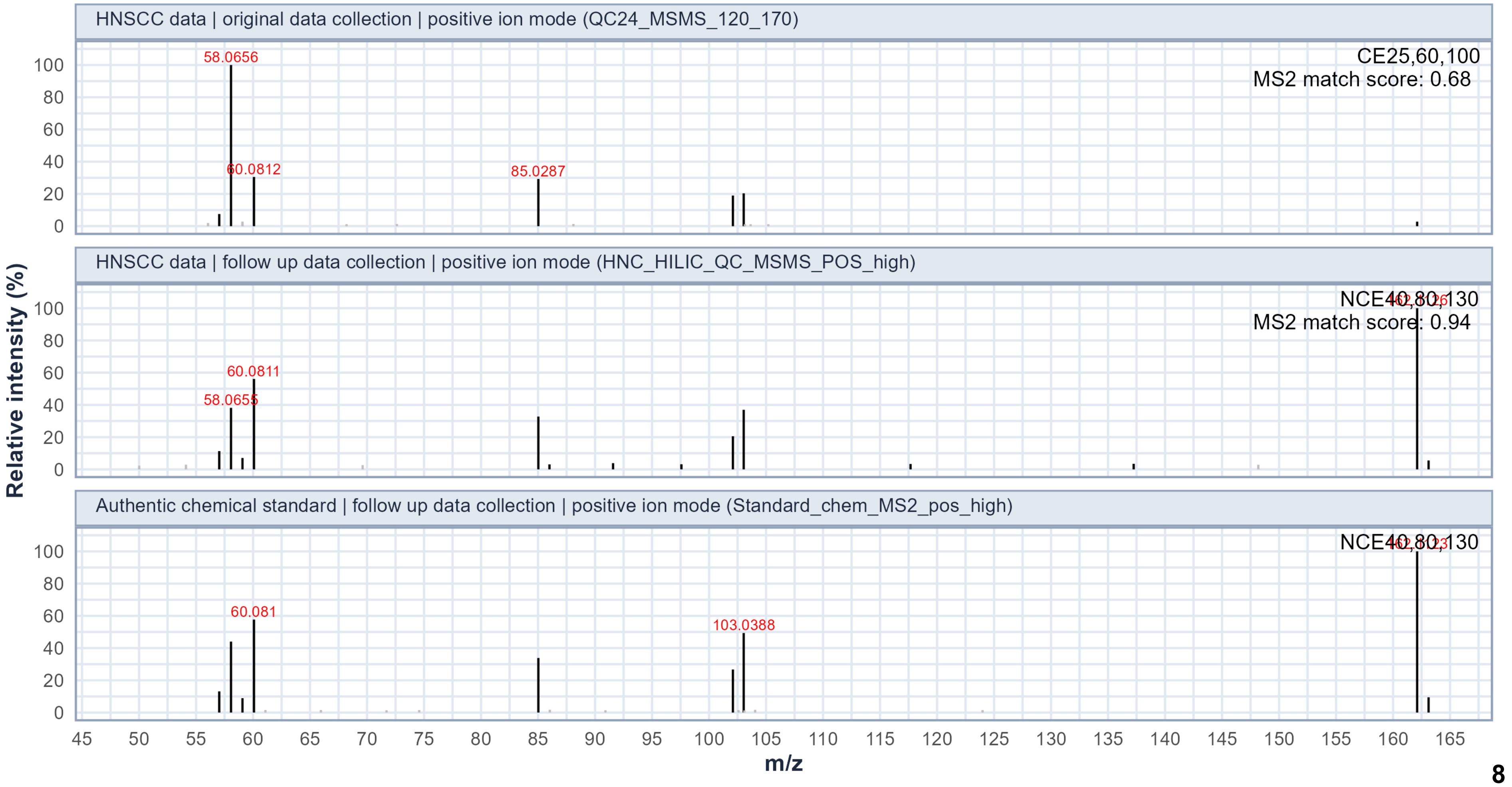

# Carnosine [M+H]+ | HMDB0000033

Positive ion mode: 227.1139 m/z | Instrument: QE focus

## Chromatogram

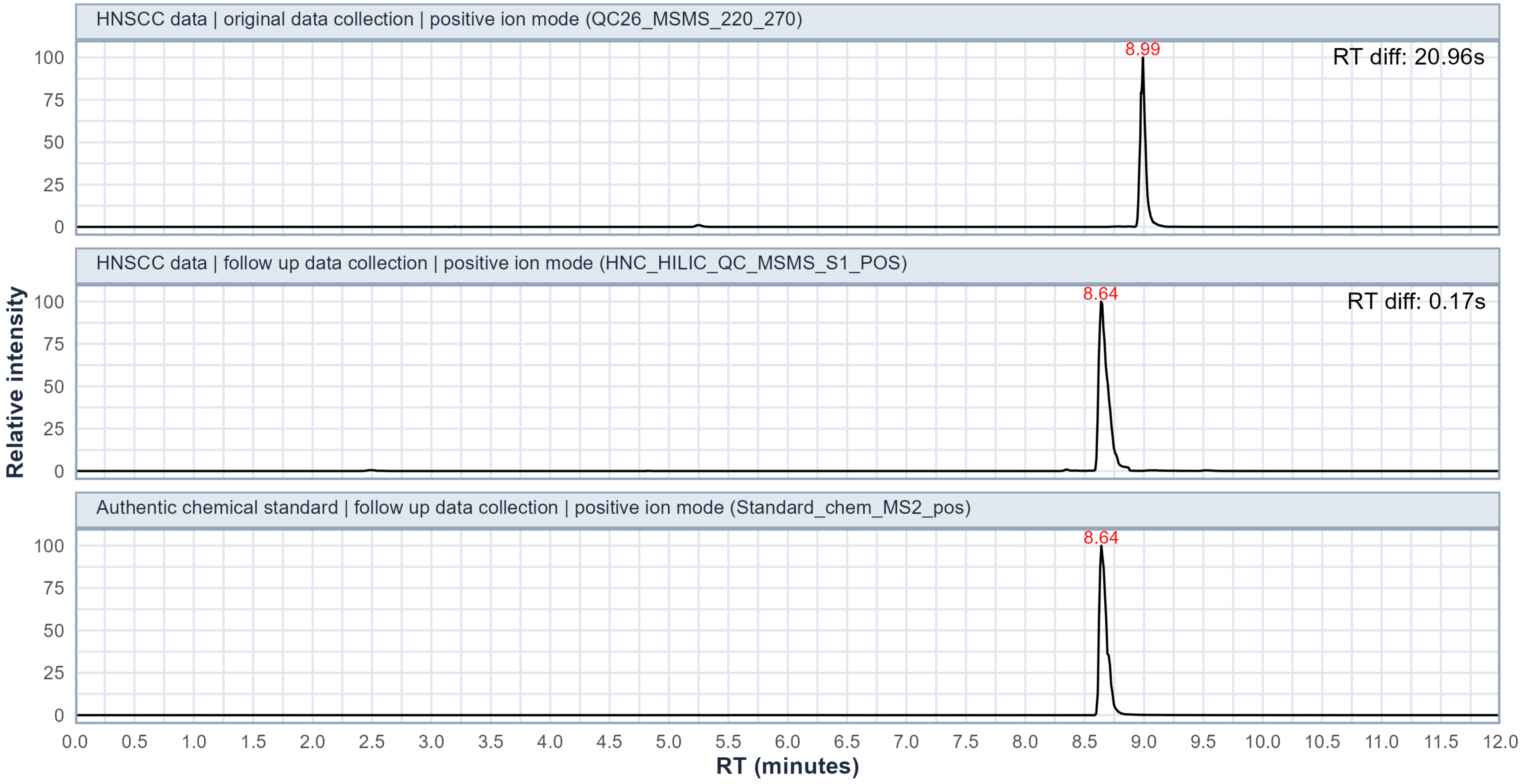


## MS/MS

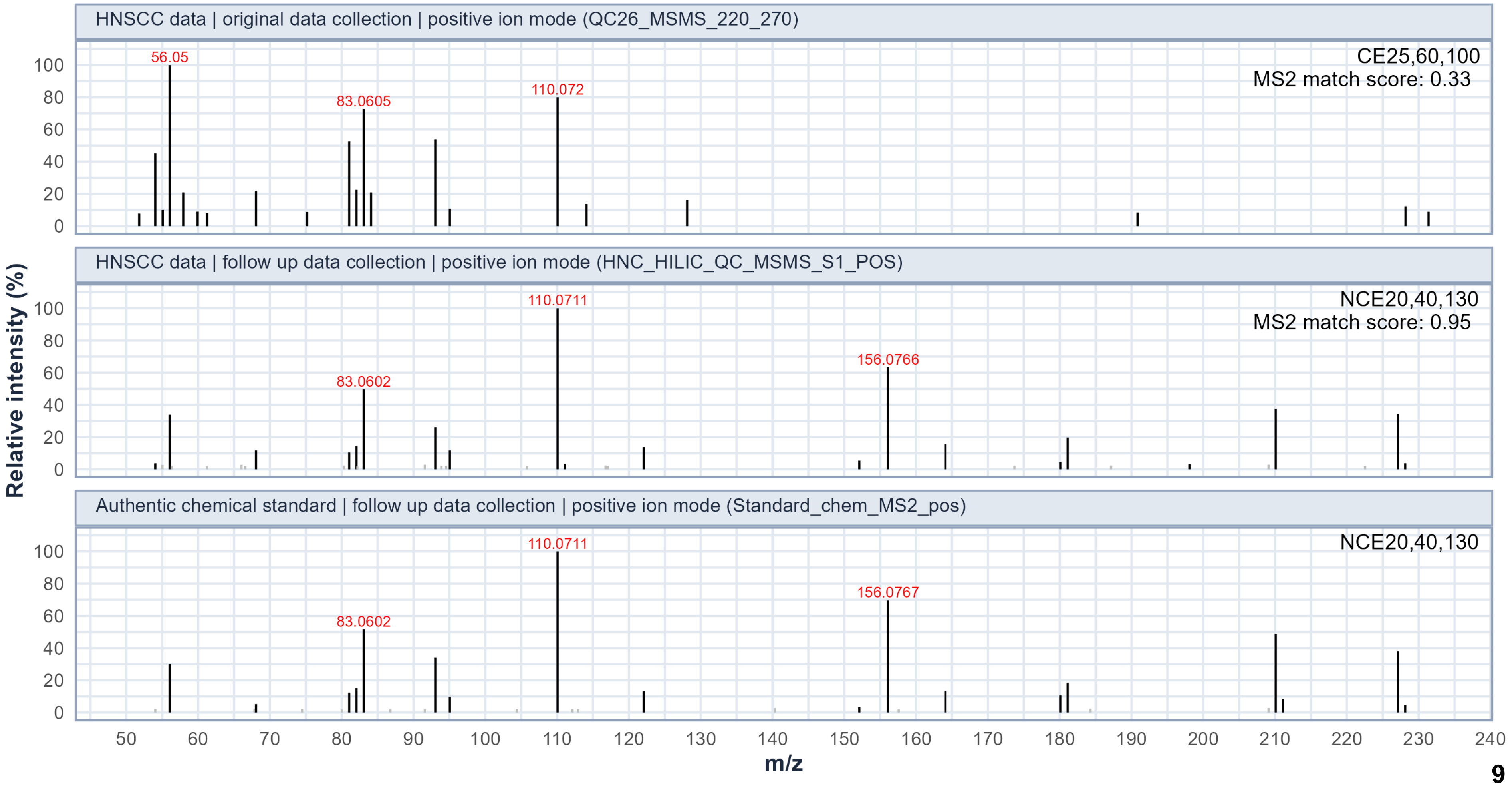

# Cytidine 5'-diphosphocholine [M+H]+ | HMDB0260340

Positive ion mode: 489.1146 m/z | Instrument: QE focus
Authentic chemical standard not available in library; identification based on MS/MS match to mzCloud.

## Chromatogram

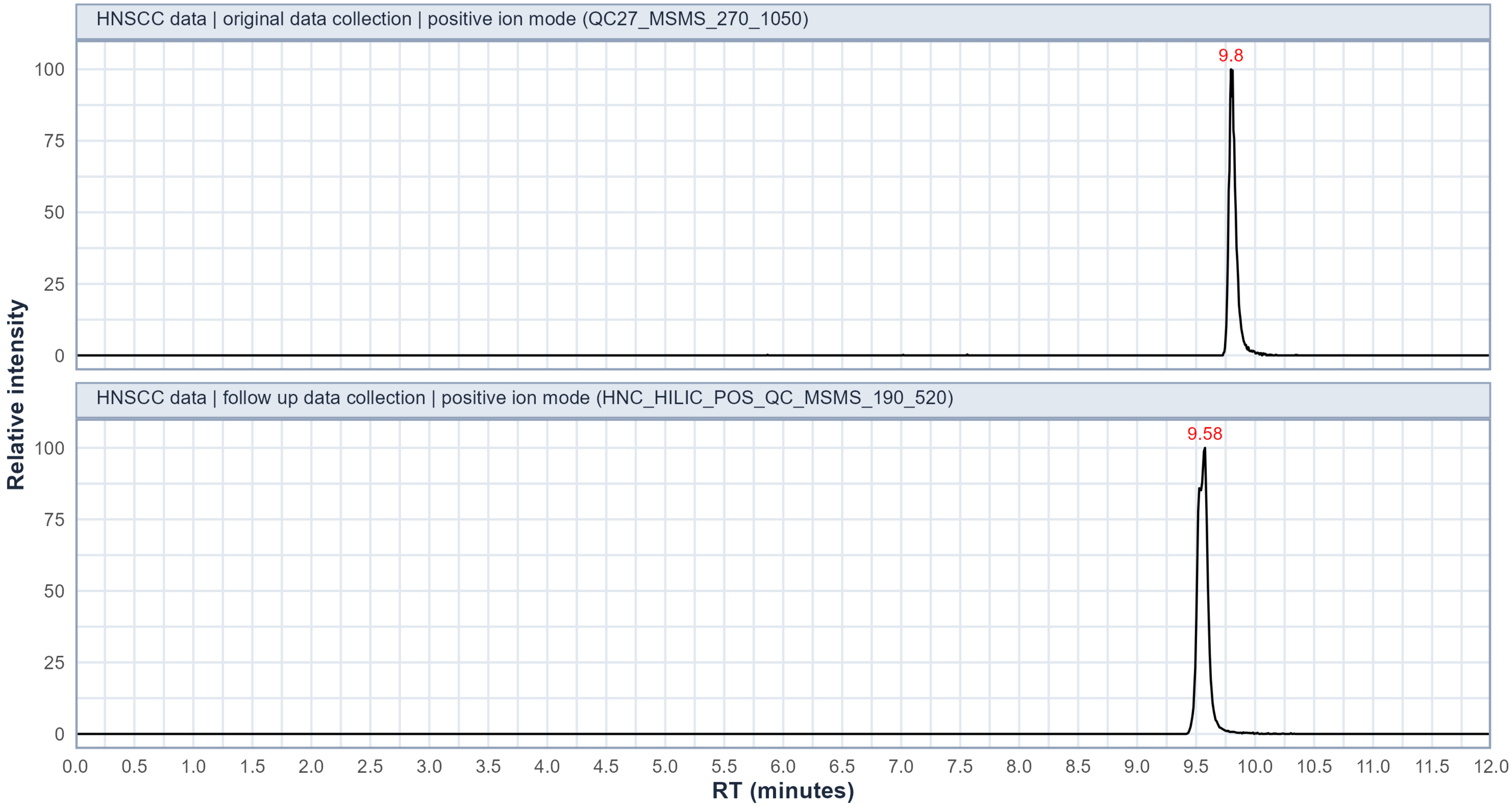


## MS/MS

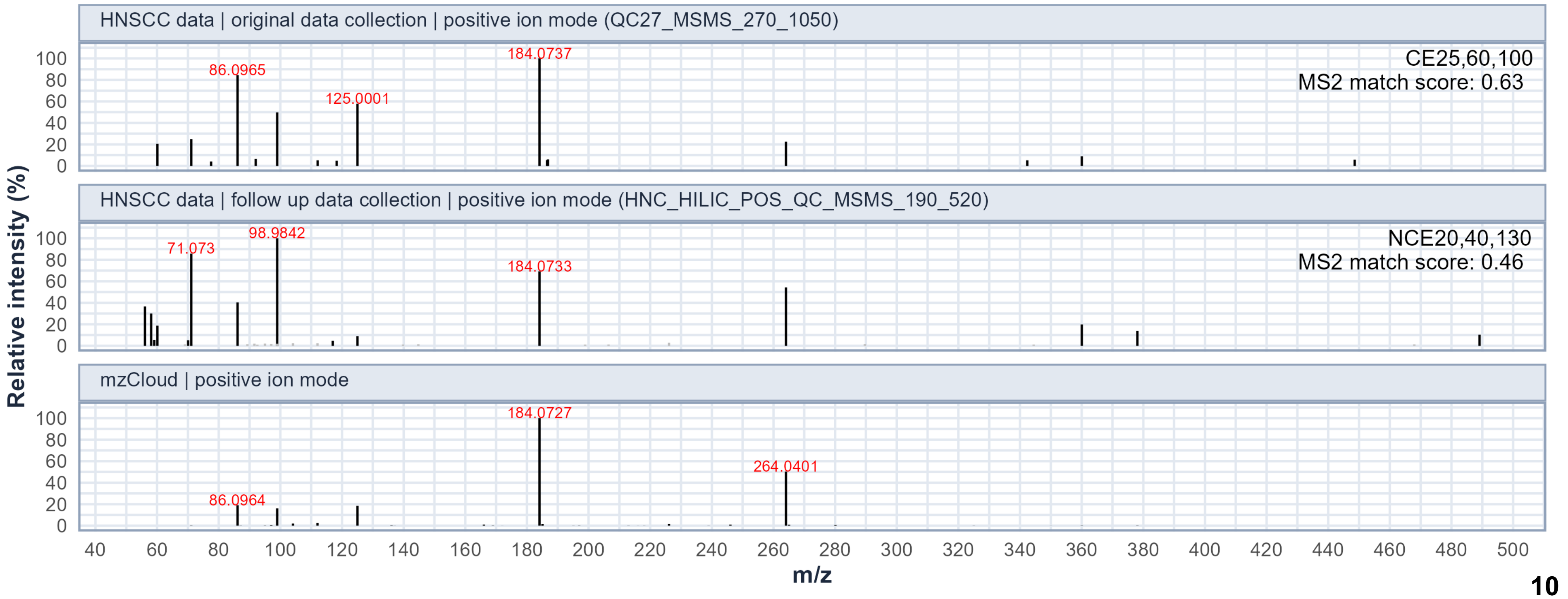

# Choline [M-e]+ | HMDB0000097

Positive ion mode: 104.107 m/z | Instrument: QE focus

## Chromatogram

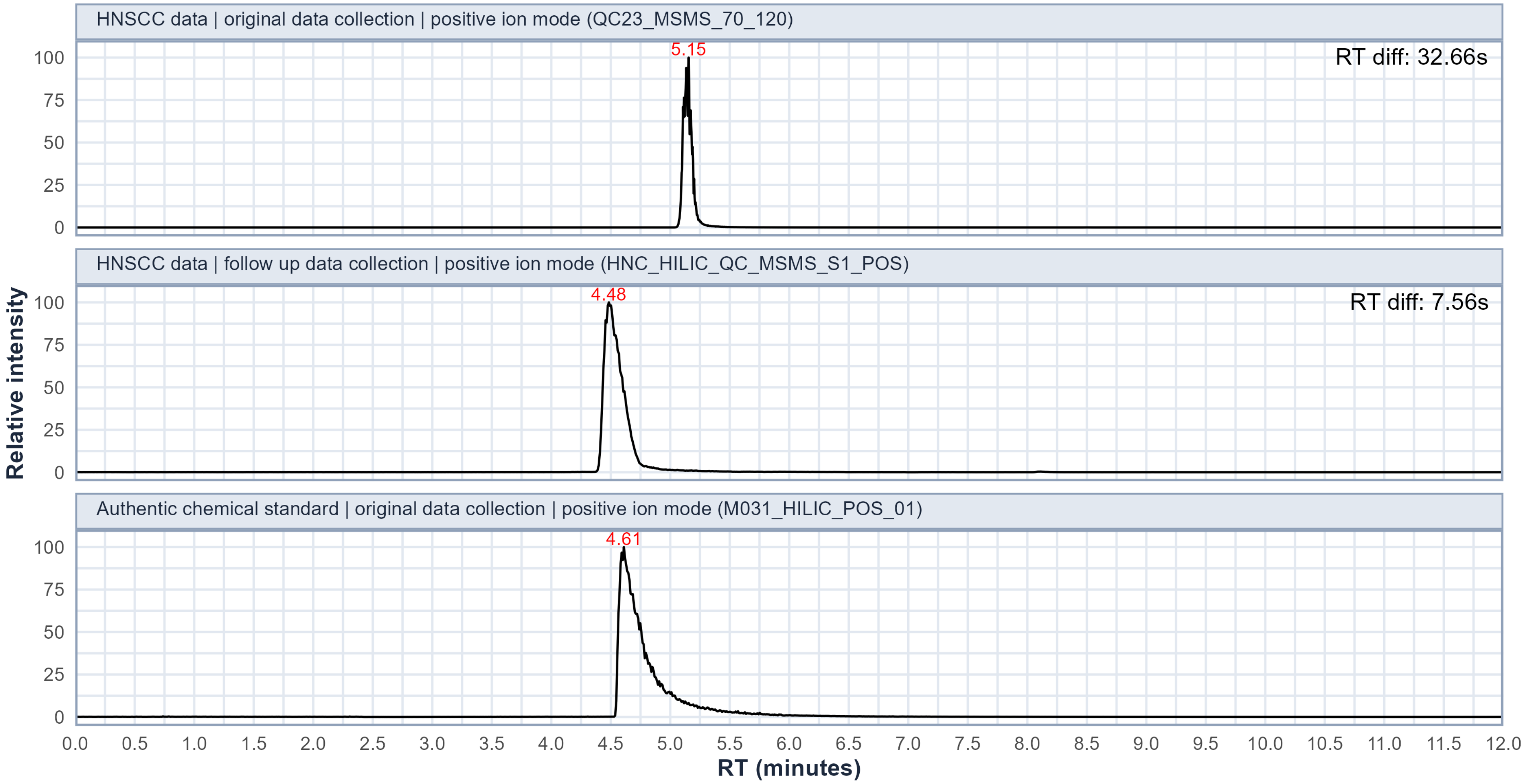


## MS/MS

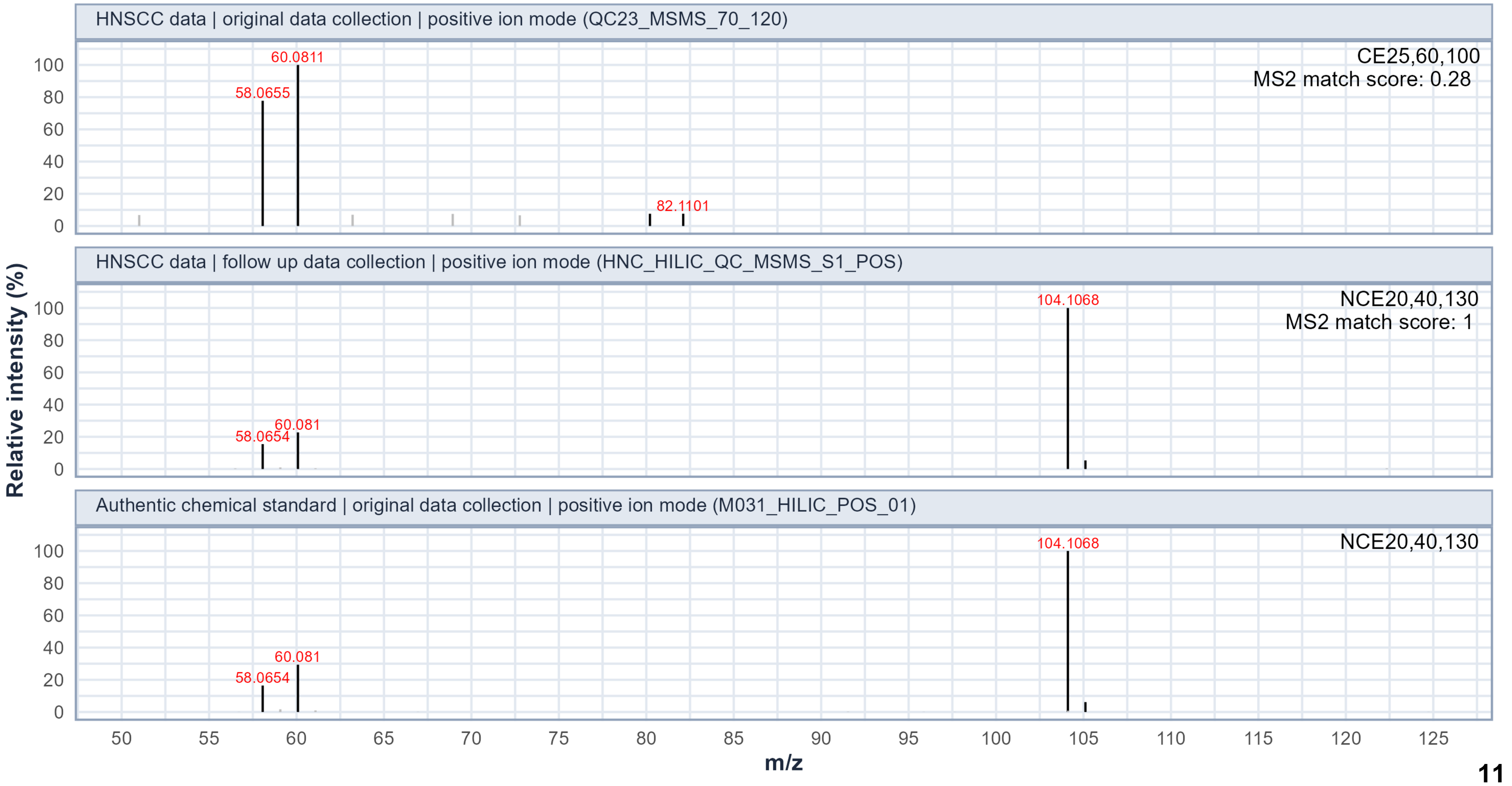

# Deoxyadenosine [M+H]+ | HMDB0000101

Positive ion mode: 252.1091 m/z | Instrument: QE focus

## Chromatogram

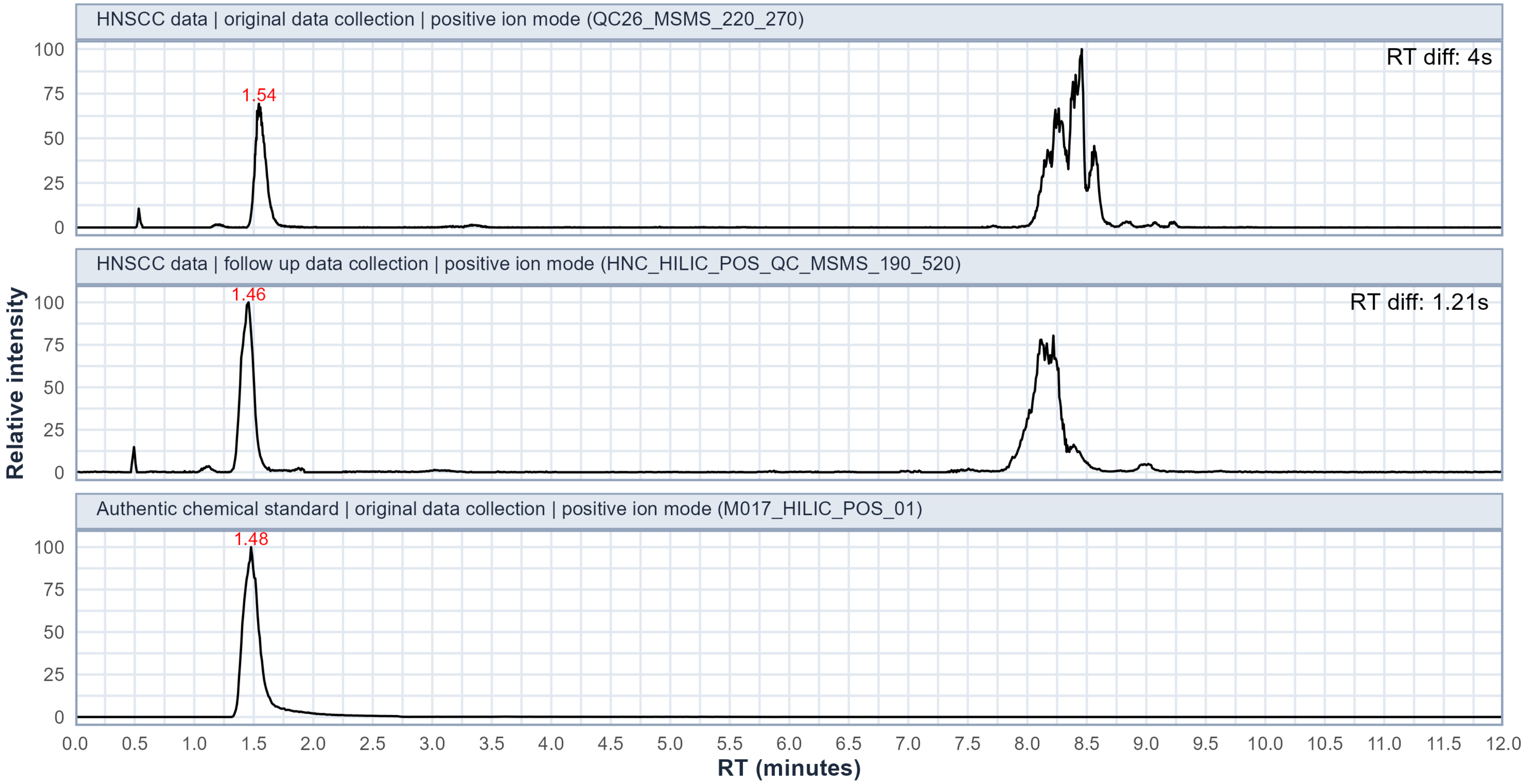


## MS/MS

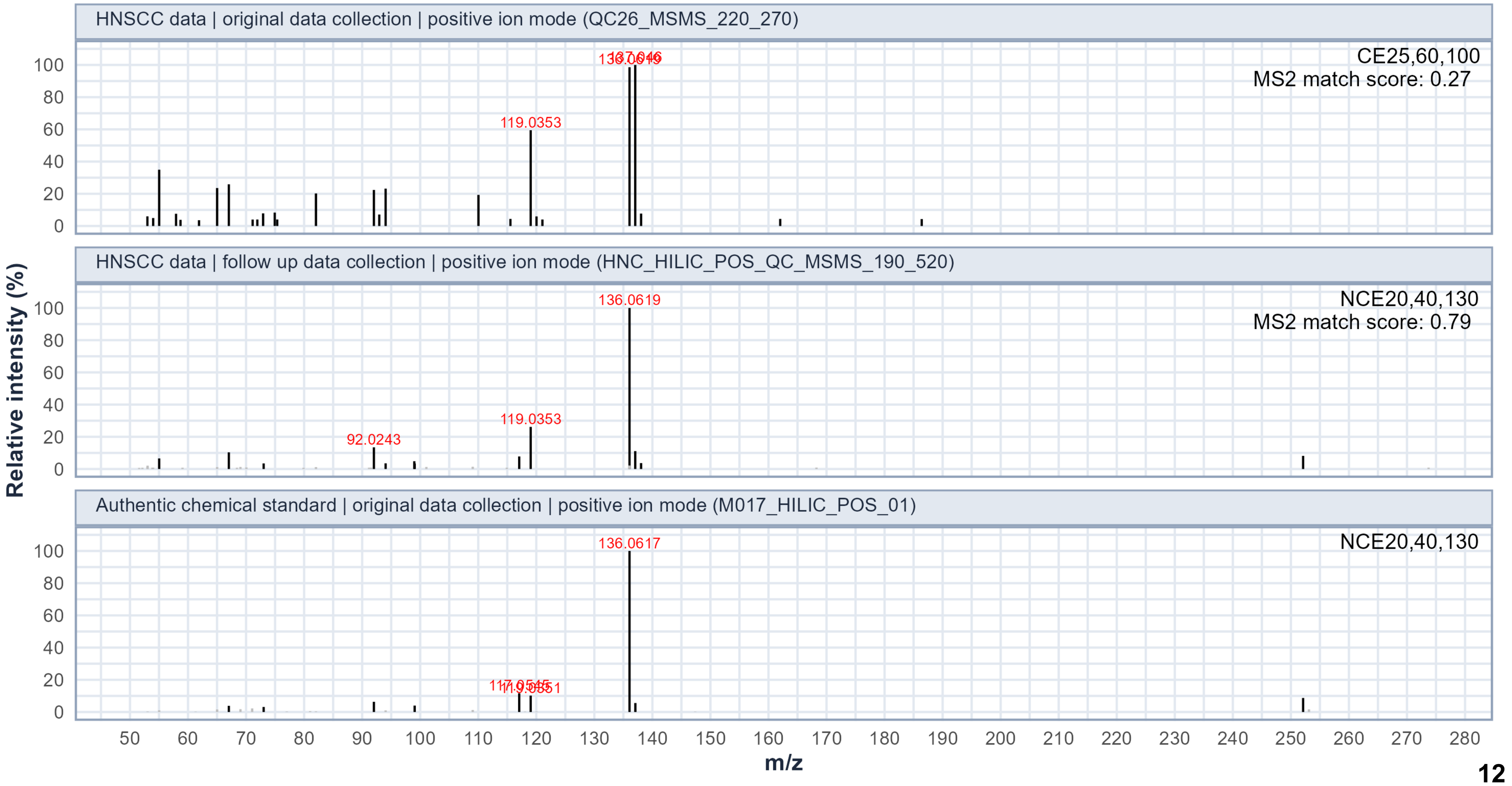

# Deoxyguanosine [M-H]- | HMDB0000085

Negative ion mode: 266.0895 m/z | Instrument: QE focus

## Chromatogram

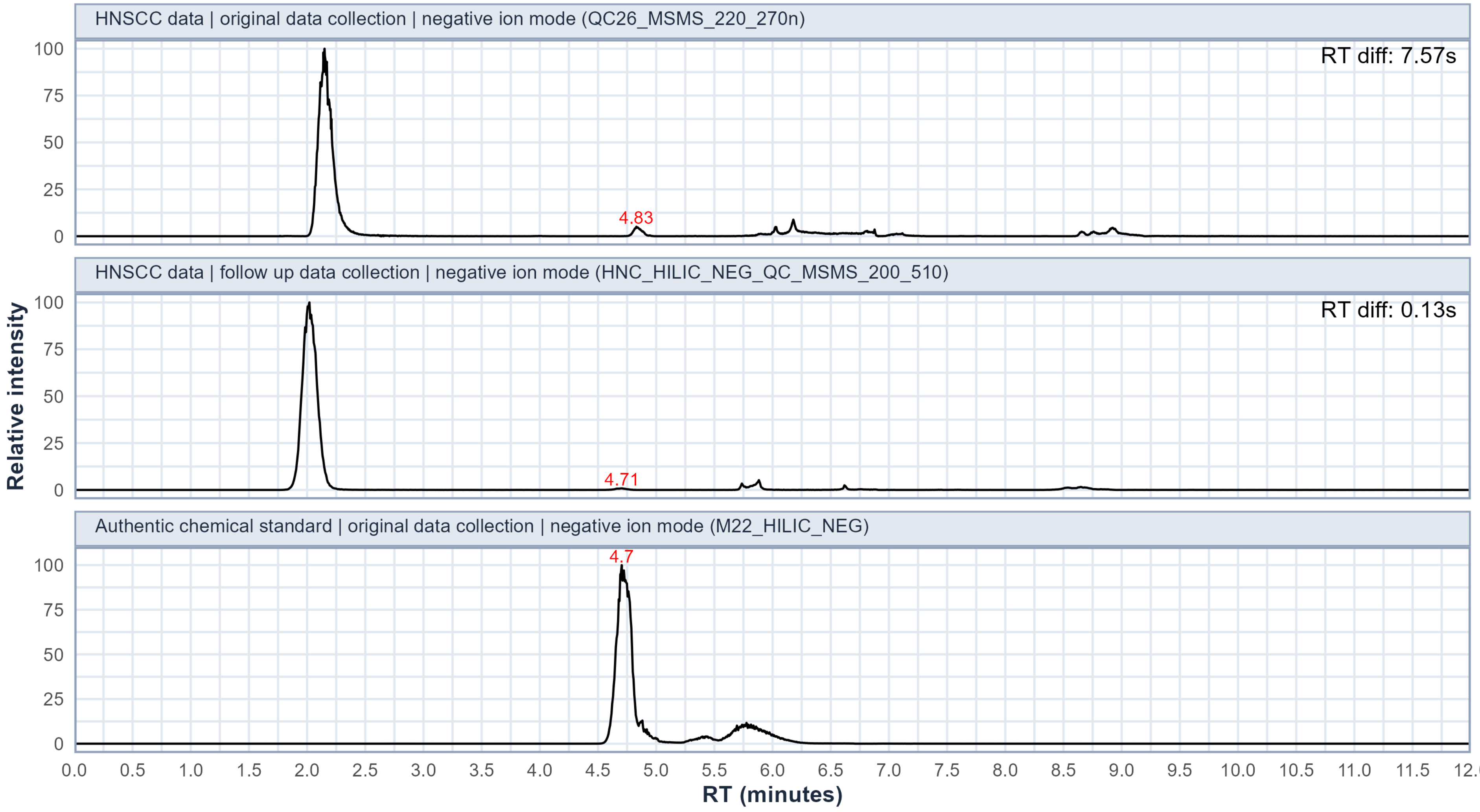


## MS/MS

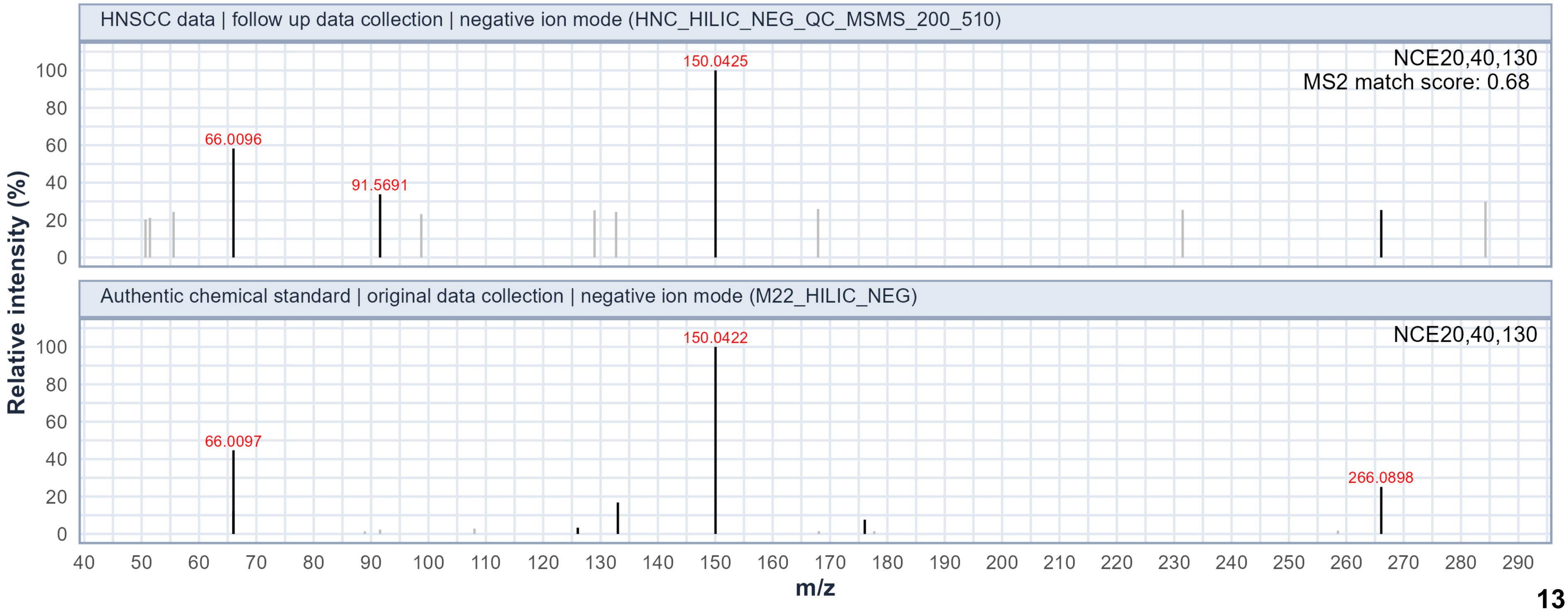

# Deoxyinosine [M-H]- | HMDB0000071

Negative ion mode: 251.0786 m/z | Instrument: QE focus

## Chromatogram

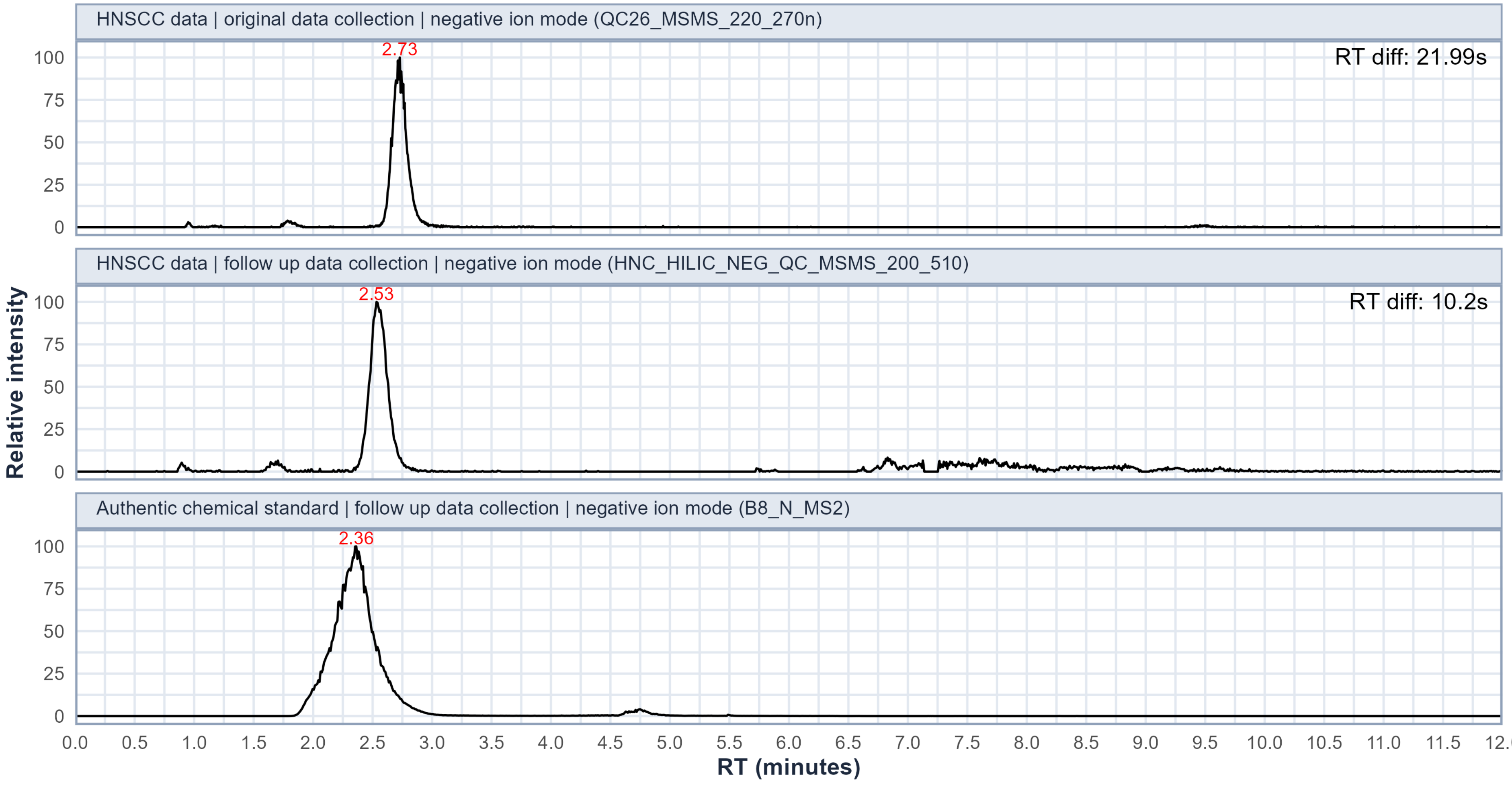


## MS/MS

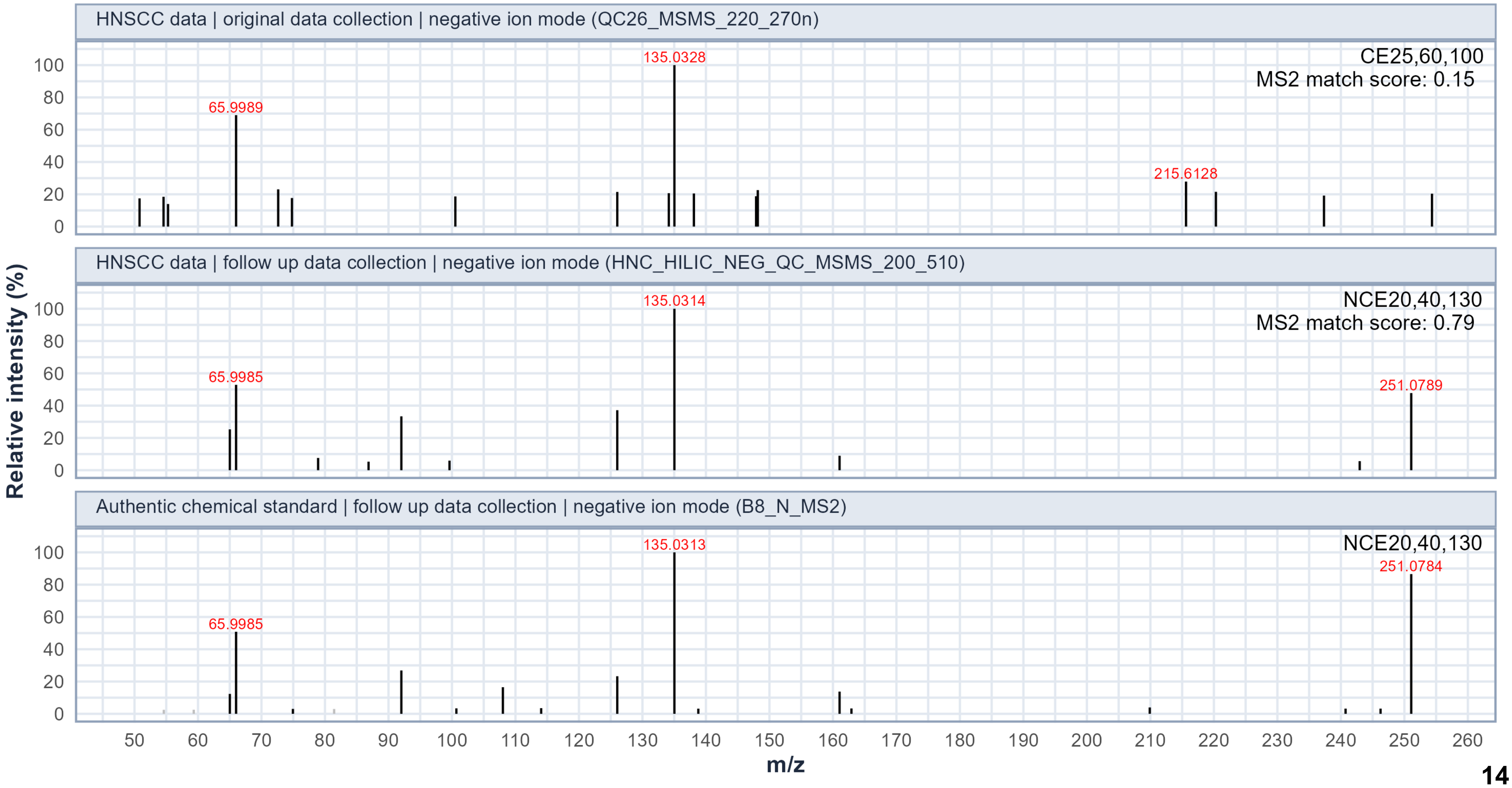

# Dimethylarginine, Asymmetric [M+H]+ | HMDB0001539

Positive ion mode: 203.1502 m/z | Instrument: QE focus

## Chromatogram

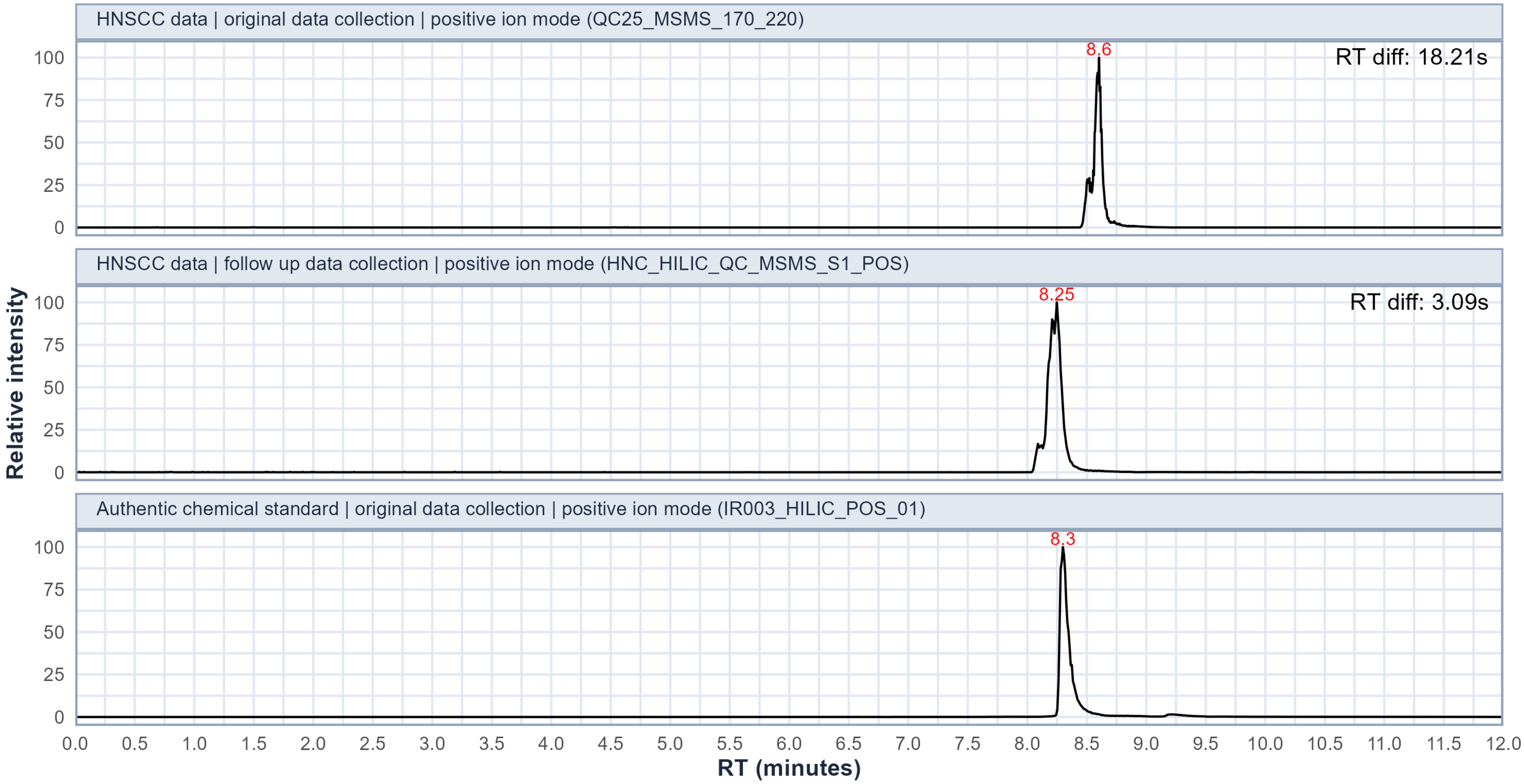


## MS/MS

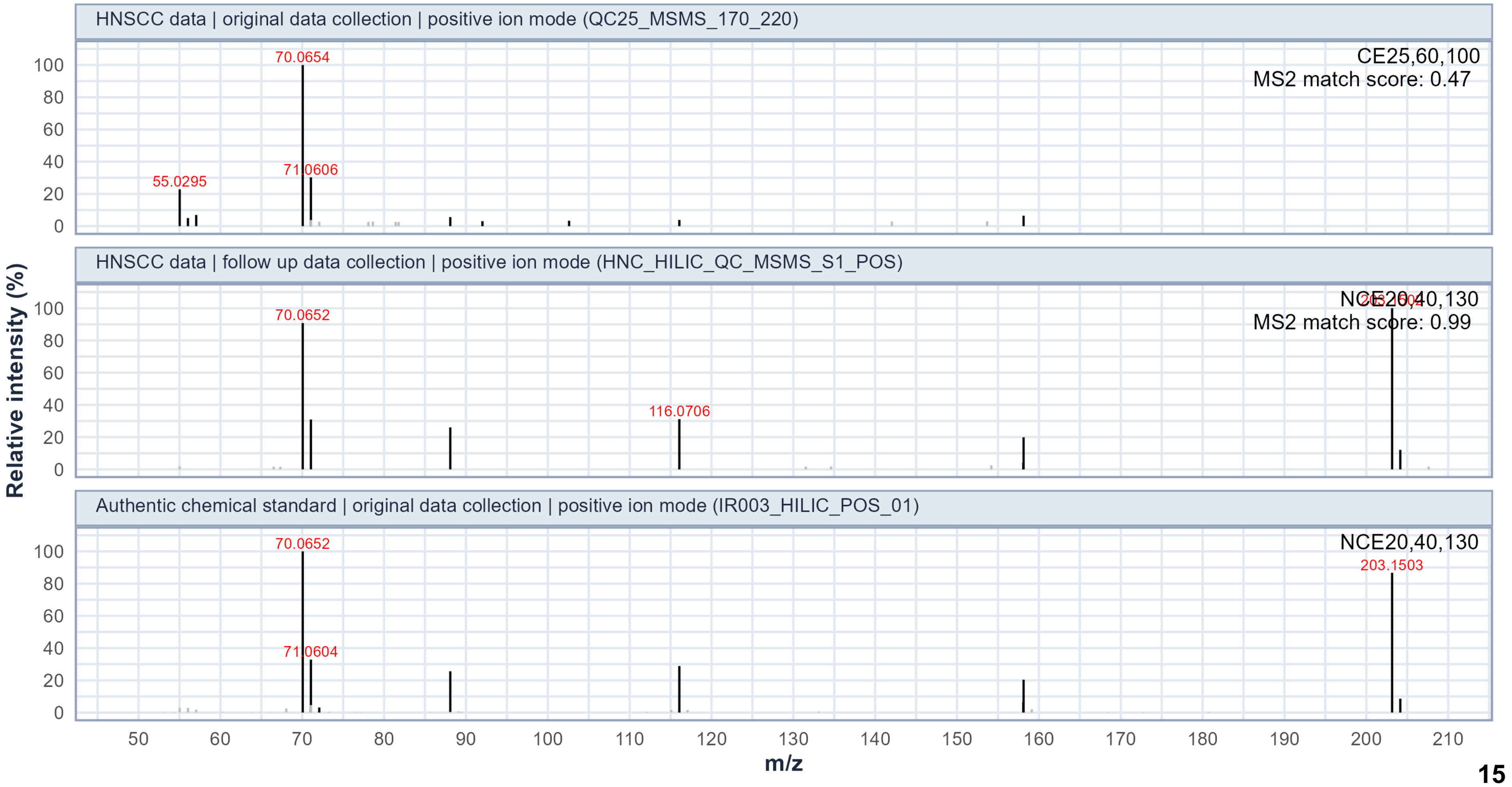

# Dimethylarginine, Symmetric [M+H]+ | HMDB0003334

Positive ion mode: 203.1502 m/z | Instrument: QE focus

## Chromatogram

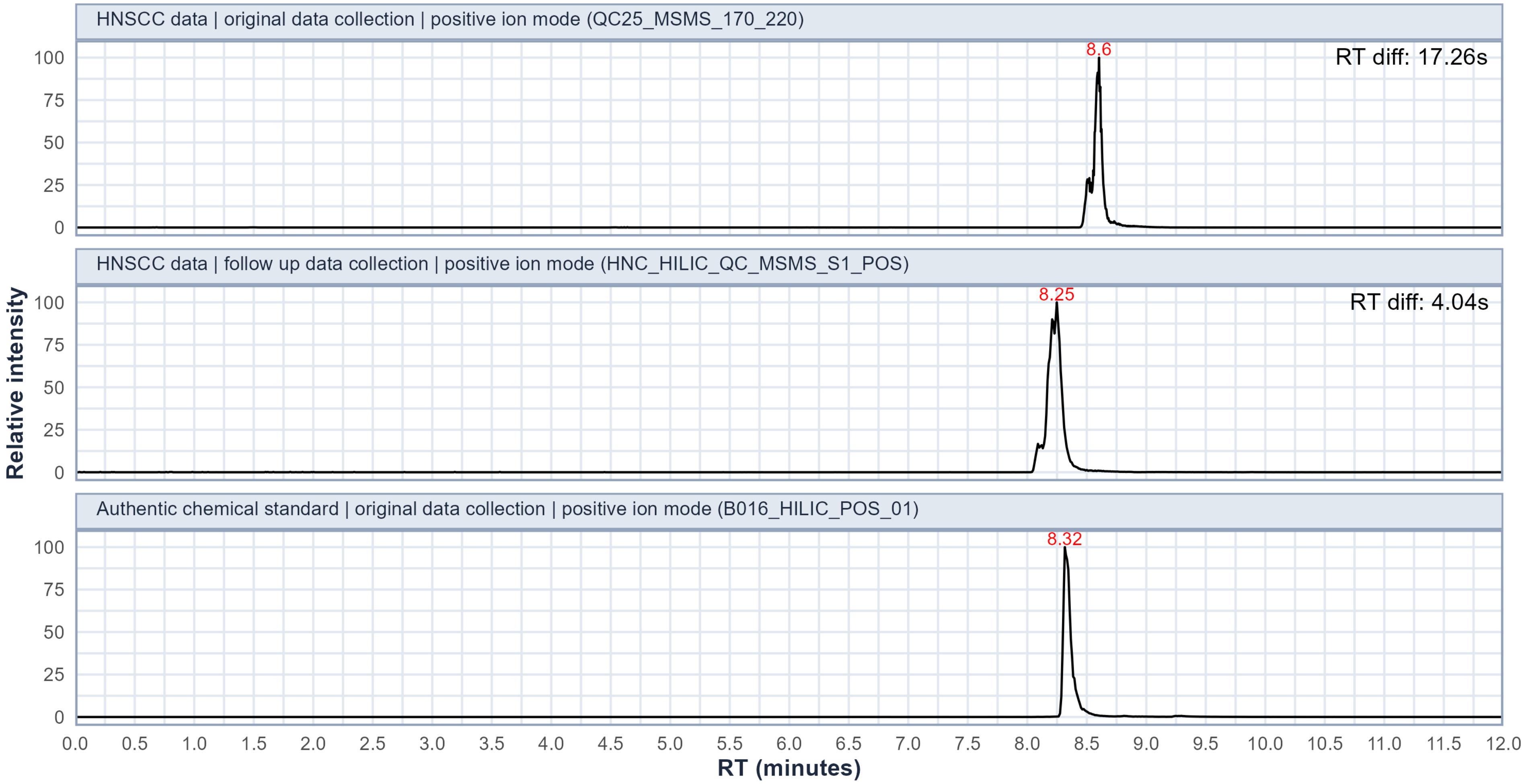


## MS/MS

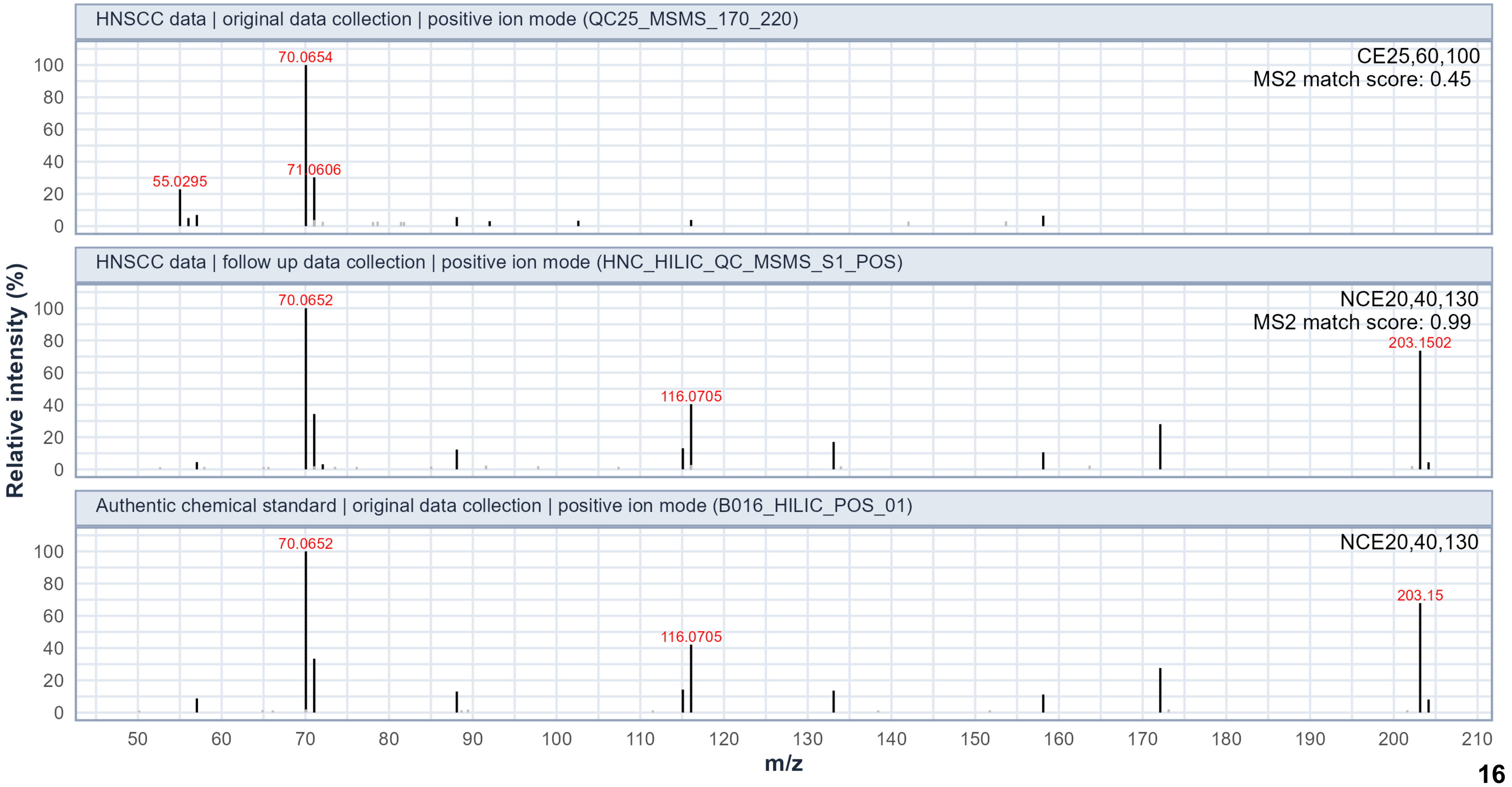

# Dimethylglycine [M+H]+ | HMDB0000092

Positive ion mode: 104.0706 m/z | Instrument: QE focus
A match score below 0.6 DPC likely indicates interference from a second peak that was co-isolated within the fragmentation window.

## Chromatogram

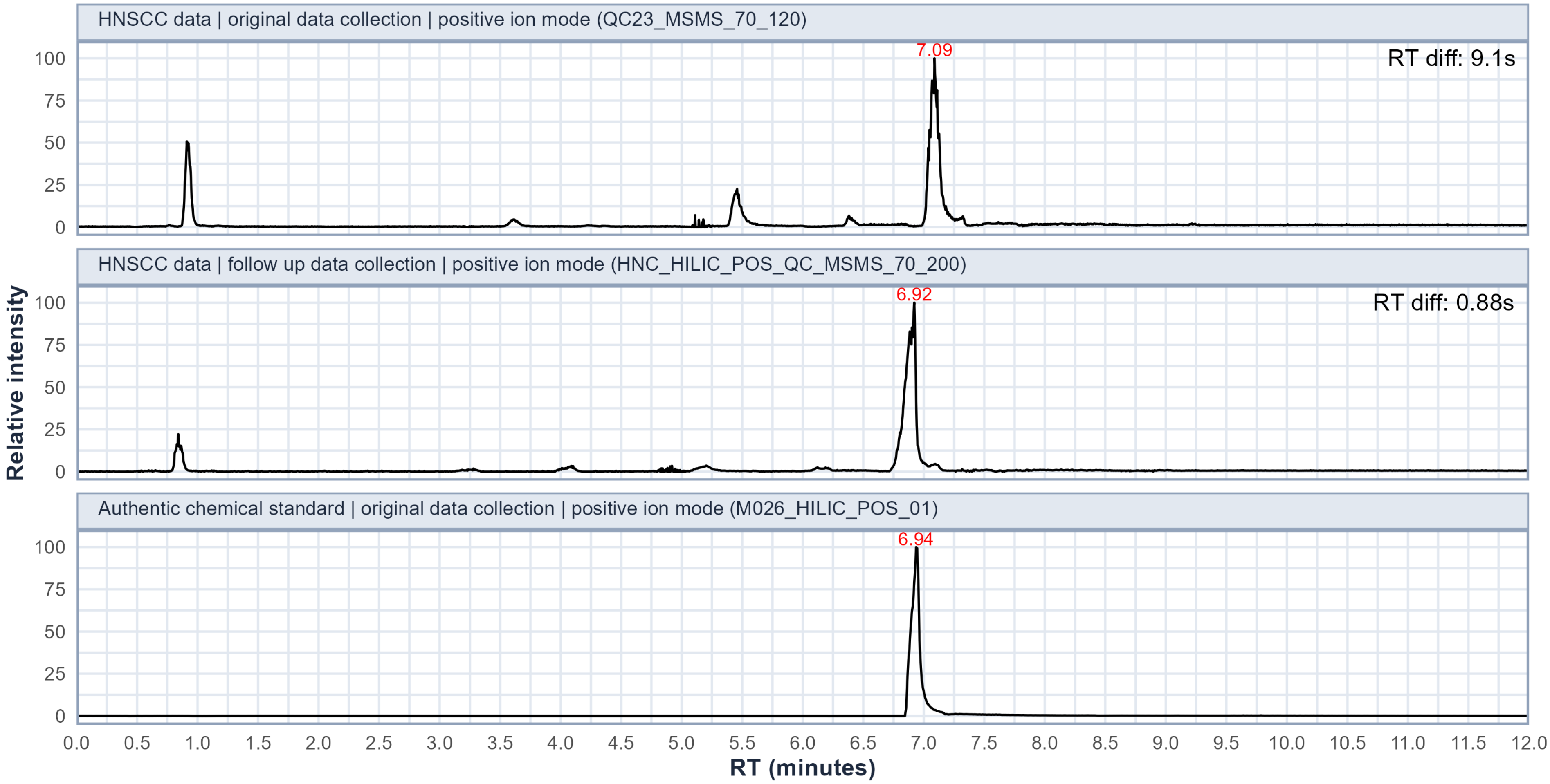


## MS/MS

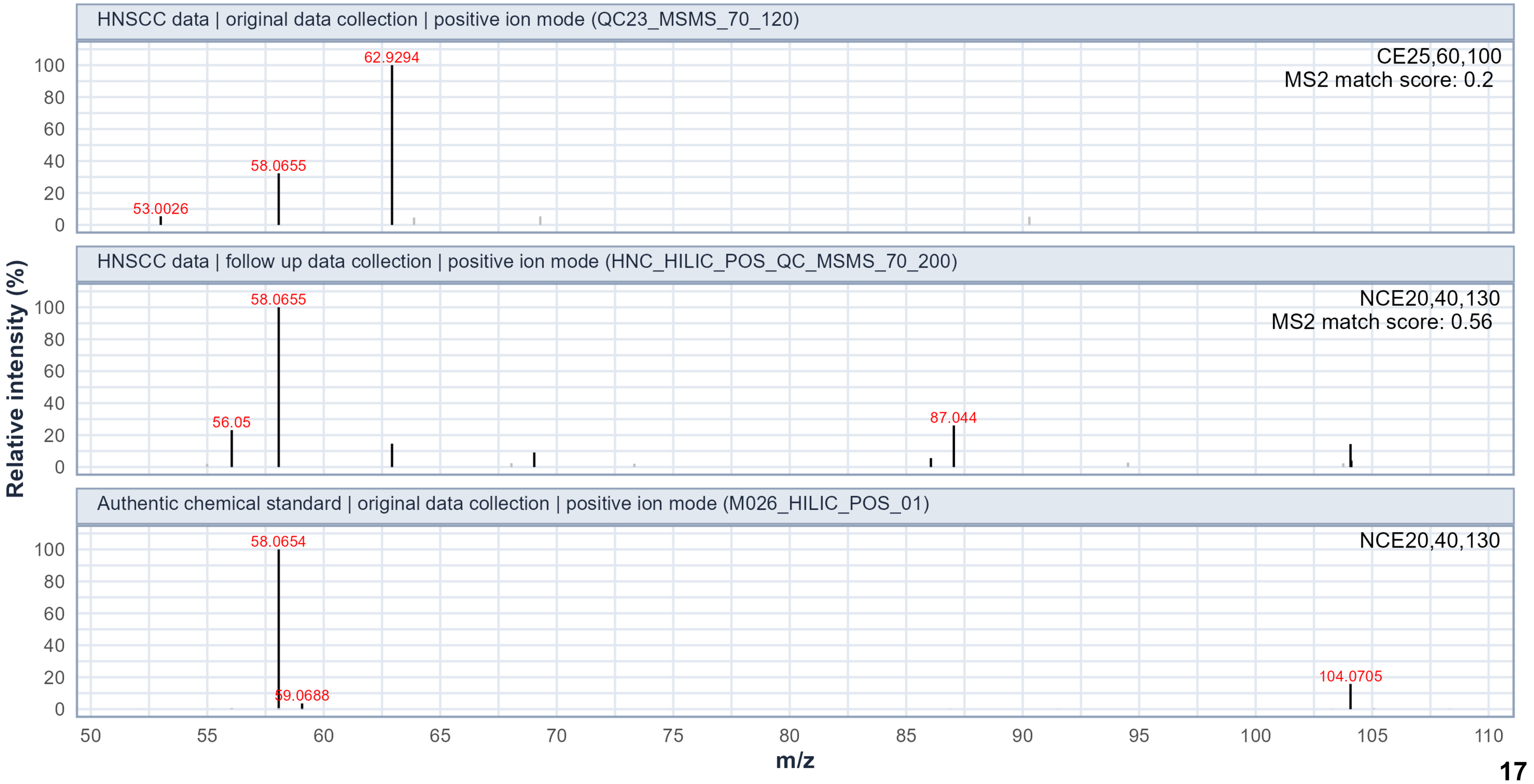

# Folic acid [M-H]- | HMDB0000121

Negative ion mode: 440.1324 m/z | Instrument: QE focus

## Chromatogram

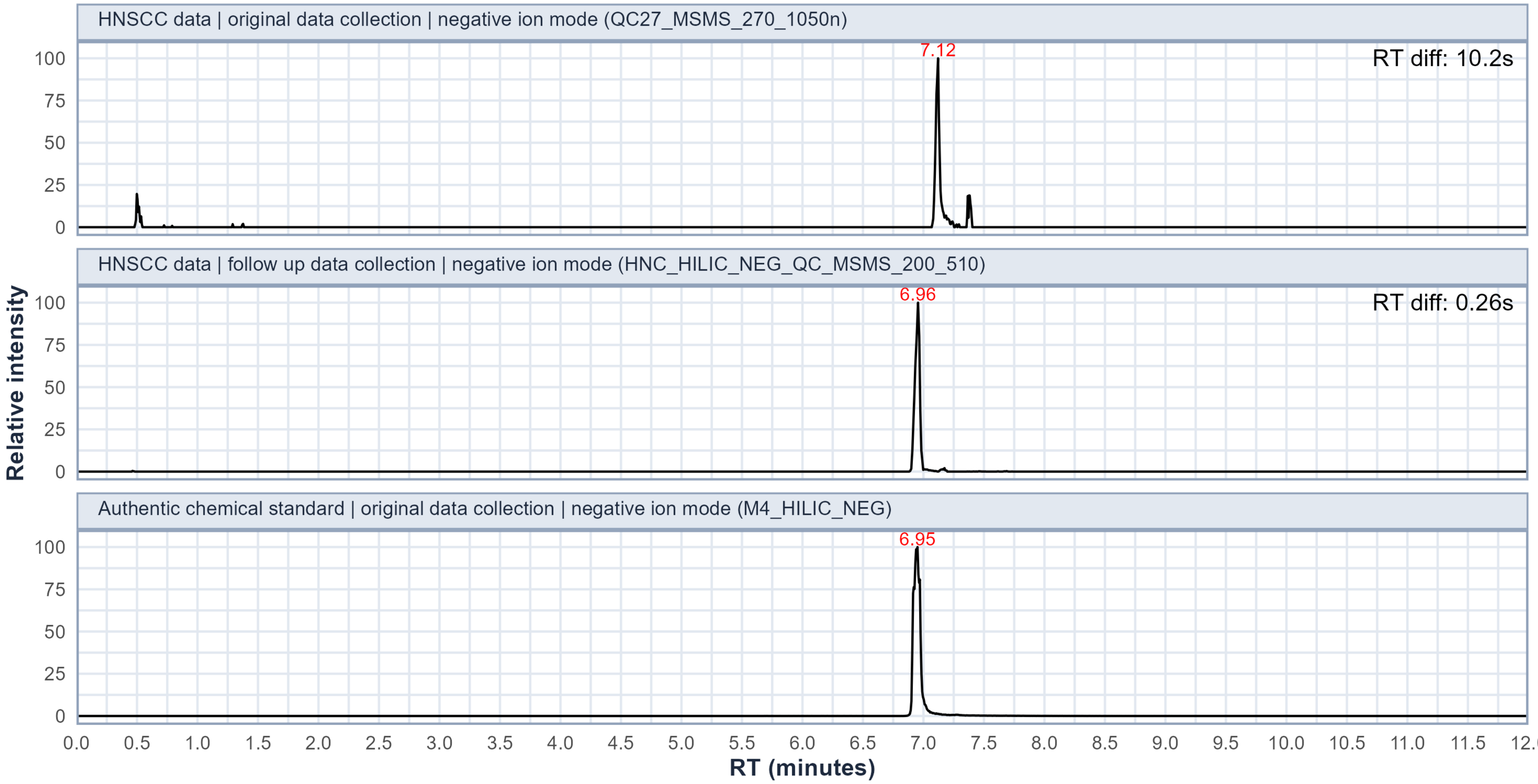


## MS/MS

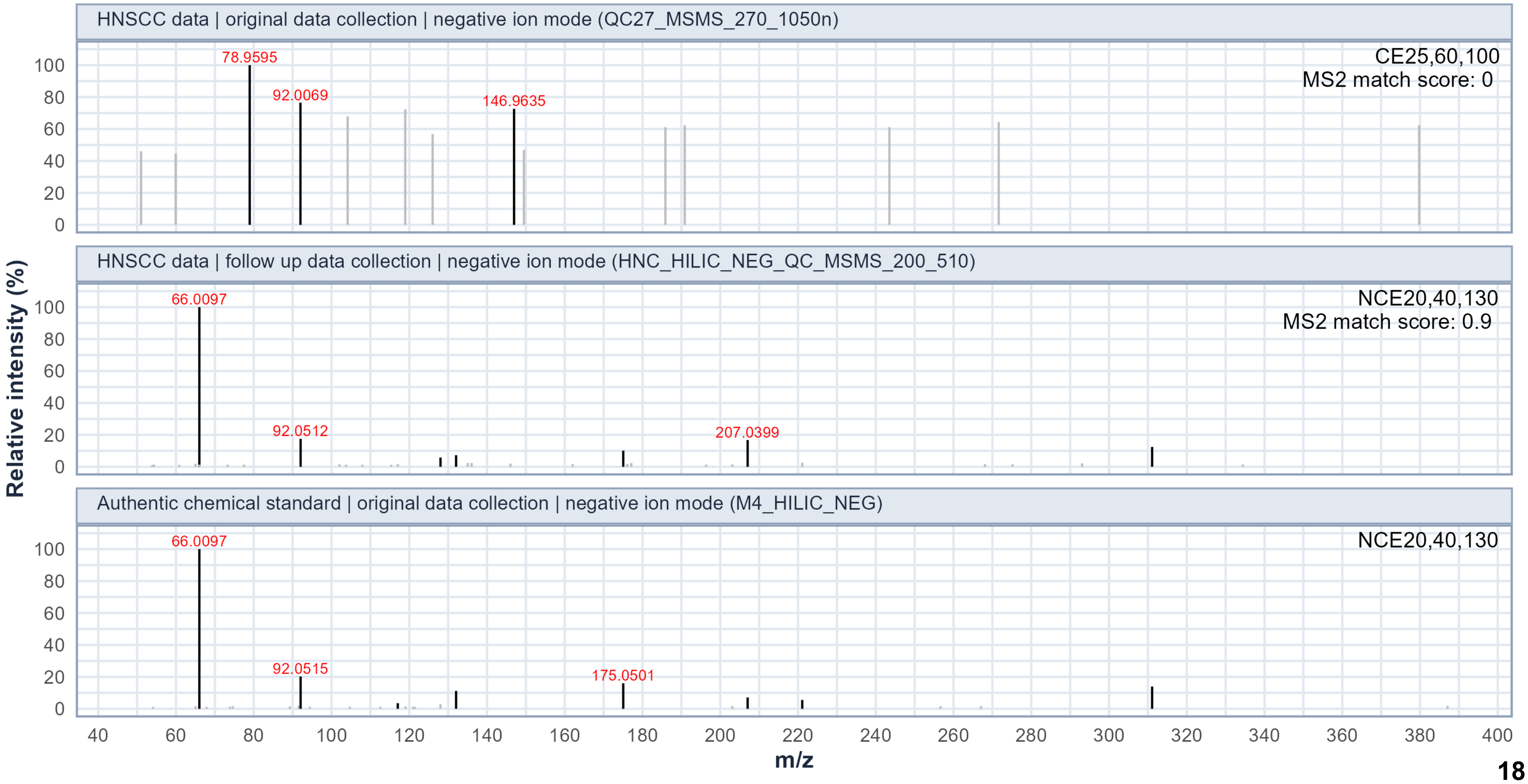

# Glucose [M-H]- | HMDB0000122

Negative ion mode: 179.0561 m/z | Instrument: QE focus

## Chromatogram

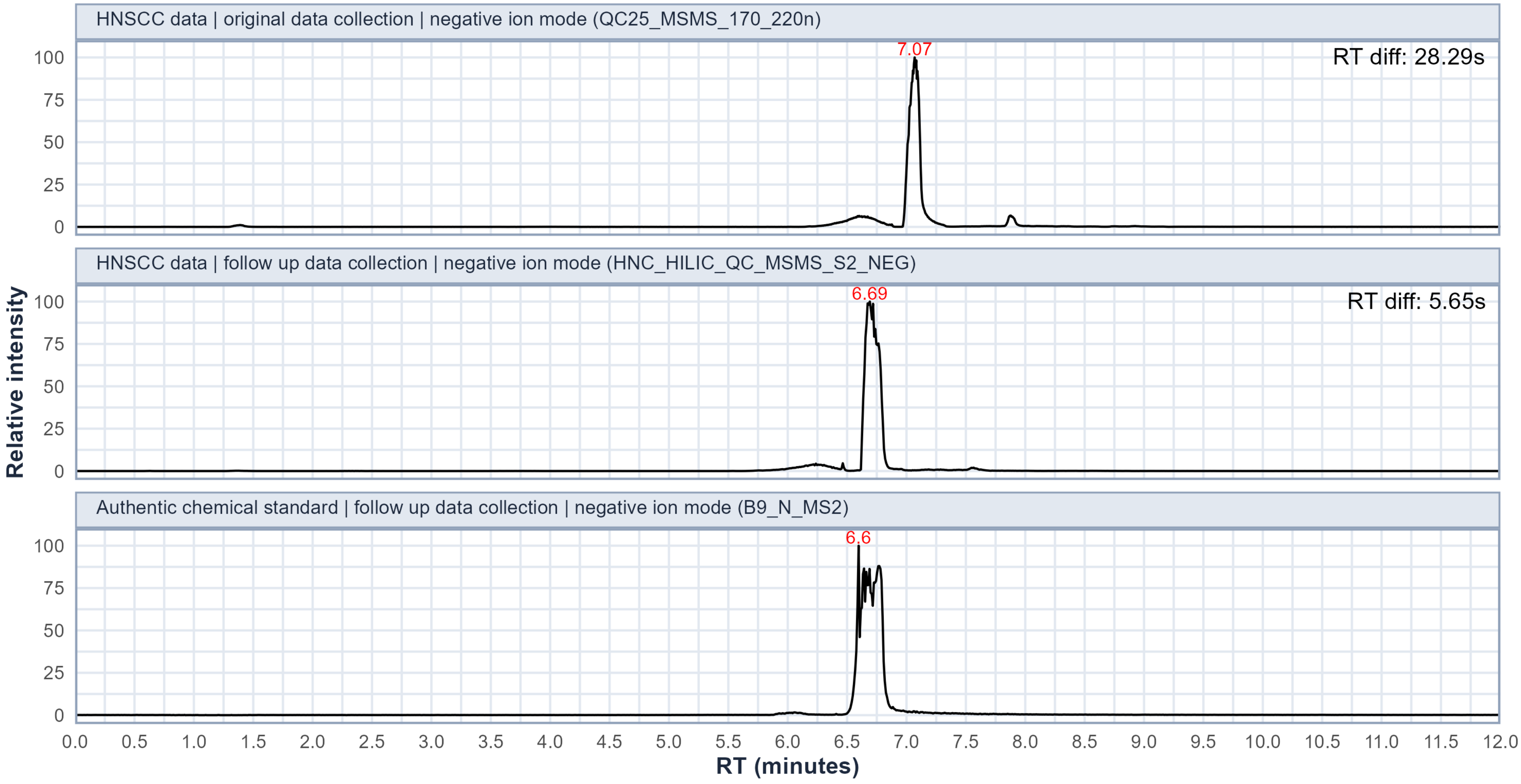


## MS/MS

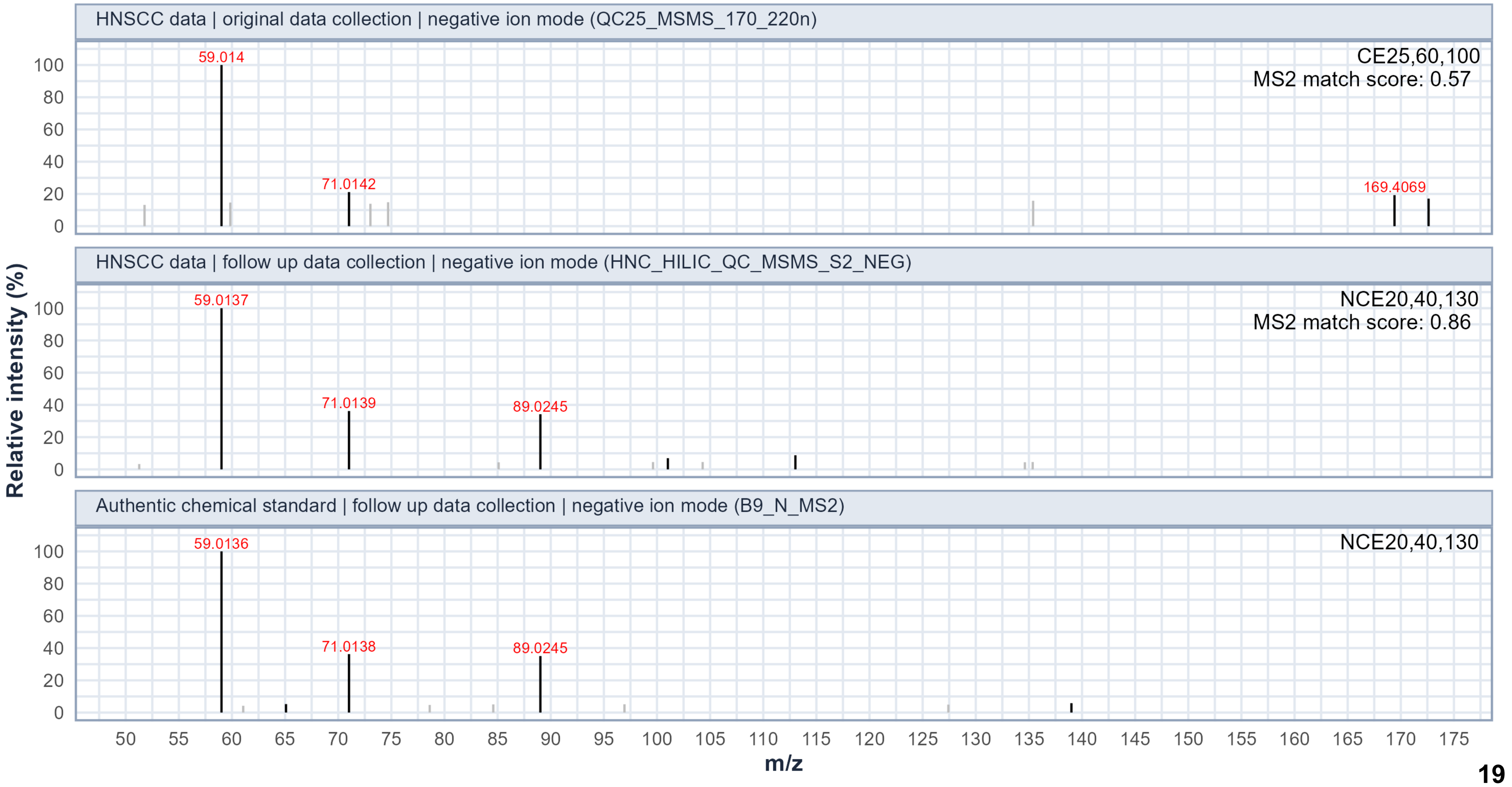

# Guanine [M+H]+ | HMDB0000132

Positive ion mode: 152.0567 m/z | Instrument: QE focus

## Chromatogram

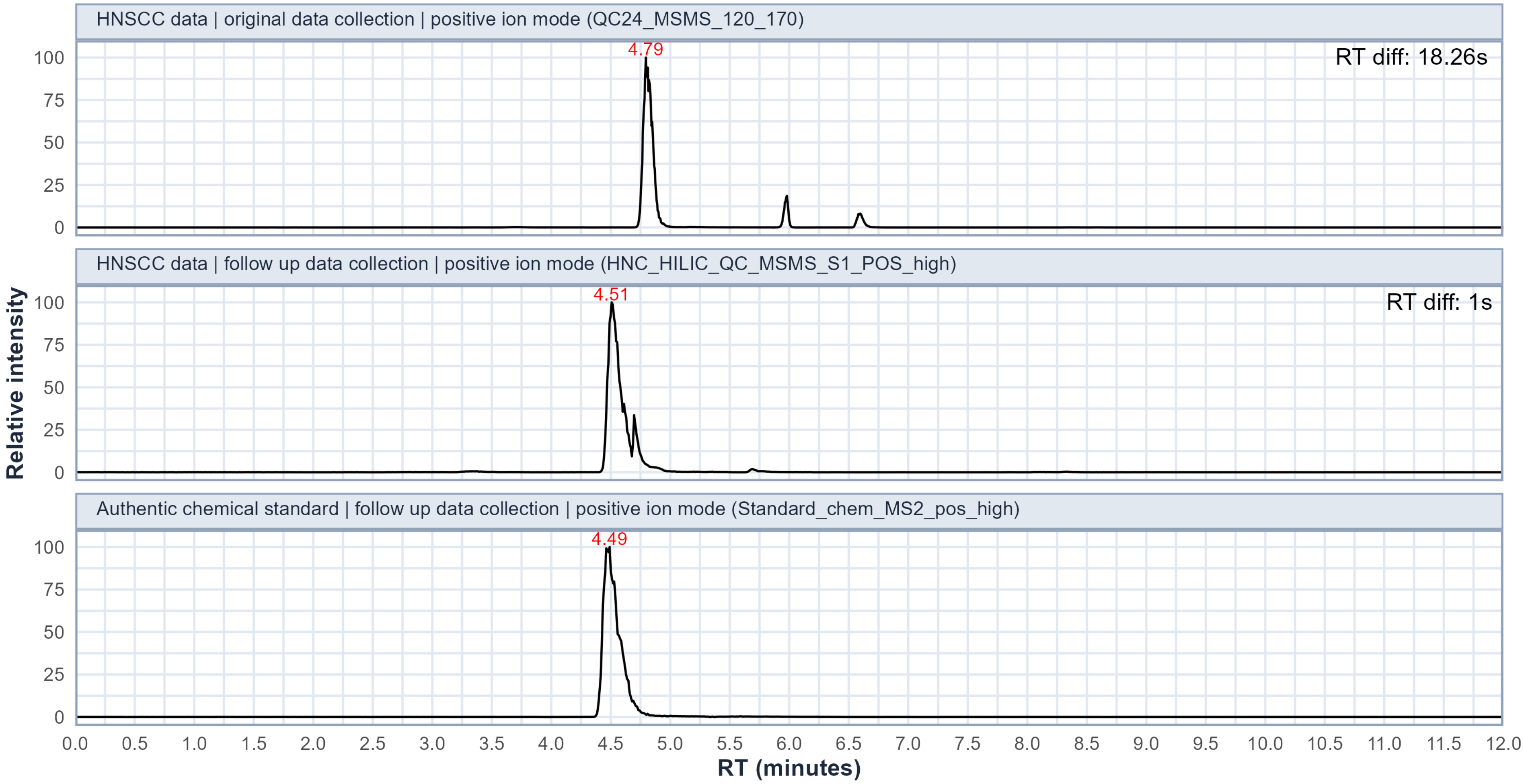


## MS/MS

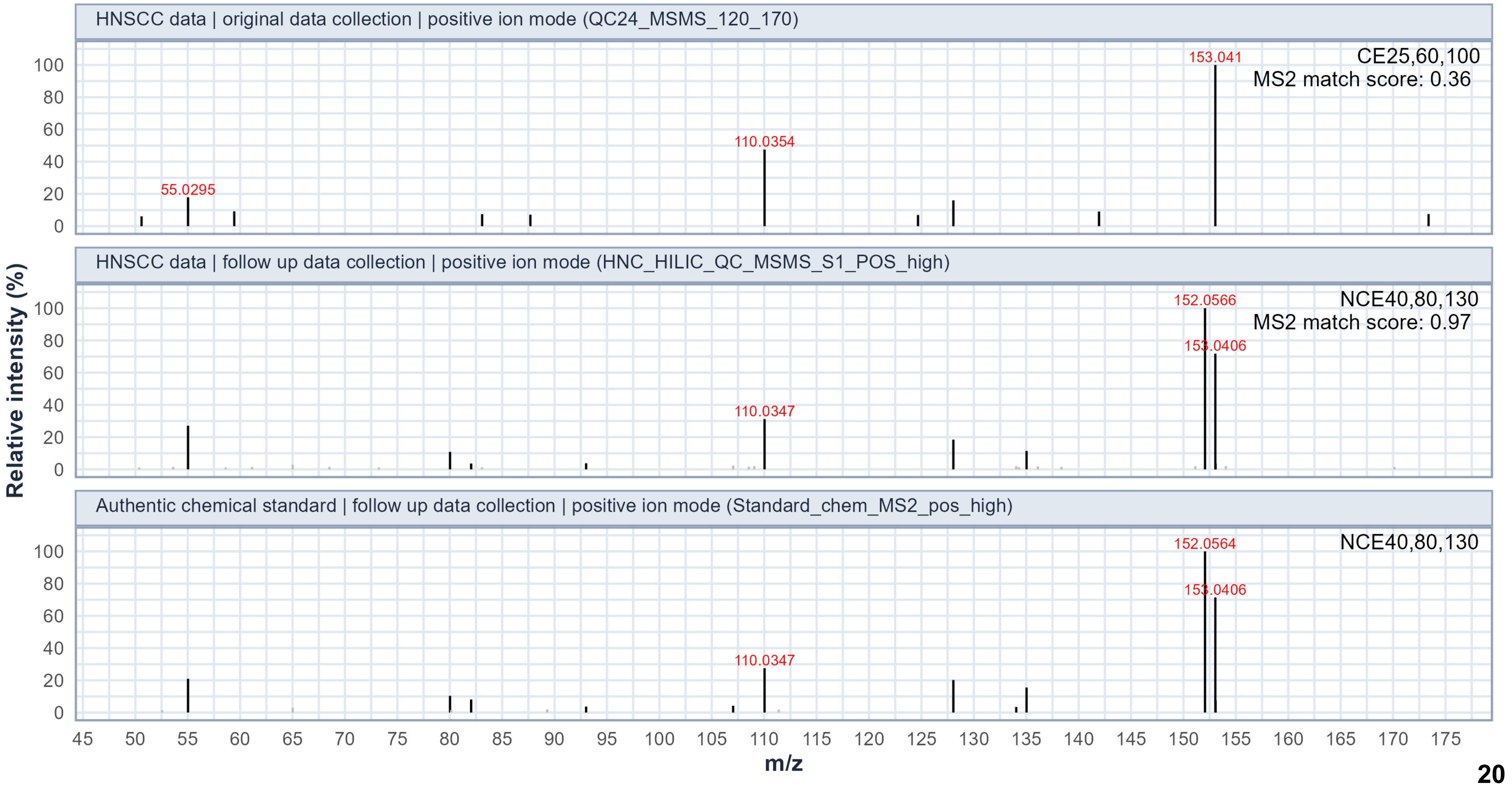

# Guanosine [M+H]+ | HMDB0000133

Positive ion mode: 284.0989 m/z | Instrument: QE focus

## Chromatogram

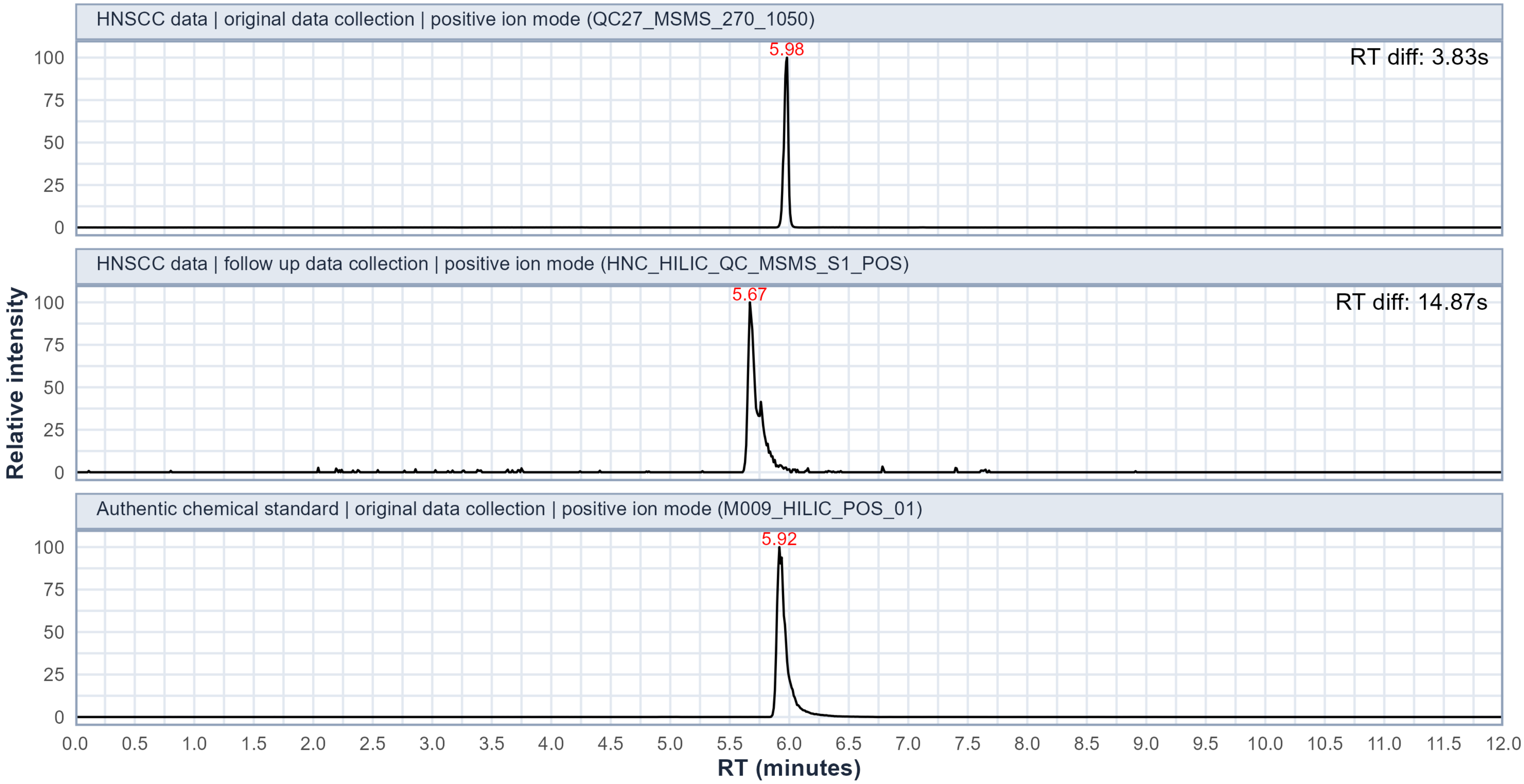


## MS/MS

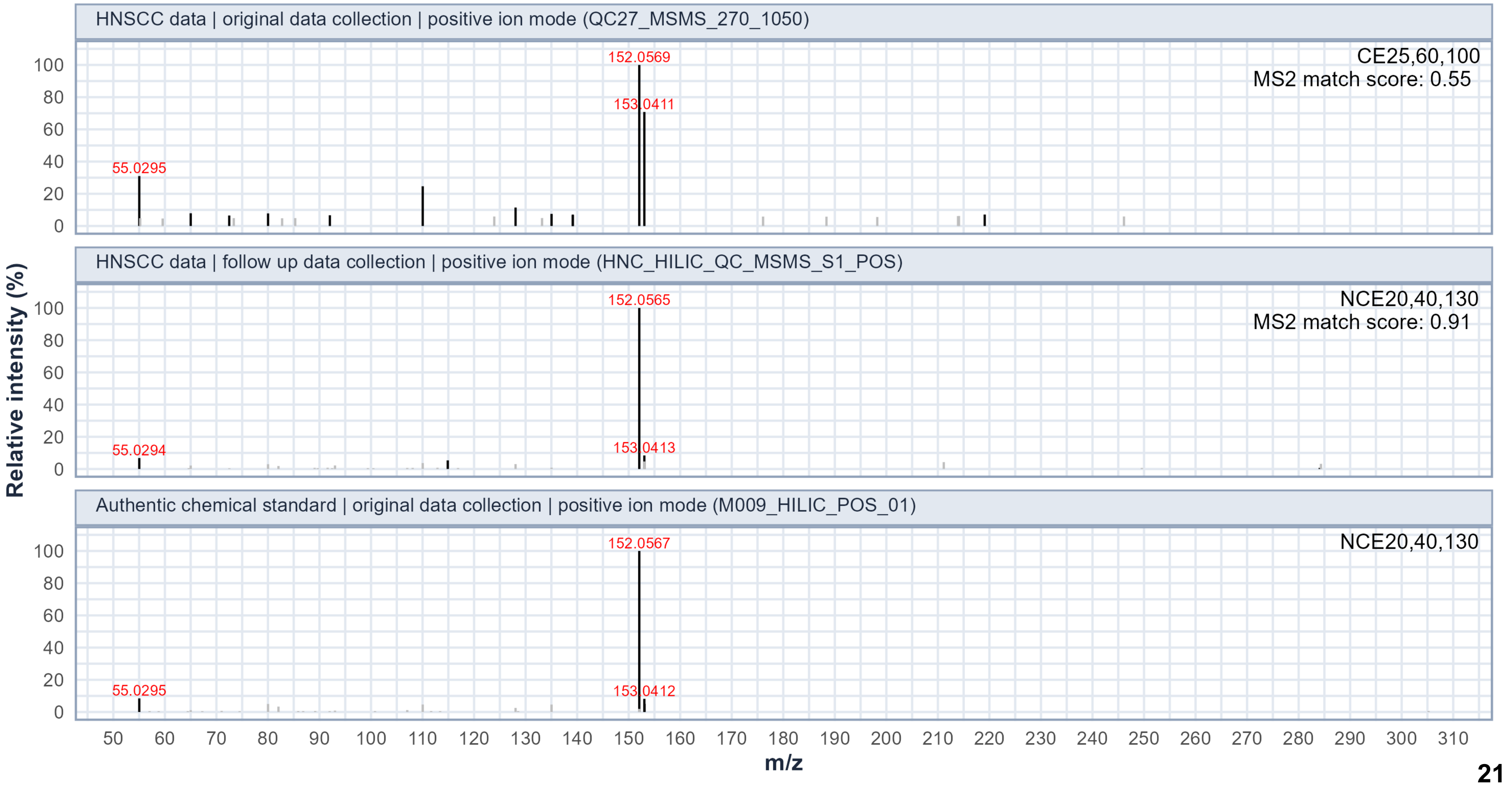

# Guanosine monophosphate, GMP [M-H]- | HMDB0001397

Negative ion mode: 362.0507 m/z | Instrument: QE focus

## Chromatogram

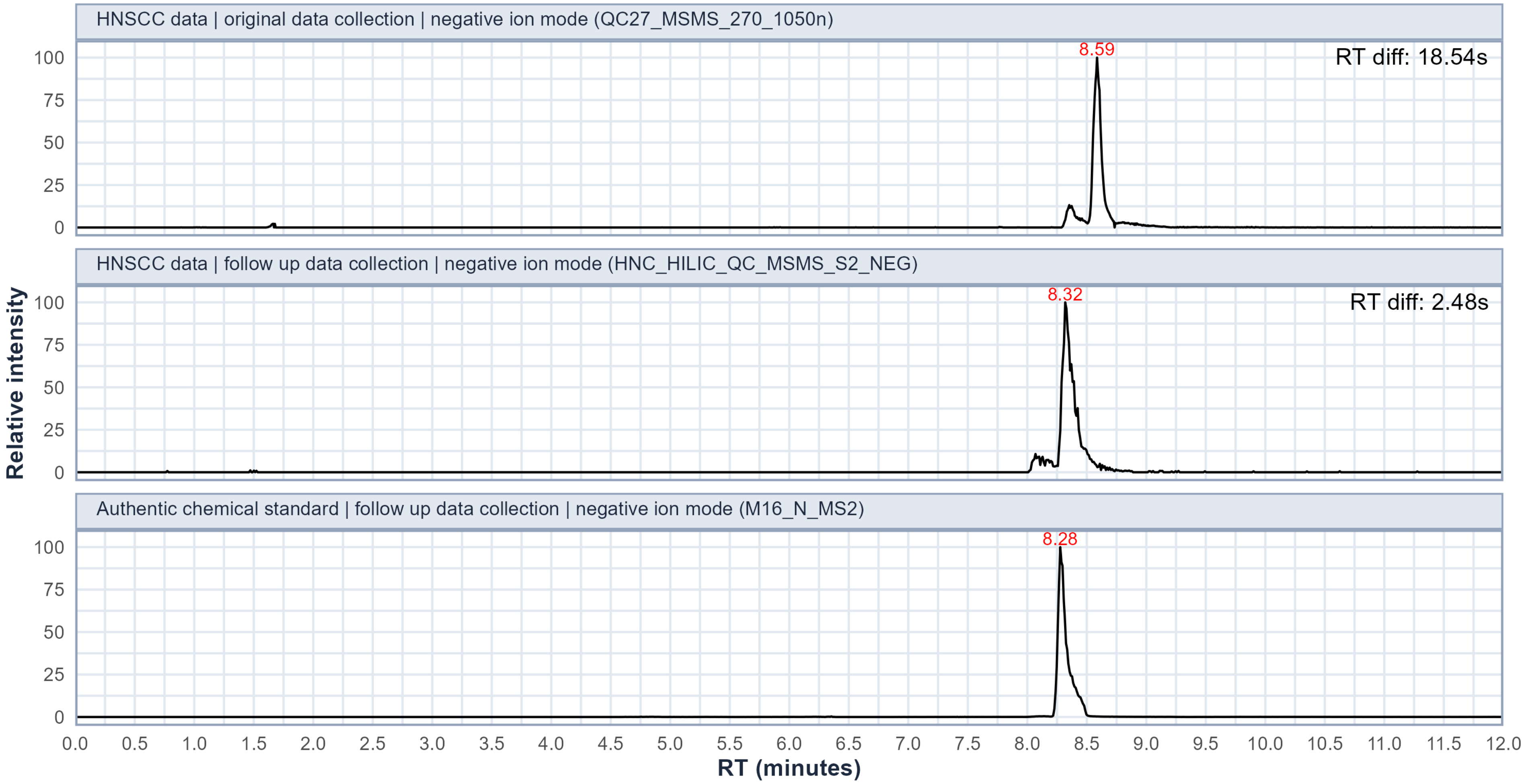


## MS/MS

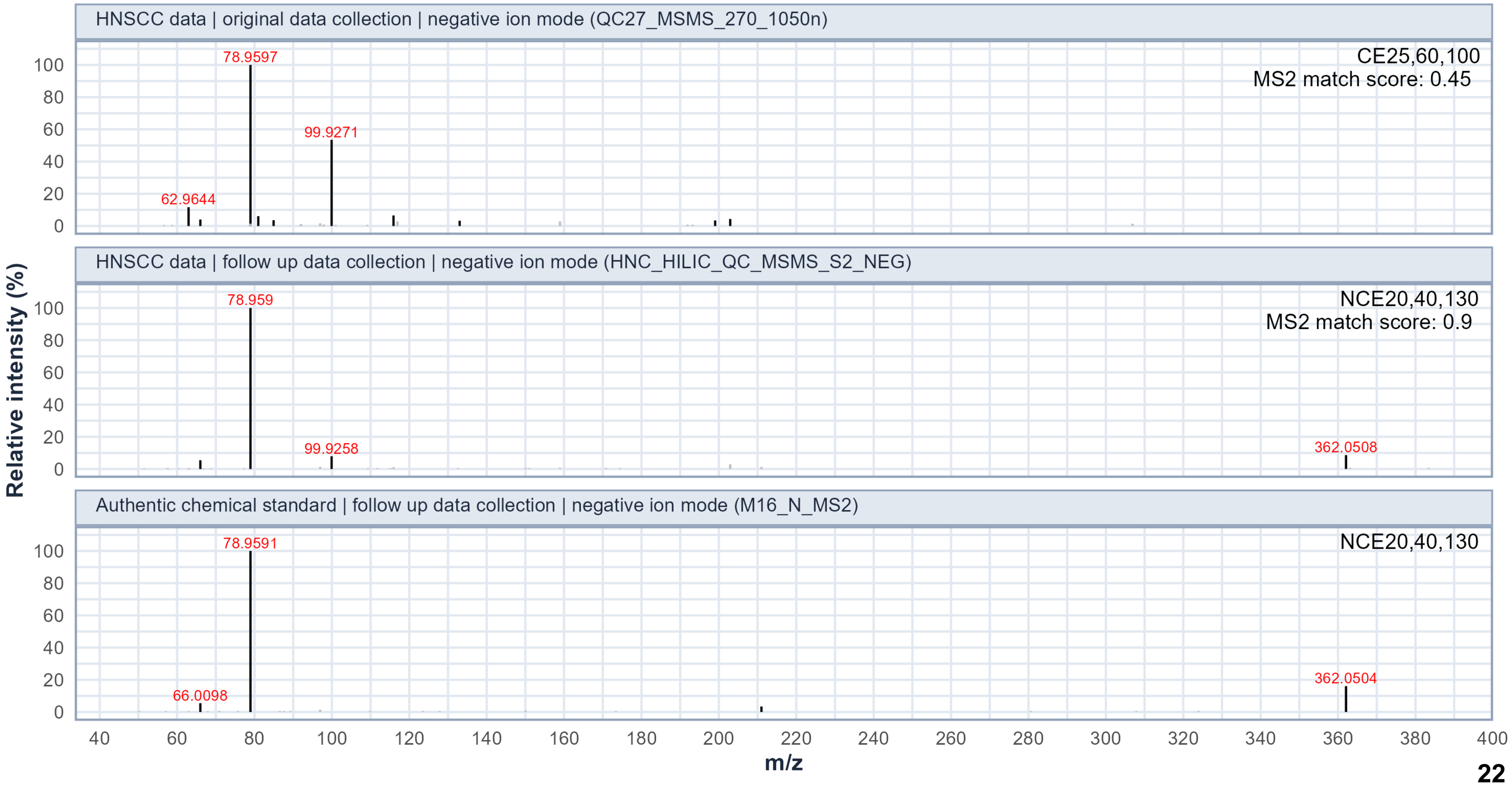

# Histamine [M+H]+ | HMDB0000870

Positive ion mode: 112.0869 m/z | Instrument: QE focus

## Chromatogram

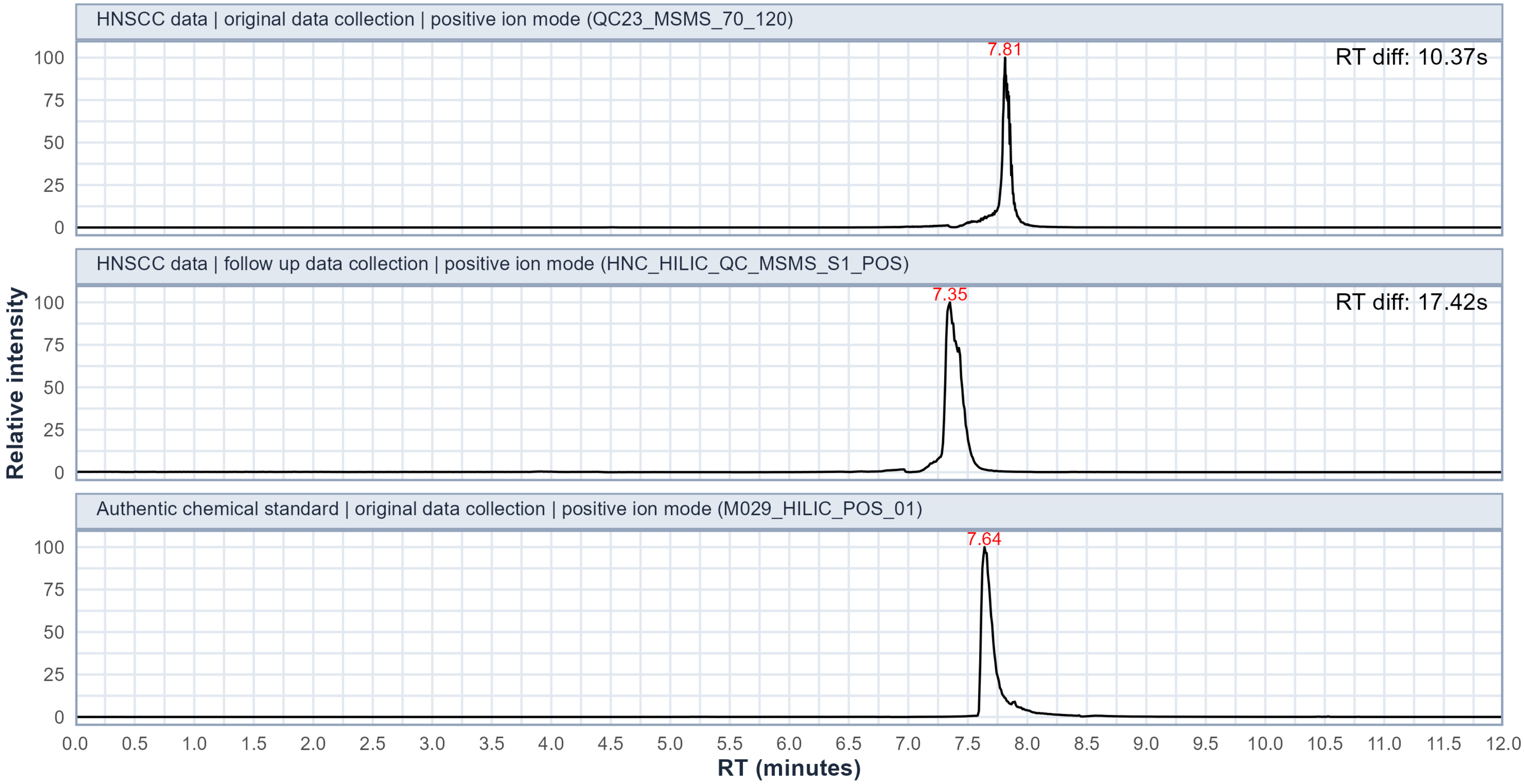


## MS/MS

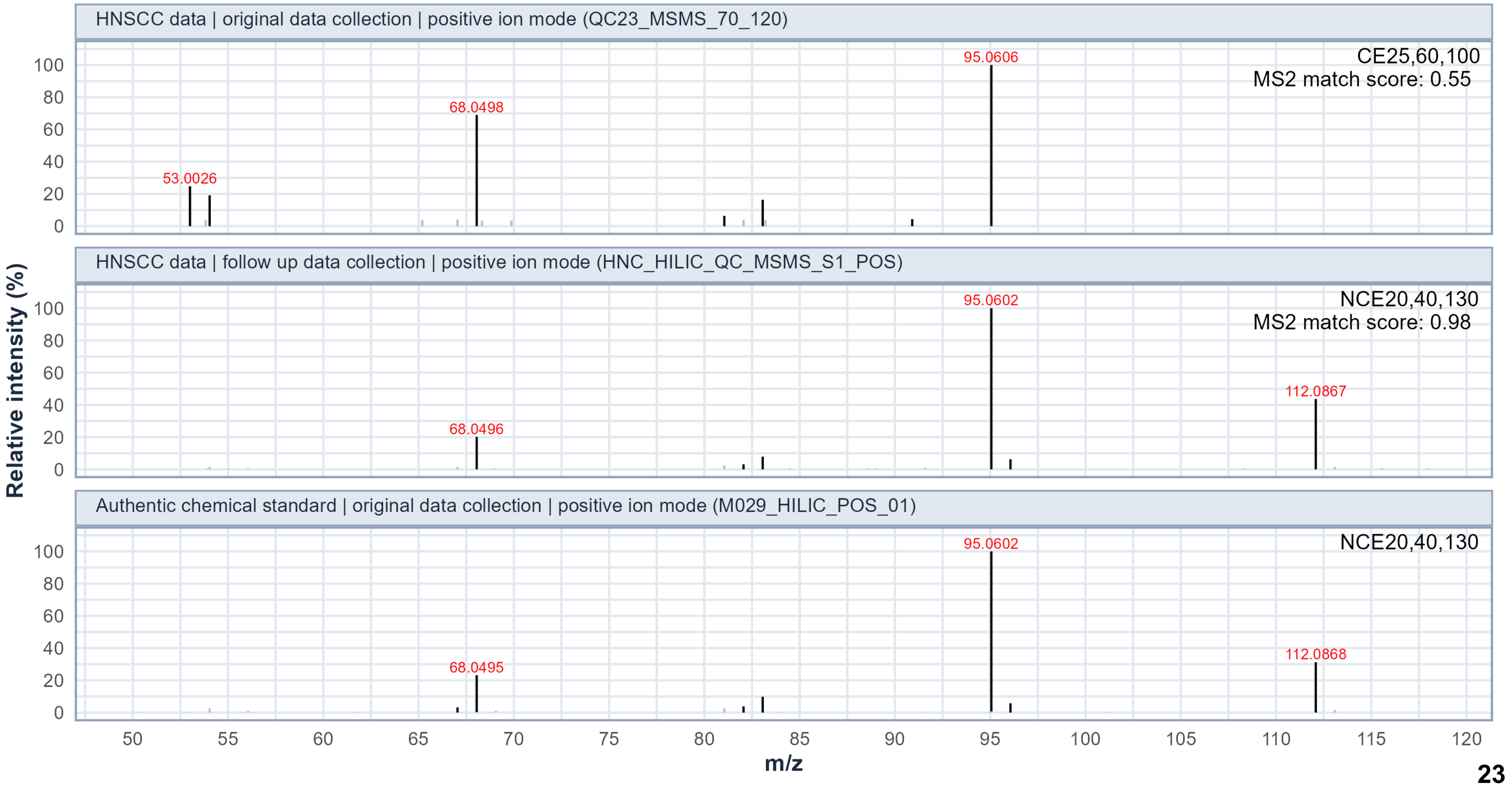

# Hypoxanthine [M+H]+ | HMDB0000157

Positive ion mode: 137.0458 m/z | Instrument: QE focus

## Chromatogram

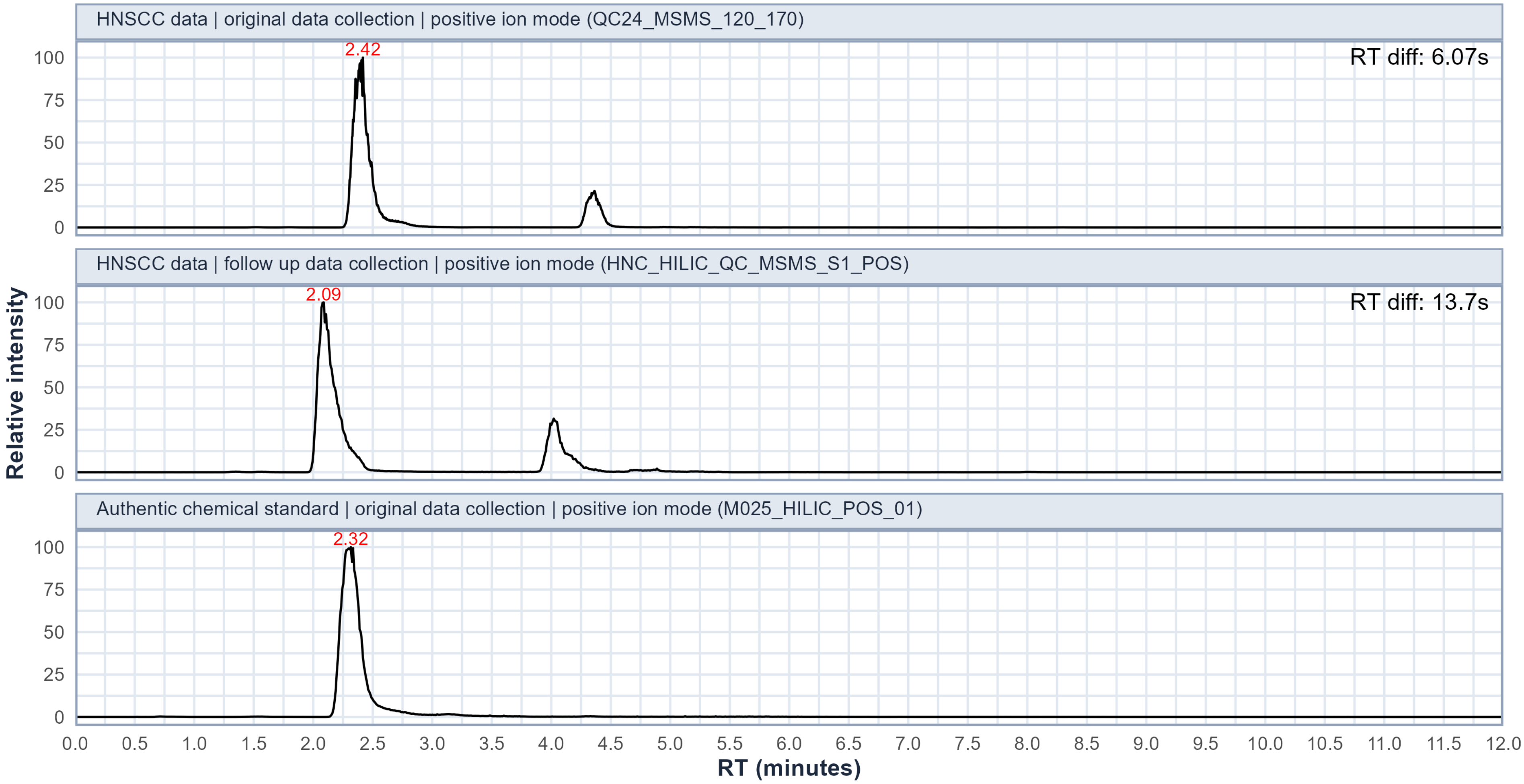


## MS/MS

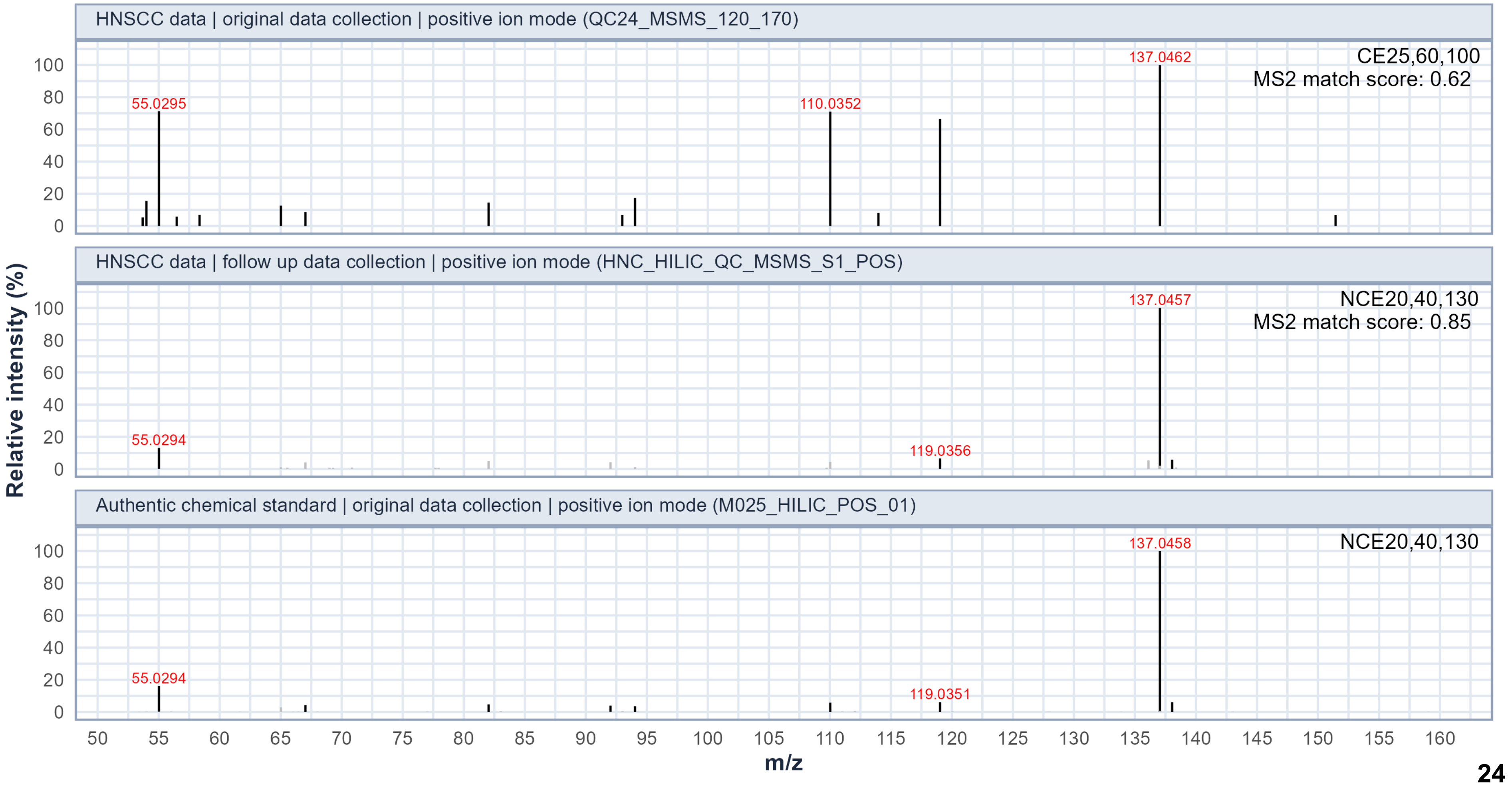

# Inosine [M+H]+ | HMDB0000195

Positive ion mode: 269.088 m/z | Instrument: QE focus

## Chromatogram

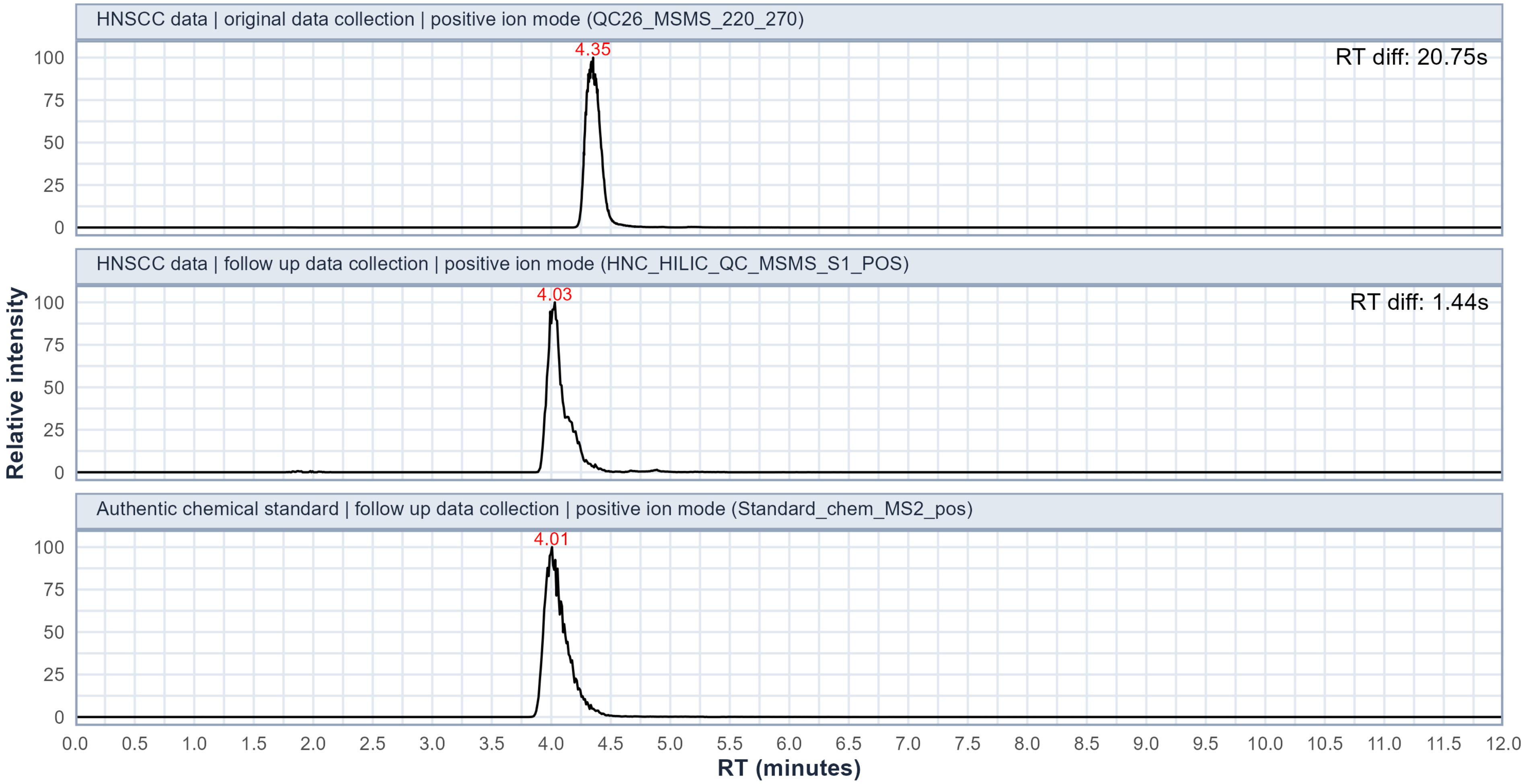


## MS/MS

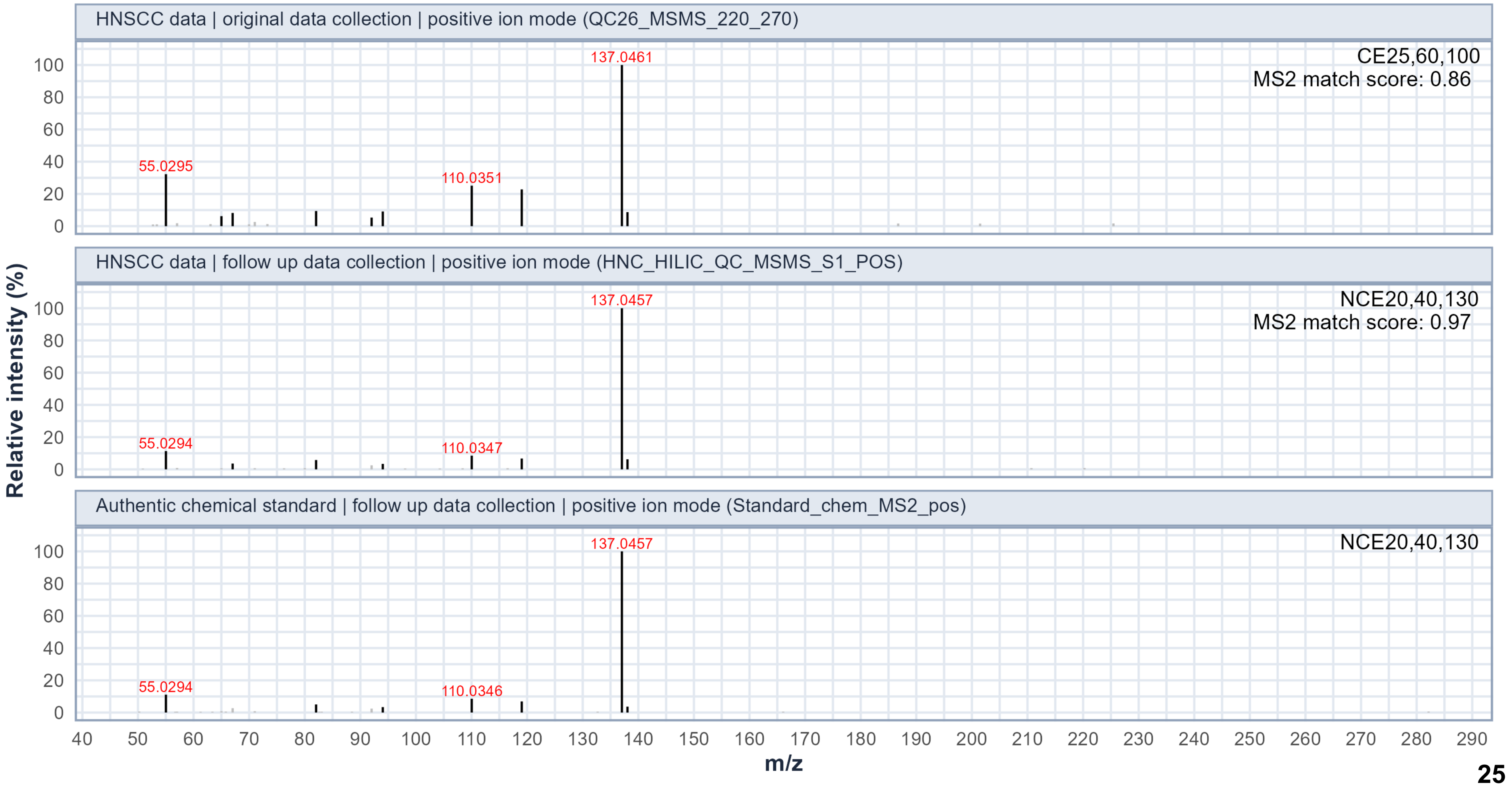

# Inosine monophosphate, IMP, Inosinic acid [M-H]- | HMDB0000175

Negative ion mode: 347.0398 m/z | Instrument: QE focus

## Chromatogram

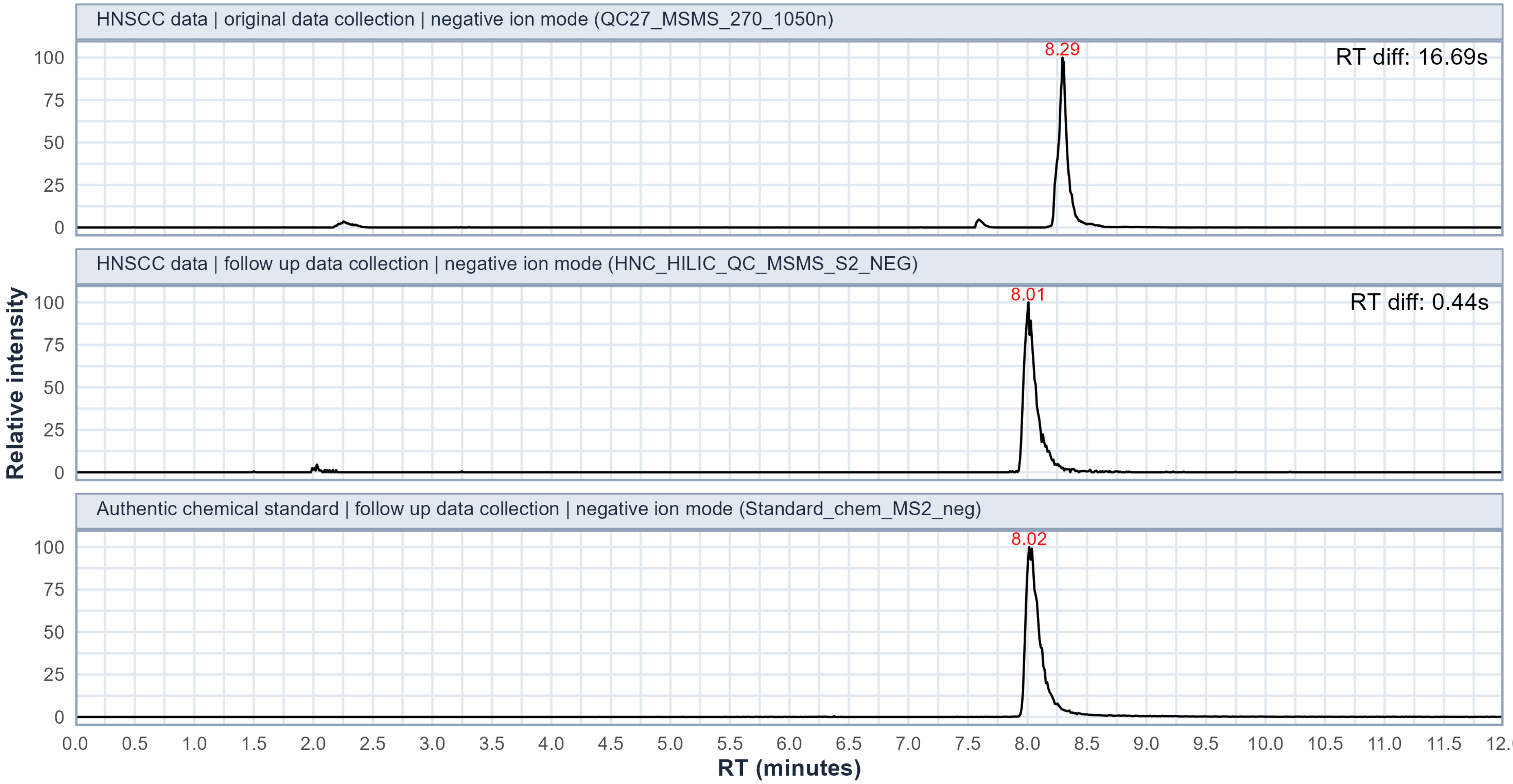


## MS/MS

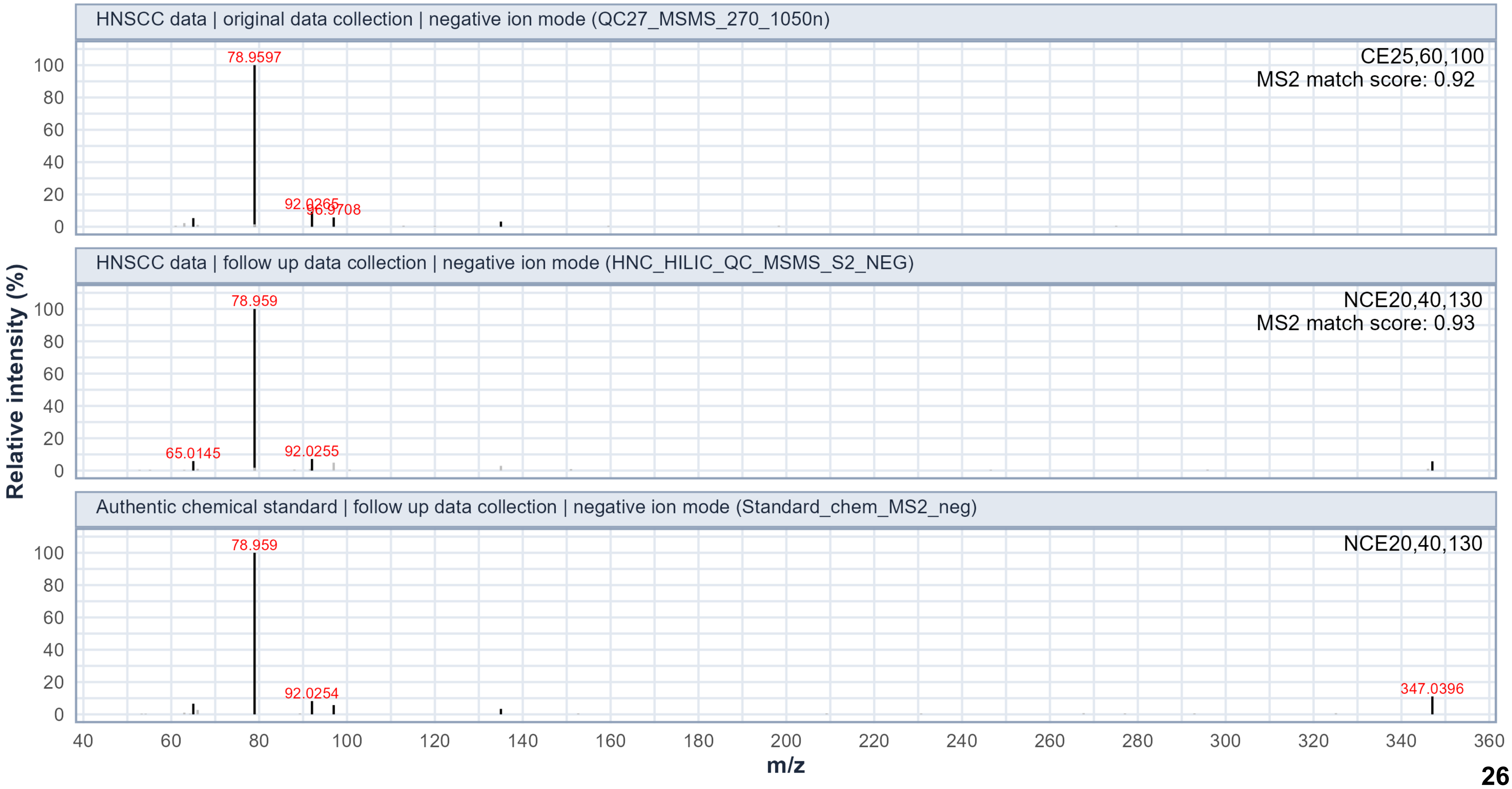

# L-Acetylcarnitine [M+H]+ | HMDB0000201

Positive ion mode: 204.123 m/z | Instrument: QE focus

## Chromatogram

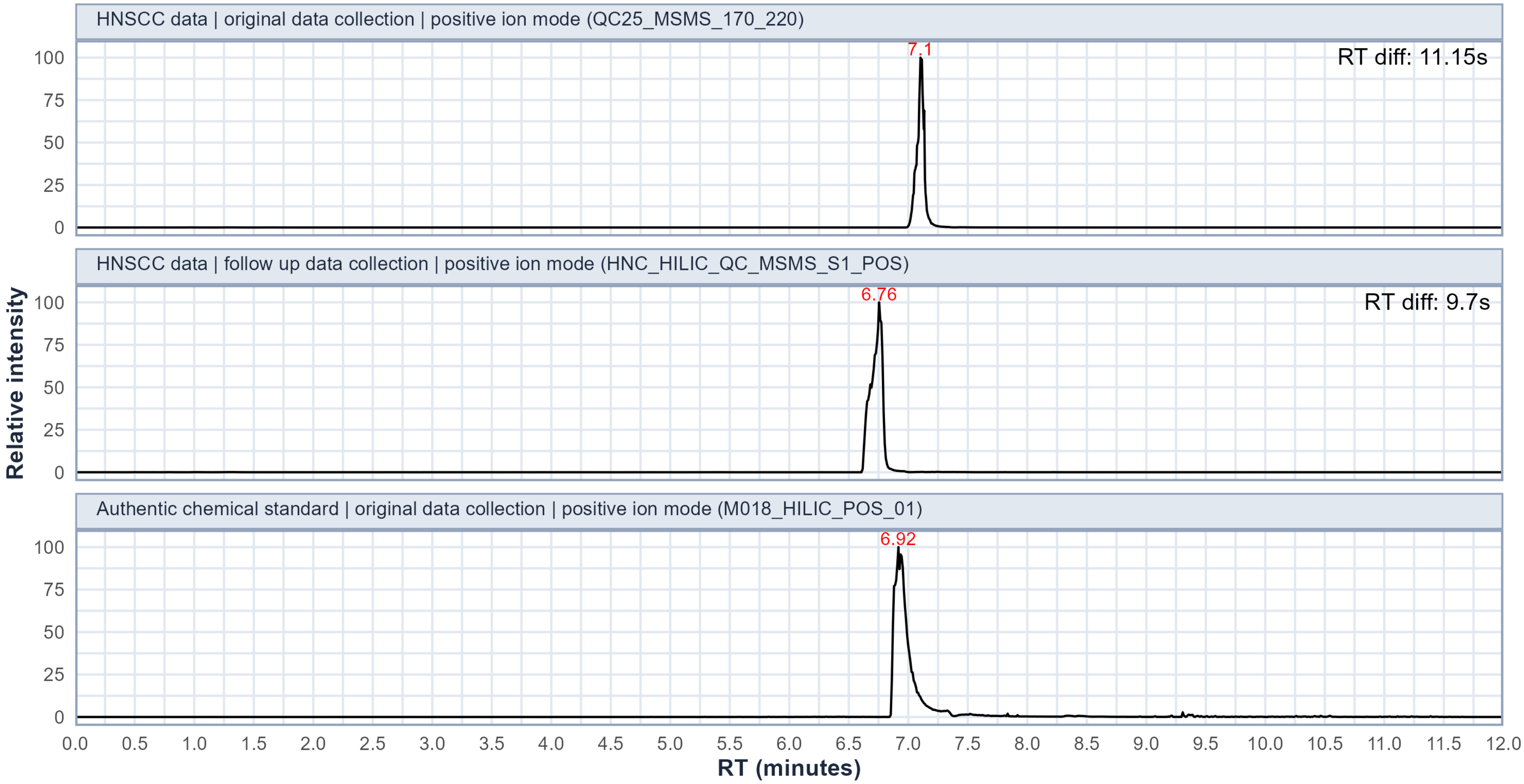


## MS/MS

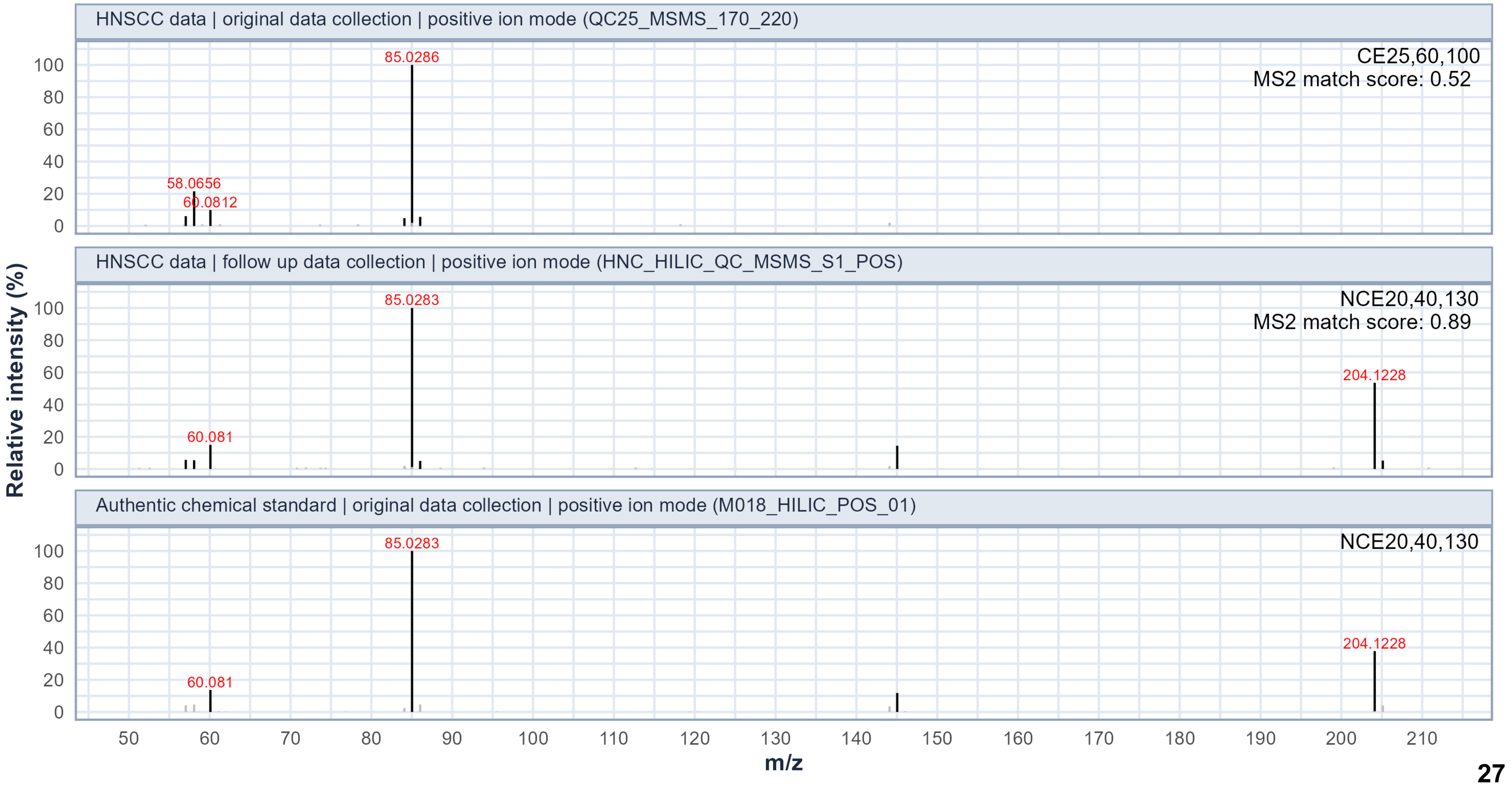

# L-Alanine [M+H]+ | HMDB0000161

Positive ion mode: 90.055 m/z | Instrument: QE focus
MS/MS data for L-alanine is unreliable because it fragments poorly; identification is based on retention time match only.

## Chromatogram

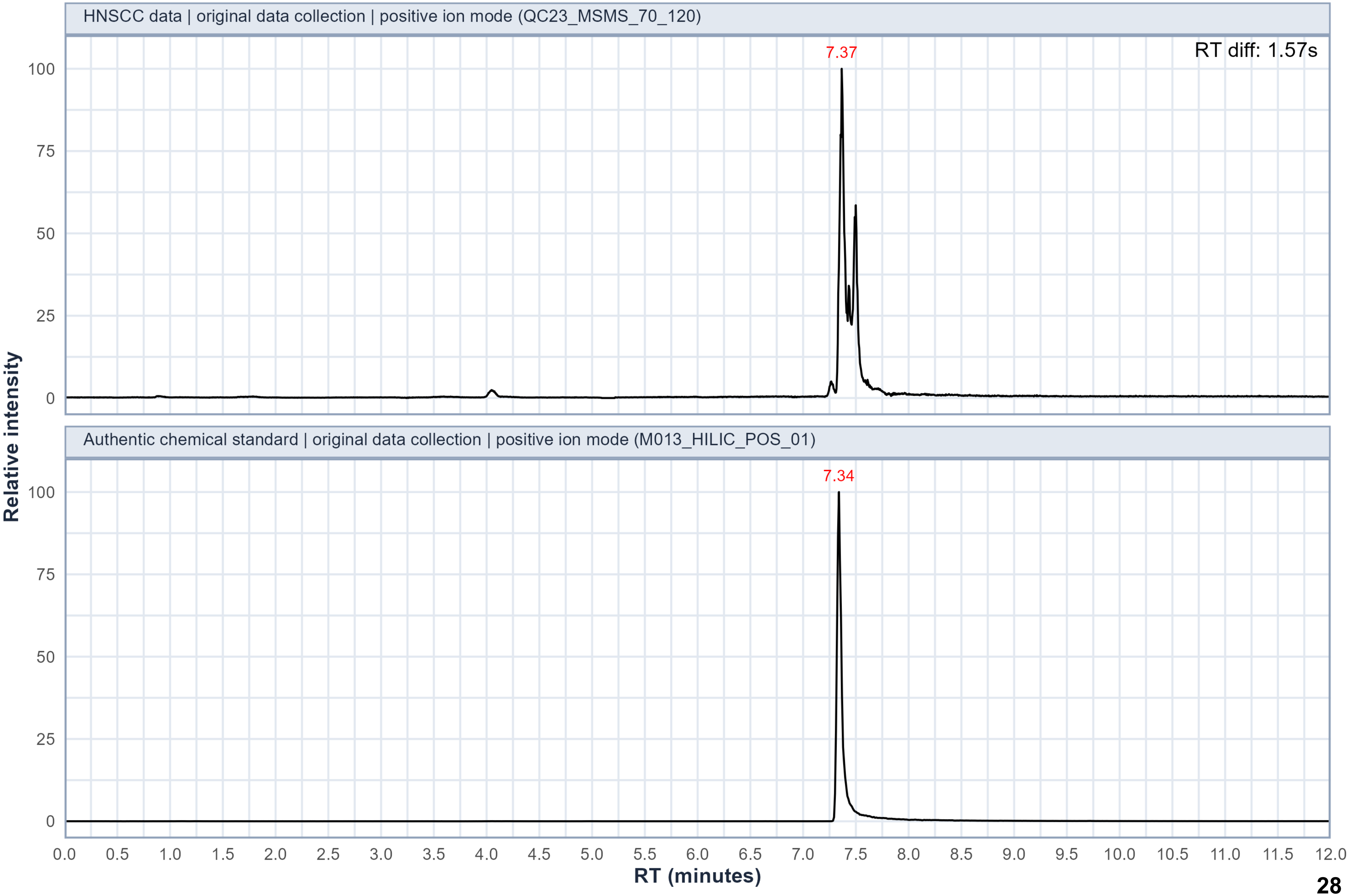

# L-Alanine [M+H]+ | HMDB0000161

Positive ion mode: 90.055 m/z | Instrument: QE focus
Other common biological metabolites that share the same m/z as L-alanine are shown; identification is indicated by retention time match.

## Chromatogram

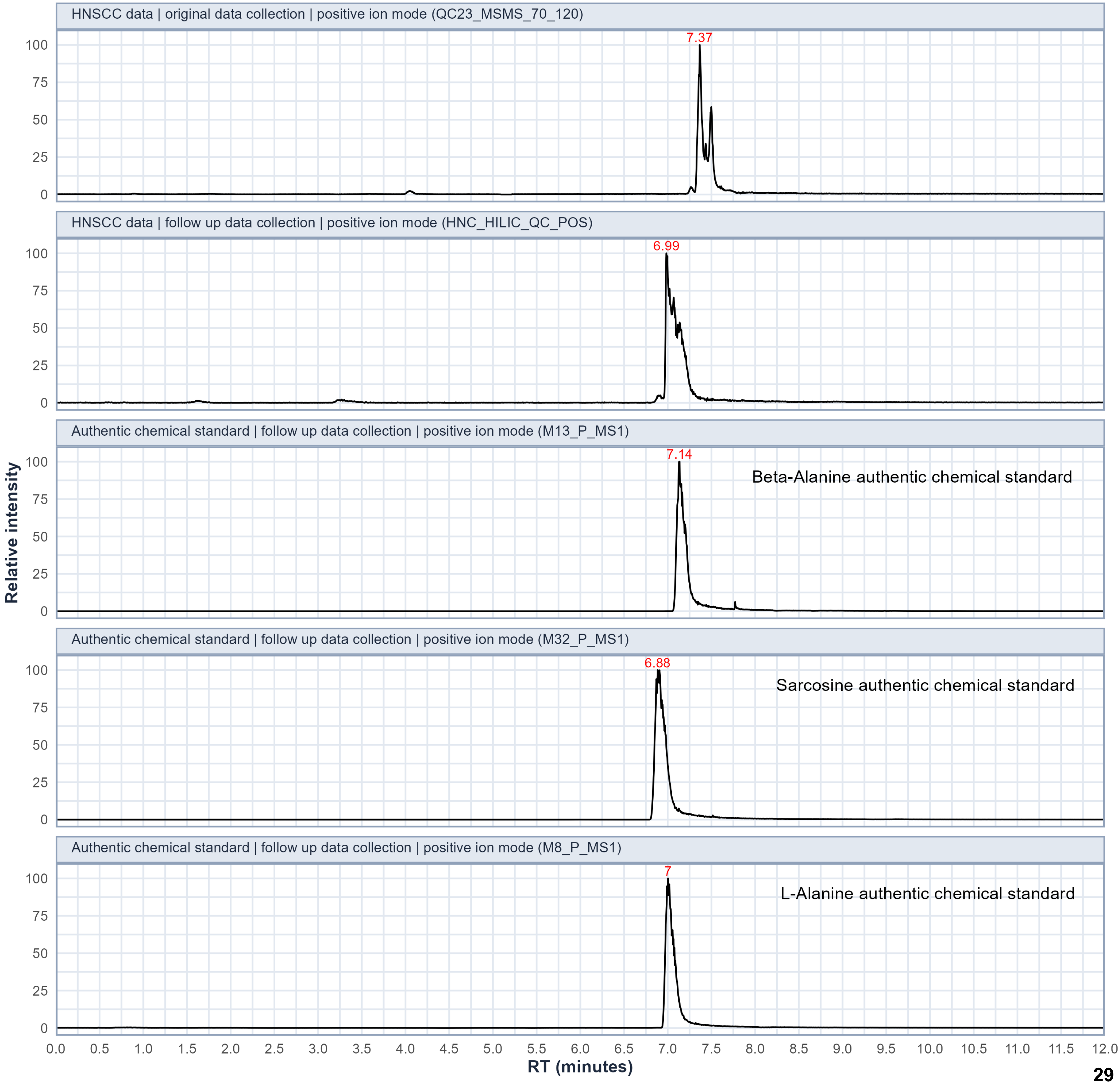

# L-Arginine [M+H]+ | HMDB0000161

Positive ion mode: 175.119 m/z | Instrument: QE focus
Other common biological metabolites that share the same m/z as L-alanine are shown; identification is indicated by retention time match.

## Chromatogram

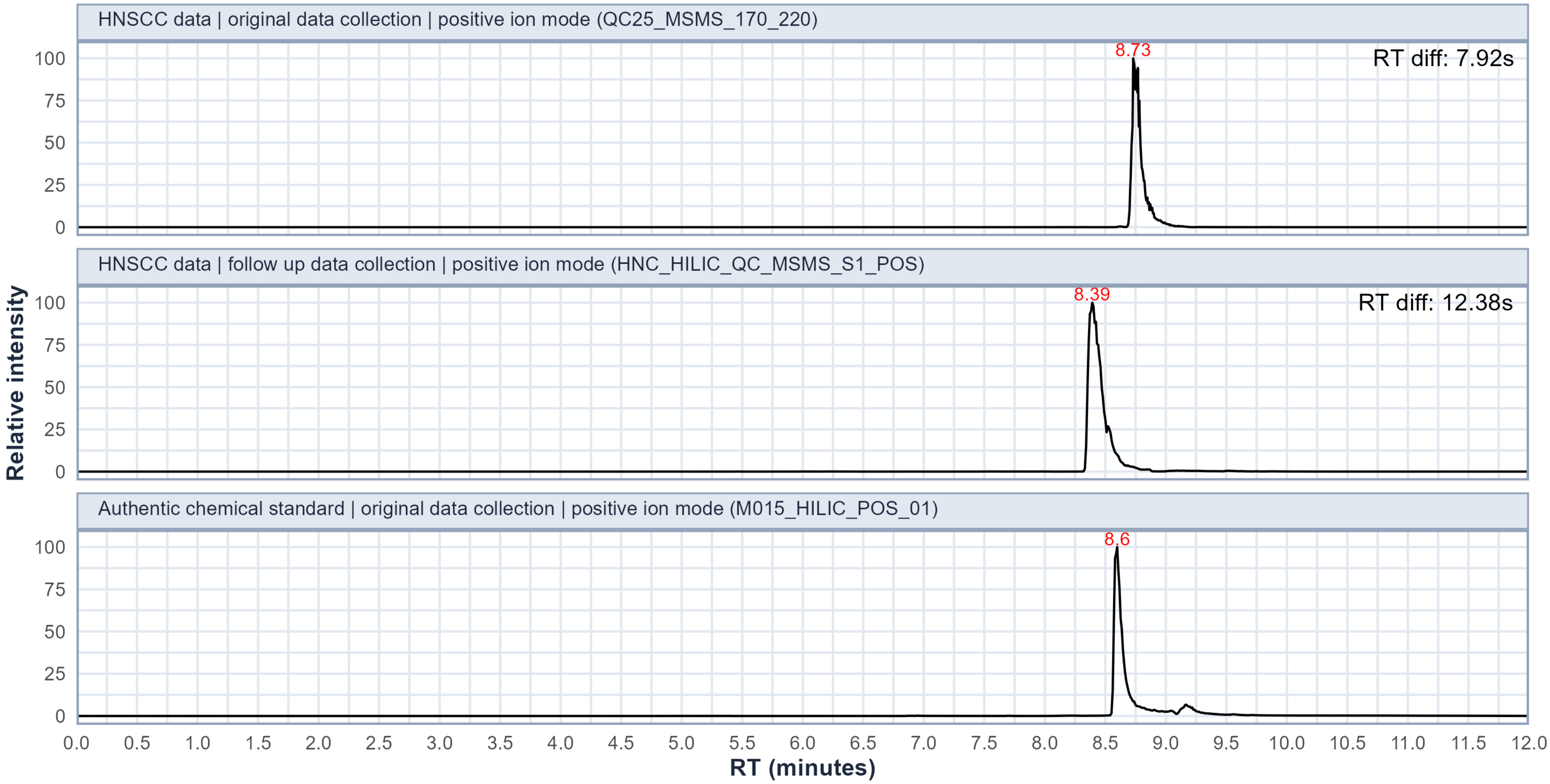


## MS/MS

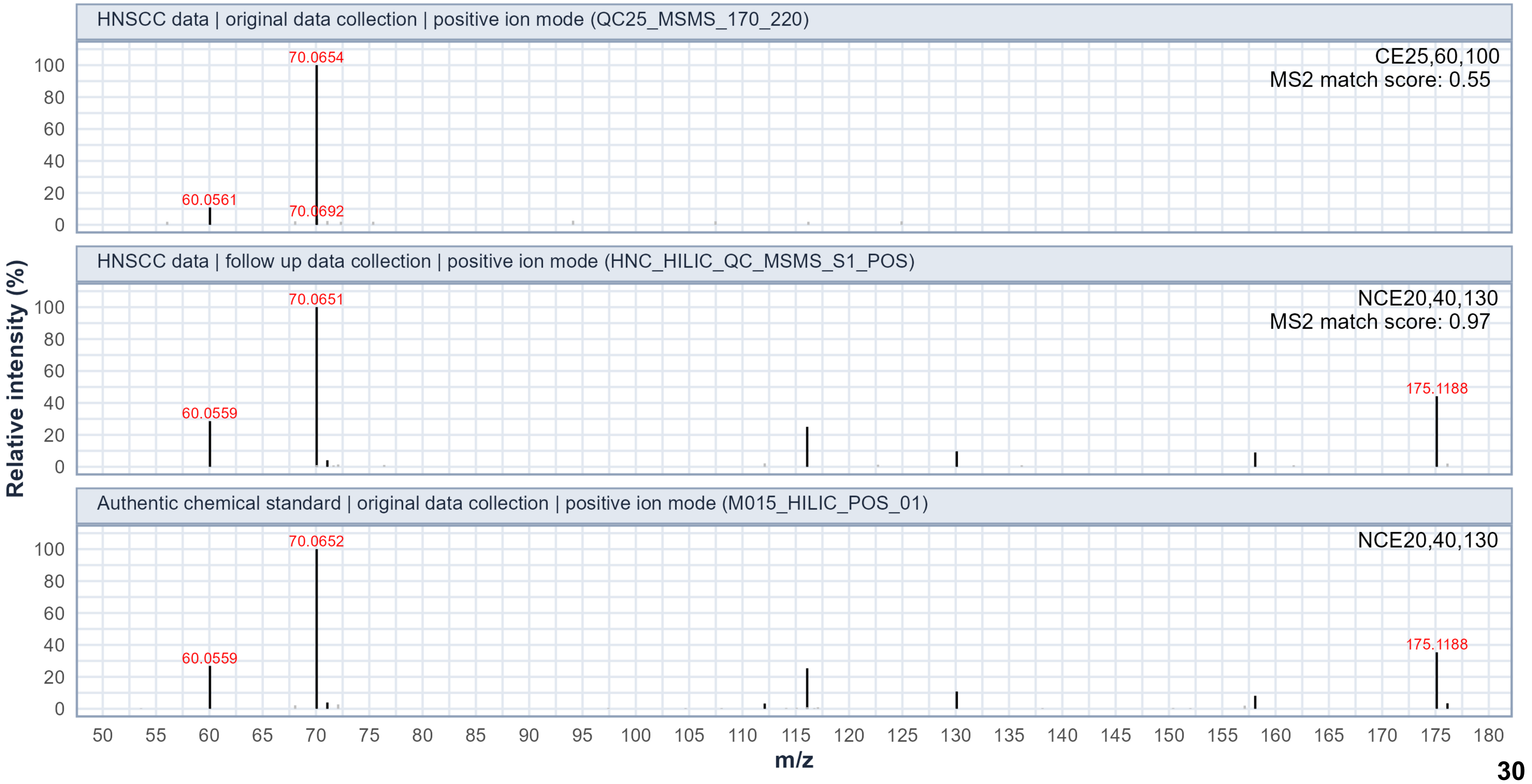

# L-Cystathionine [M+H]+ | HMDB0000099

Positive ion mode: 223.0747 m/z | Instrument: QE focus

## Chromatogram

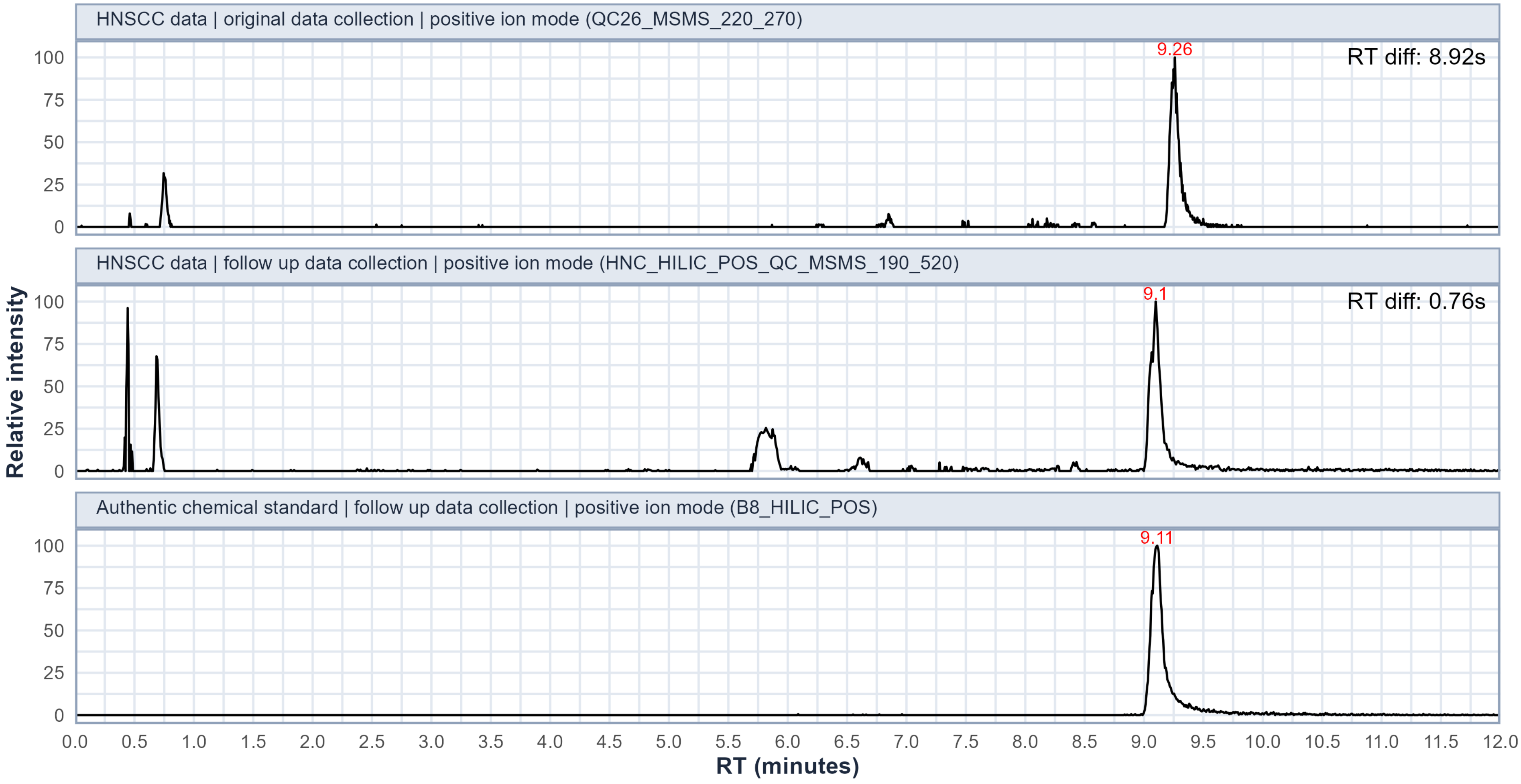


## MS/MS

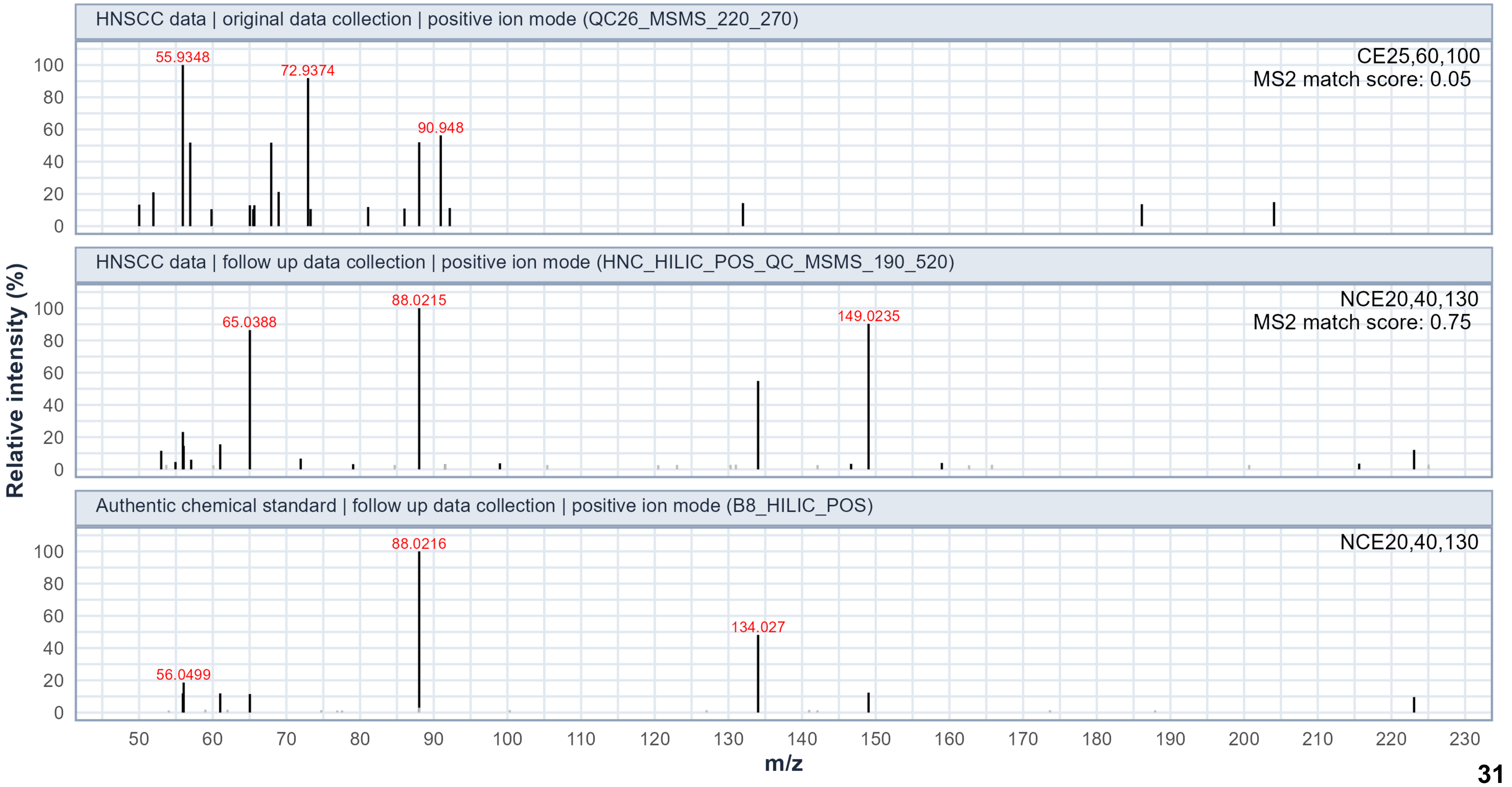

# L-Glutamic acid [M-H]- | HMDB0000148

Negative ion mode: 146.0459 m/z | Instrument: QE focus

## Chromatogram

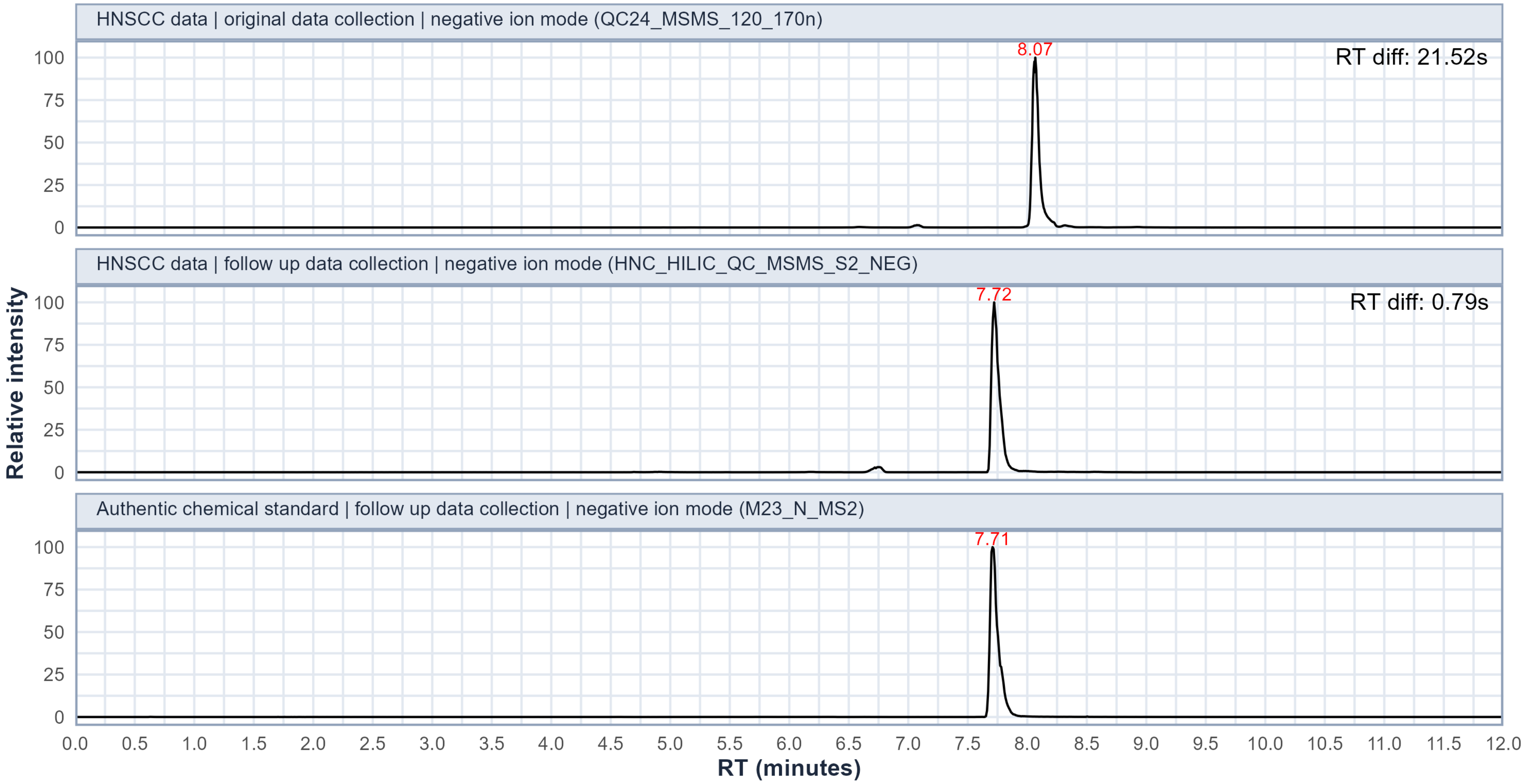


## MS/MS

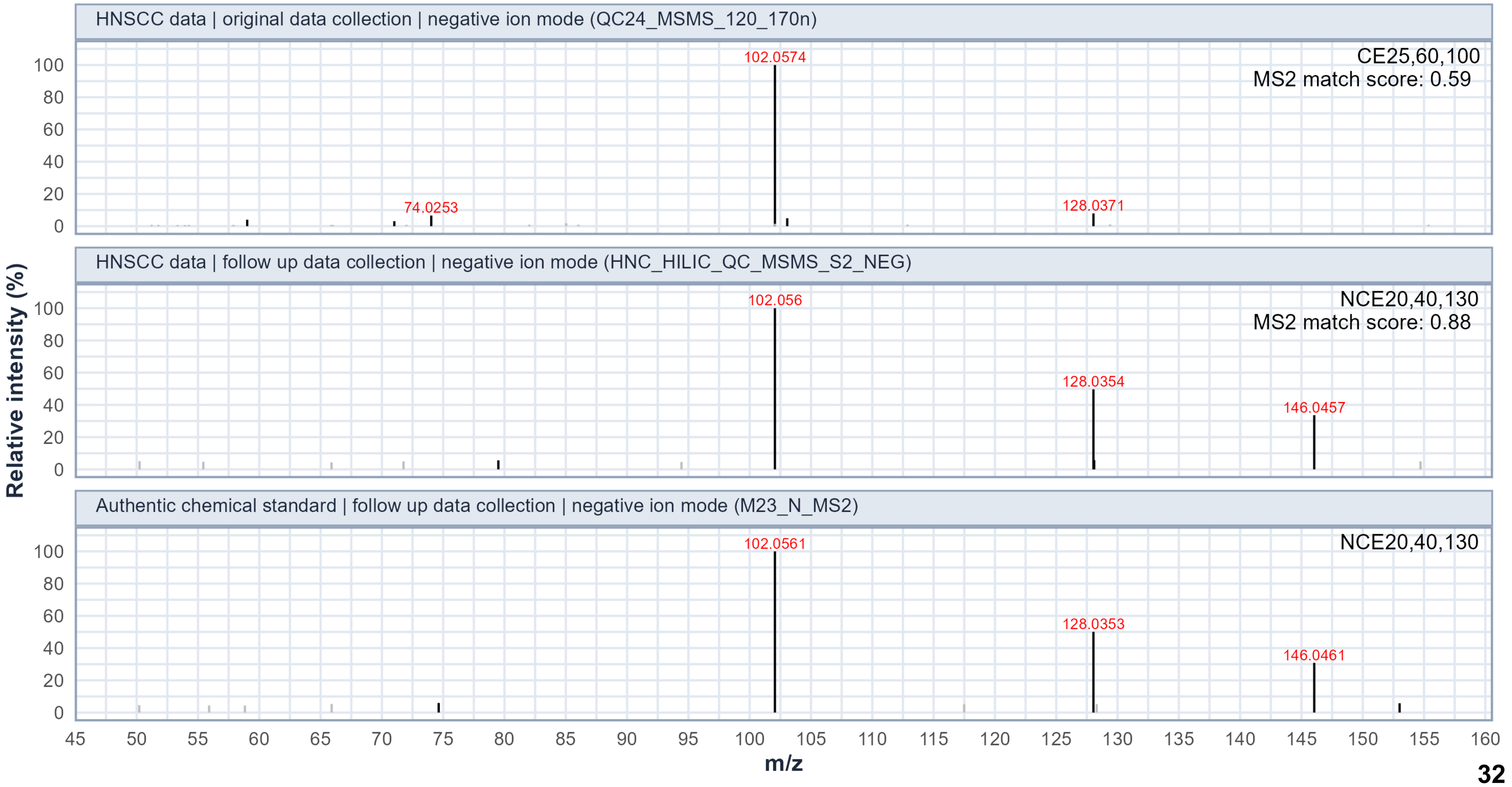

# L-Glutamine [M+H]+ | HMDB0000641

Positive ion mode: 147.0764 m/z | Instrument: QE focus

## Chromatogram

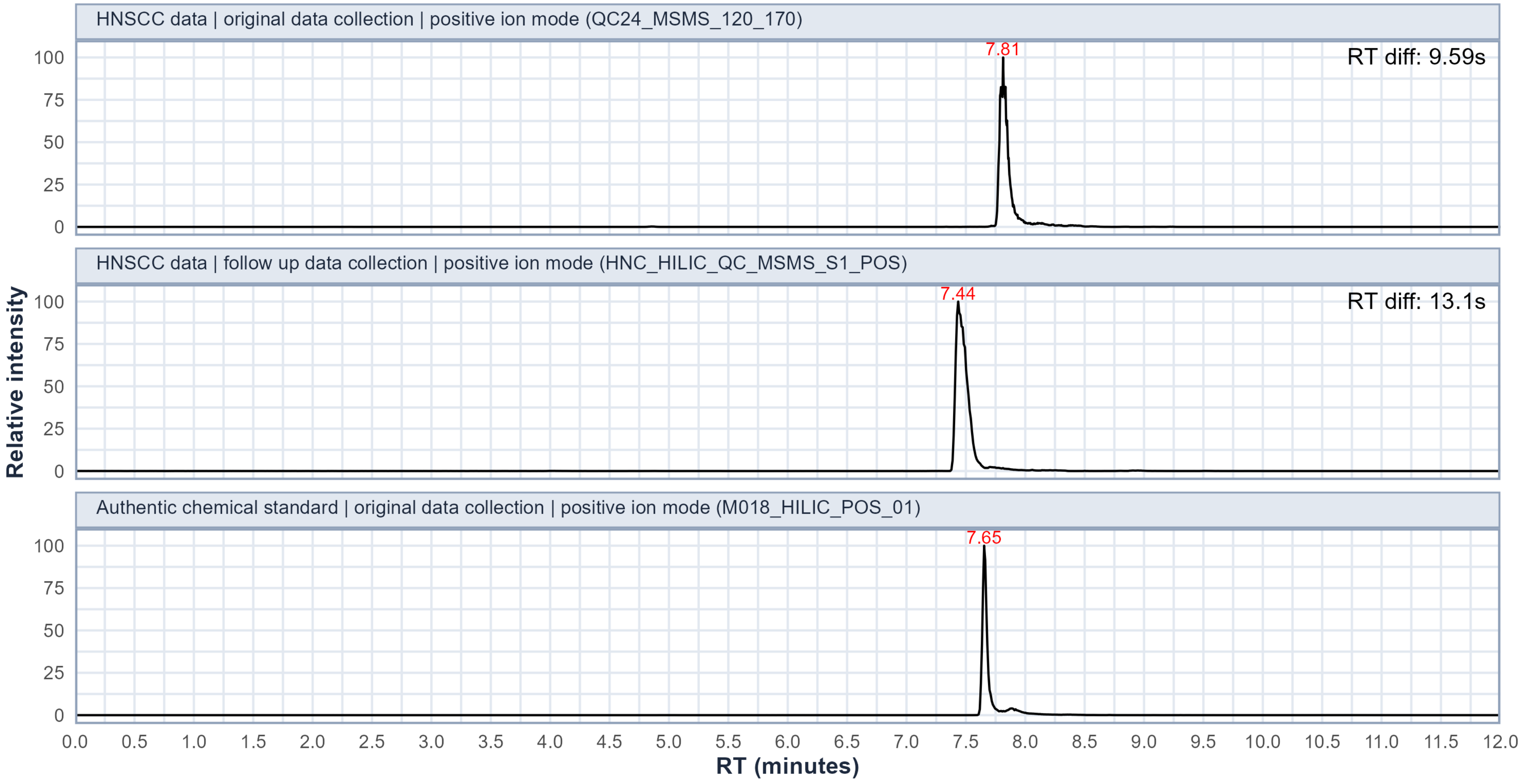


## MS/MS

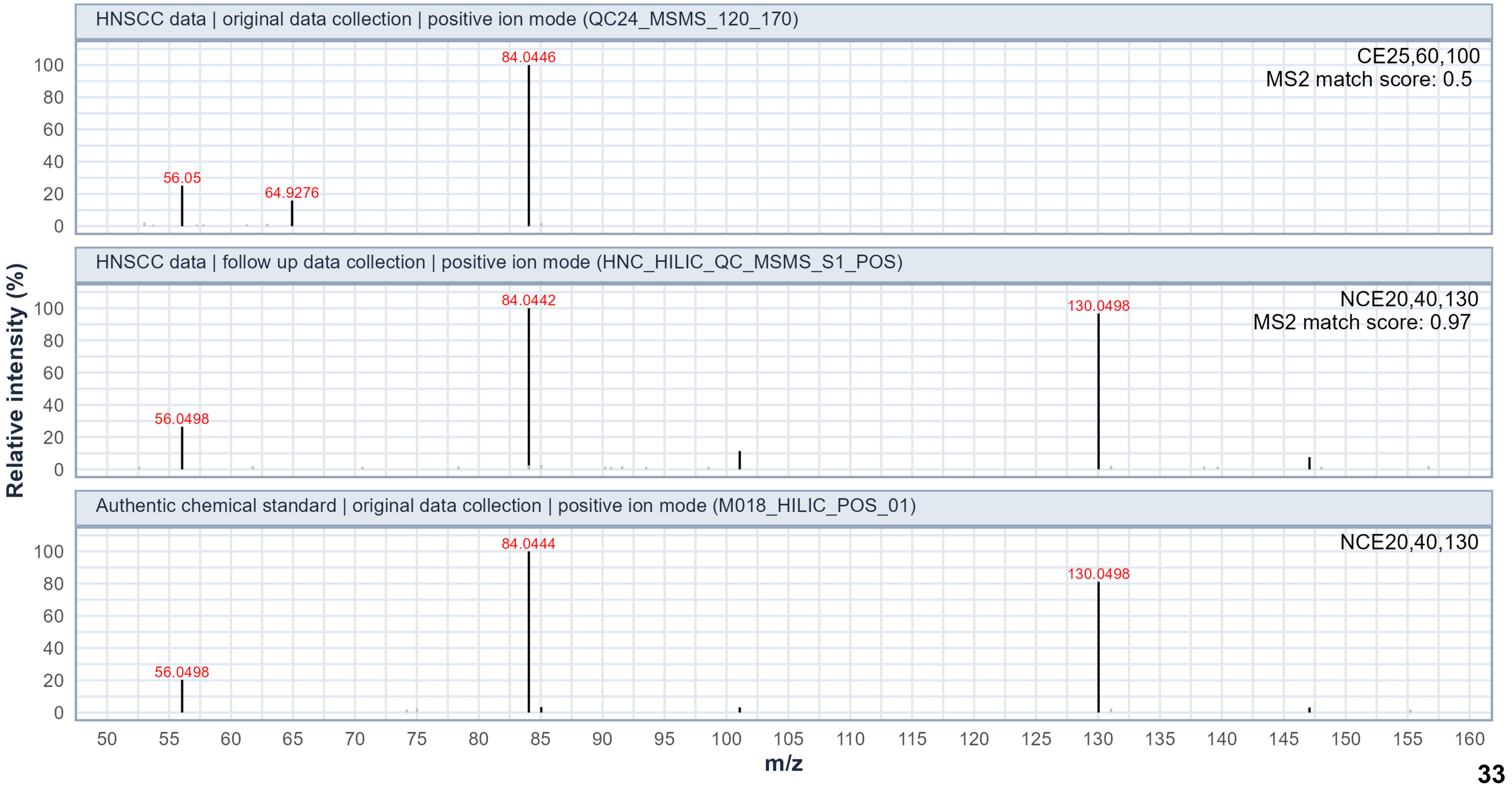

# L-Glycine [M+H]+ | HMDB0000123

Positive ion mode: 76.0393 m/z | Instrument: QE focus
MS/MS data for L-glycine is unreliable because it fragments poorly; identification is based on retention time match only.

## Chromatogram

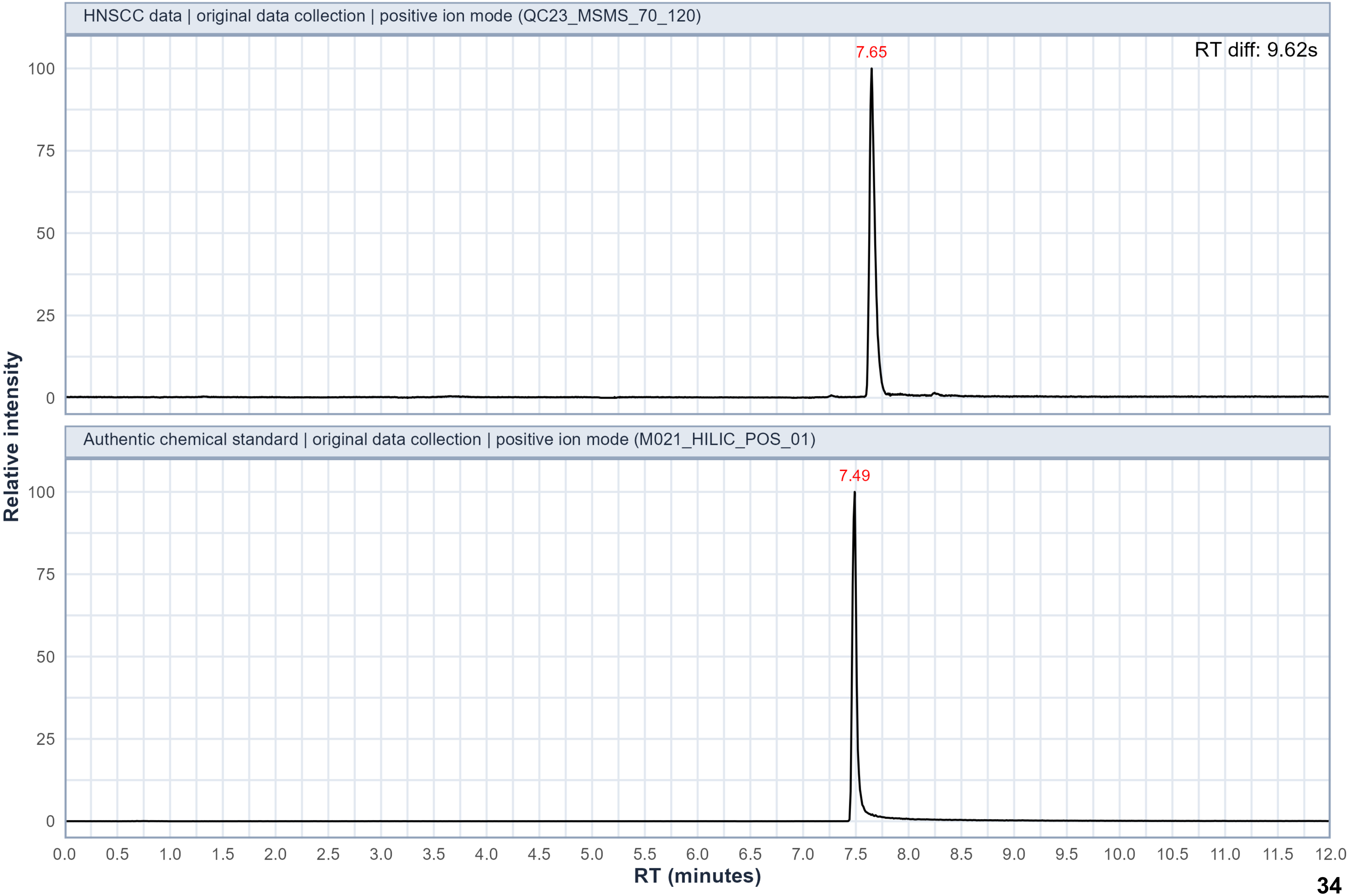

# L-Histidine [M+H]+ | HMDB0000177

Positive ion mode: 156.0768 m/z | Instrument: QE focus

## Chromatogram

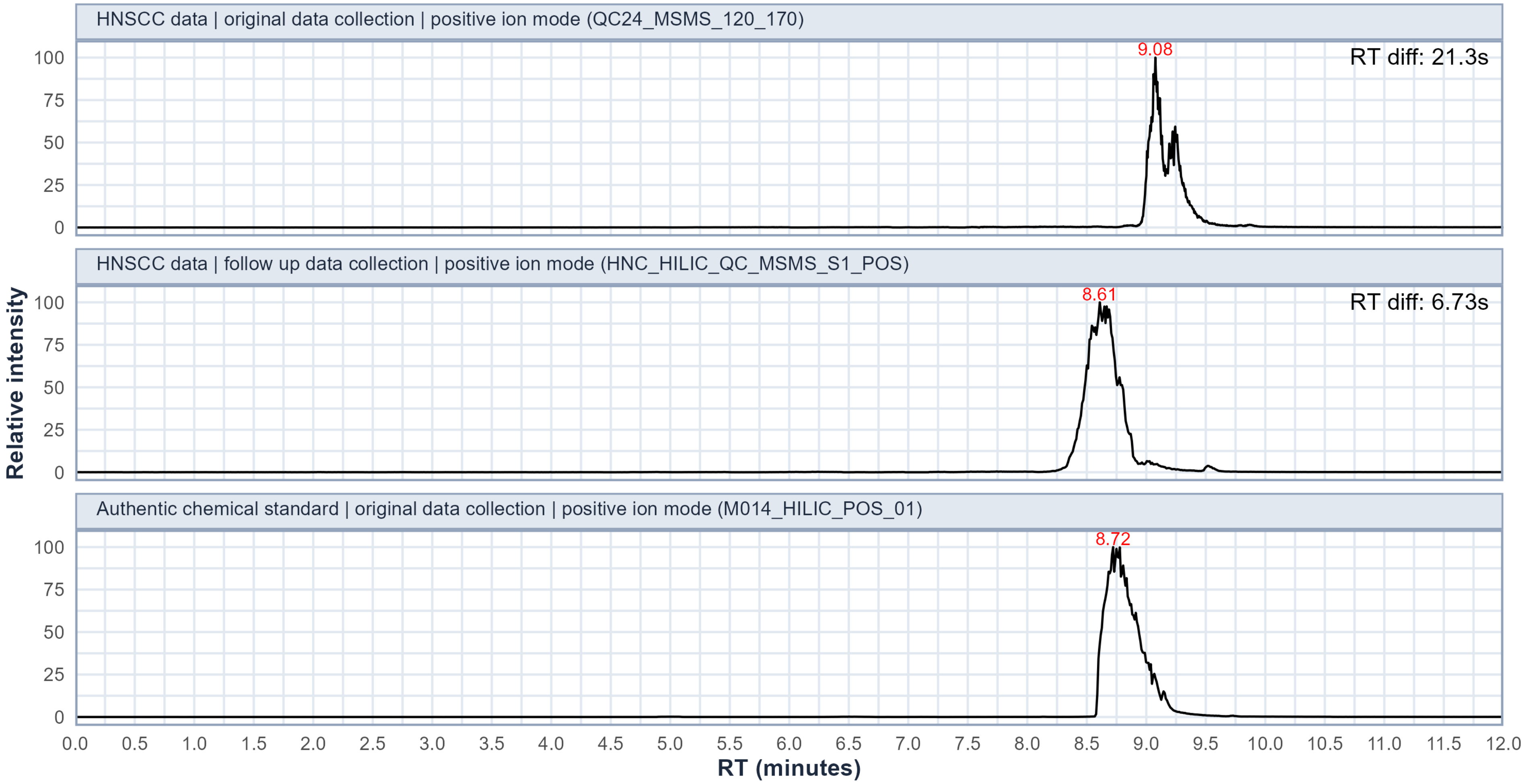


## MS/MS

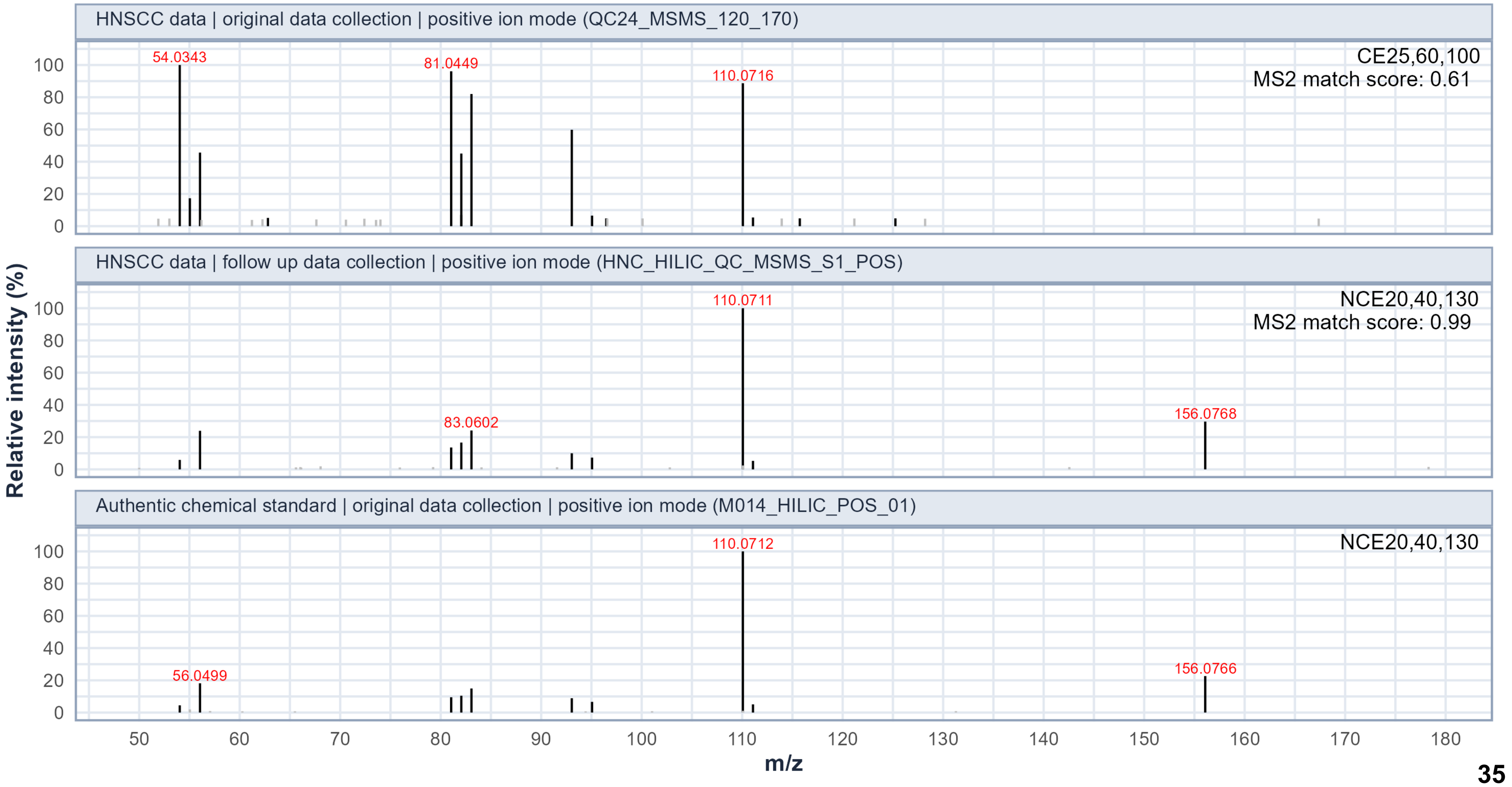

# L-Kynurenine [M+H]+ | HMDB0000684

Positive ion mode: 209.0921 m/z | Instrument: QE focus

## Chromatogram

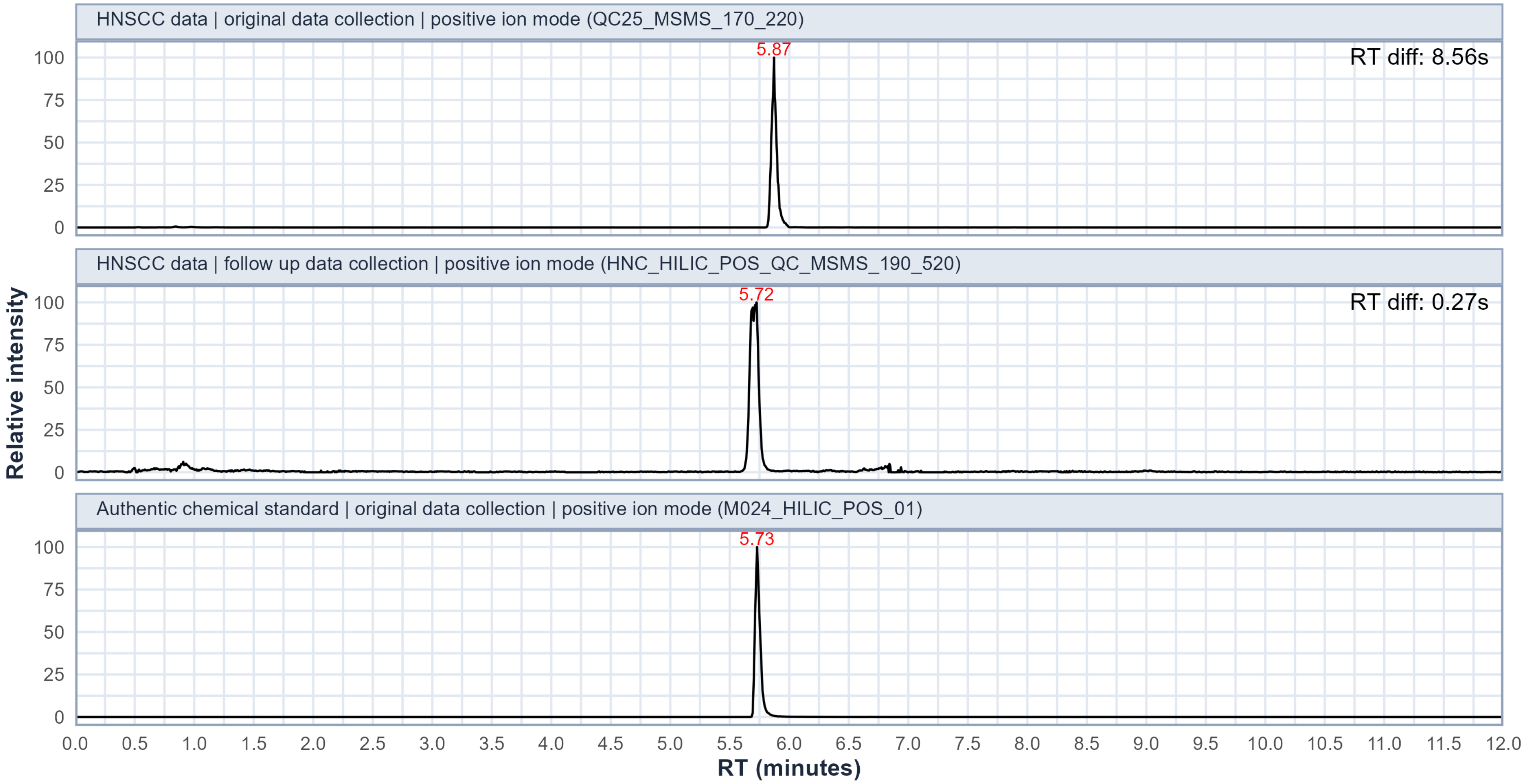


## MS/MS

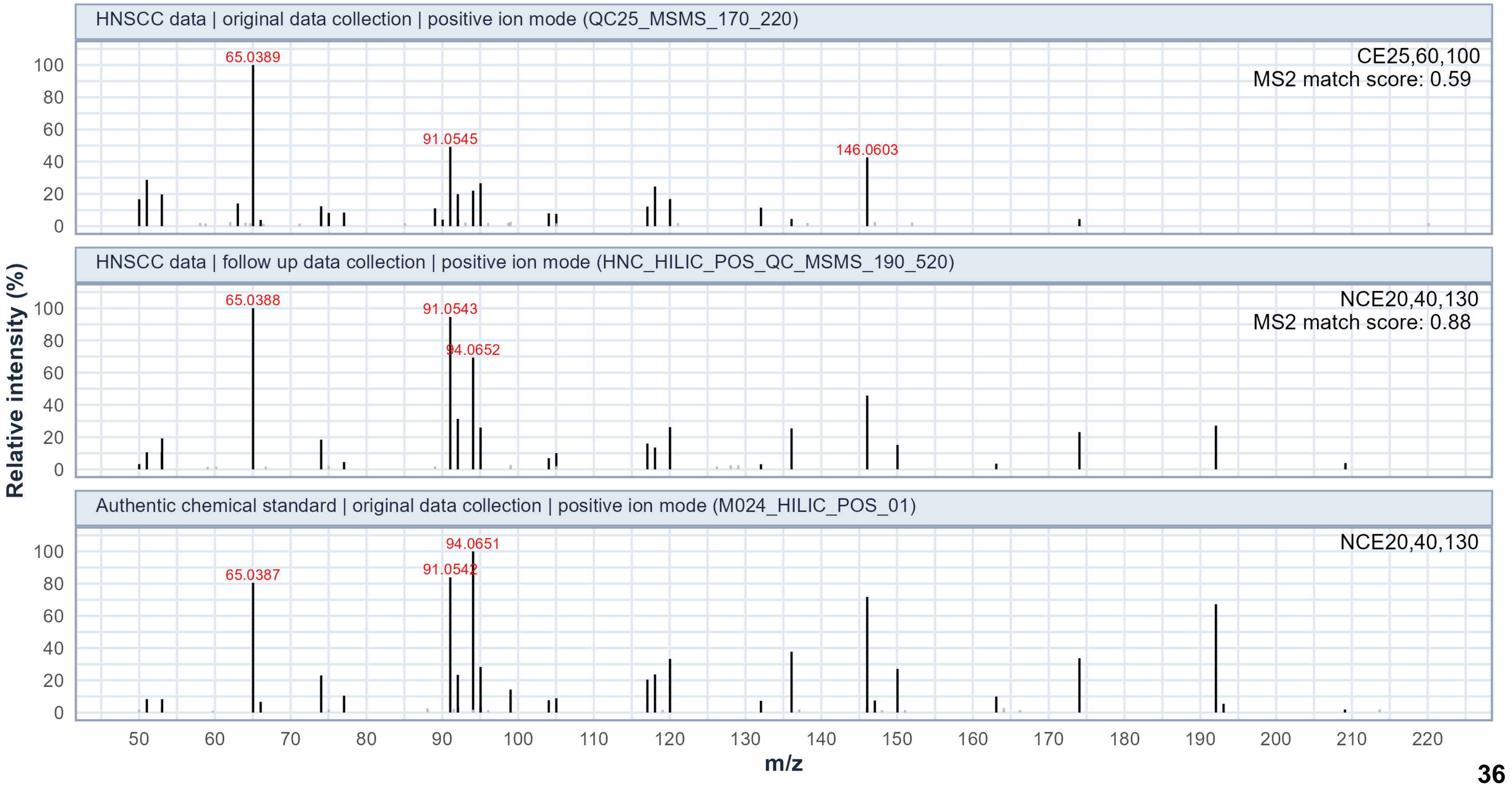

# L-Lysine [M+H]+ | HMDB0000182

Positive ion mode: 147.1128 m/z | Instrument: QE focus

## Chromatogram

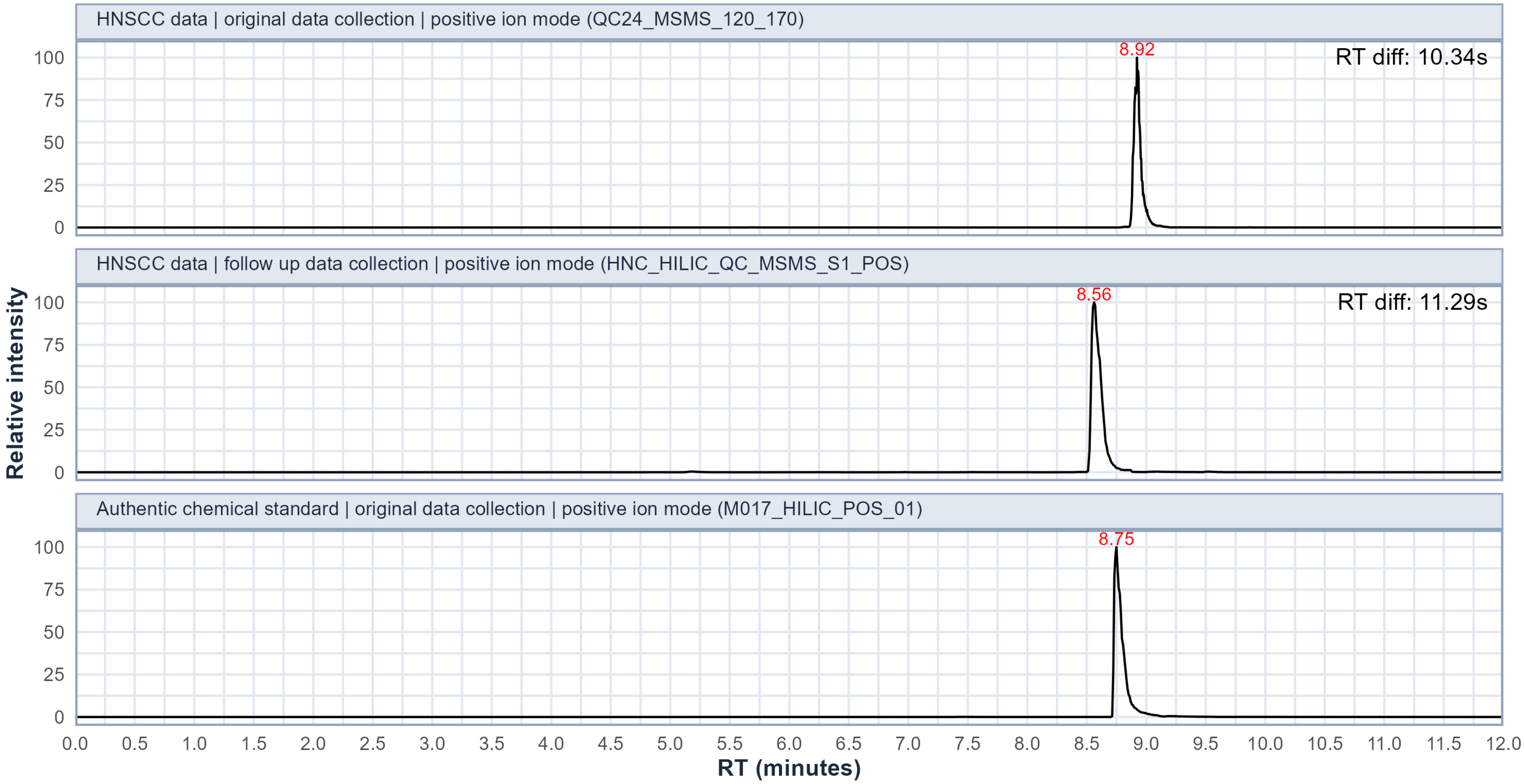


## MS/MS

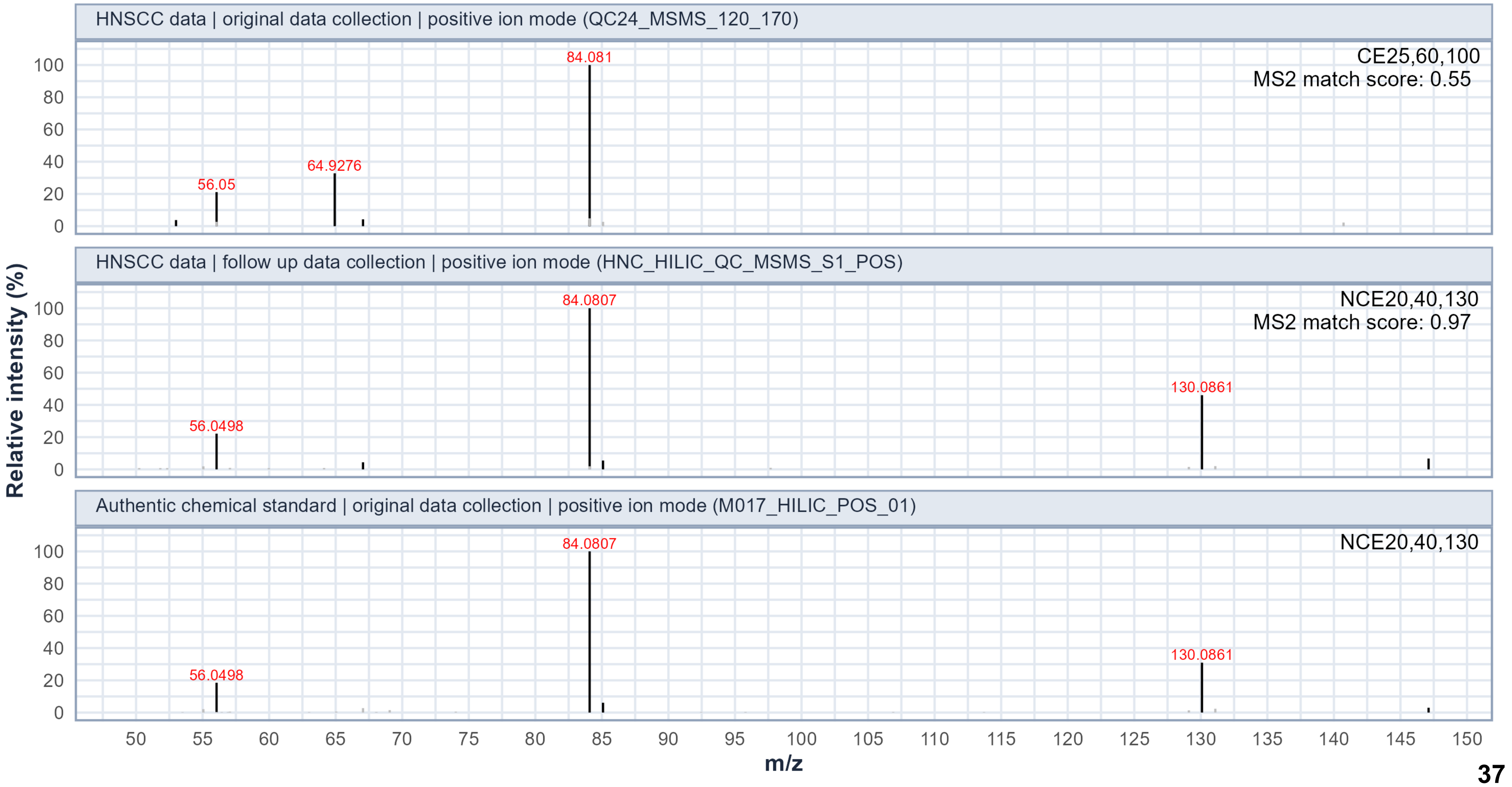

# L-Methionine [M+H]+ | HMDB0000696

Positive ion mode: 150.0583 m/z | Instrument: QE focus

## Chromatogram

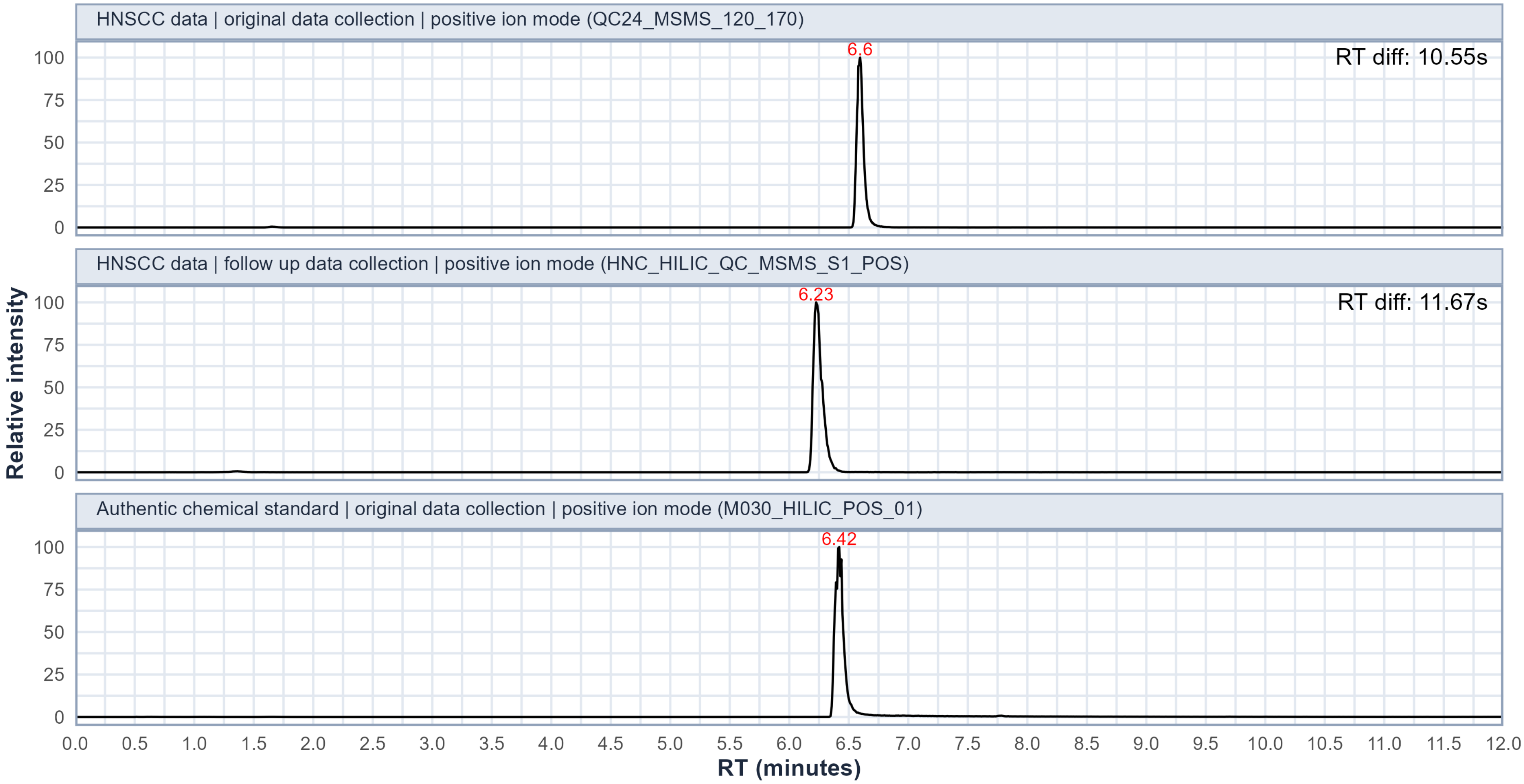


## MS/MS

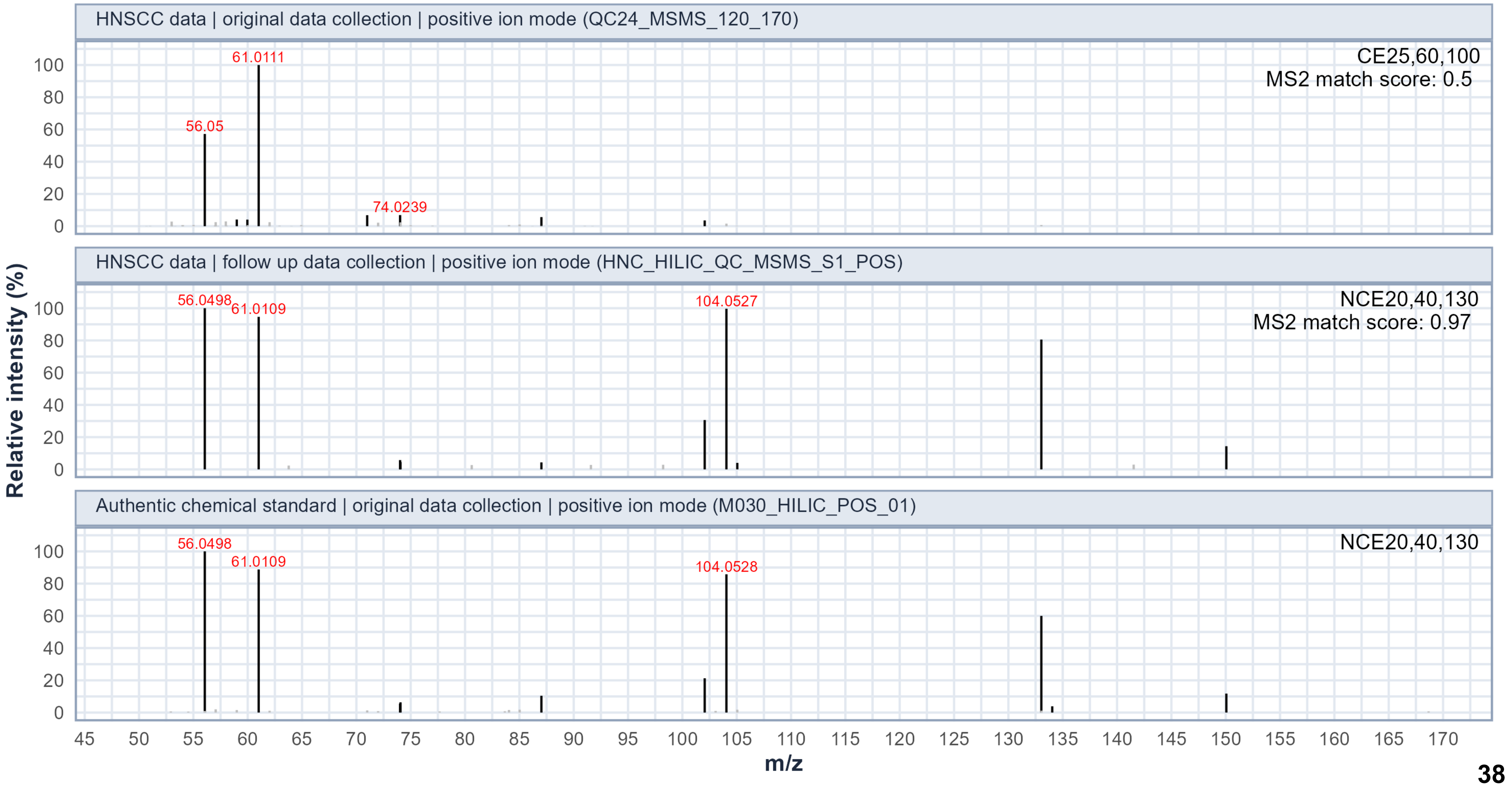

# L-Proline [M+H]+ | HMDB0000162

Positive ion mode: 116.0706 m/z | Instrument: QE focus

## Chromatogram

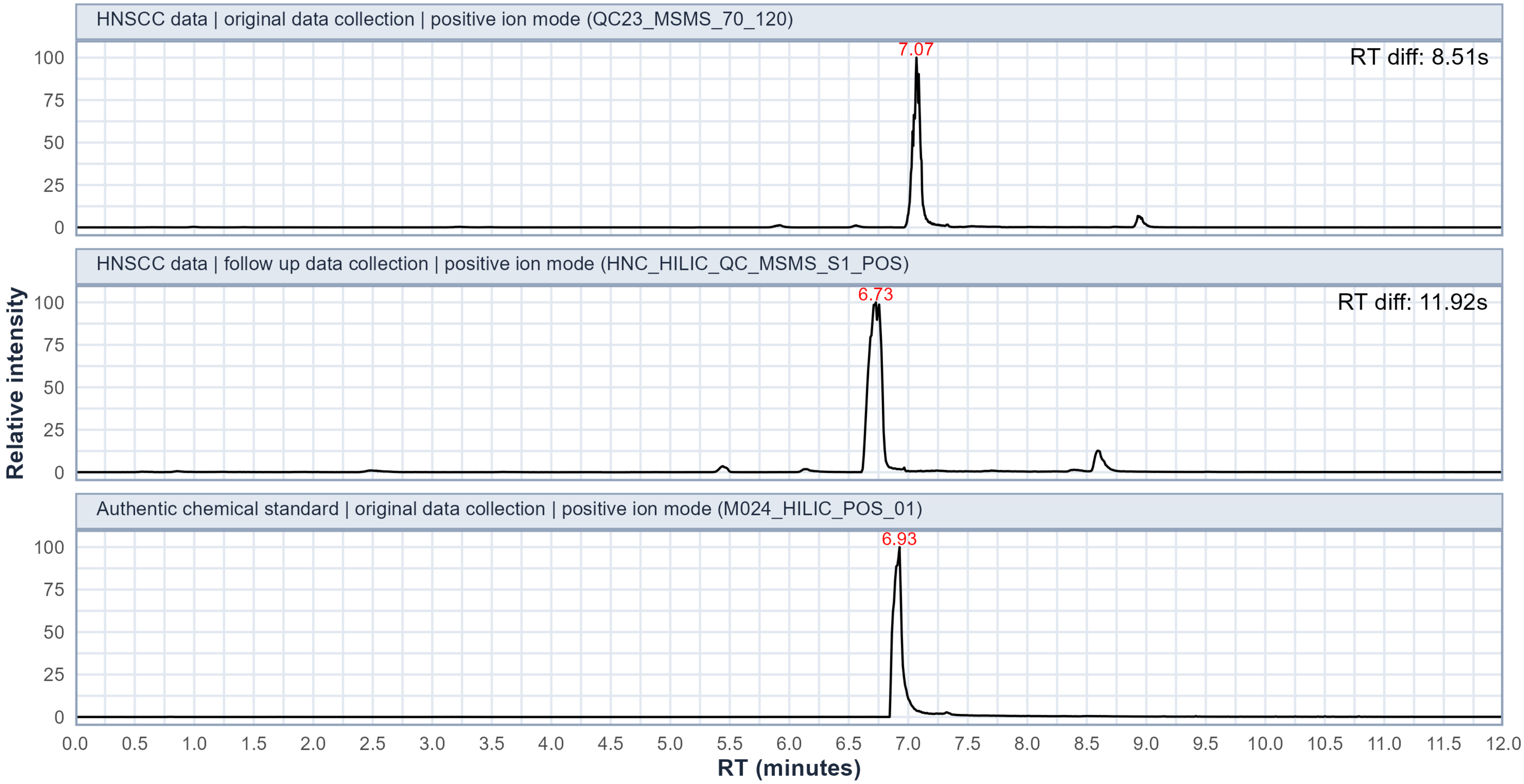


## MS/MS

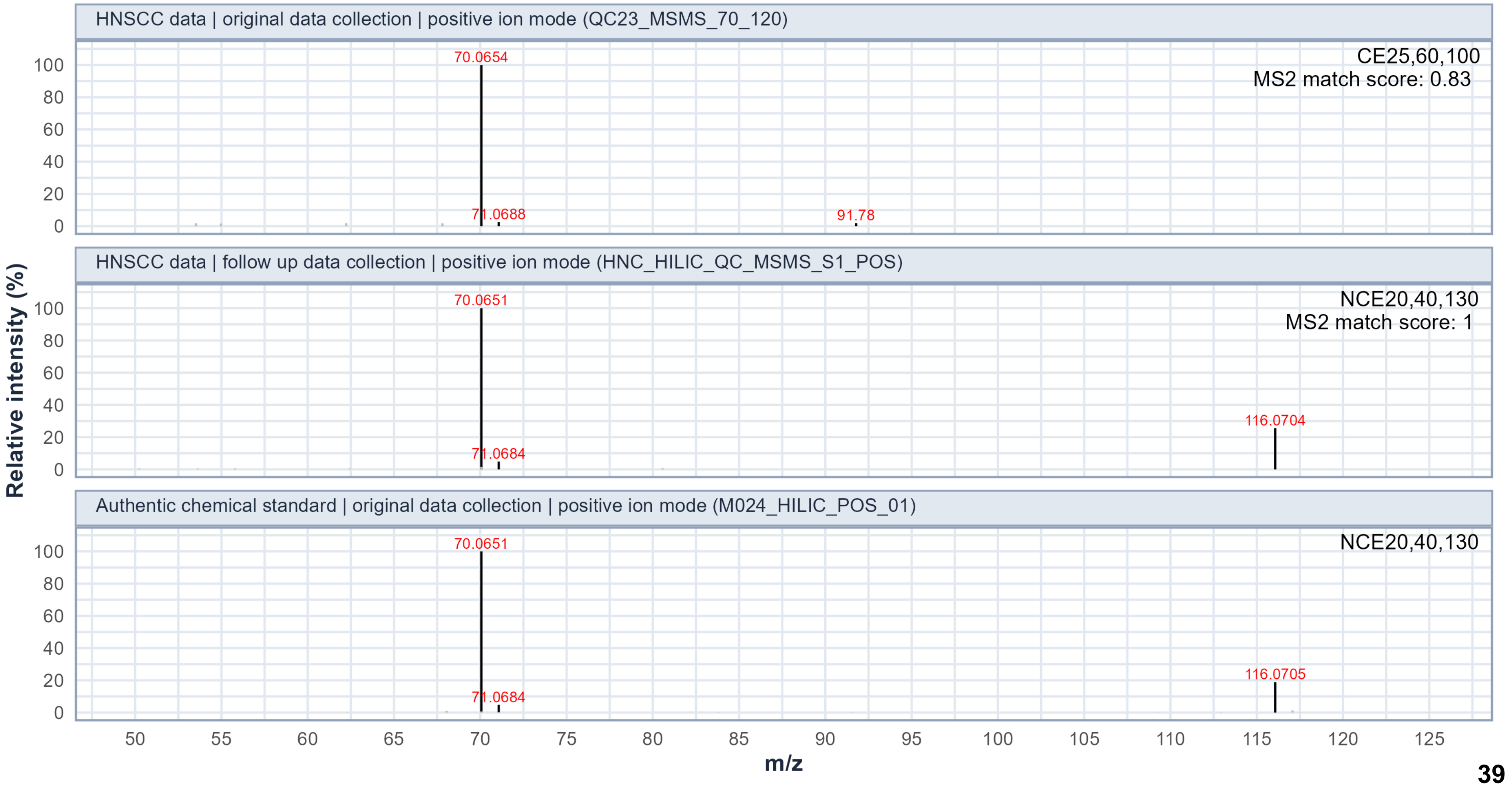

# L-Serine [M-H]- | HMDB0000187

Negative ion mode: 104.0353 m/z | Instrument: QE focus

## Chromatogram

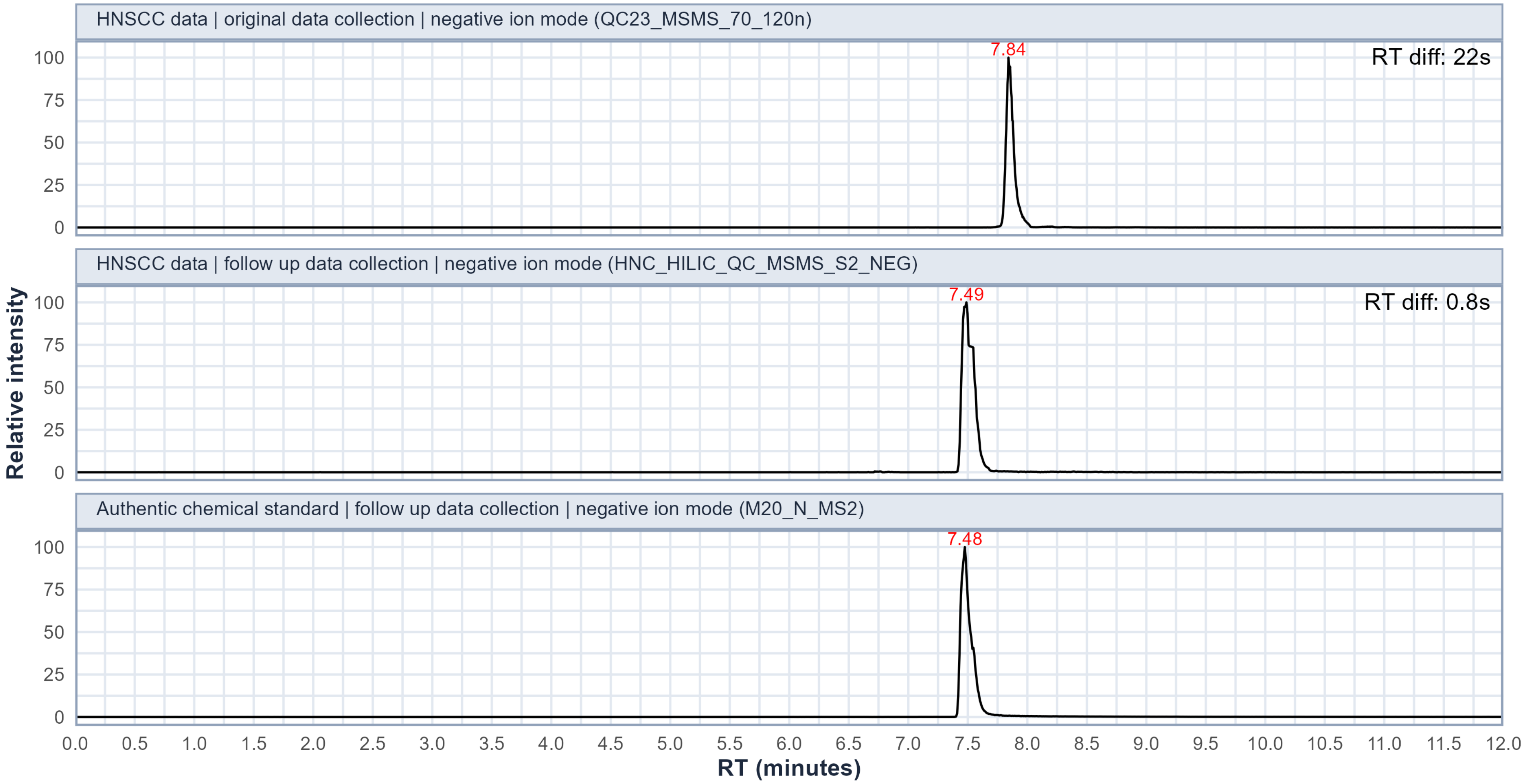


## MS/MS

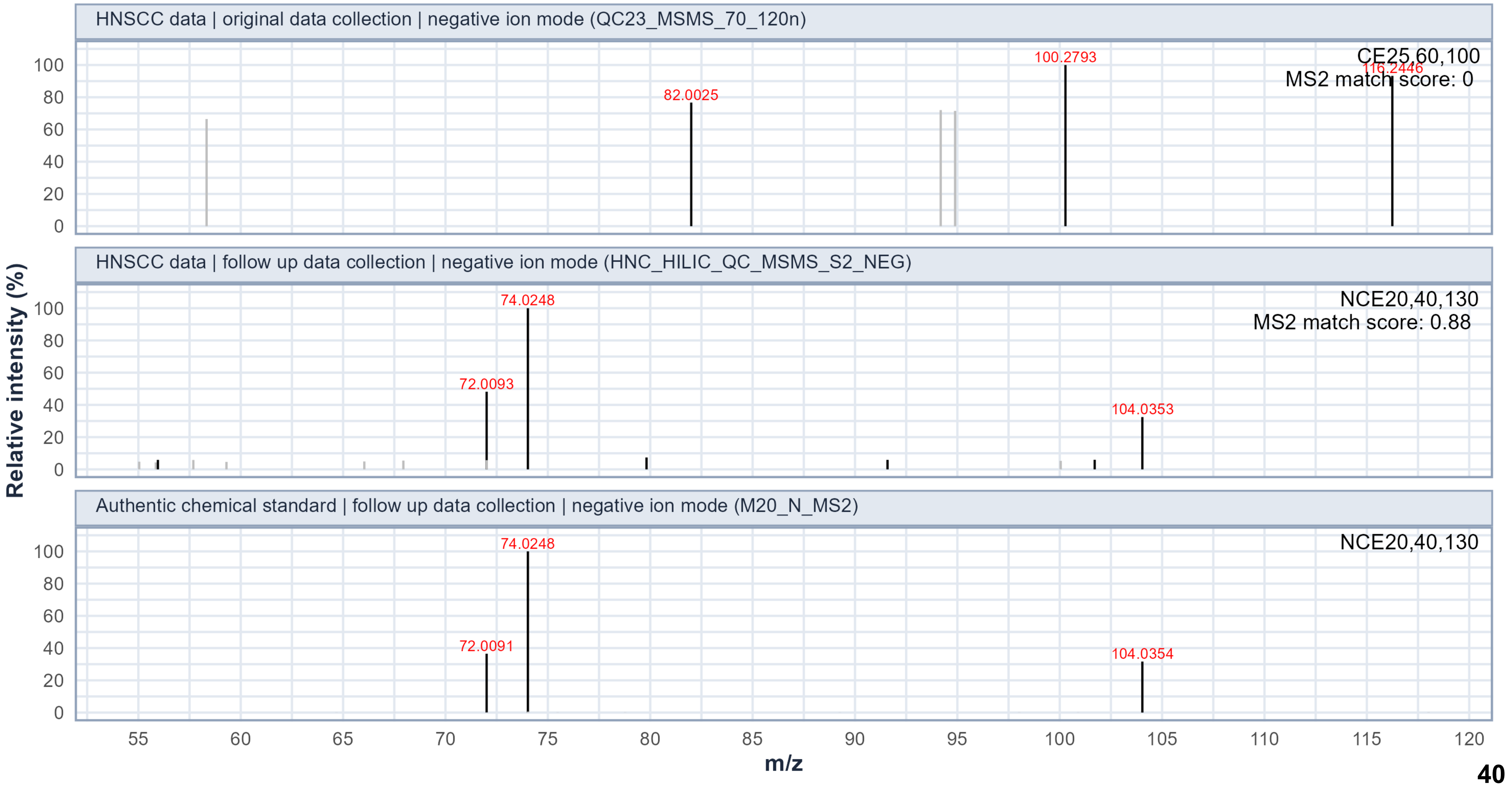

# L-Tryptophan [M+H]+ | HMDB0000929

Positive ion mode: 205.0972 m/z | Instrument: QE focus

## Chromatogram

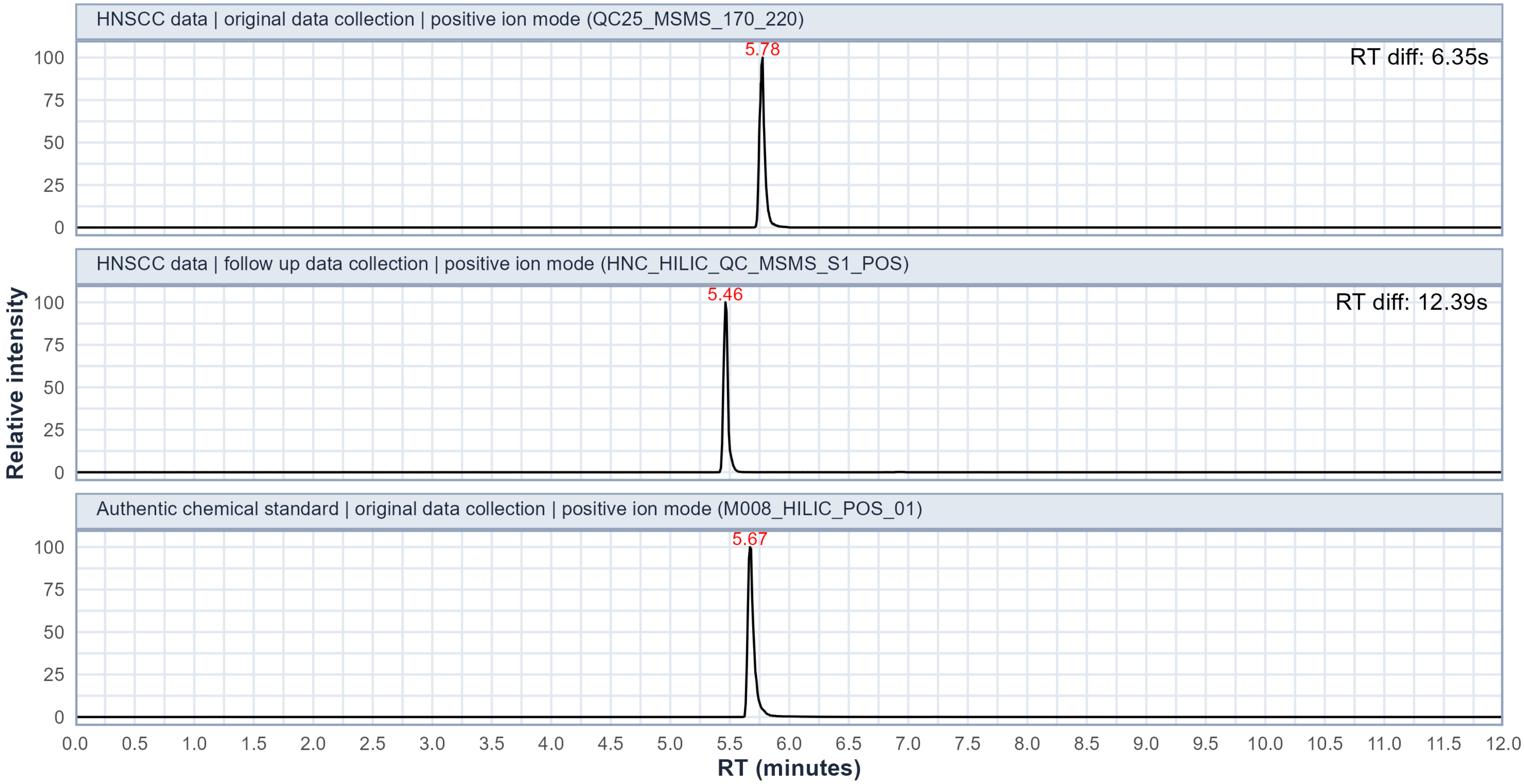


## MS/MS

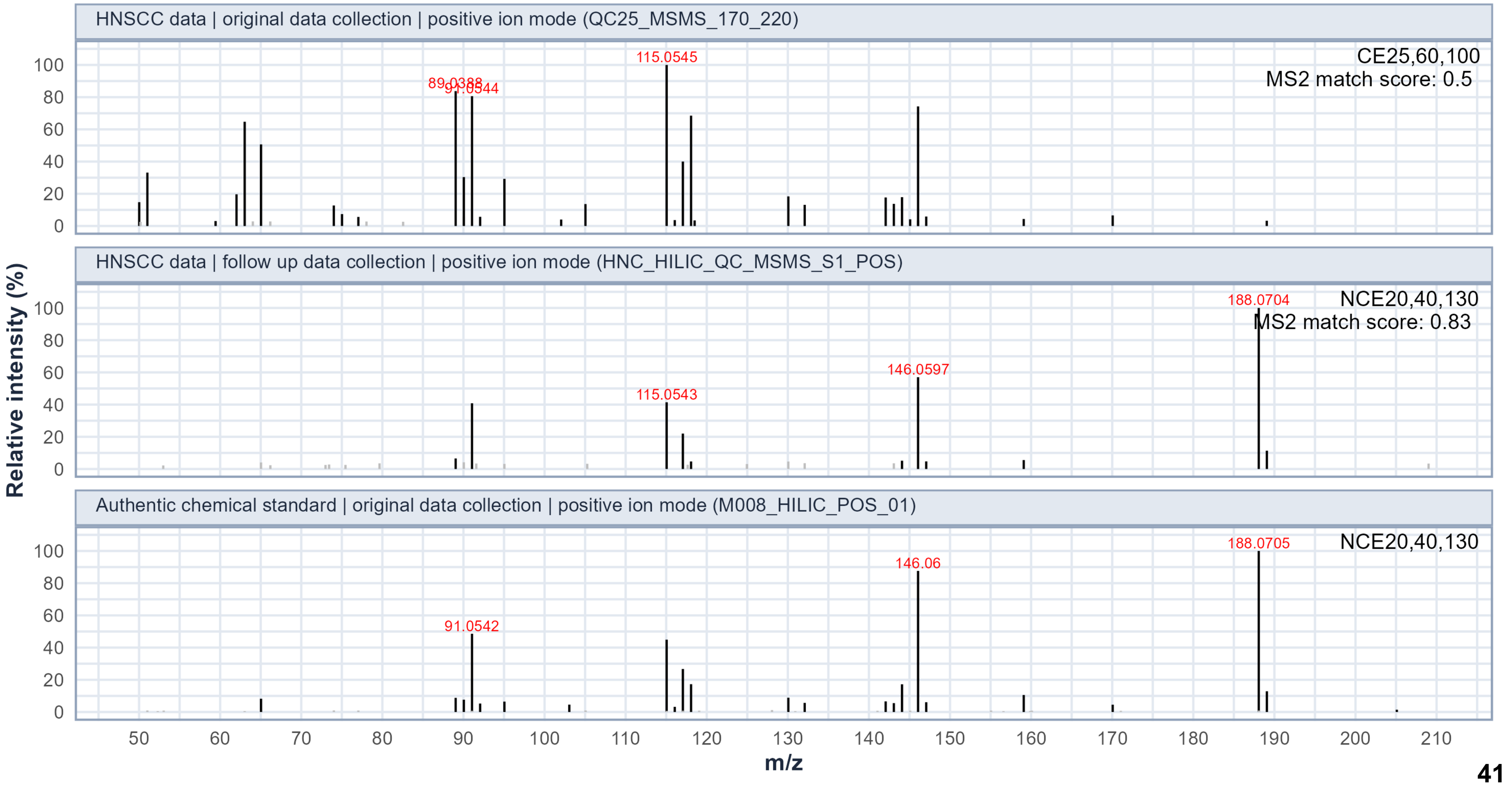

# Methylguanosine [M+H]+ (isoform #1) | HMDB0001563, HMDB0005862, HMDB0001107

Positive ion mode: 298.1146 m/z | Instrument: QE focus
Authentic chemical standard not available in library; identification based on MS/MS match to mzCloud.

## Chromatogram

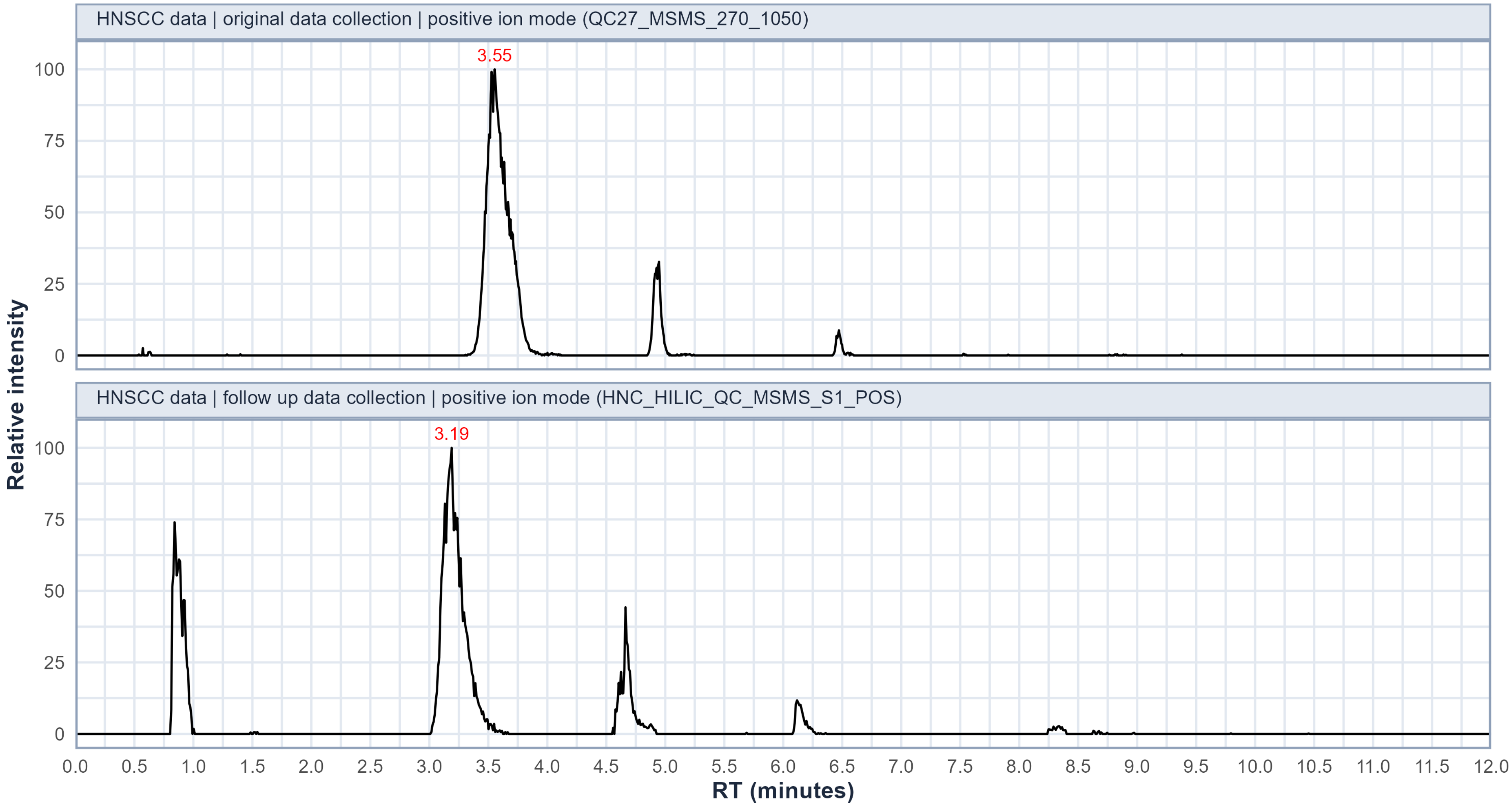


## MS/MS

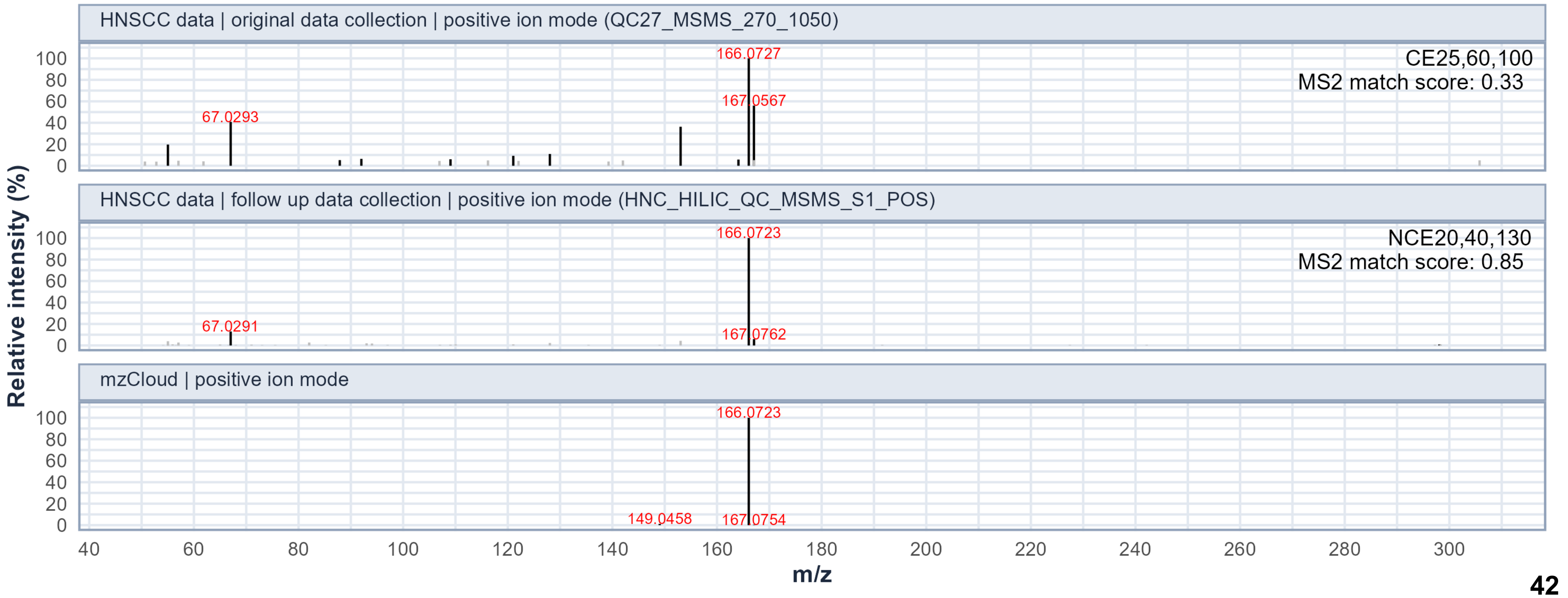

# Methylguanosine [M+H]+ (isoform #2) | HMDB0001563, HMDB0005862, HMDB0001107

Positive ion mode: 298.1146 m/z | Instrument: QE focus
Authentic chemical standard not available in library; identification based on MS/MS match to mzCloud.

## Chromatogram

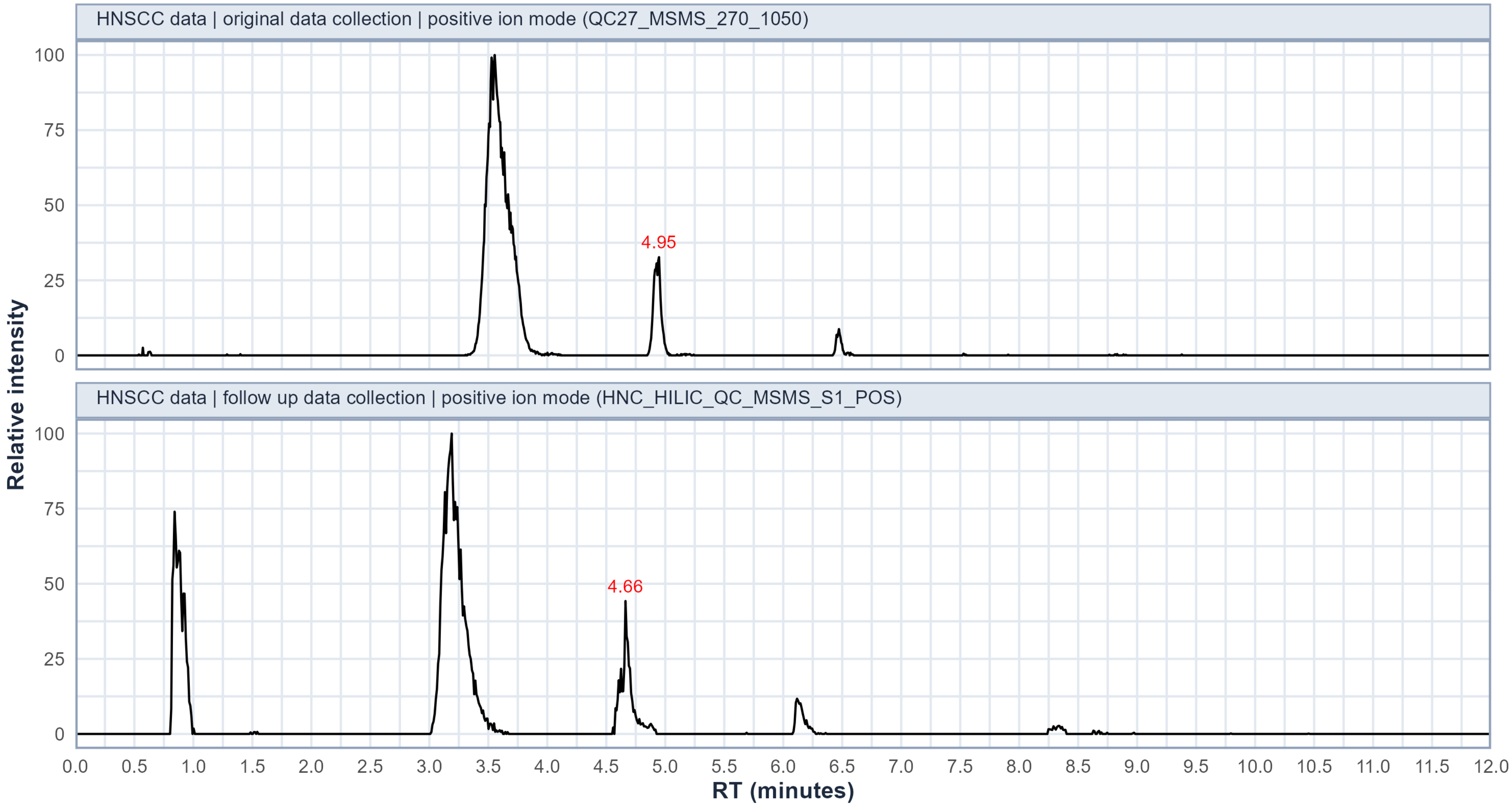


## MS/MS

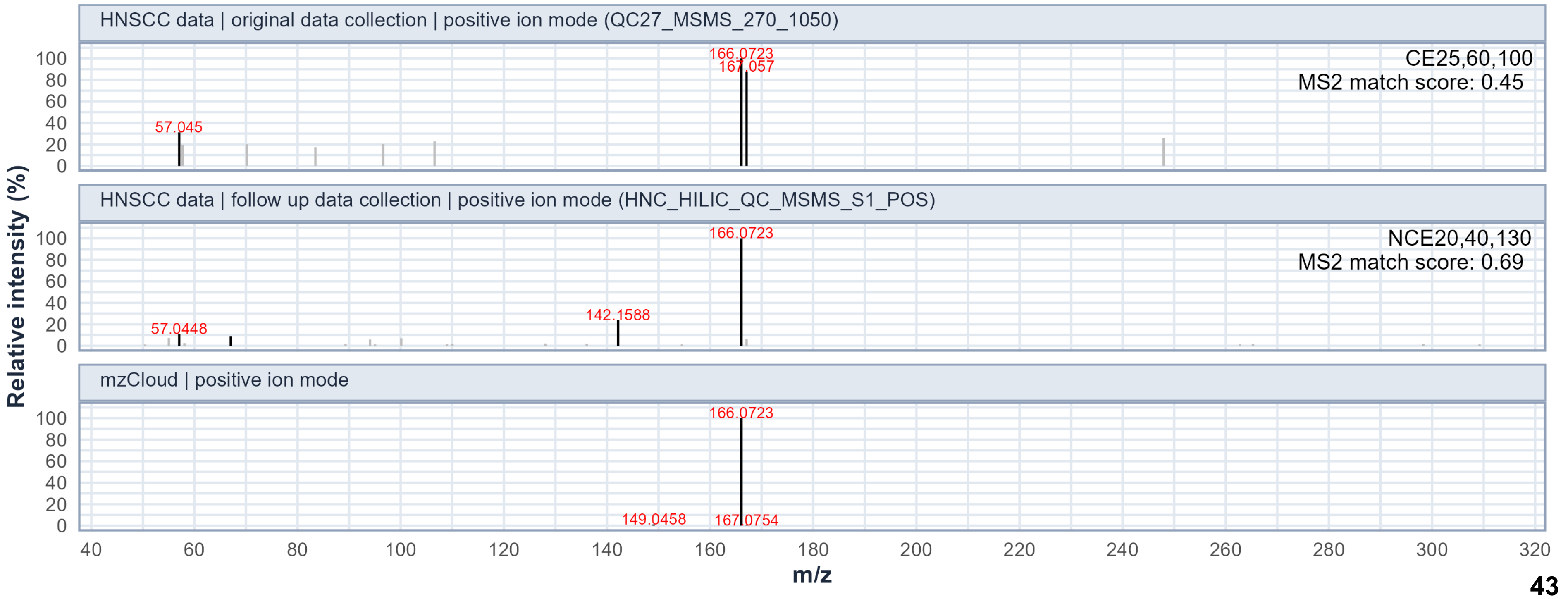

# Methylhistamine [M+H]+ | HMDB0000898, HMDB0001861

Positive ion mode: 126.1026 m/z | Instrument: QE focus
Authentic chemical standard not available in library; identification based on MS/MS match to mzCloud.

## Chromatogram

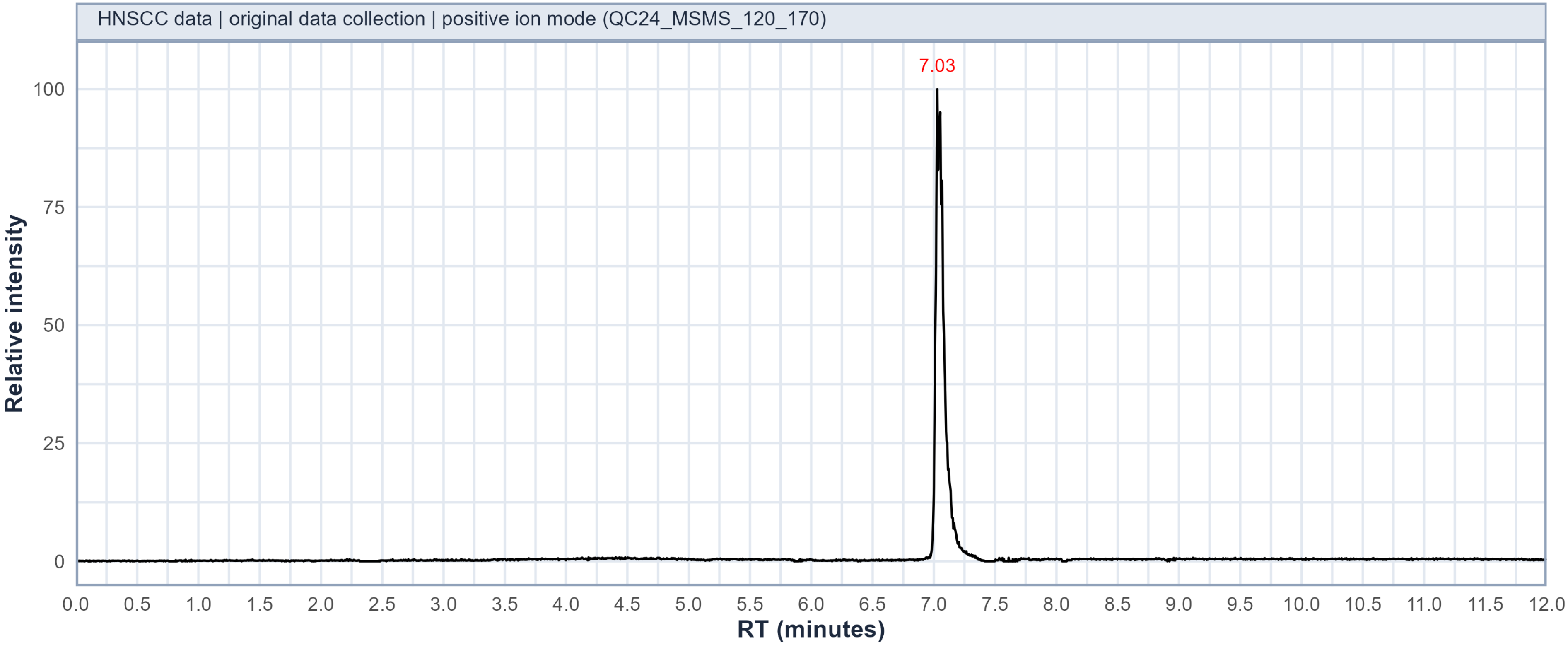


## MS/MS

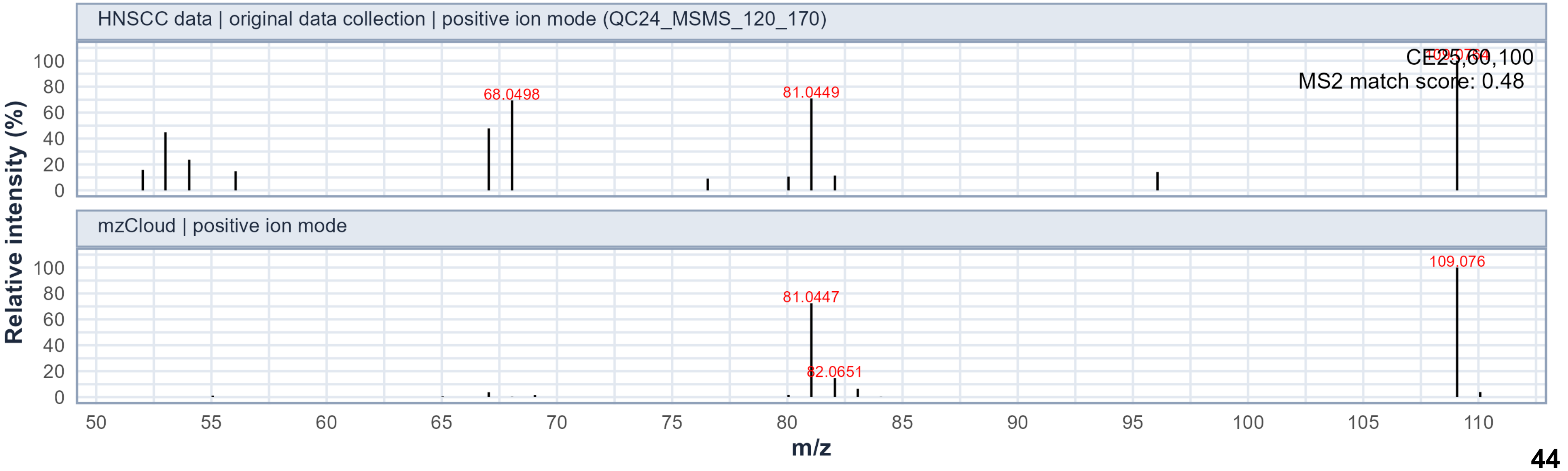

# N1-Acetylspermidine [M+H]+ | HMDB0001276

Positive ion mode: 188.1757 m/z | Instrument: QE focus

## Chromatogram

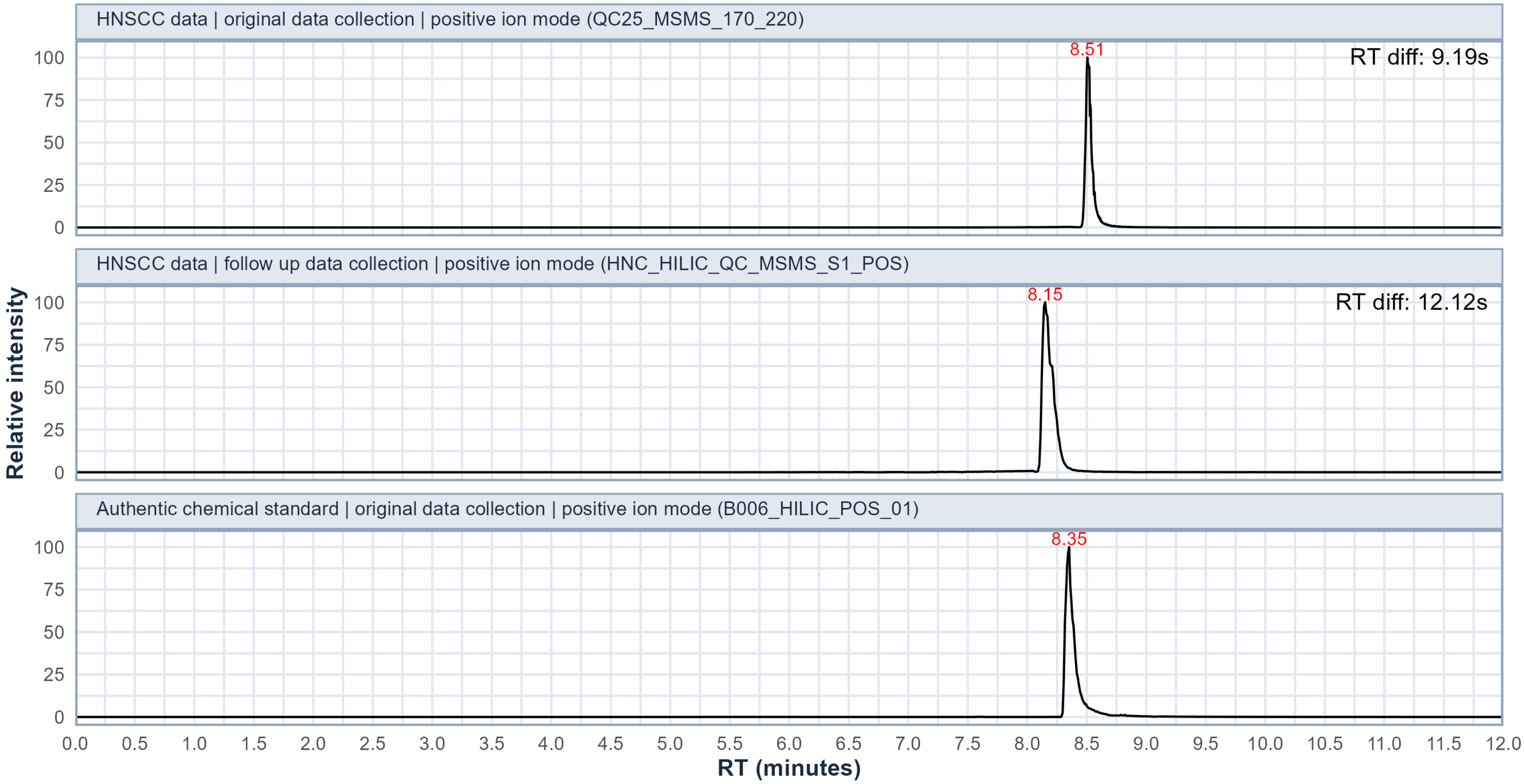


## MS/MS

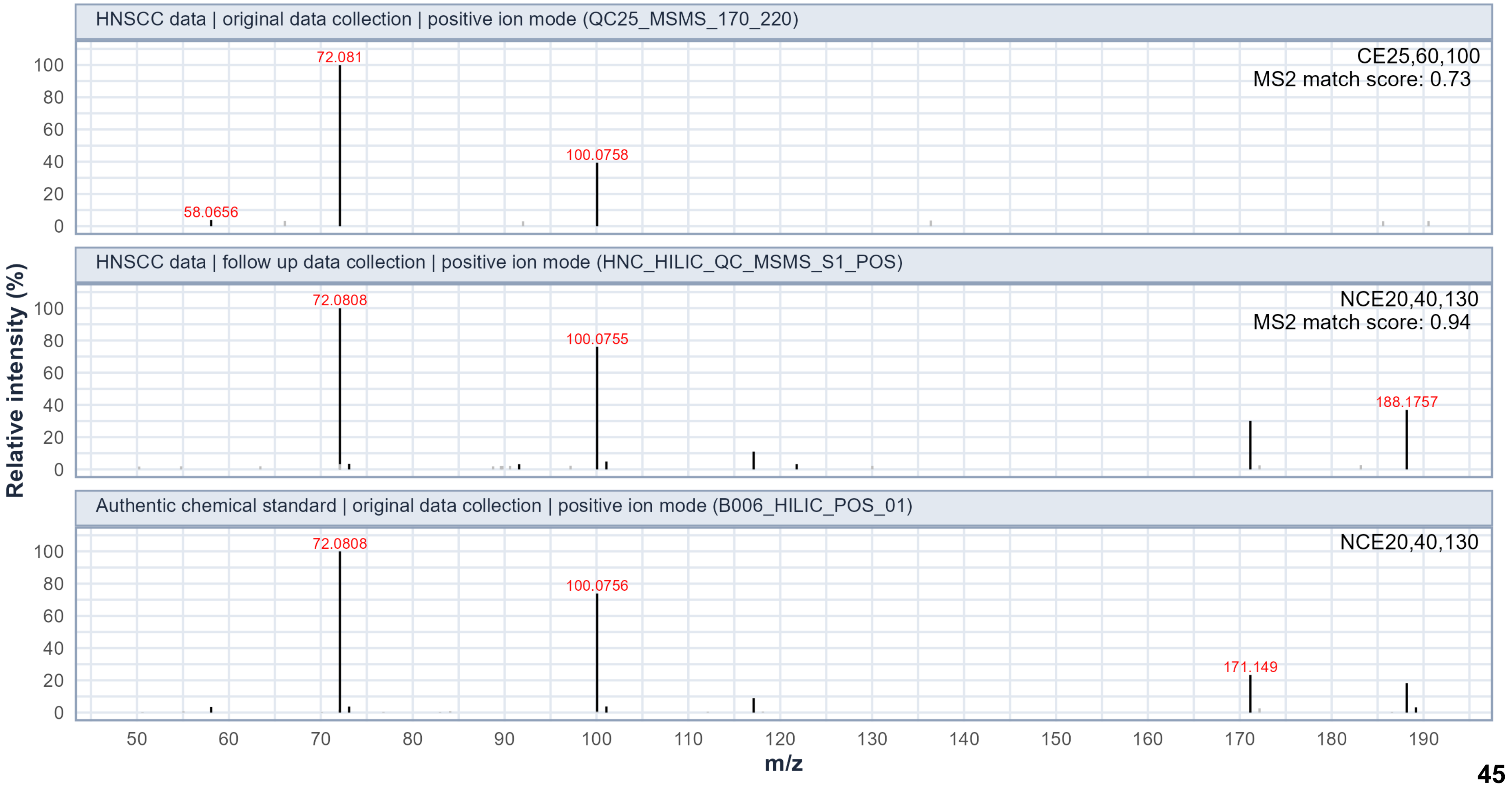

# N6,N6,N6-Trimethyl-L-lysine [M+H]+ | HMDB0001325

Positive ion mode: 189.1598 m/z | Instrument: QE focus
Authentic chemical standard not available in library; identification based on MS/MS match to mzCloud.

## Chromatogram

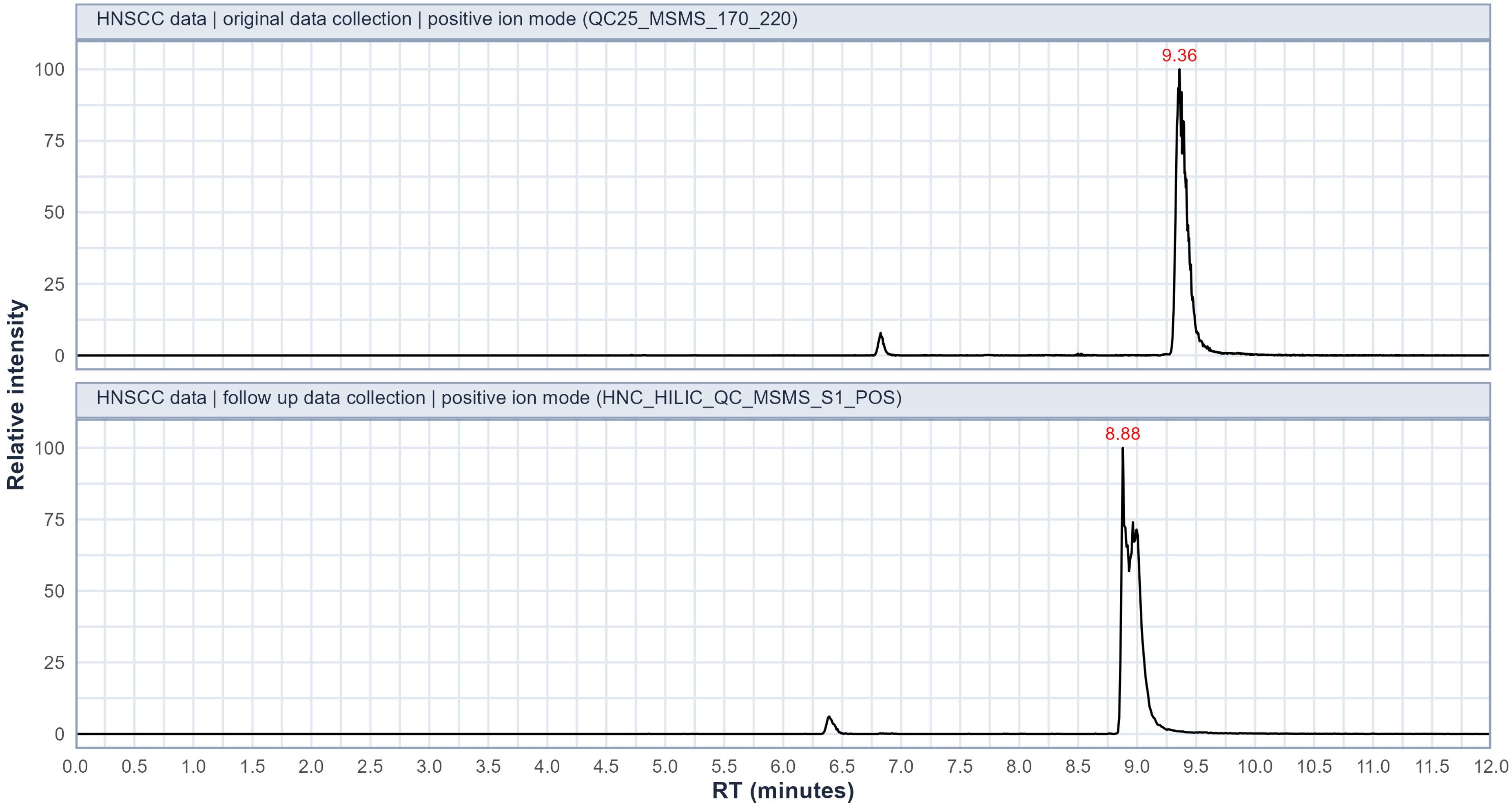


## MS/MS

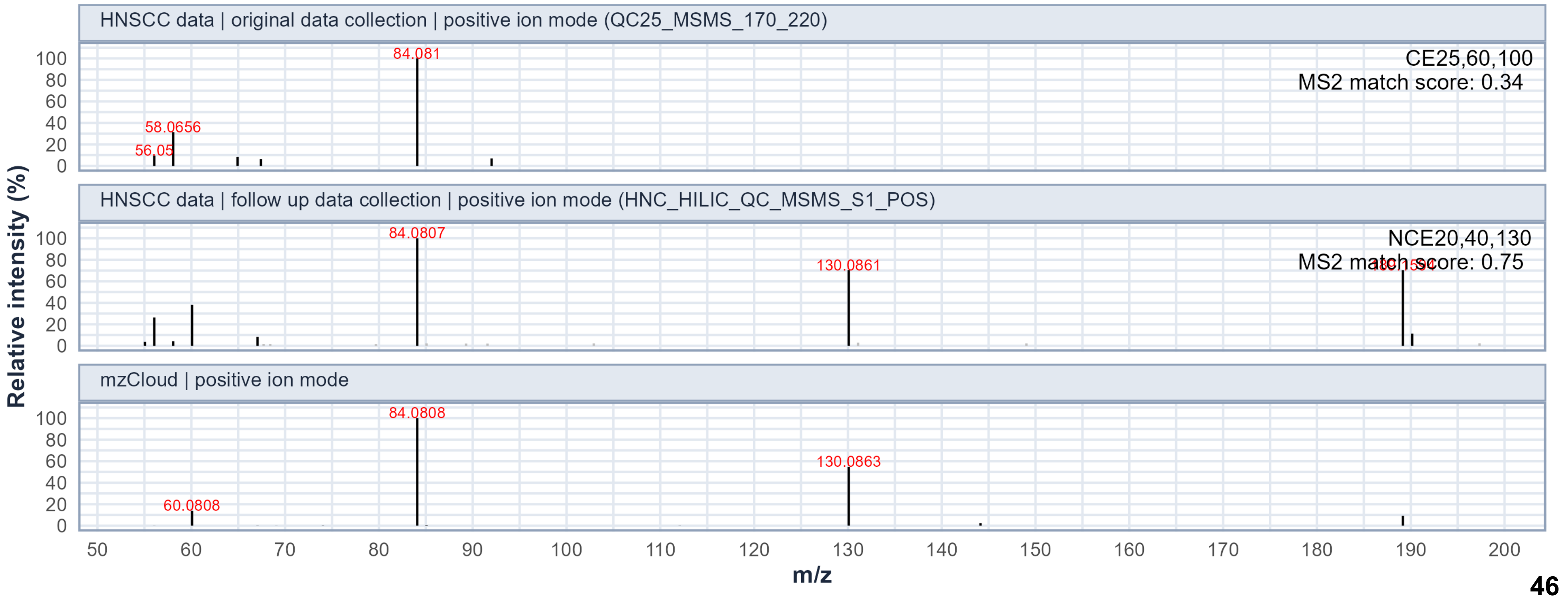

# N6-methyladenosine [M+H]+ | HMDB0004044

Positive ion mode: 282.1197 m/z | Instrument: QE focus

## Chromatogram

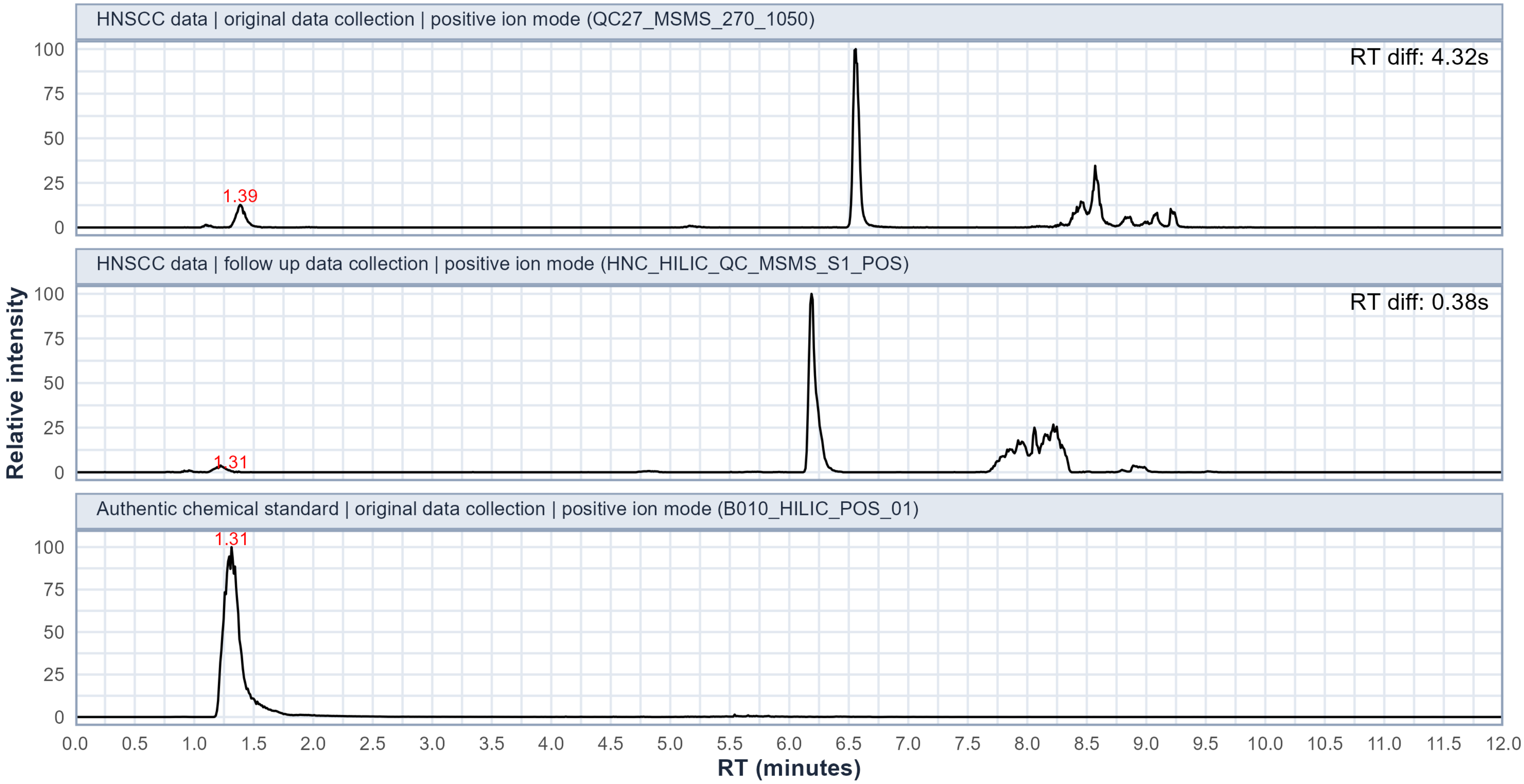


## MS/MS

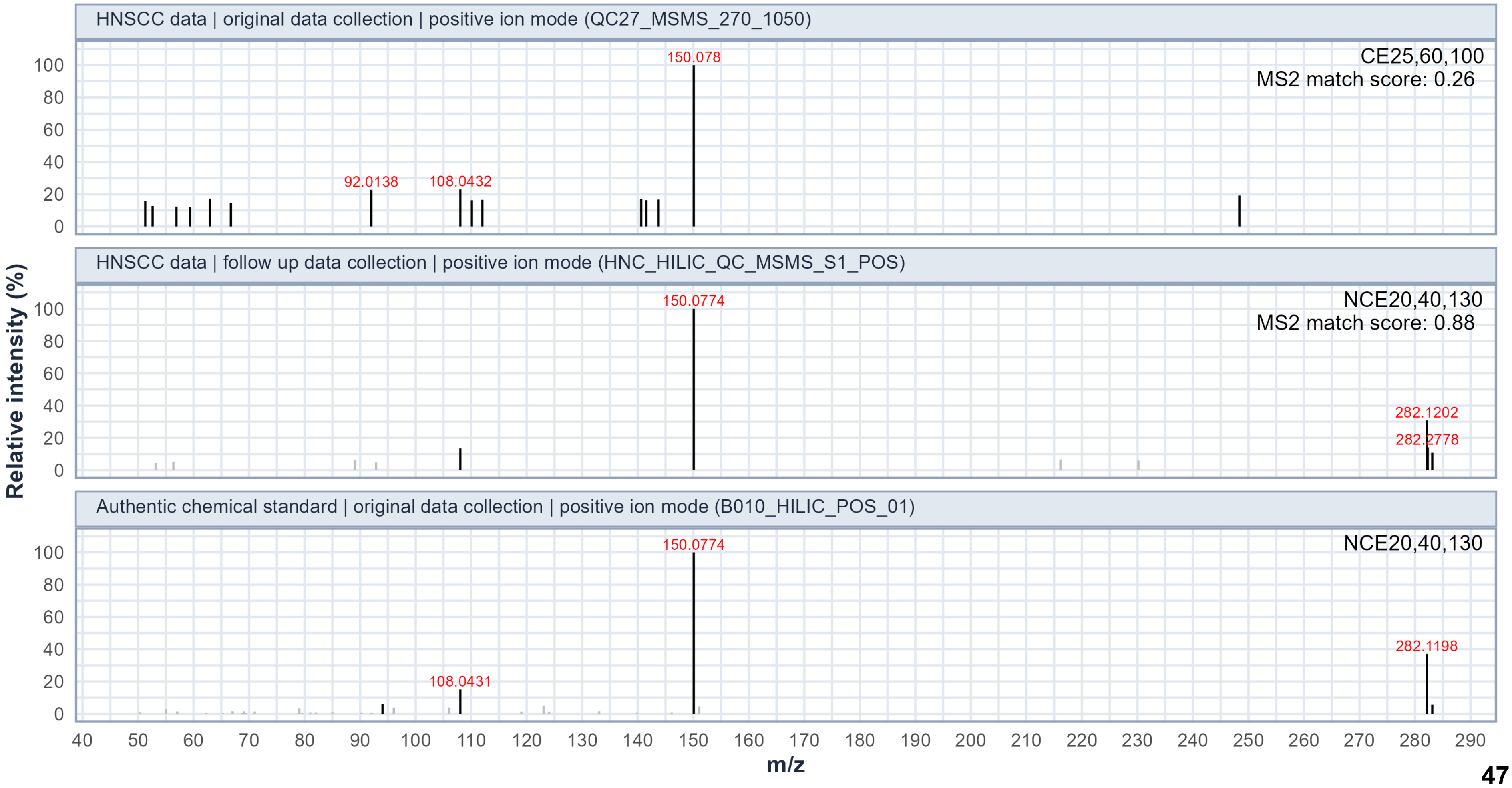

# N-Acetyl-L-carnosine [M+H]+ | HMDB0012881

Positive ion mode: 269.1244 m/z | Instrument: QE focus
Authentic chemical standard not available in library; identification based on MS/MS match to mzCloud.

## Chromatogram

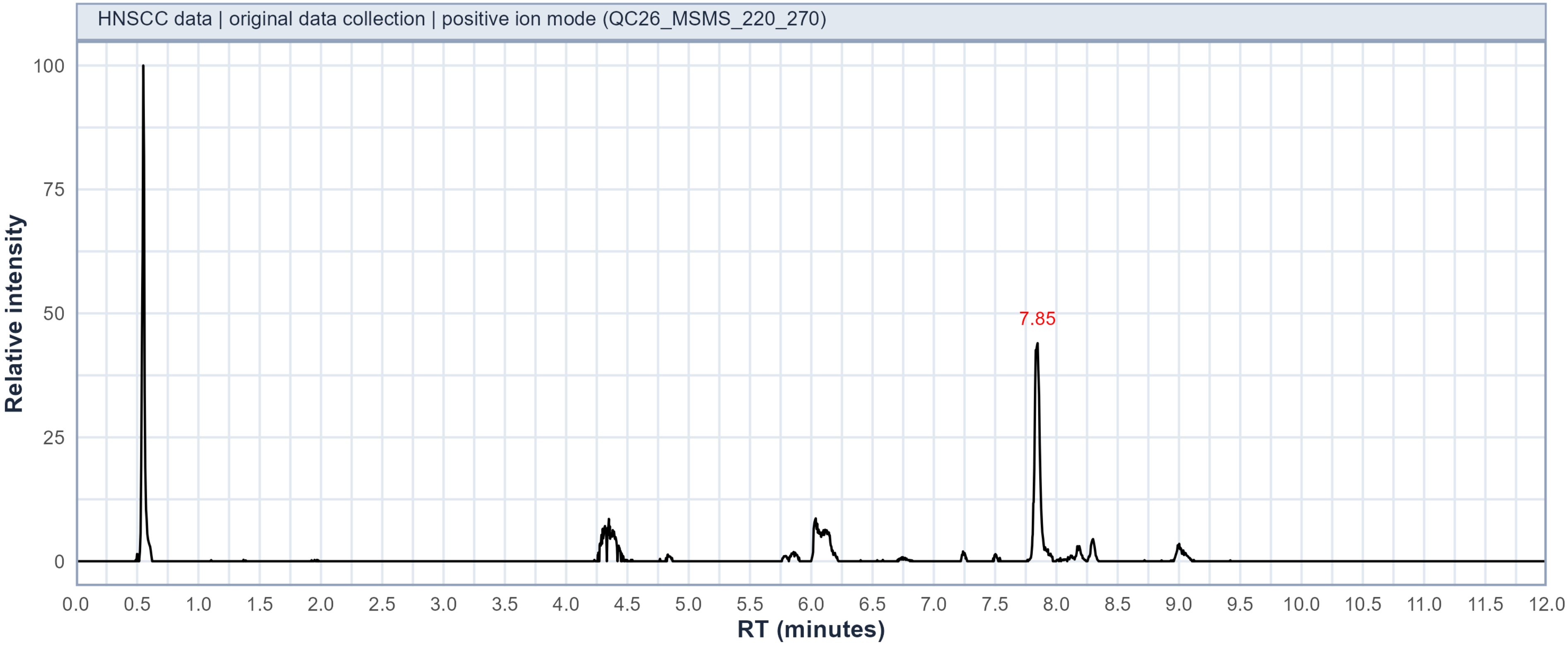


## MS/MS

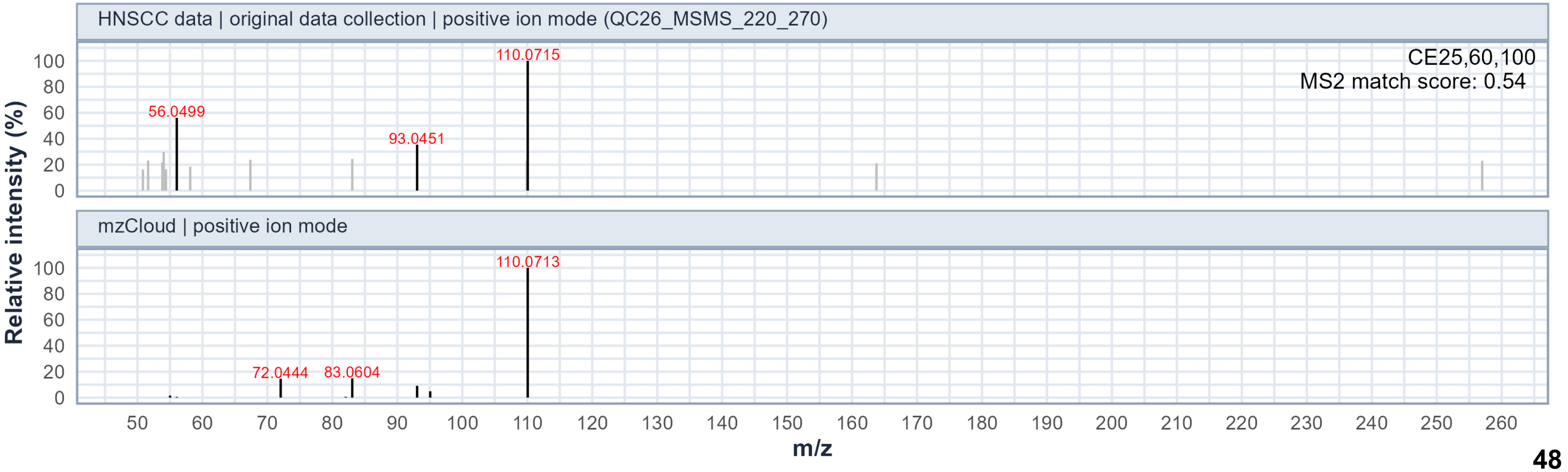

# Niacinamide [M+H]+ | HMDB0001406

Positive ion mode: 123.0553 m/z | Instrument: QE focus

## Chromatogram

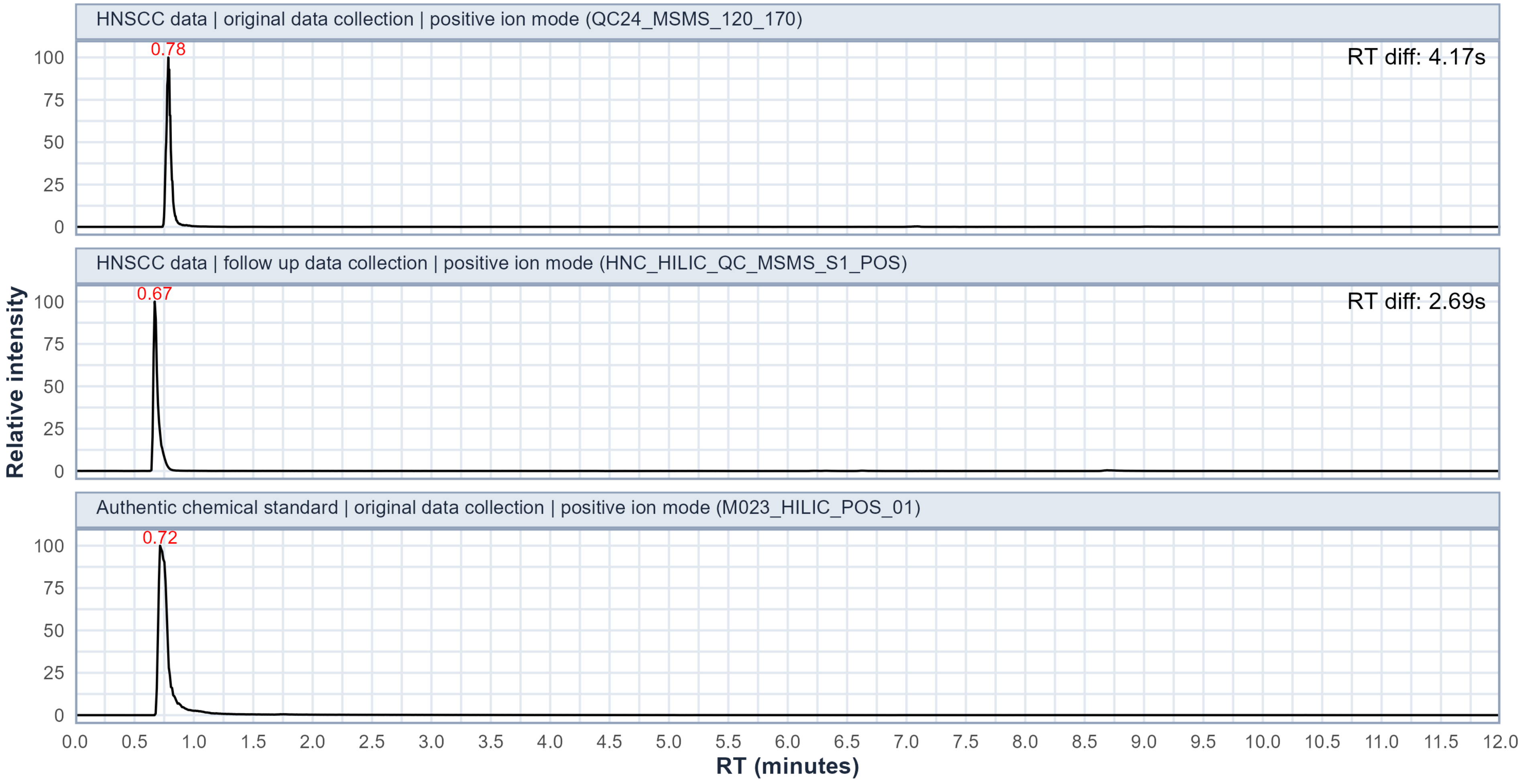


## MS/MS

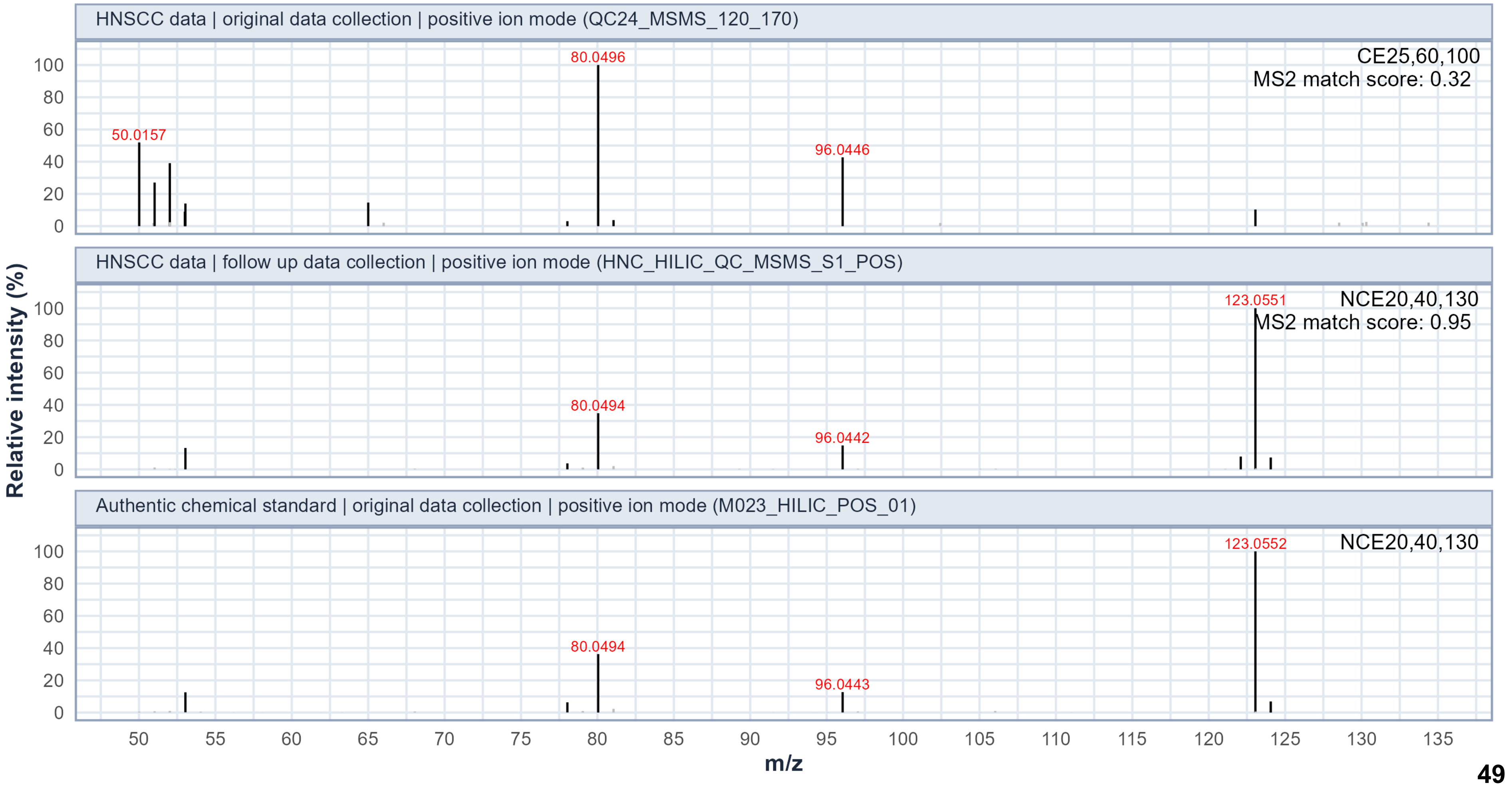

# Ornithine [M+H]+ | HMDB0000214

Positive ion mode: 133.0972 m/z | Instrument: QE focus

## Chromatogram

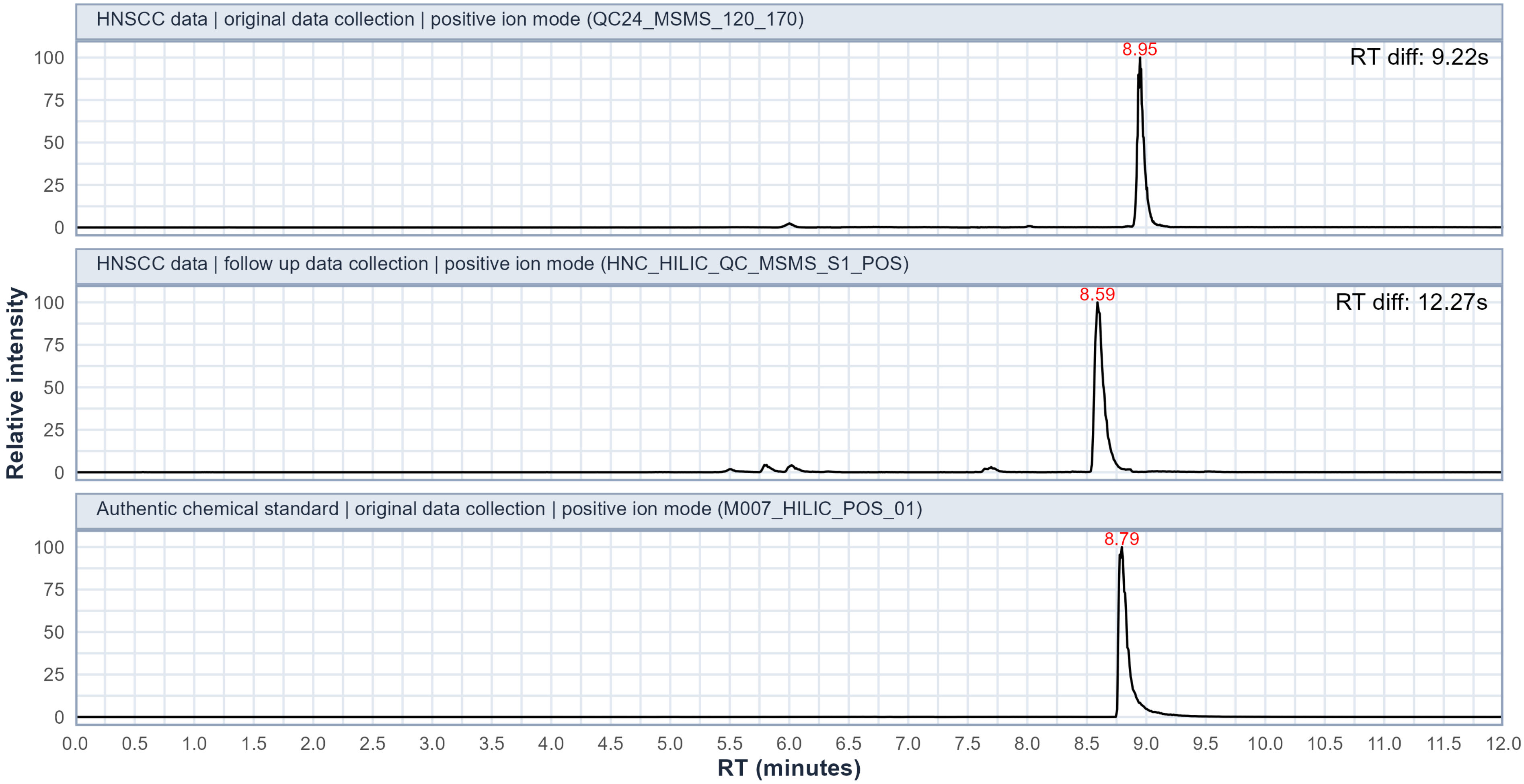


## MS/MS

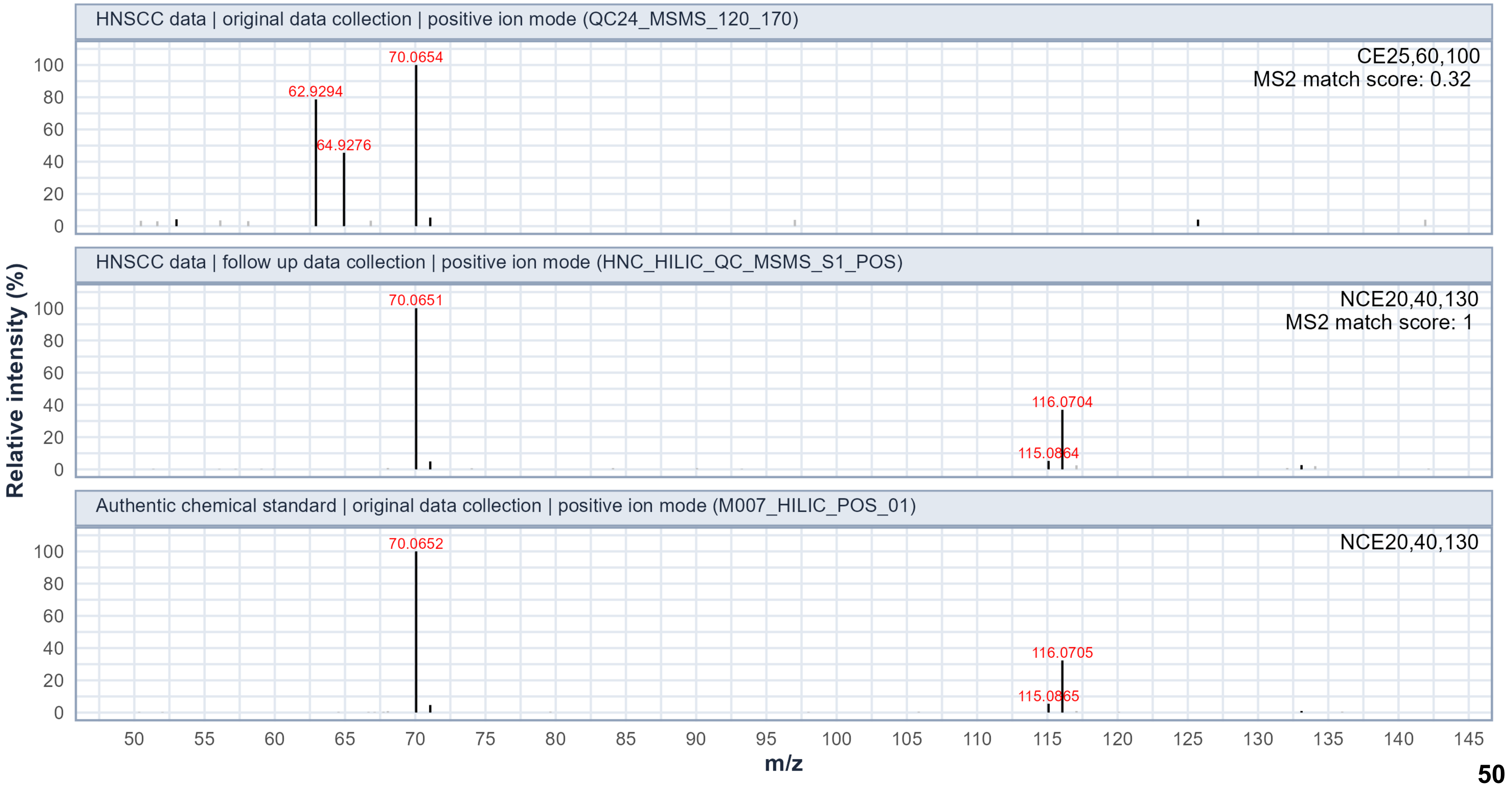

# Oxidised glutathione (GSSG) [M+H]+ | HMDB0003337

Positive ion mode: 613.1592 m/z | Instrument: QE focus

## Chromatogram

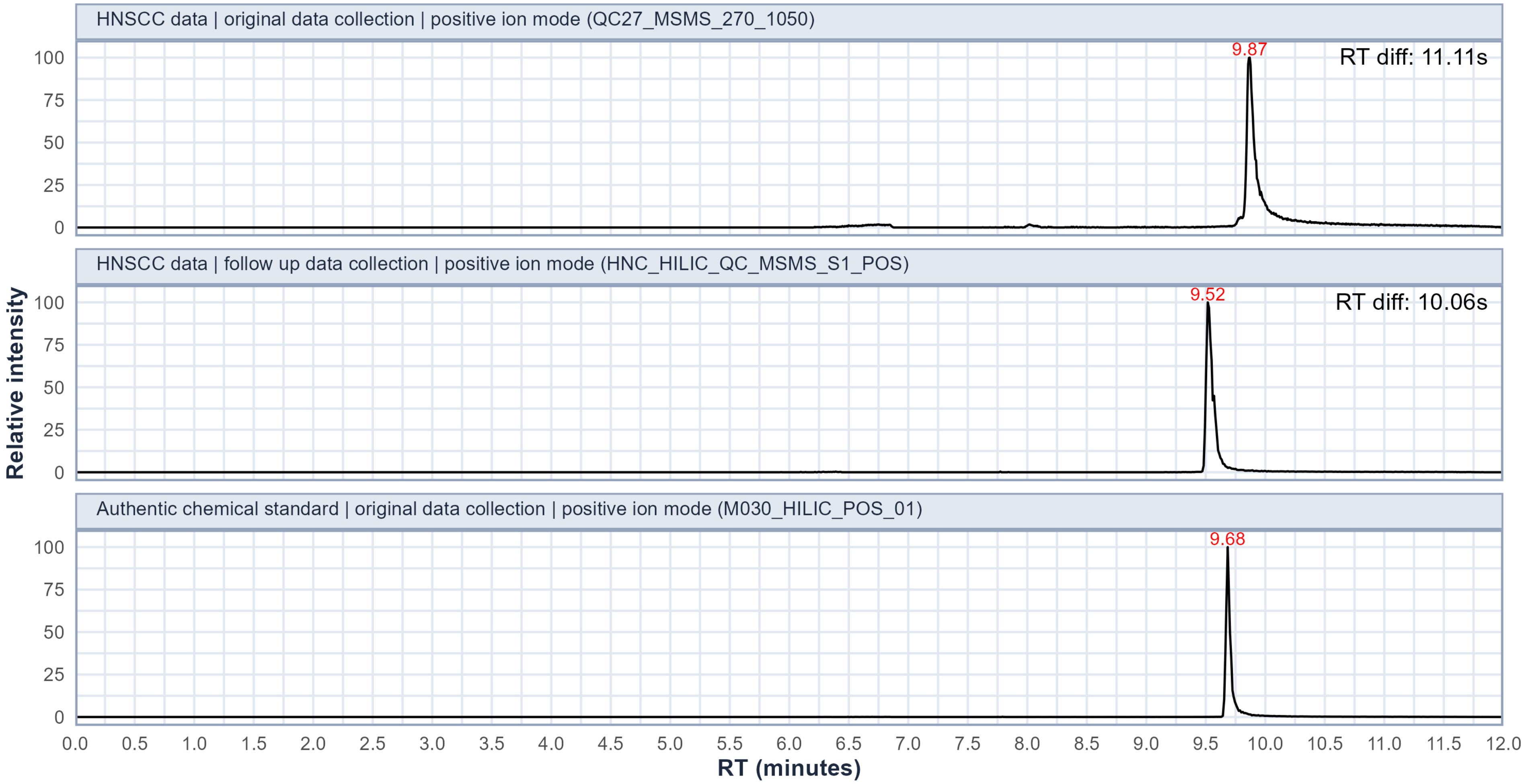


## MS/MS

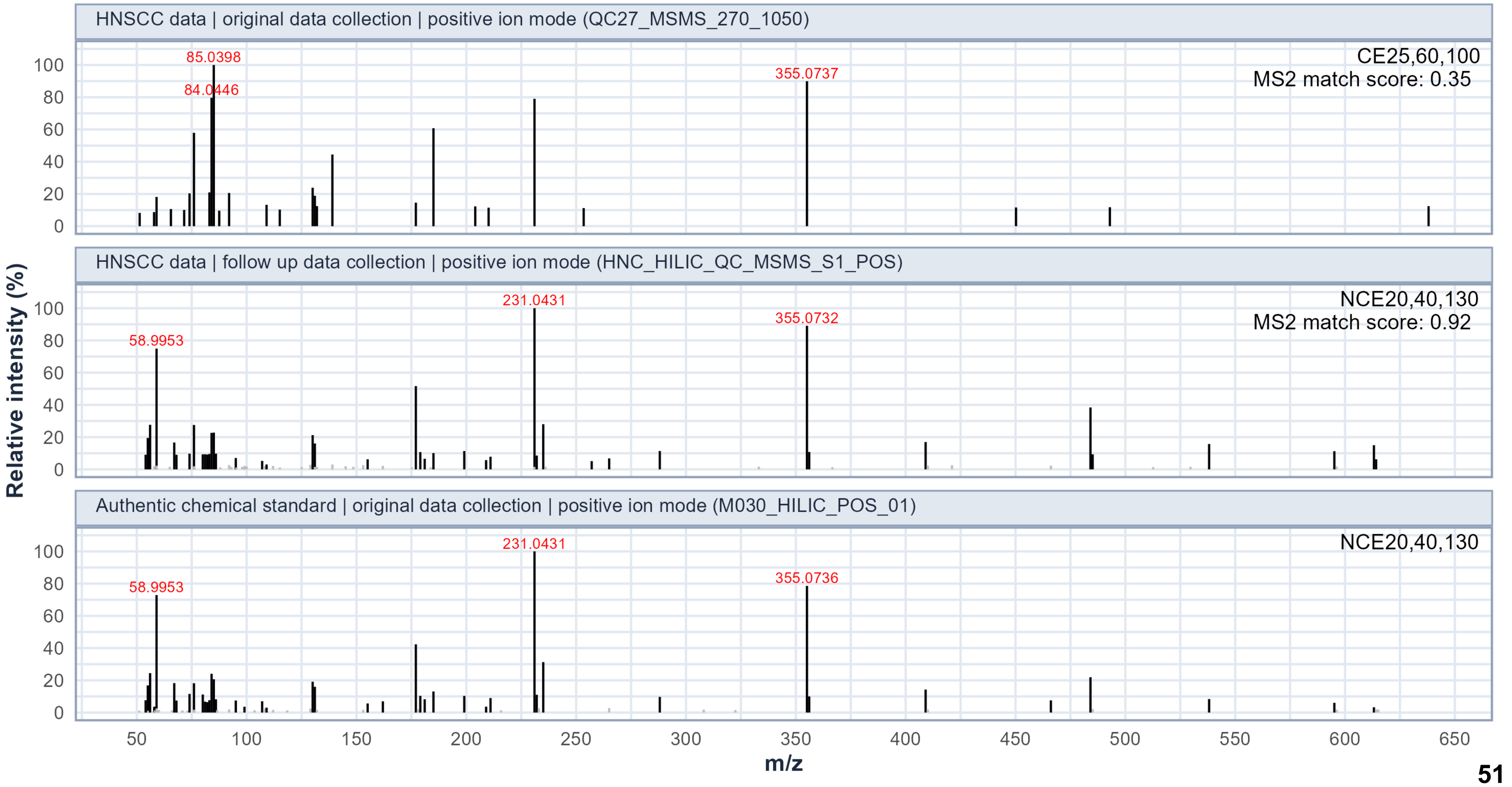

# Phosphocreatine [M+H]+ | HMDB0001511

Positive ion mode: 212.0431 m/z | Instrument: QE focus

## Chromatogram

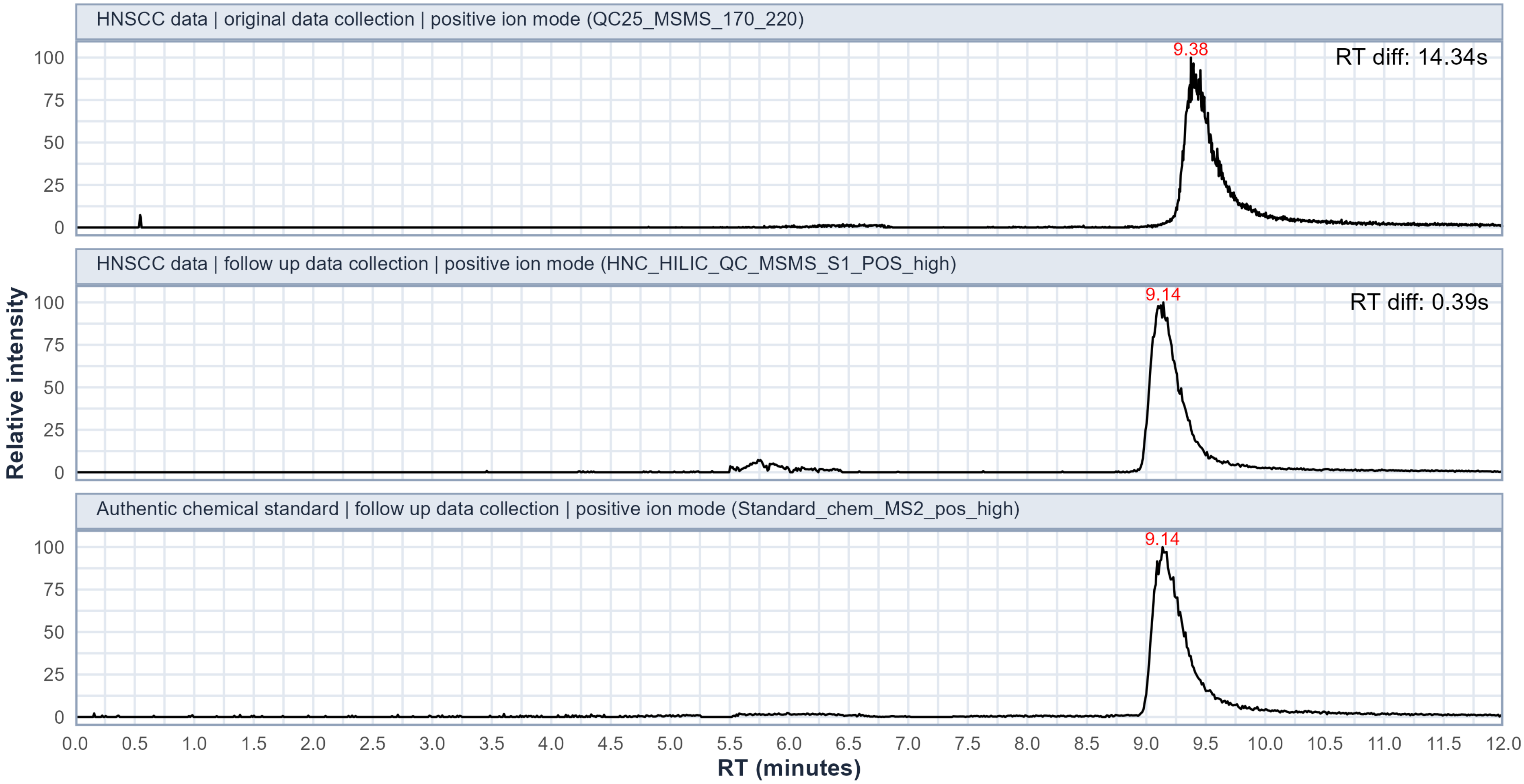


## MS/MS

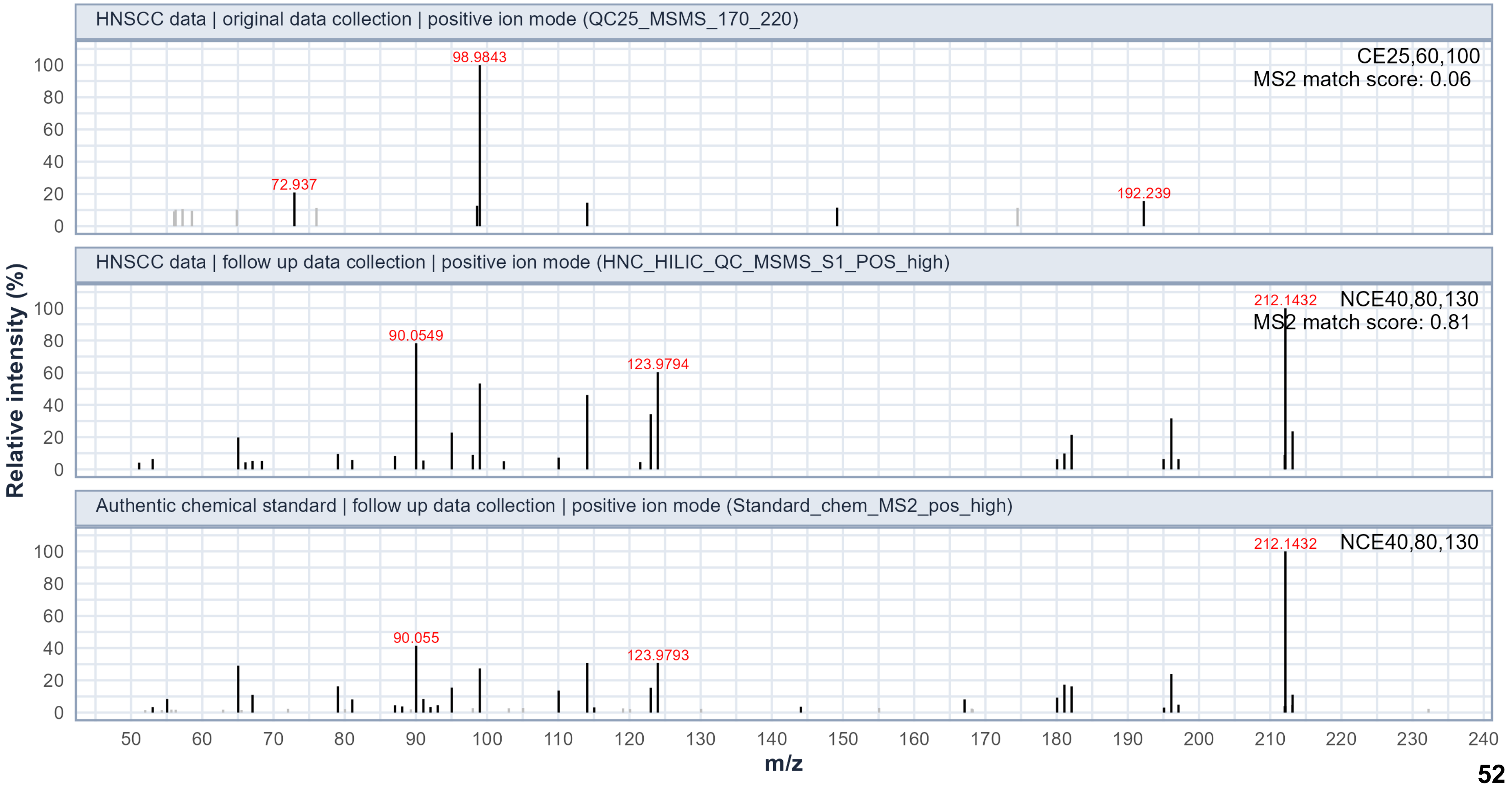

# Putrescine [M+H]+ | HMDB0001414

Positive ion mode: 89.1073 m/z | Instrument: QE focus

## Chromatogram

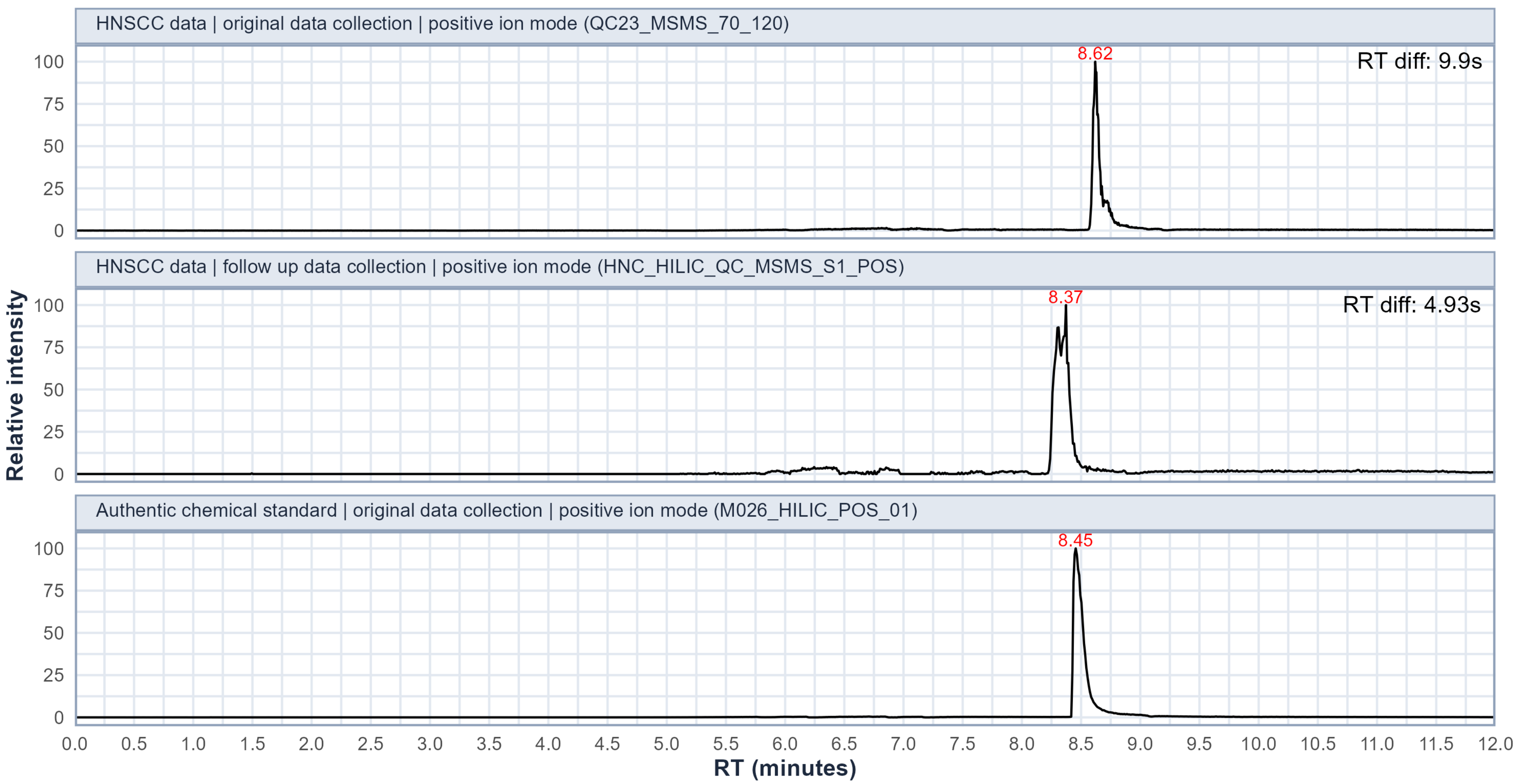


## MS/MS

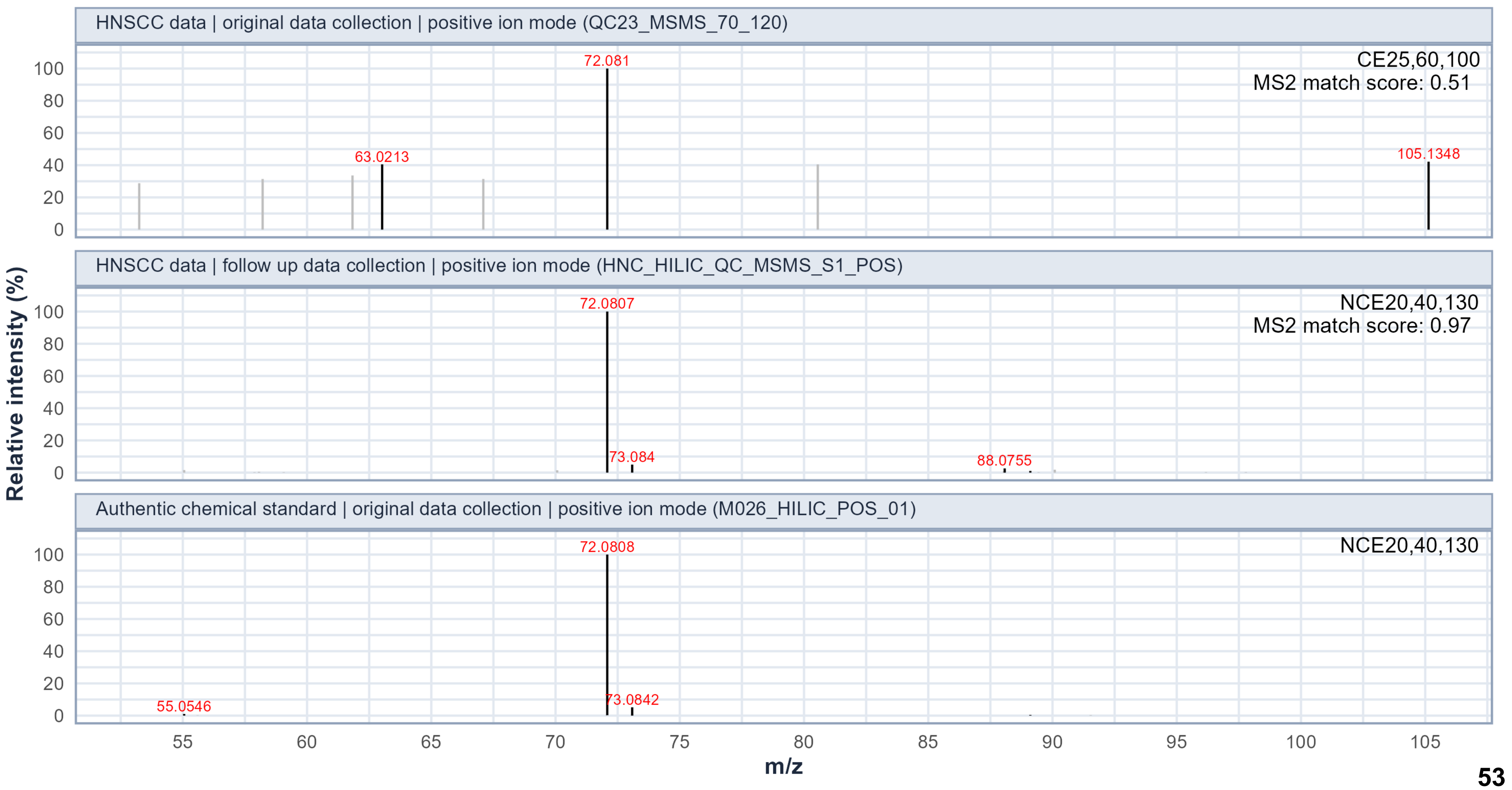

# Reduced glutathione [M+H]+ (GSH) | HMDB00143

Positive ion mode: 308.0911 m/z | Instrument: QE focus

## Chromatogram

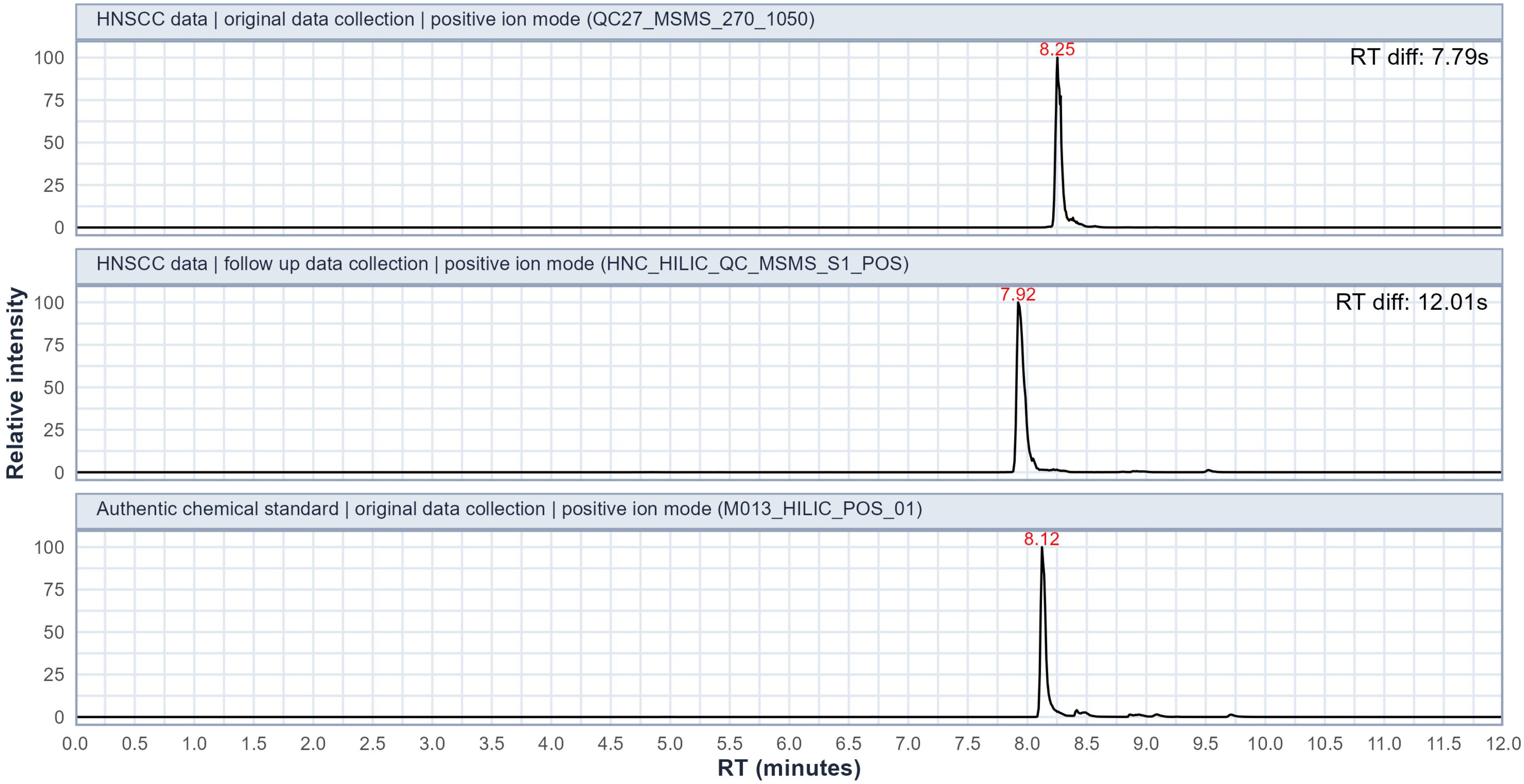


## MS/MS

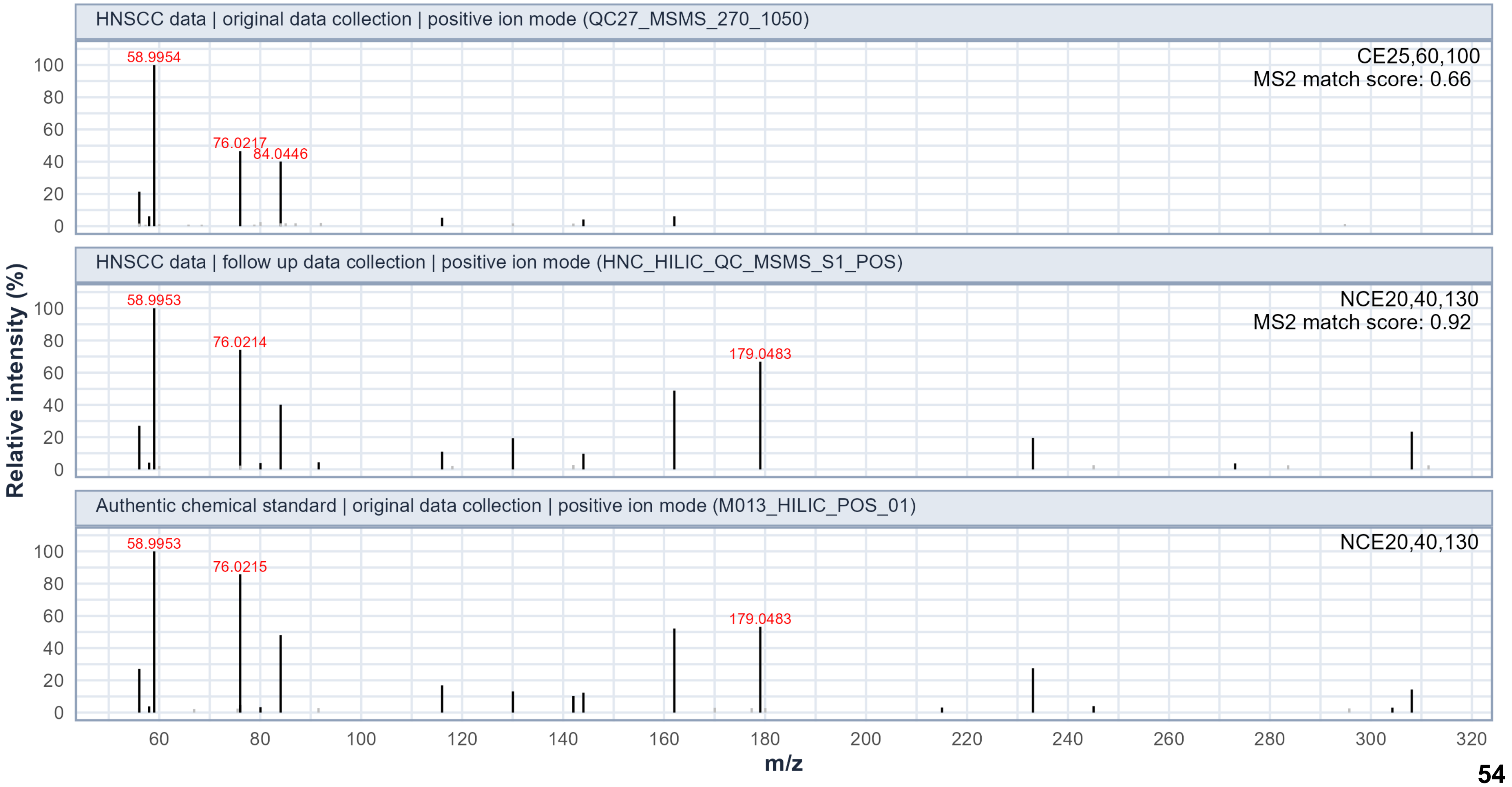

# S-adenosylhomocysteine [M+H]+ | HMDB00939

Positive ion mode: 385.1289 m/z | Instrument: QE focus

## Chromatogram

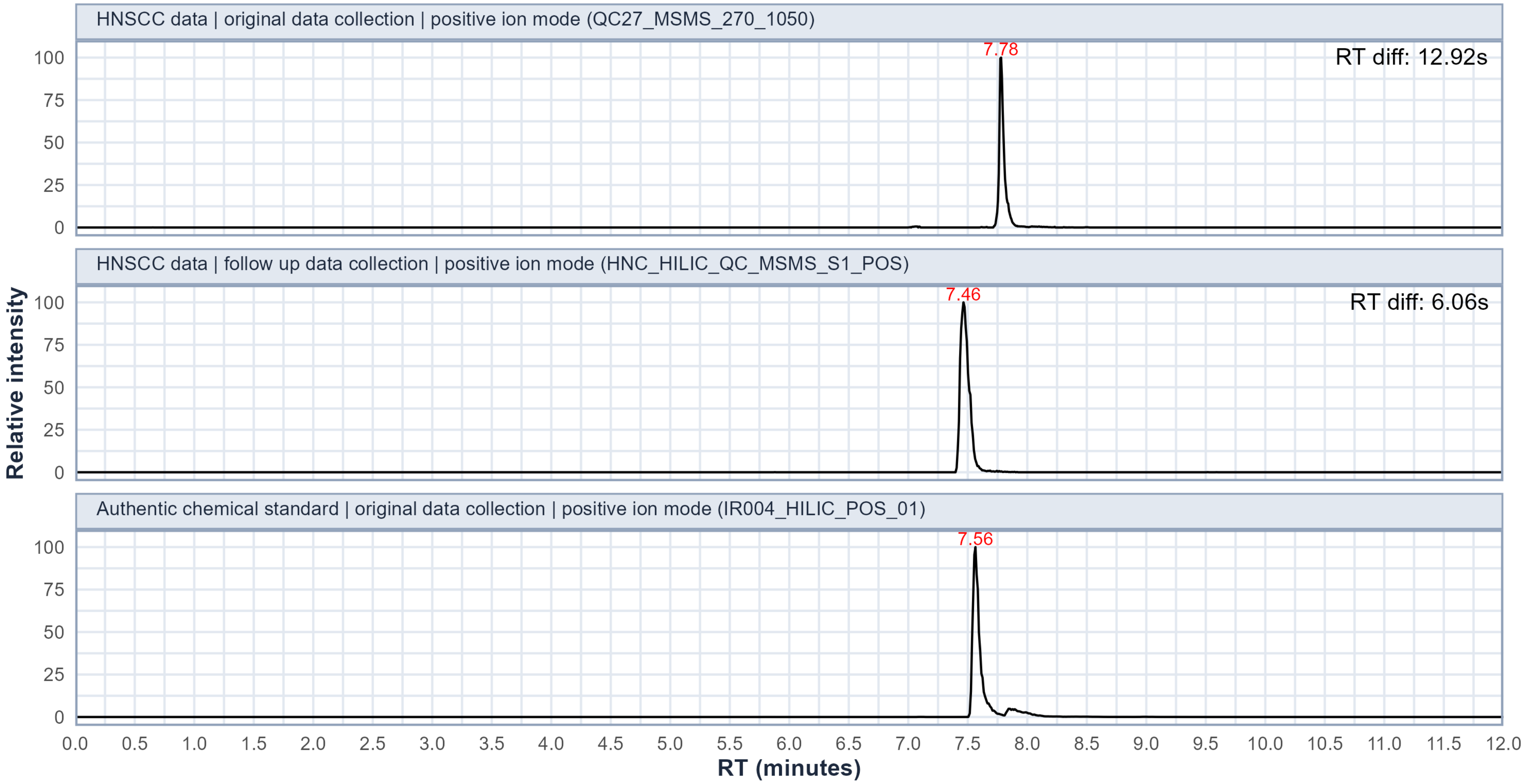


## MS/MS

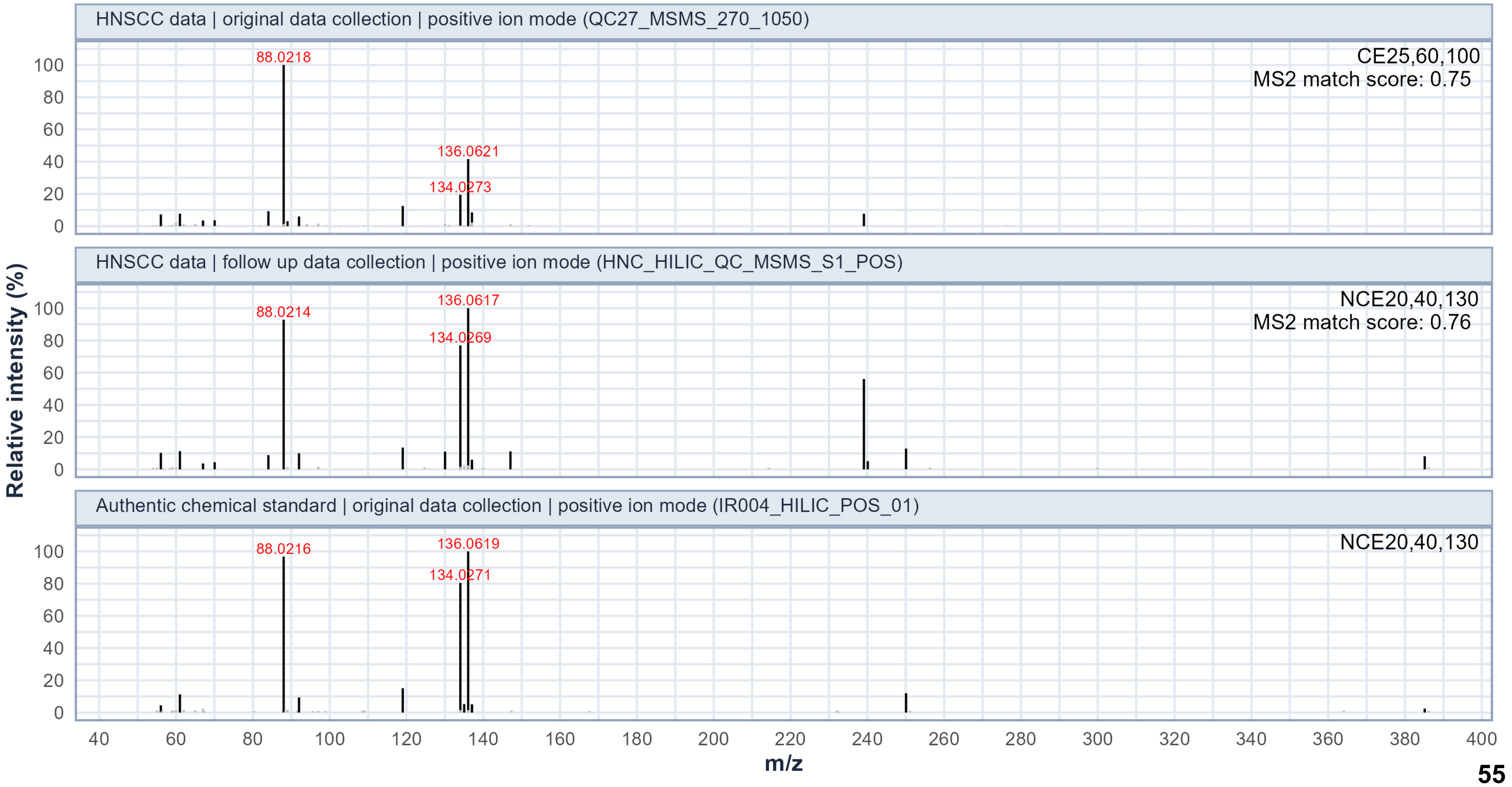

# S-adenosylmethionine [M-e]+ | HMDB0001185

Positive ion mode: 399.1445 m/z | Instrument: QE focus

## Chromatogram

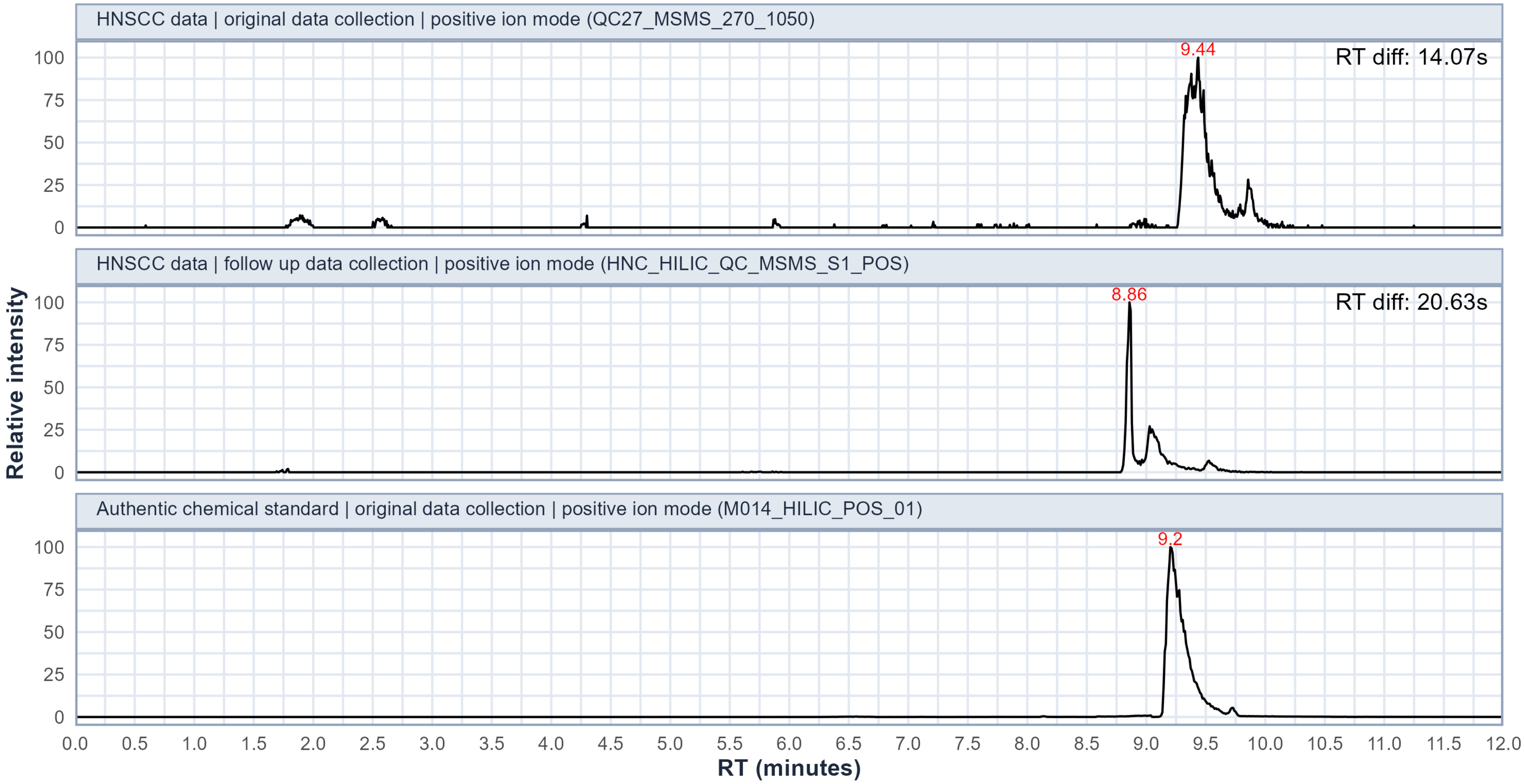


## MS/MS

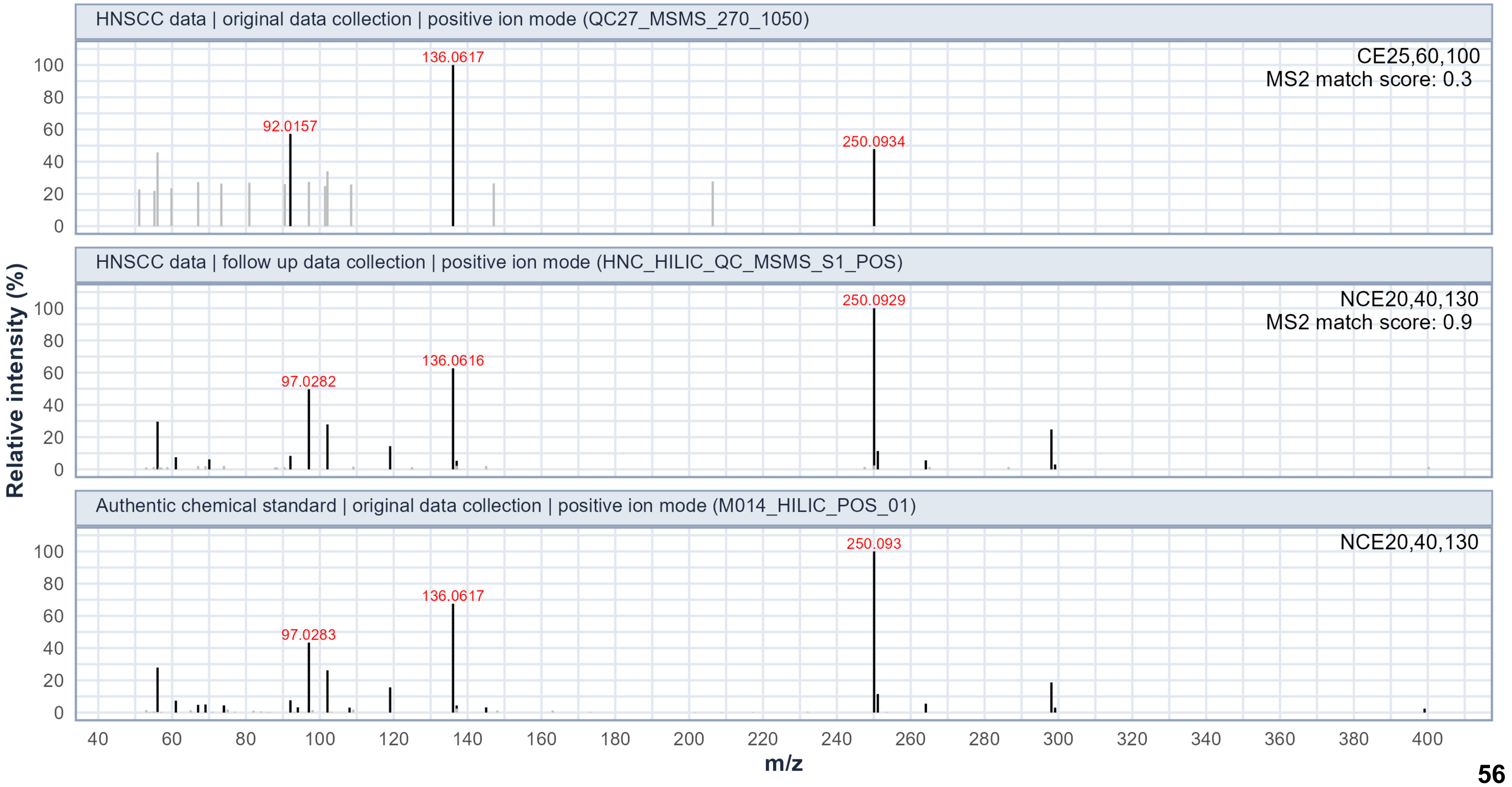

# Xanthine [M-H]- | HMDB0000292

Negative ion mode: 151.0262 m/z | Instrument: QE focus

## Chromatogram

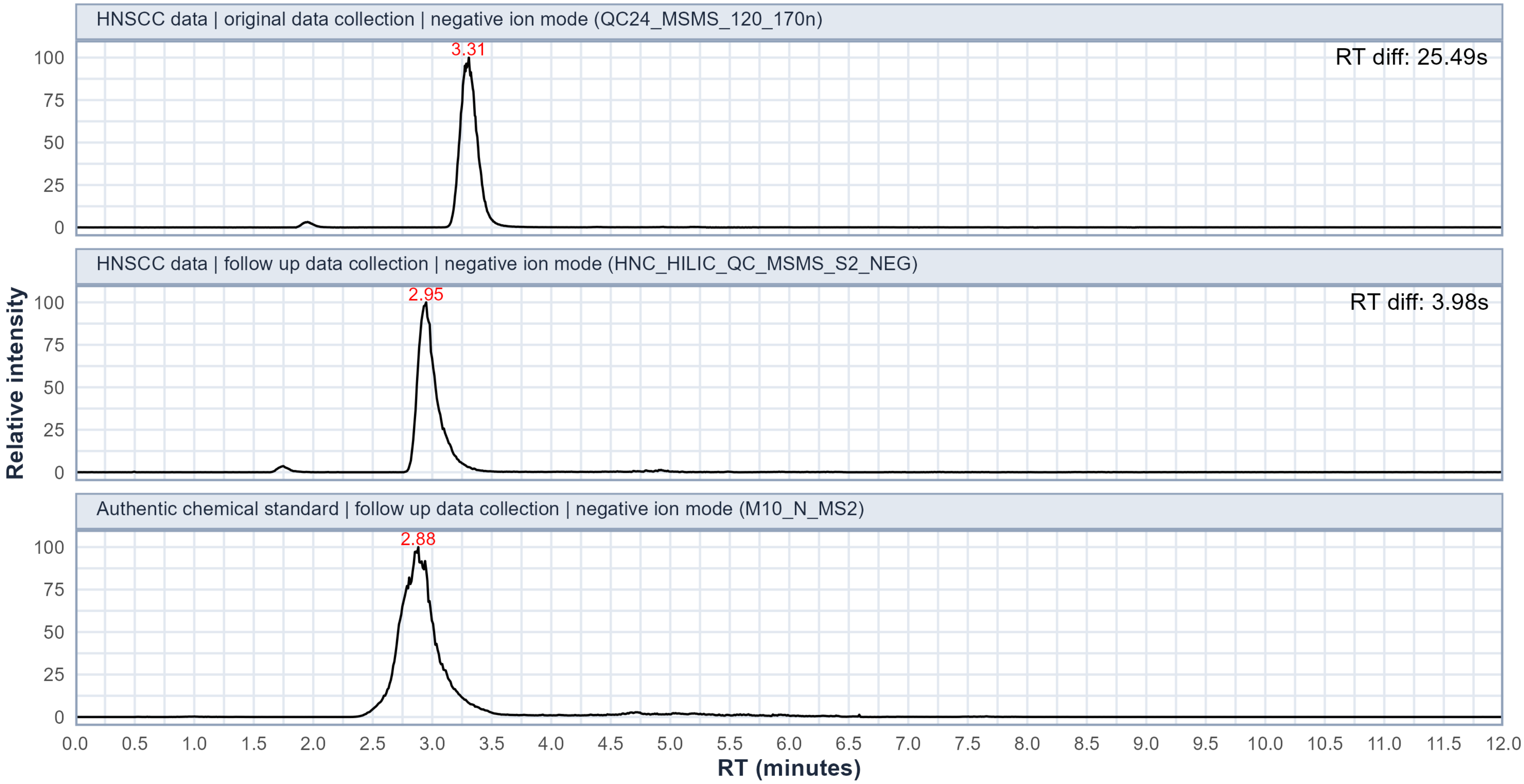


## MS/MS

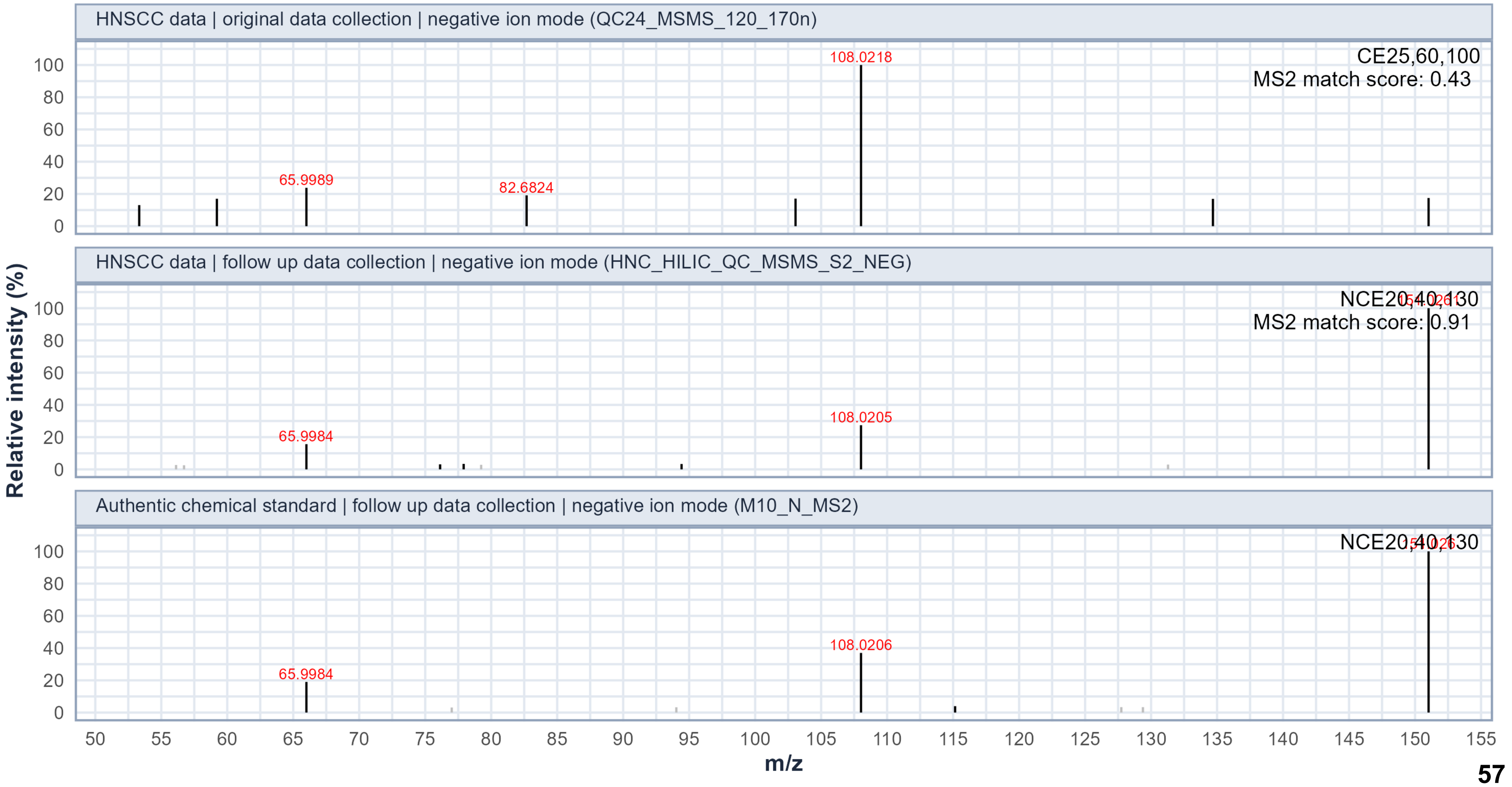

# Butyrylcarnitine [M+H]+ | HMDB0002013

Positive ion mode: 232.1543 m/z | Instrument: QE focus

## Chromatogram

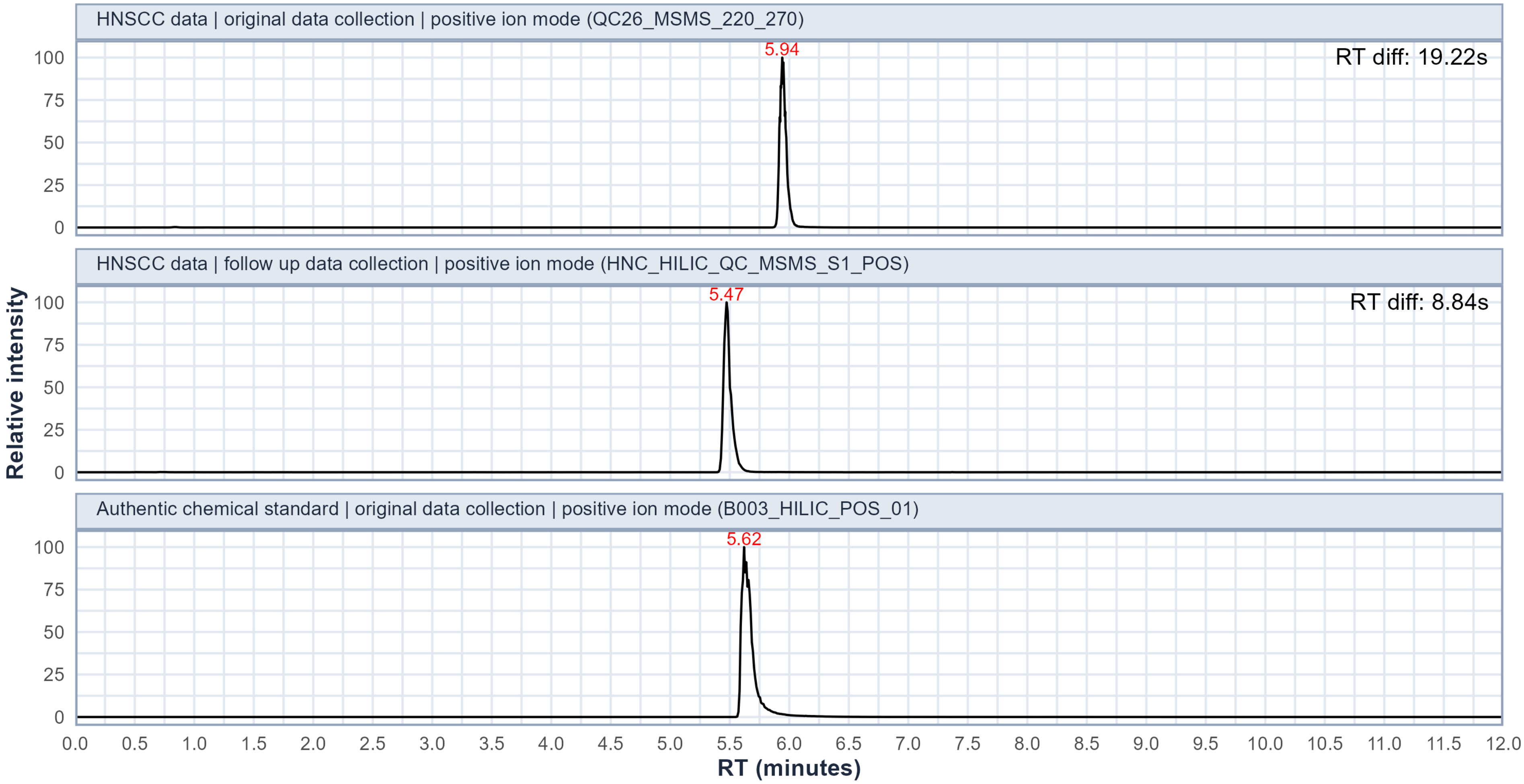


## MS/MS

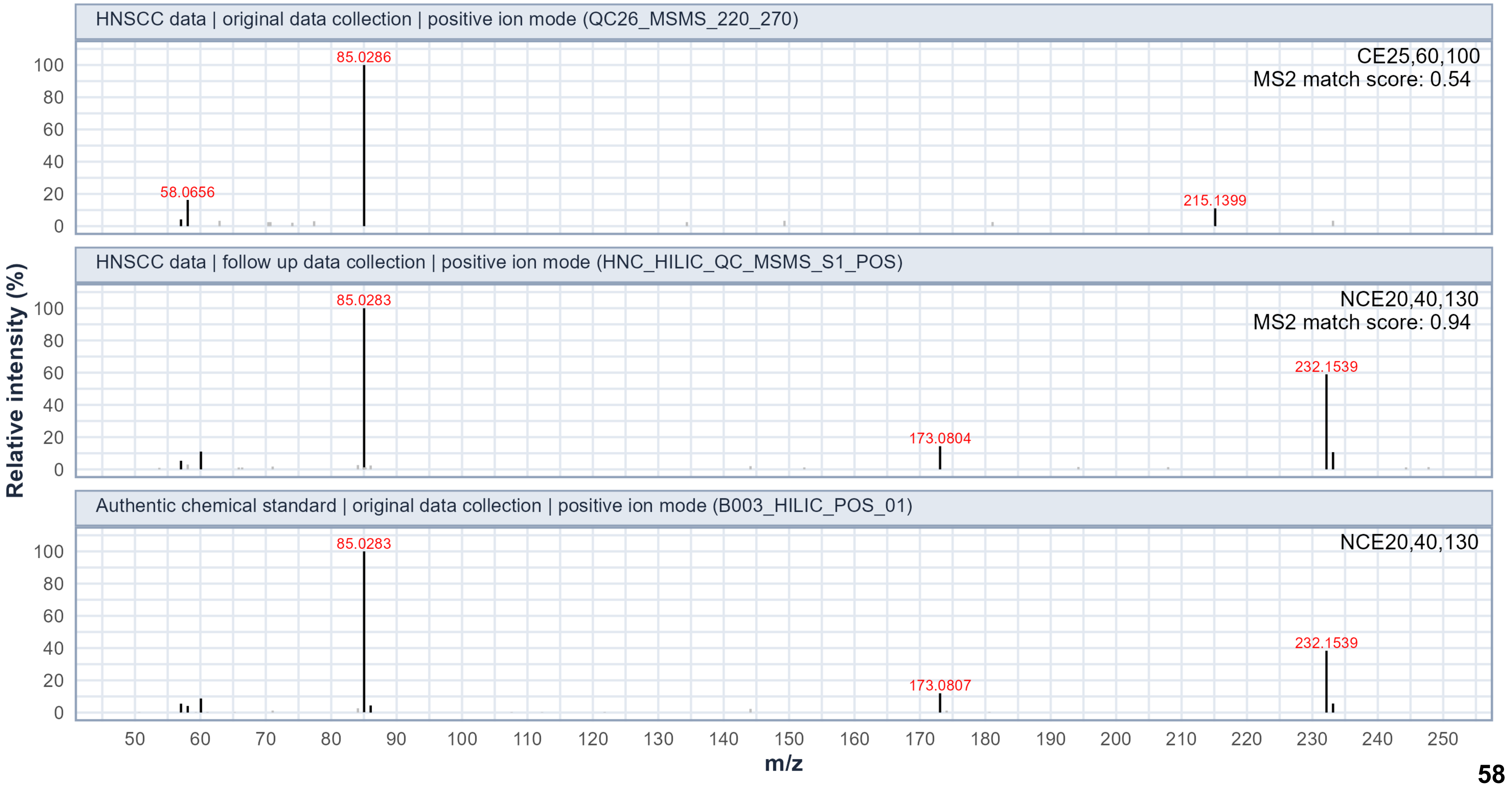

# D-Sedoheptulose 7-phosphate [M-H]- | HMDB0001068

Negative ion mode: 289.033 m/z | Instrument: QE focus
Authentic chemical standard not available in library; identification based on MS/MS match to mzCloud.

## Chromatogram

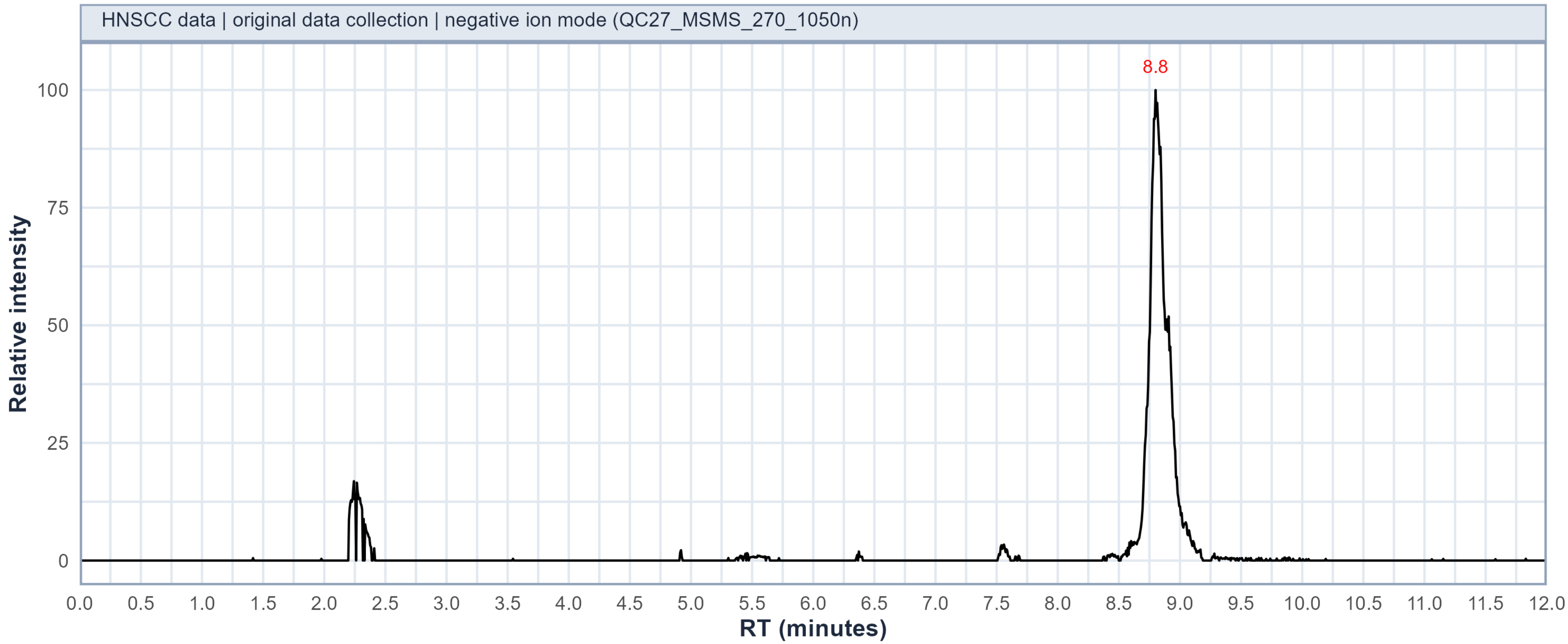


## MS/MS

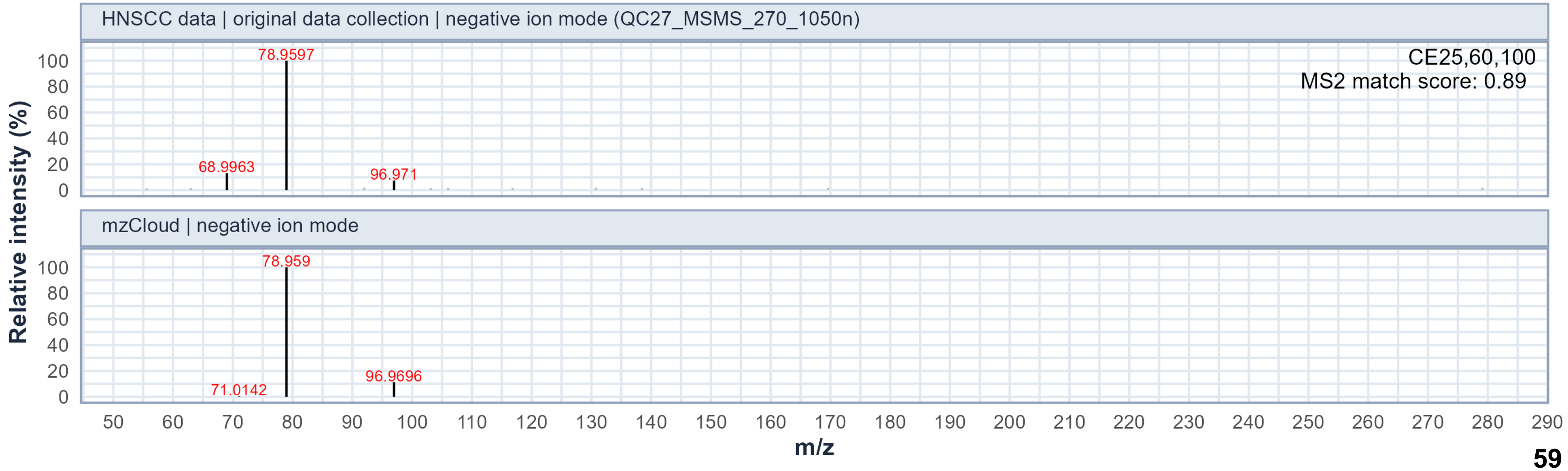

# L-Asparagine [M+H]+ | HMDB0000168

Positive ion mode: 133.0608 m/z | Instrument: QE focus

## Chromatogram

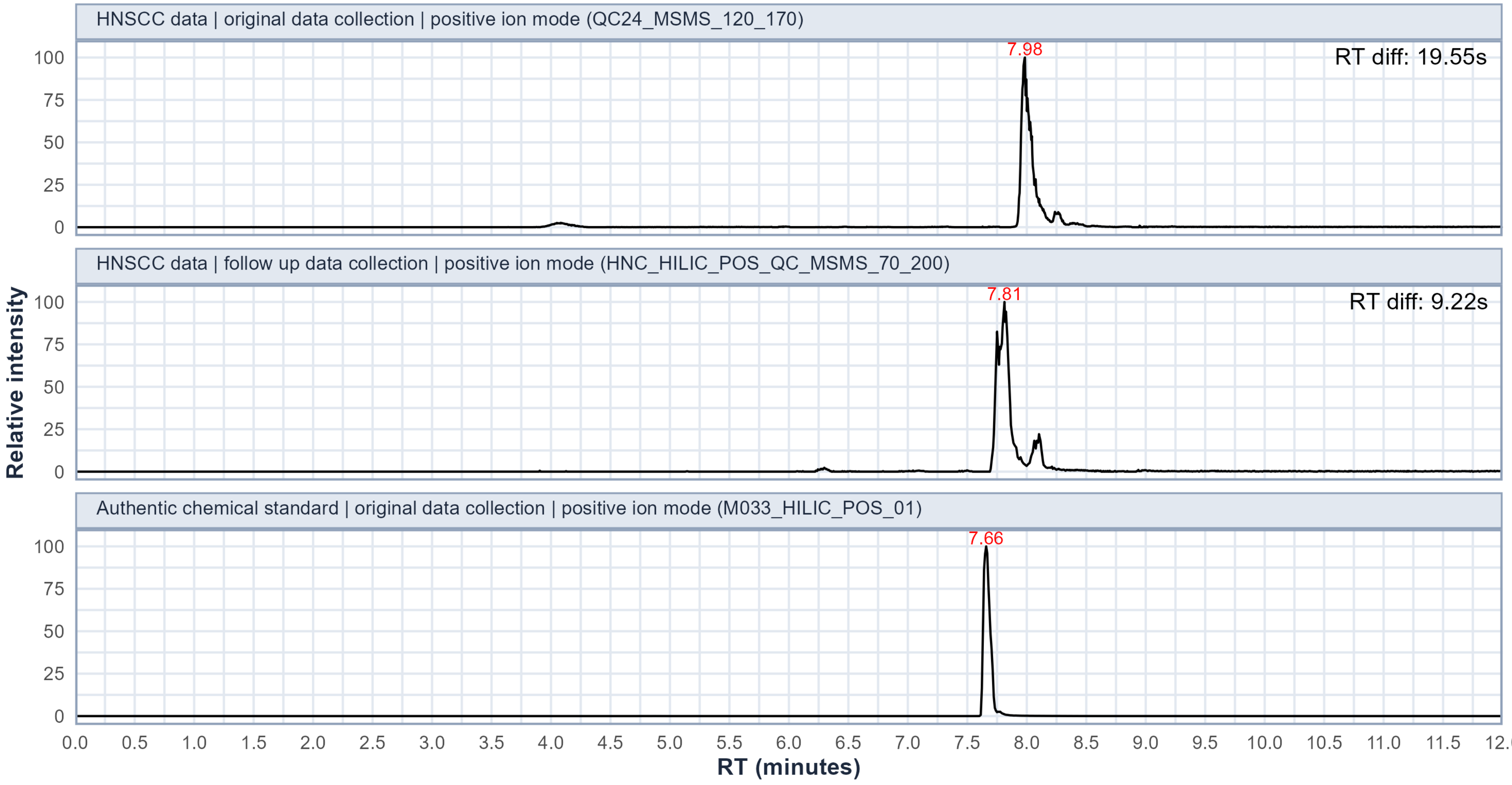


## MS/MS

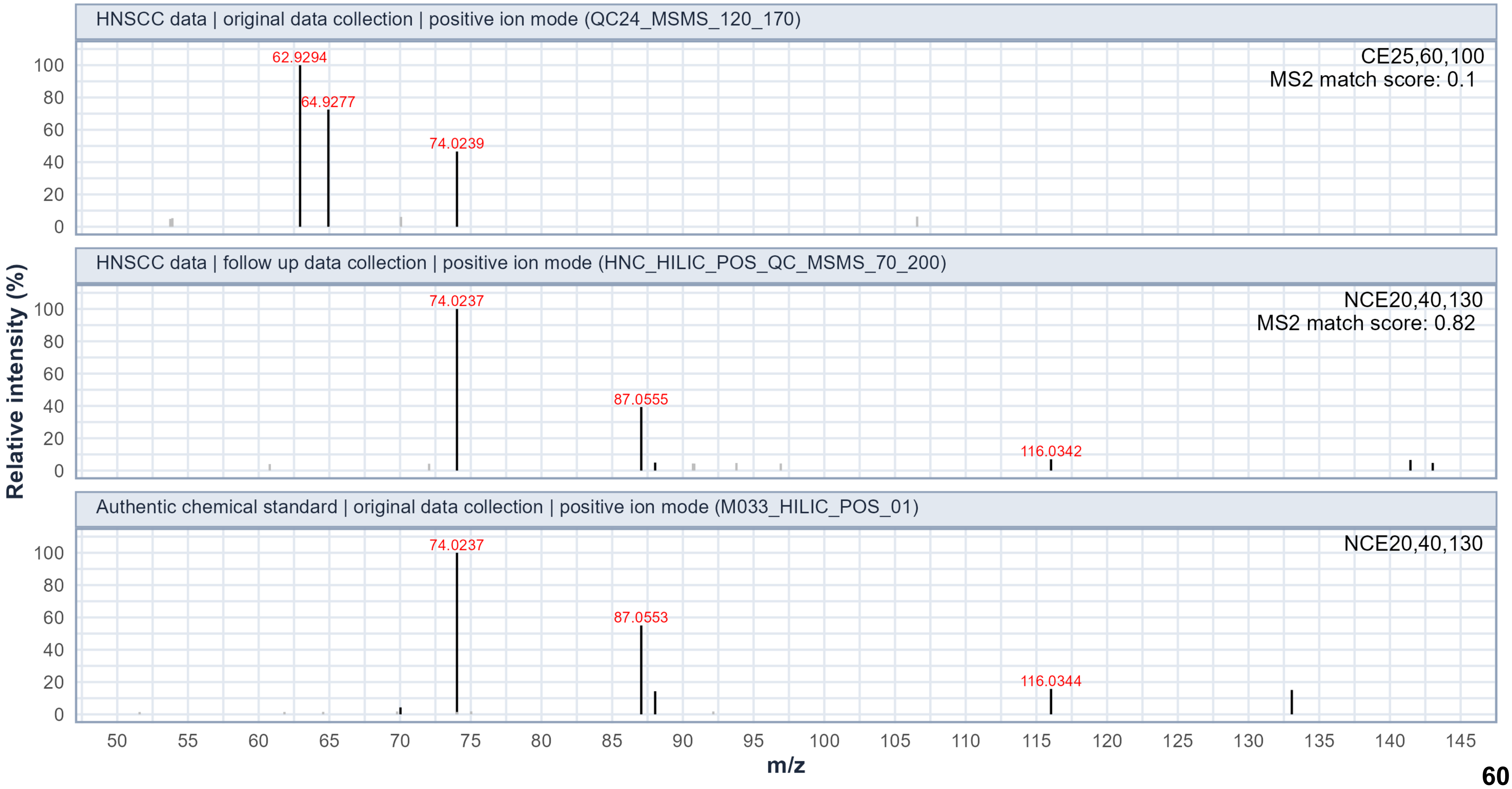

# Lysophosphatidylinositol(16:0) [M-H]- | HMDB0061695

Negative ion mode: 571.2889 m/z | Instrument: QE focus
The MS1 accurate mass suggests LPI(16:0).
Fragmentation product ions at 241 m/z (inositol ring - H2O) and 153 m/z (glycerol backbone) are indicative of a lysophosphatidylinositol (Pi et al. 2
A fragmentation product ion at m/z 253 indicates an 16:0 fatty acyl chain.

## Chromatogram

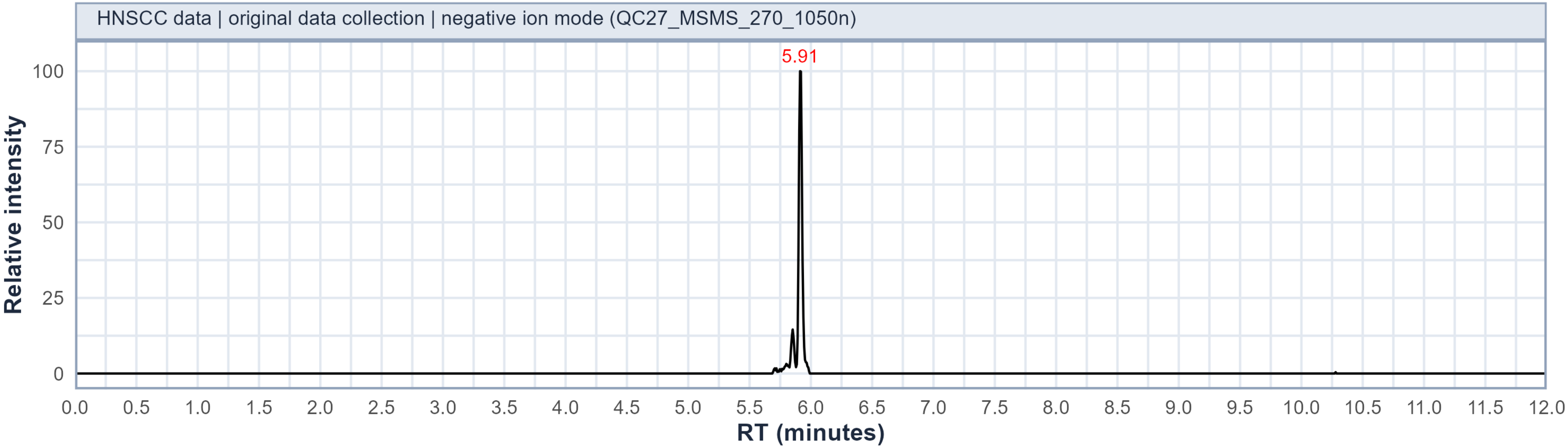


## MS/MS

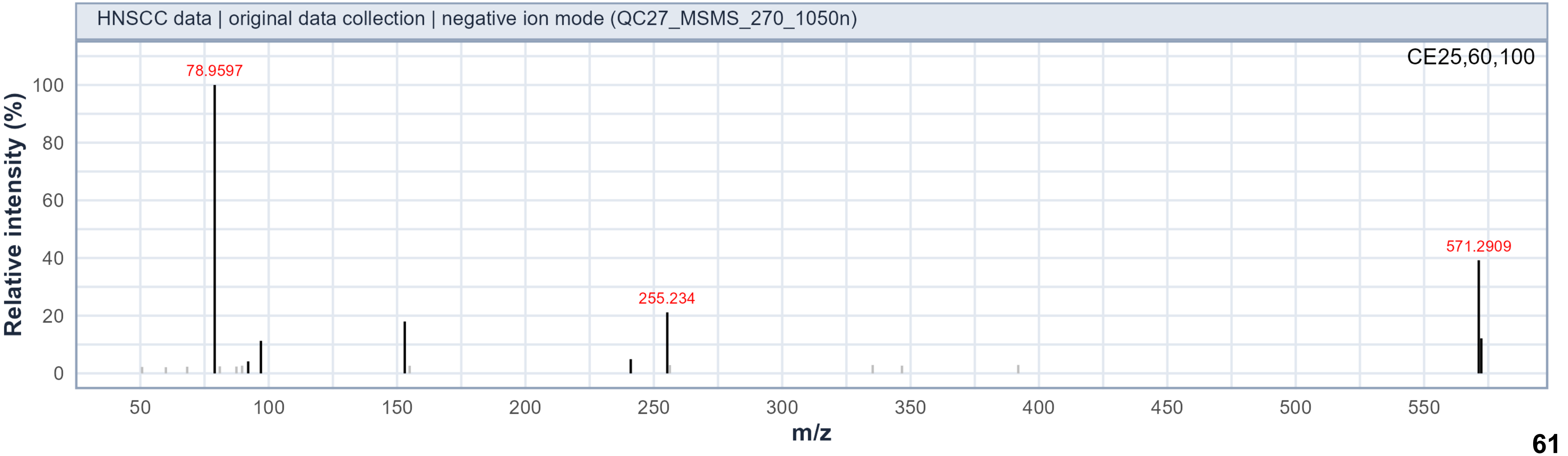

# Lysophosphatidylinositol(18:0) [M-H]- | HMDB0240261

Negative ion mode: 599.3202 m/z | Instrument: QE focus
The MS1 accurate mass suggests LPI(18:0).
Fragmentation product ions at 241 m/z (inositol ring - H2O) and 153 m/z (glycerol backbone) are indicative of a lysophosphatidylinositol (Pi et al. 2
A fragmentation product ion at 283 m/z indicates an 18:0 fatty acyl chain.

## Chromatogram

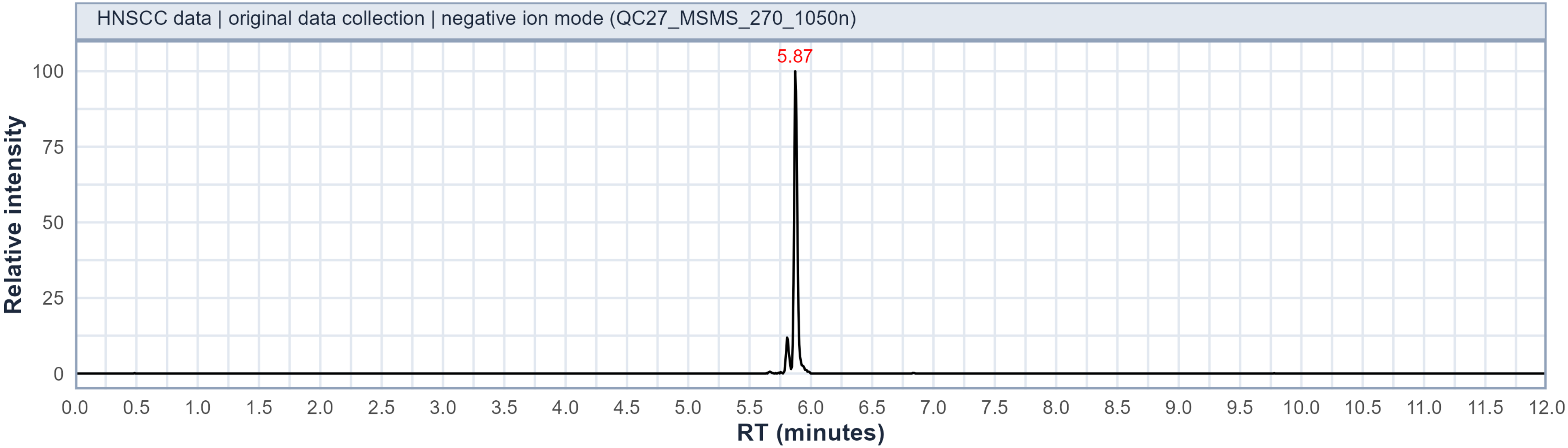


## MS/MS

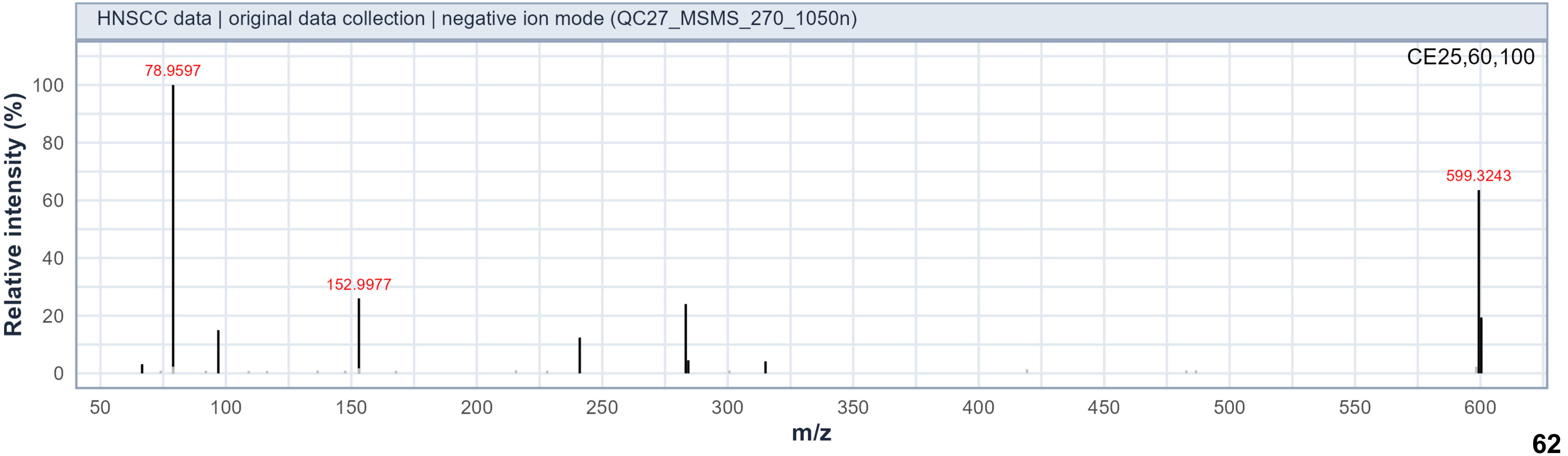

# Lysophosphatidylinositol(18:1) [M-H]- | HMDB0061693

Negative ion mode: 597.3045 m/z | Instrument: QE focus
The MS1 accurate mass suggests LPI(18:1).
Fragmentation product ions at 241 m/z (inositol ring - H2O) and 153 m/z (glycerol backbone) are indicative of a lysophosphatidylinositol (Pi et al. 2
A fragmentation product ion at 281 m/z indicates an 18:1 fatty acyl chain.

## Chromatogram

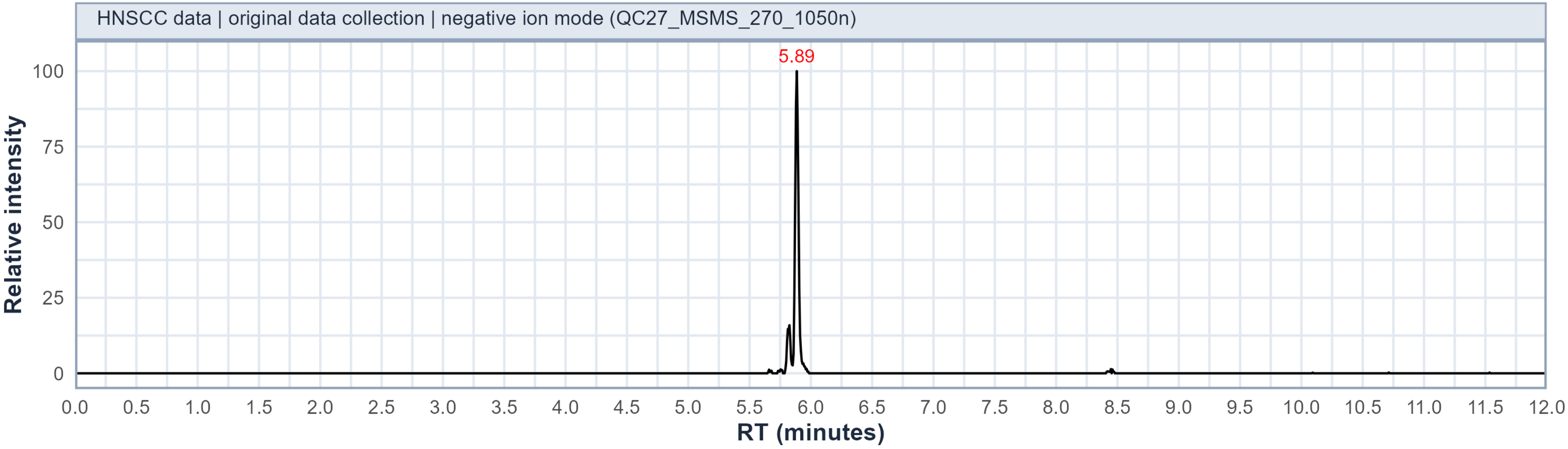


## MS/MS

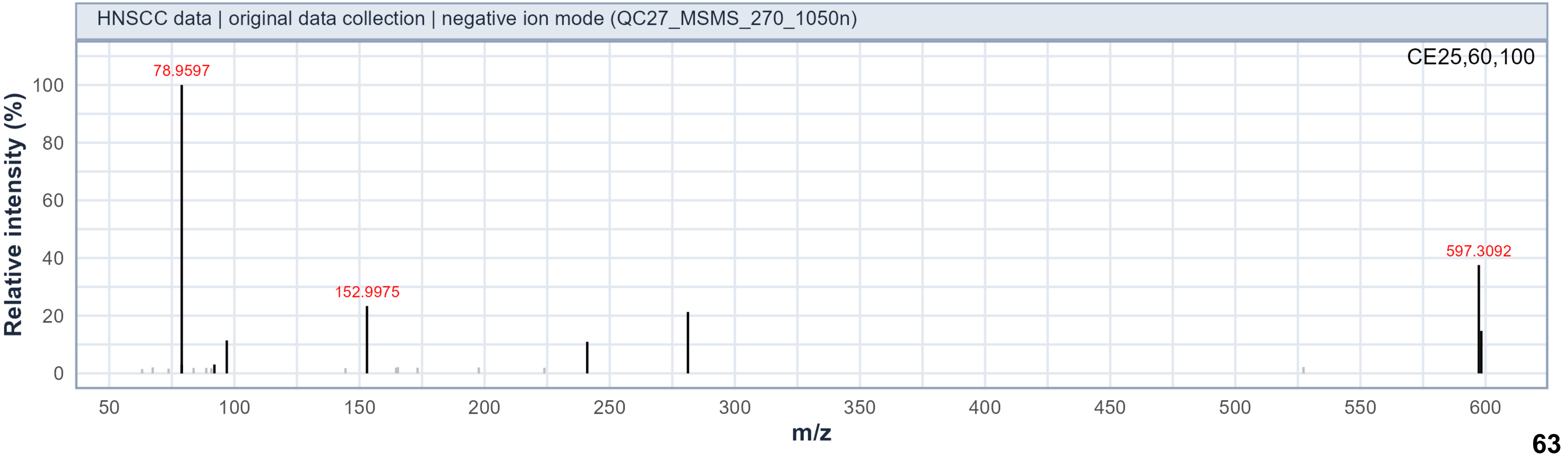

# Palmitolycarnitine [M+H]+ | HMDB0002013

Positive ion mode: 400.3421 m/z | Instrument: QE focus

## Chromatogram

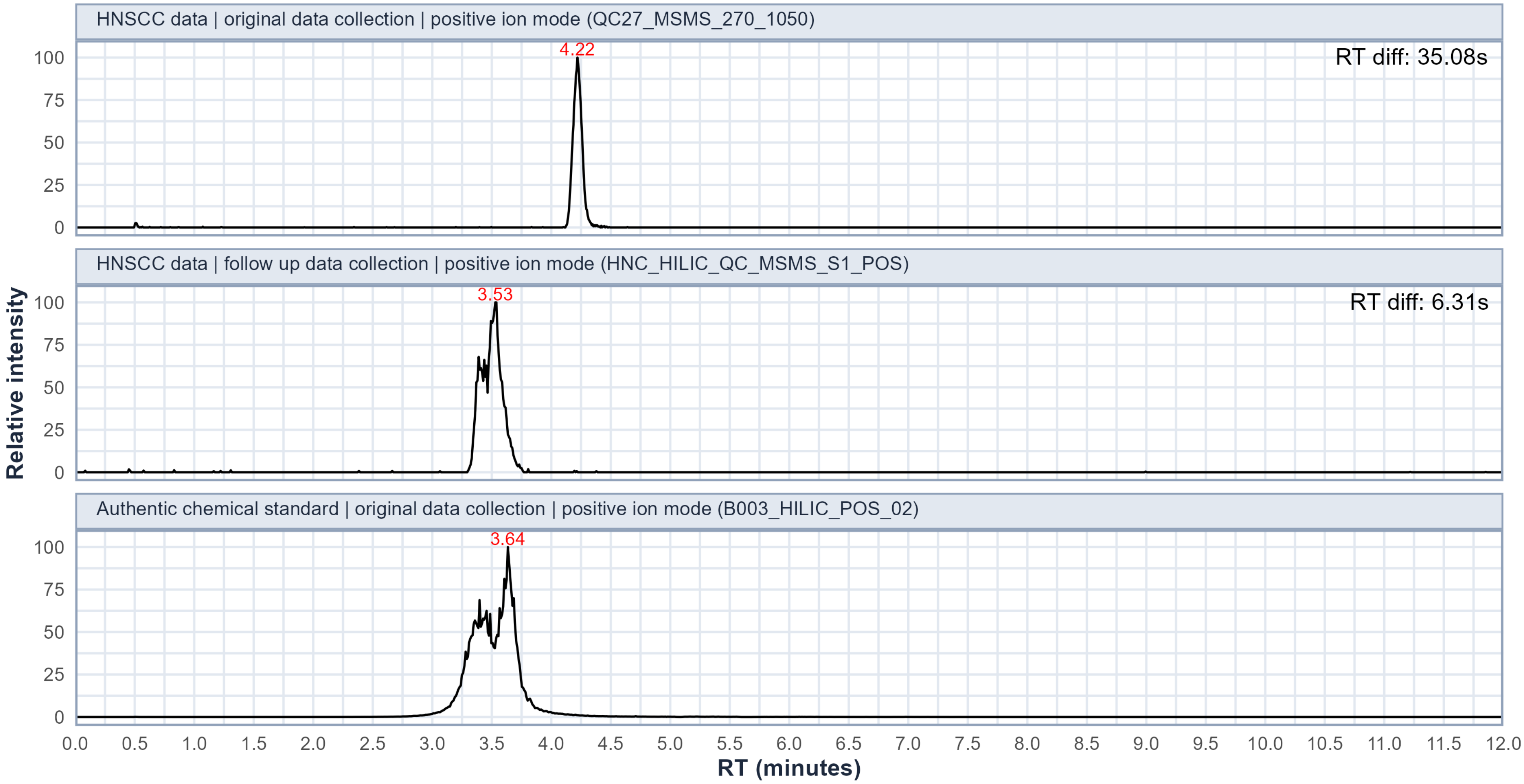


## MS/MS

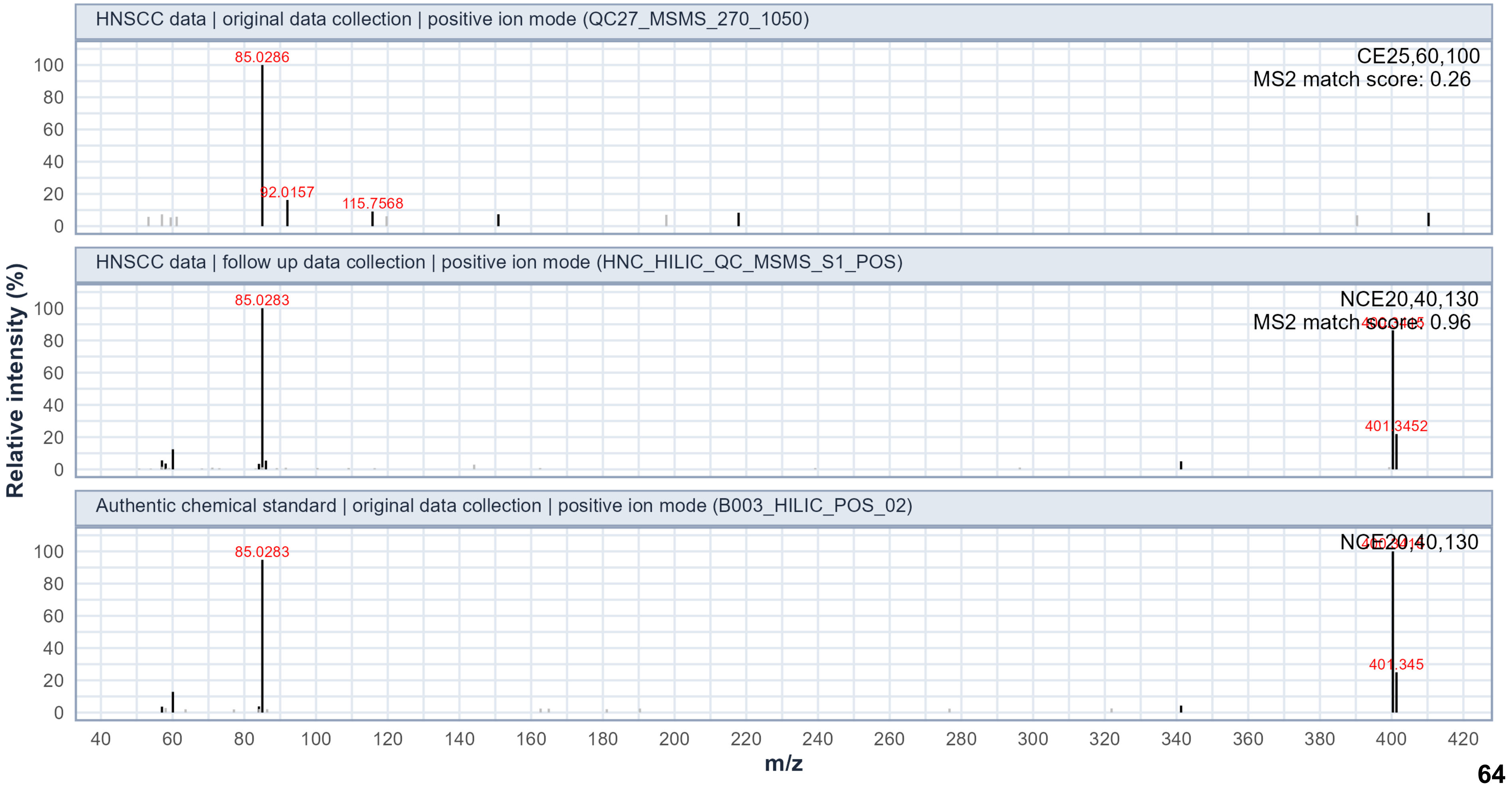

# Pantothenic acid [M+H]+ | HMDB0000210

Positive ion mode: 220.1179 m/z | Instrument: QE focus

## Chromatogram

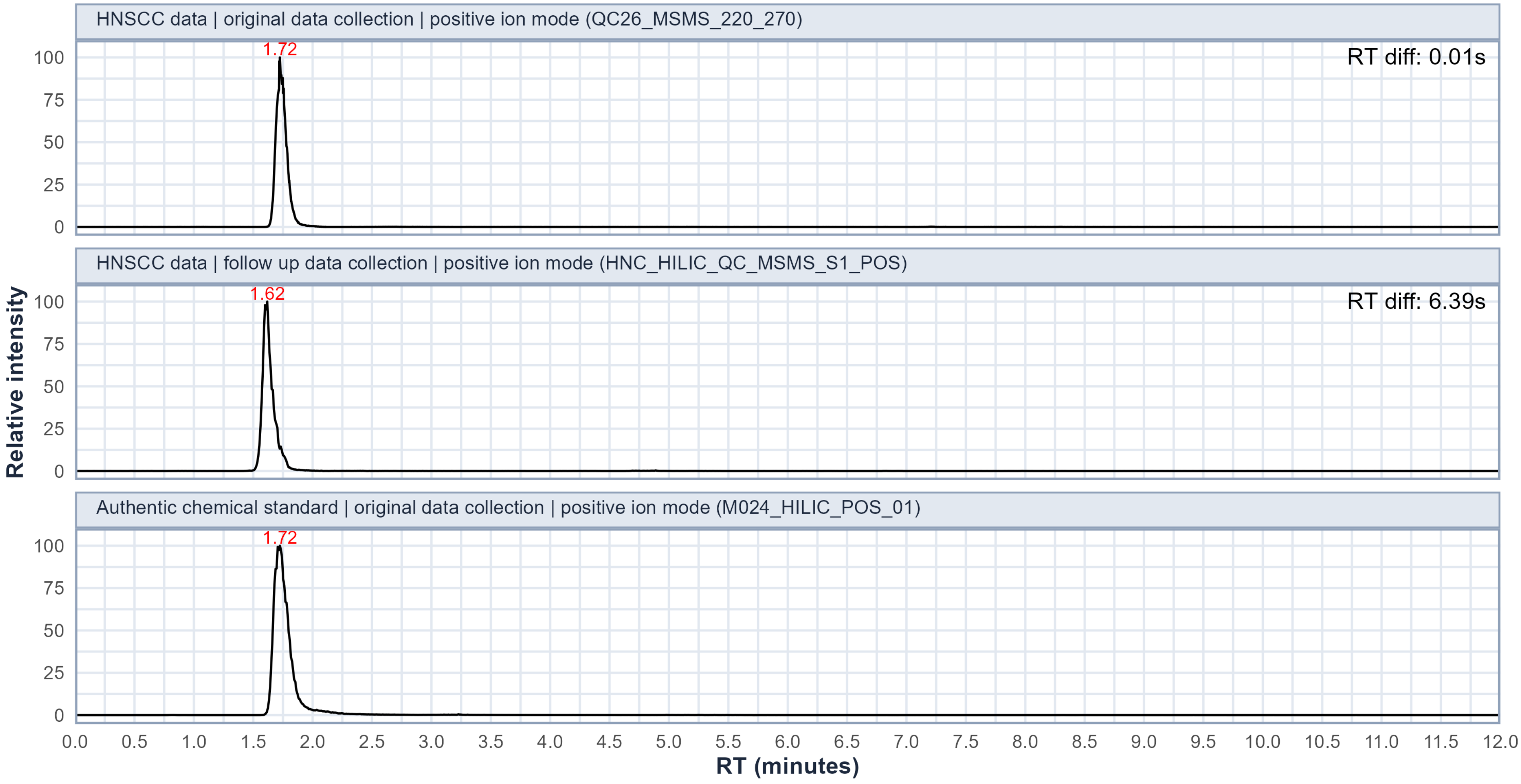


## MS/MS

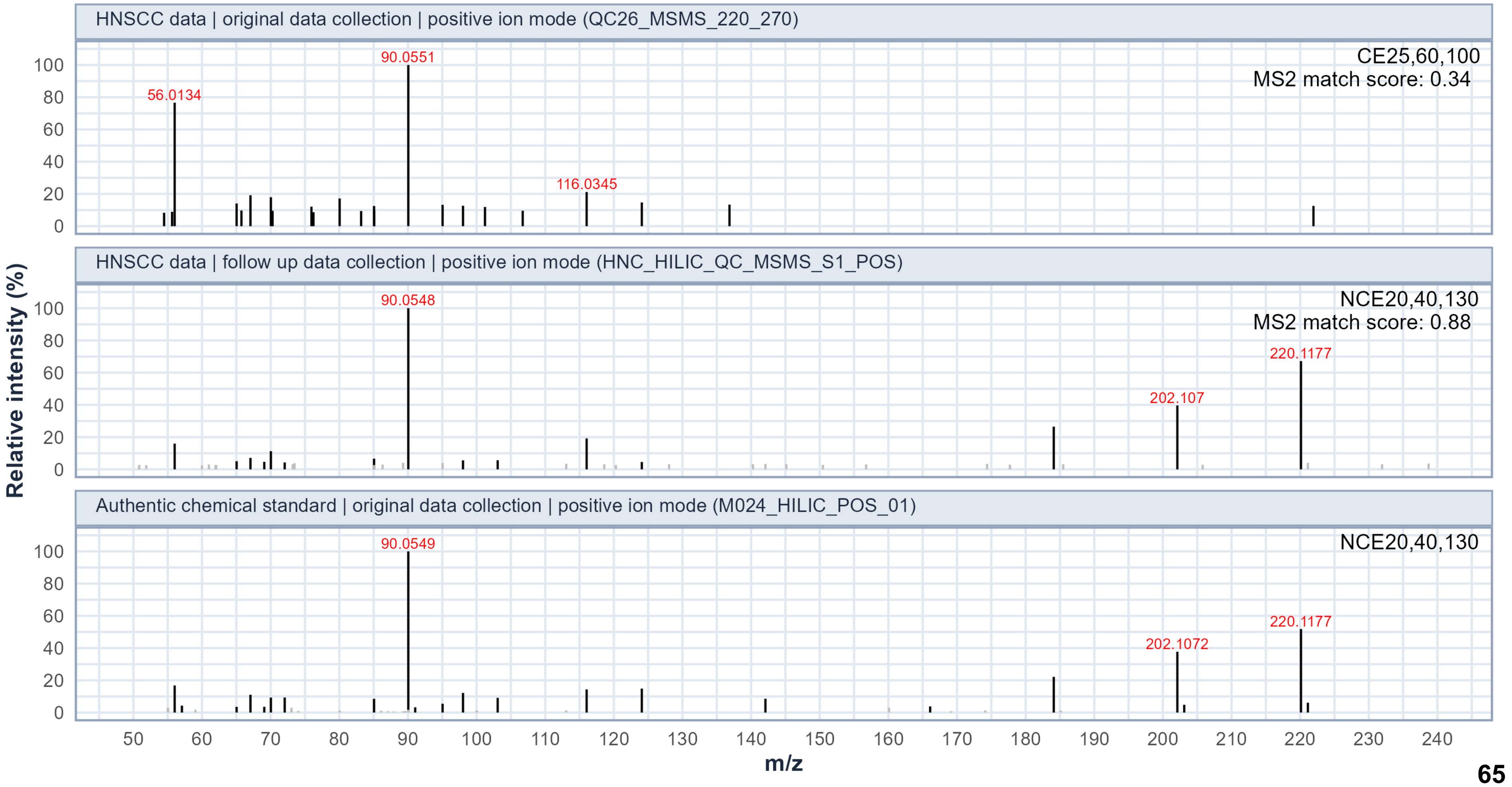

# Tetradecanoylcarnitine [M+H]+ | HMDB0005066

Positive ion mode: 372.3108 m/z | Instrument: QE focus
The MS1 accurate mass suggests tetradecanoylcarnitine.
A fragmentation product ion at 85 m/z is a key indicator of an acylcarnitine species (Yan et al. 2020; https://doi.org/10.1021/acs.analchem.0c00129

## Chromatogram

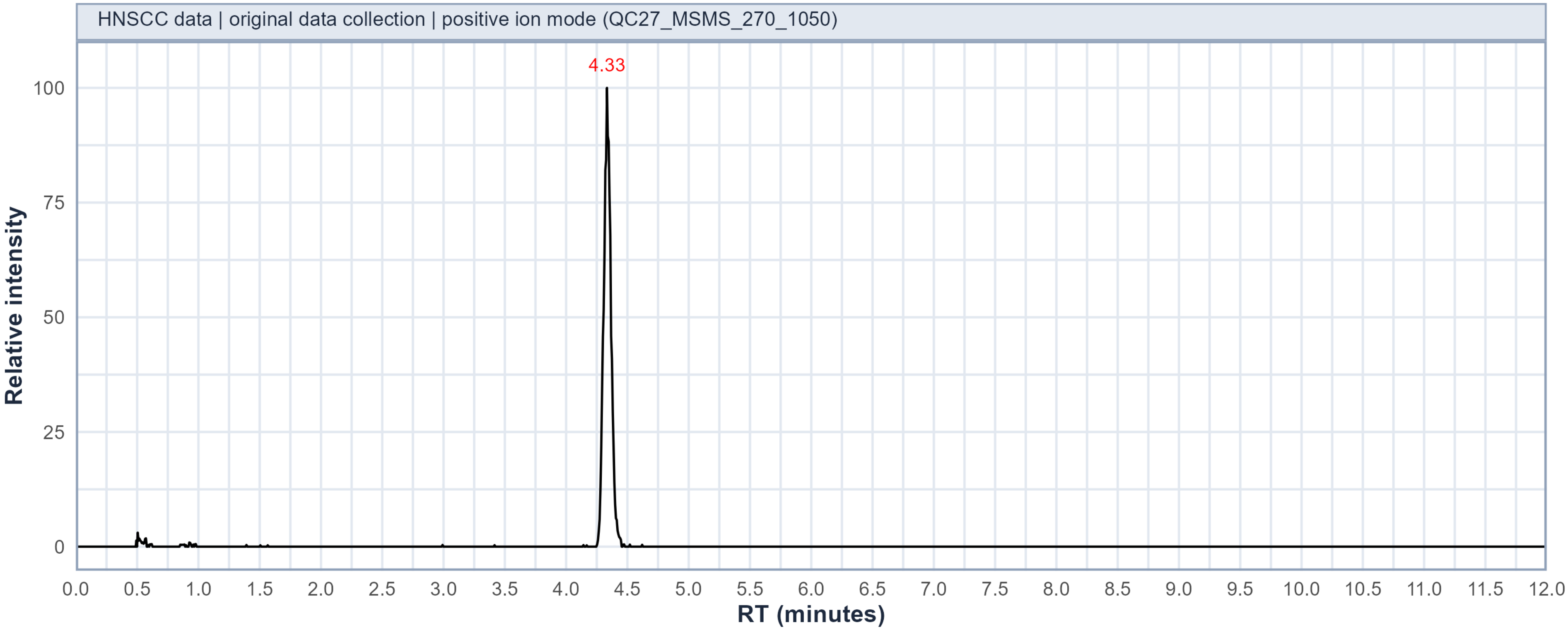


## MS/MS

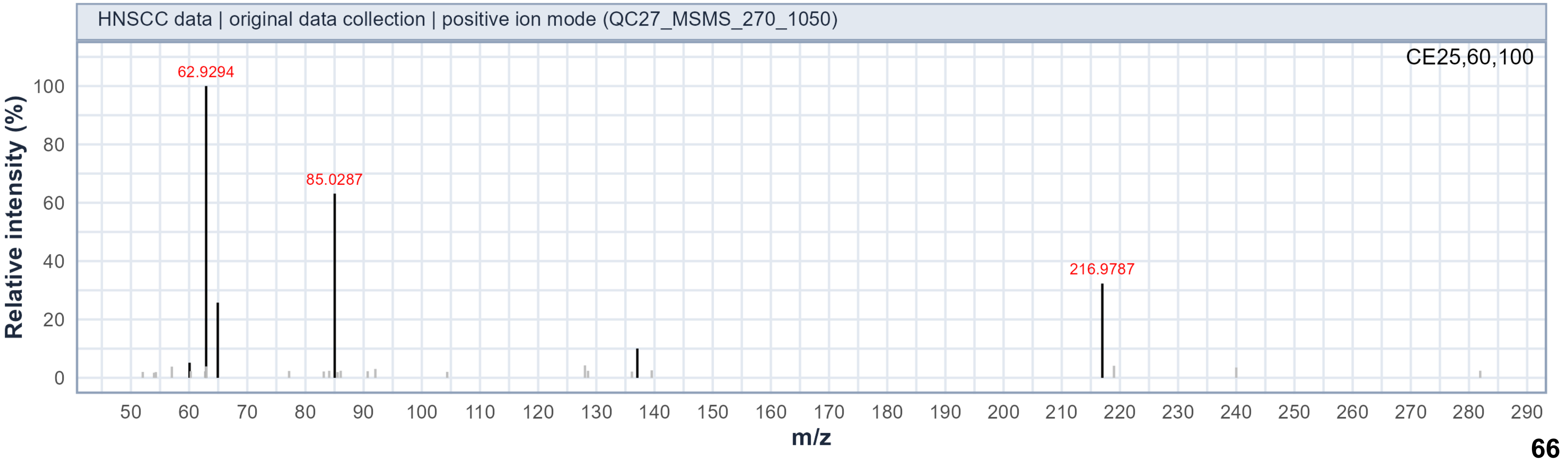

# UDP-N-acetylglucosamine [M+H]+ | HMDB0000290

Positive ion mode: 608.0888 m/z | Instrument: QE focus

## Chromatogram

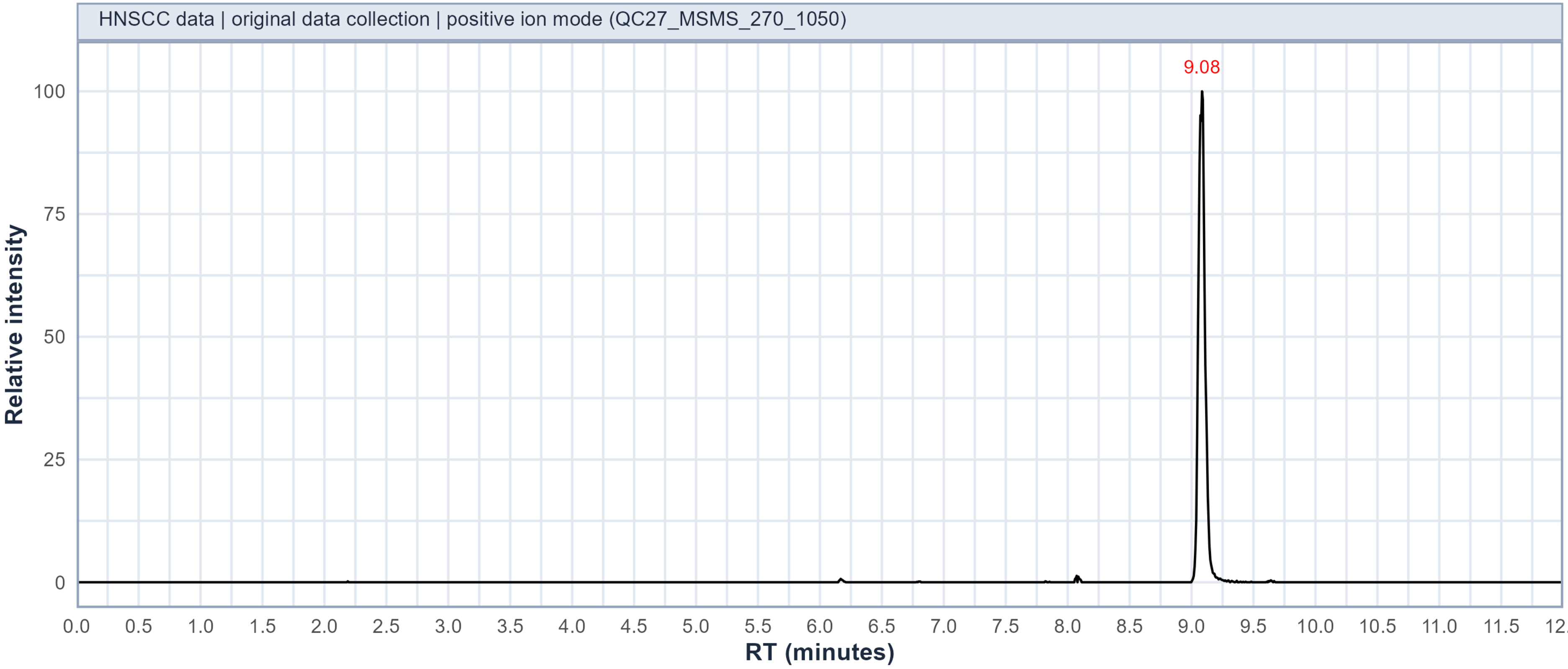


## MS/MS

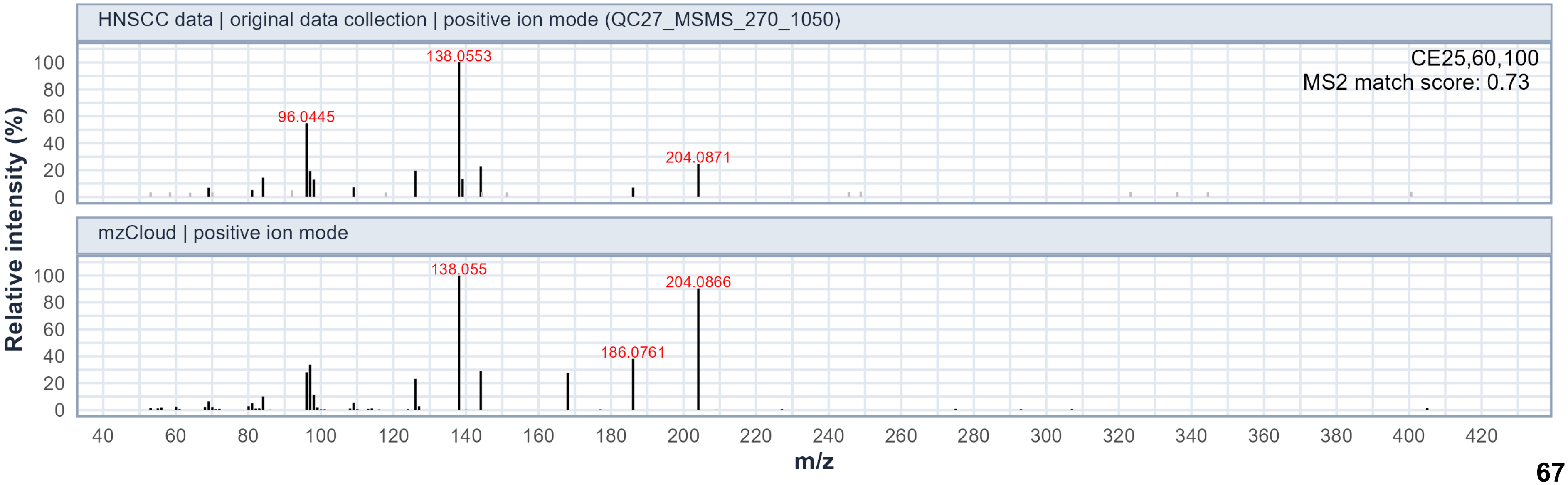

# Uridine 5'-diphosphoglucose [M-H]- | HMDB0000286

Negative ion mode: 565.0477 m/z | Instrument: QE focus
The MS1 accurate mass suggests uridine 5'-diphosphoglucose.
Fragmentation product ions of 323.0 m/z and 78.9 m/z are consistent with reported uridine 5'-diphosphoglucose fragmentation (Warth et al. 2016;

## Chromatogram

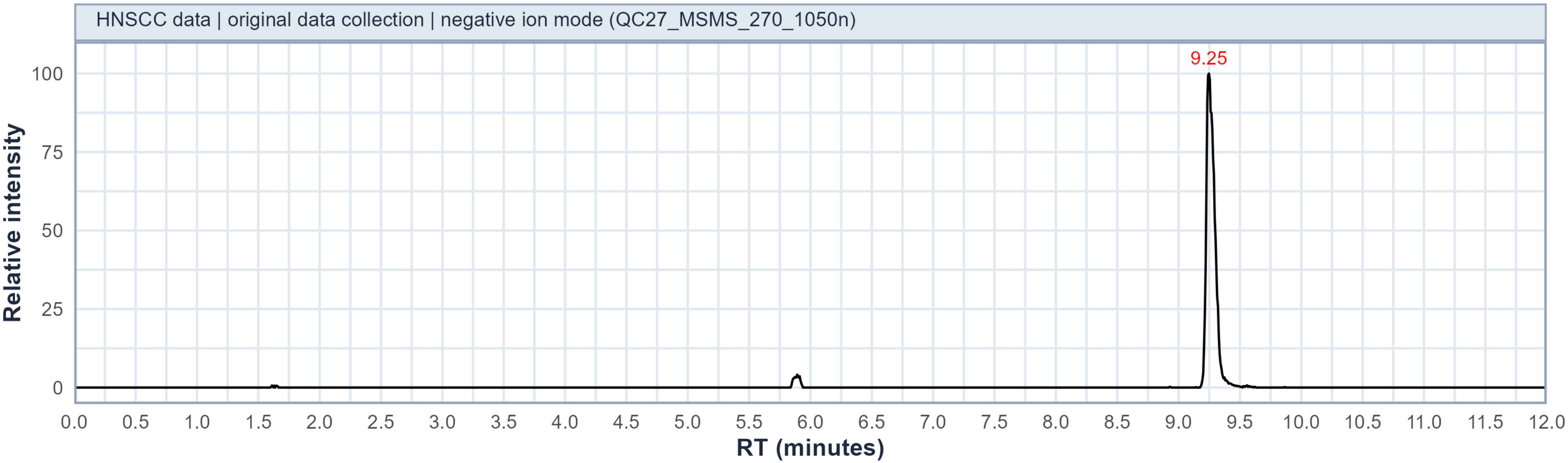


## MS/MS

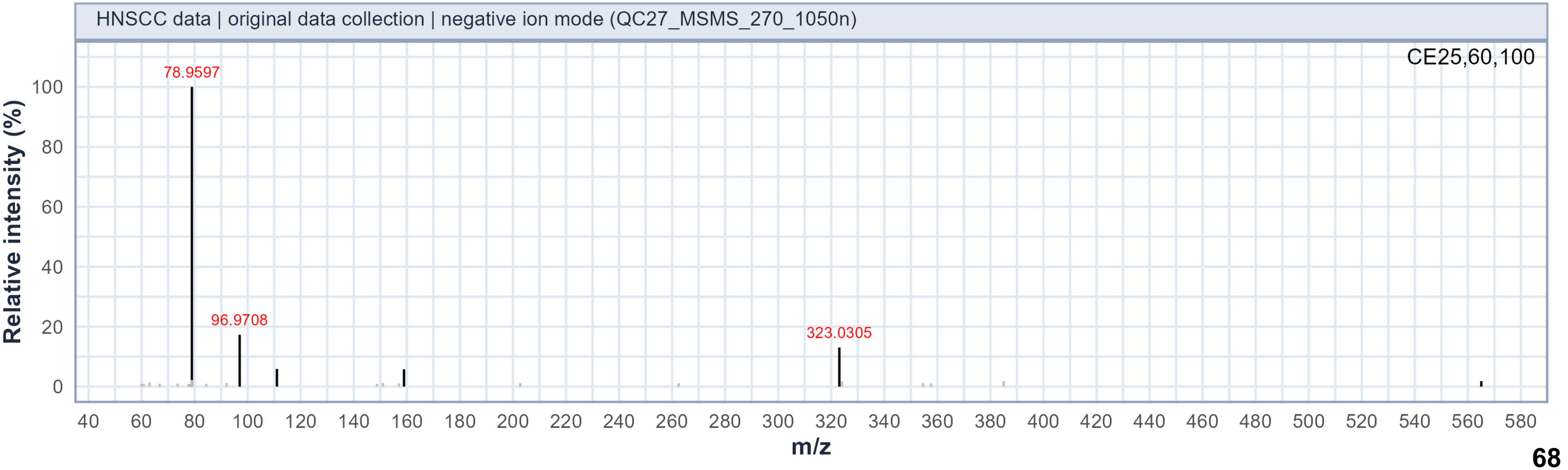

# Y-L-Glutamyl-L-glutamic acid [M+H]+ | HMDB0011737

Positive ion mode: 277.103 m/z | Instrument: QE focus
Authentic chemical standard not available in library; identification based on MS/MS match to mzCloud.

## Chromatogram

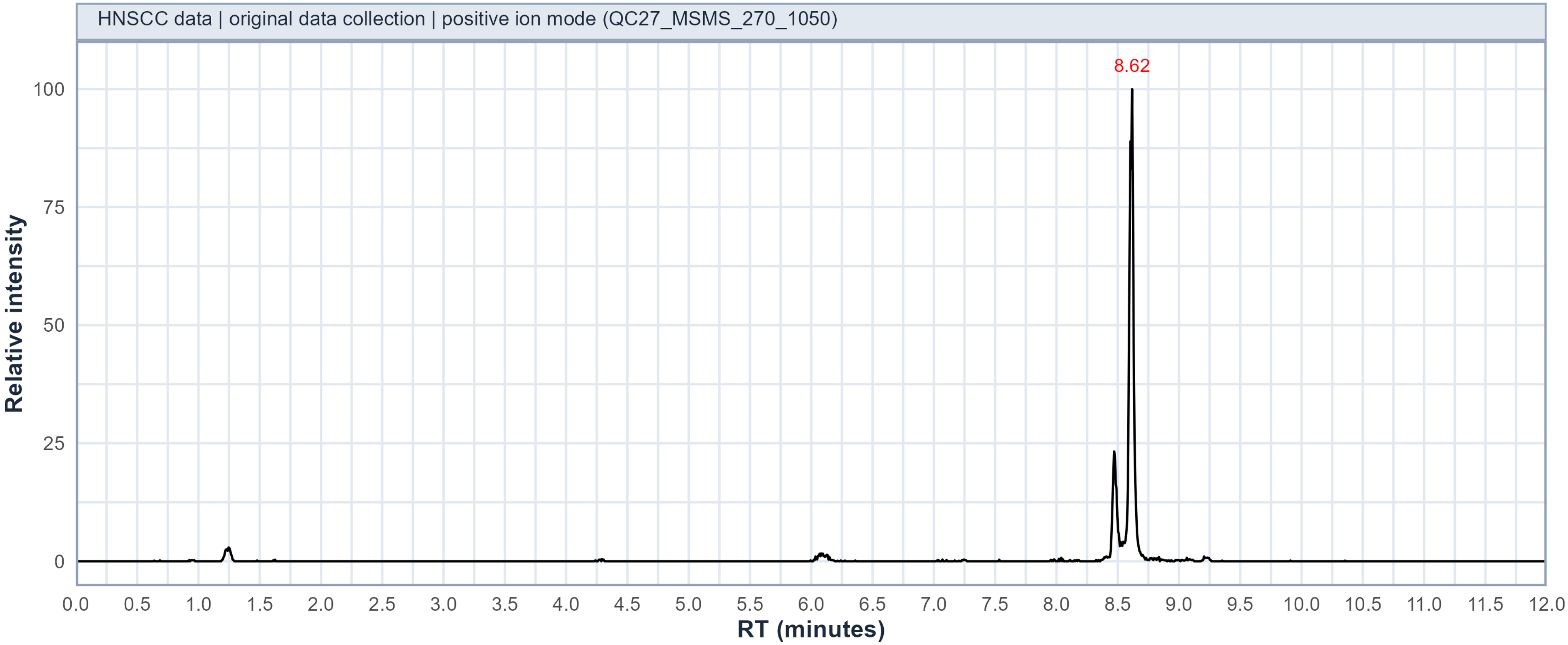


## MS/MS

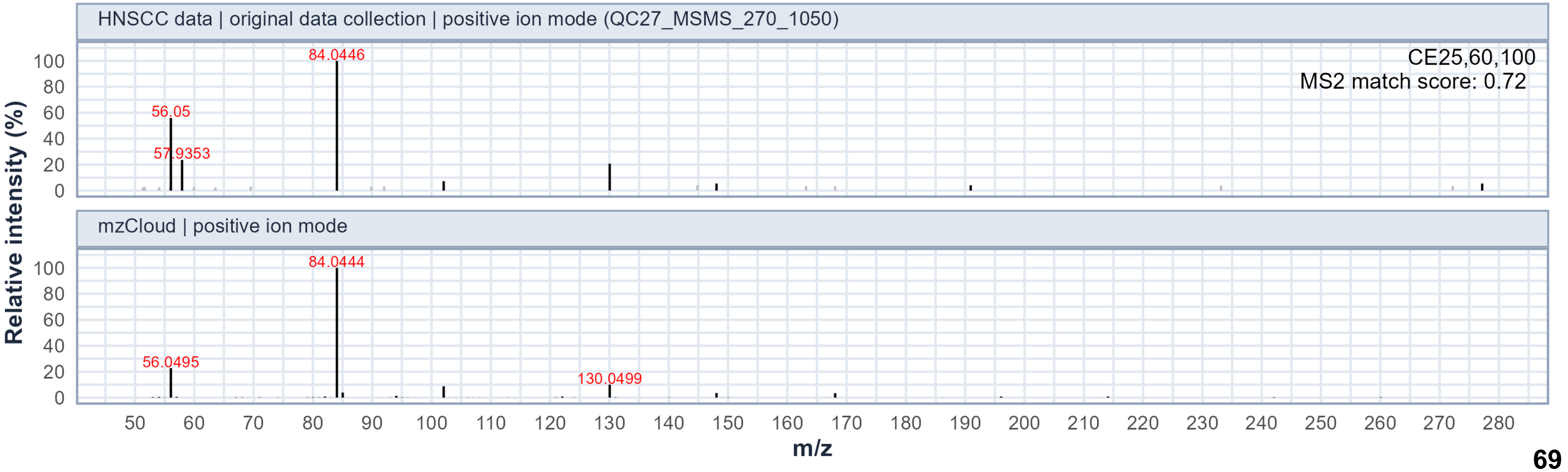

# L-Lactic acid [M-H]- | HMDB0000190

Negative ion mode: 89.0244 m/z | Instrument: Orbitrap Exploris 120

## Chromatogram

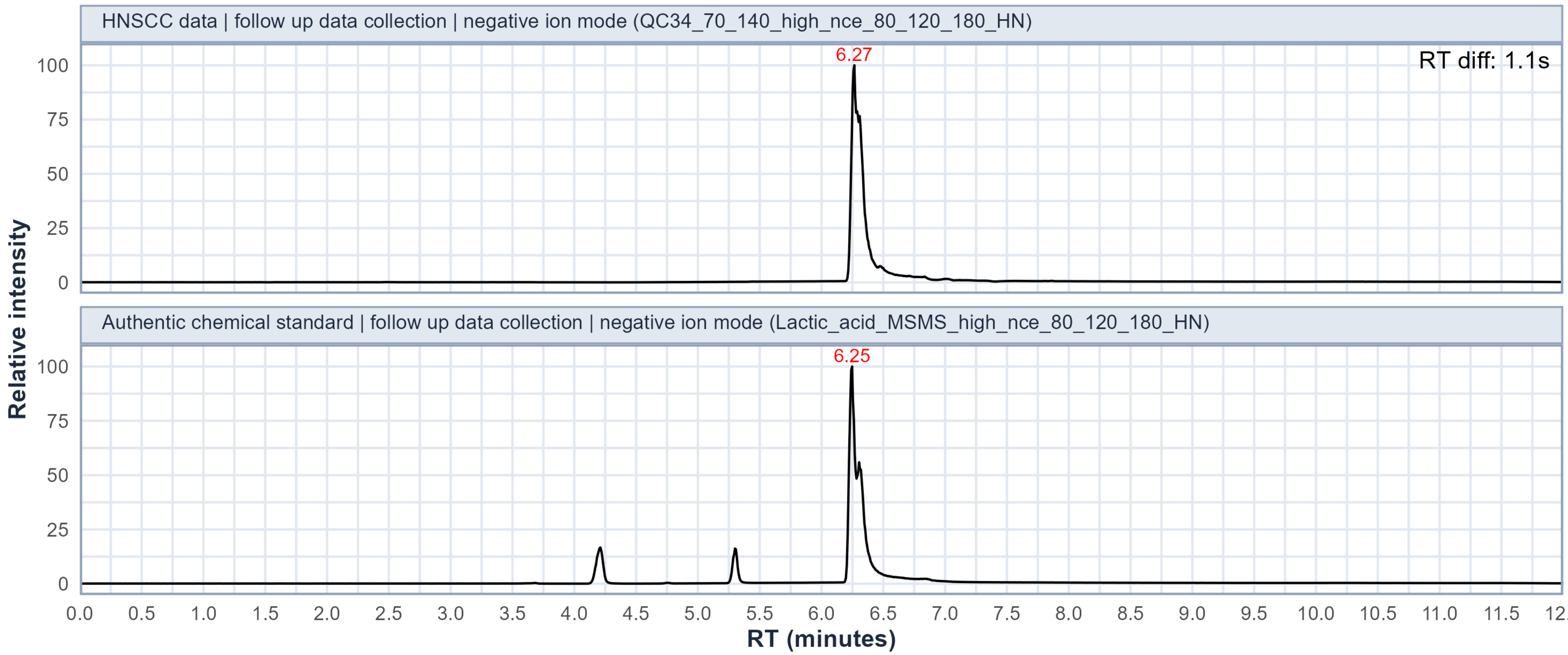


## MS/MS

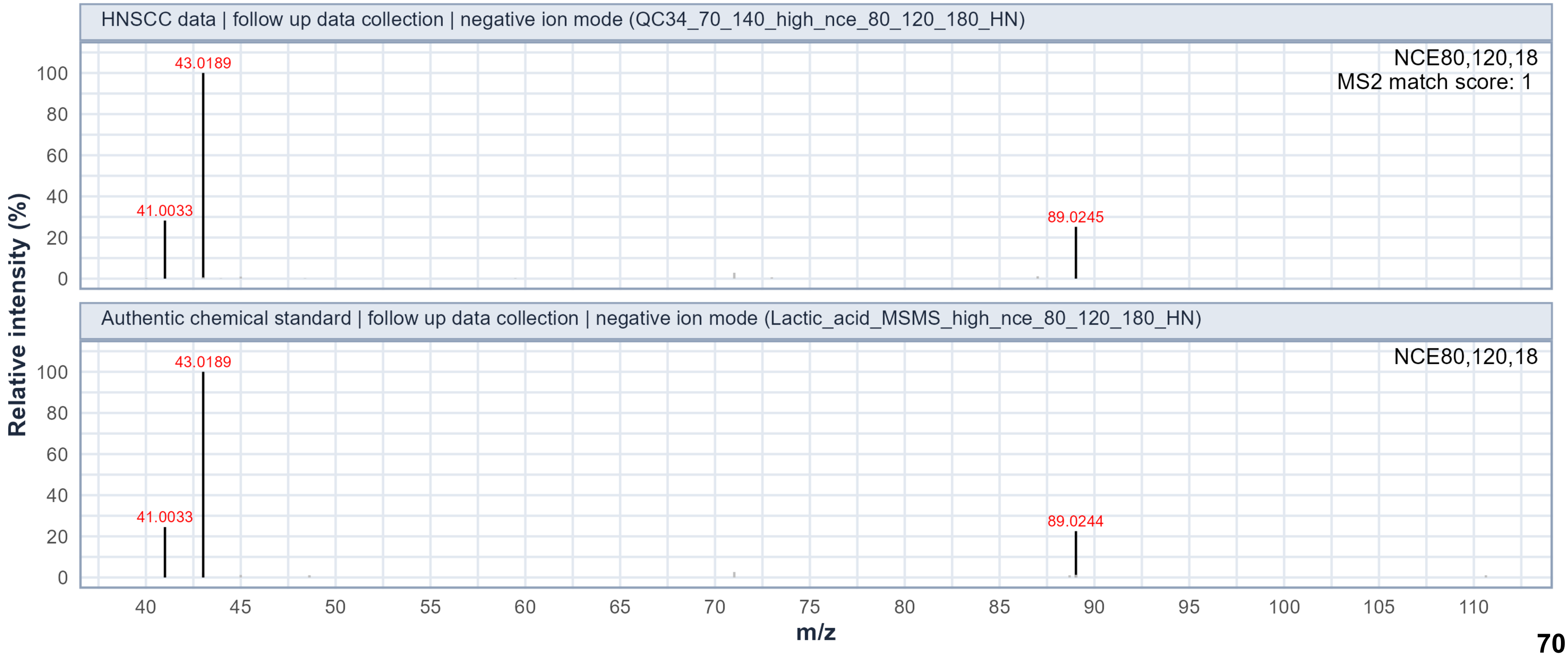

# Succinic acid [M-H]- | HMDB0000254

Negative ion mode: 117.0193 m/z | Instrument: Orbitrap Exploris 120

## Chromatogram

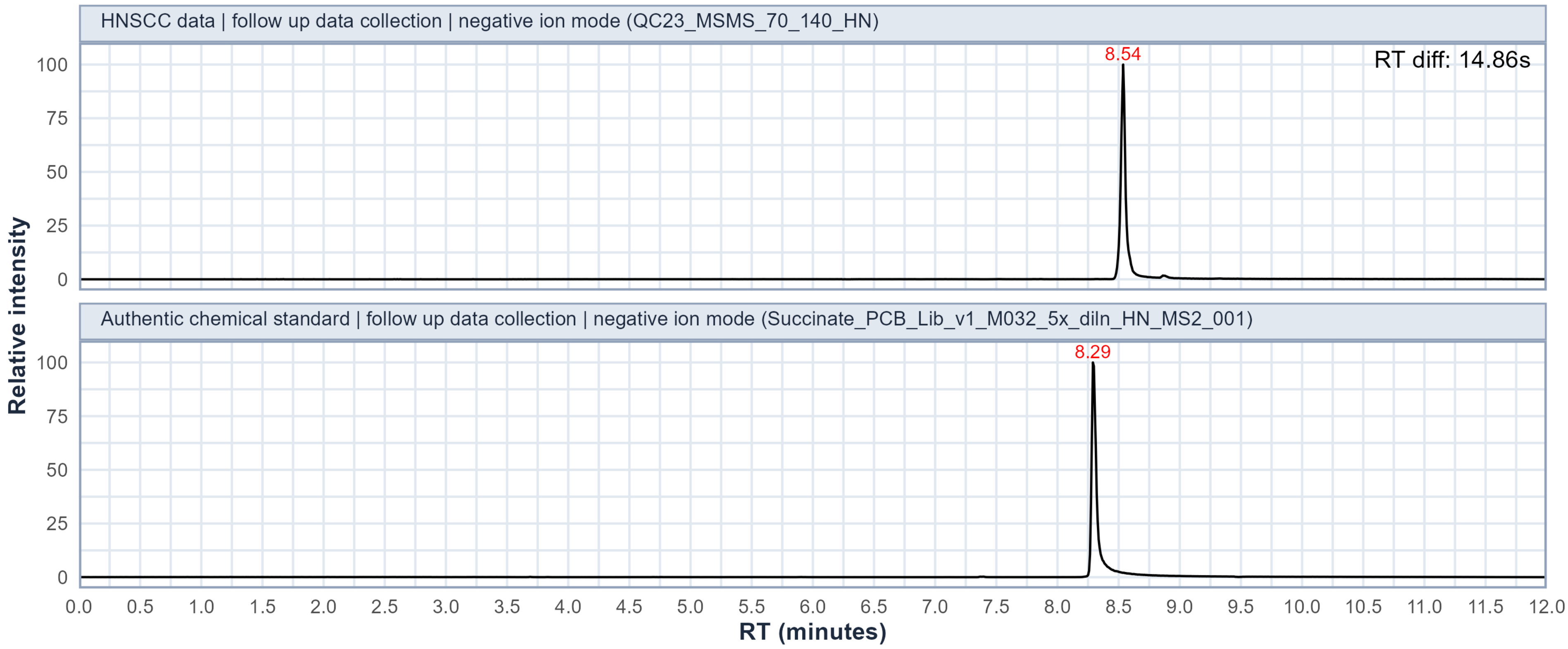


## MS/MS

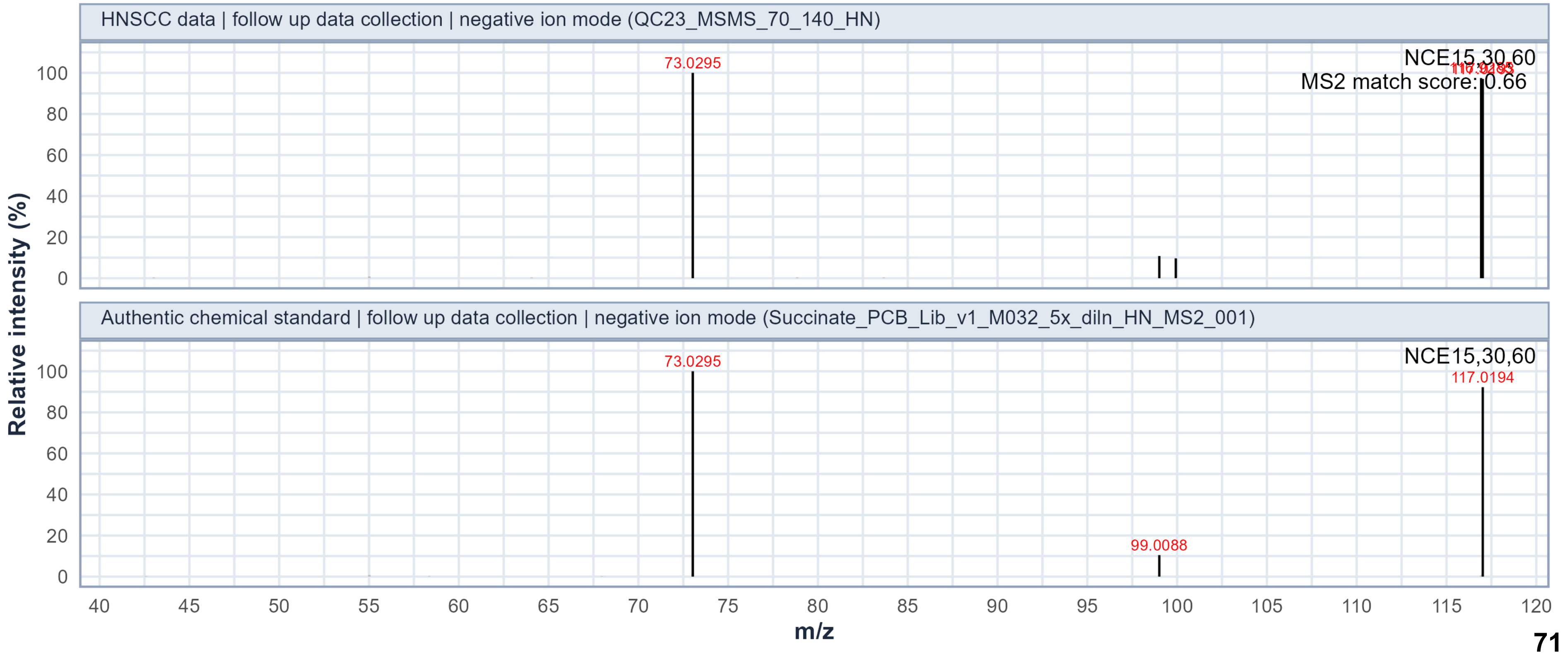

# Fumaric acid [M-H]- | HMDB0000134

Negative ion mode: 115.0037 m/z | Instrument: Orbitrap Exploris 120

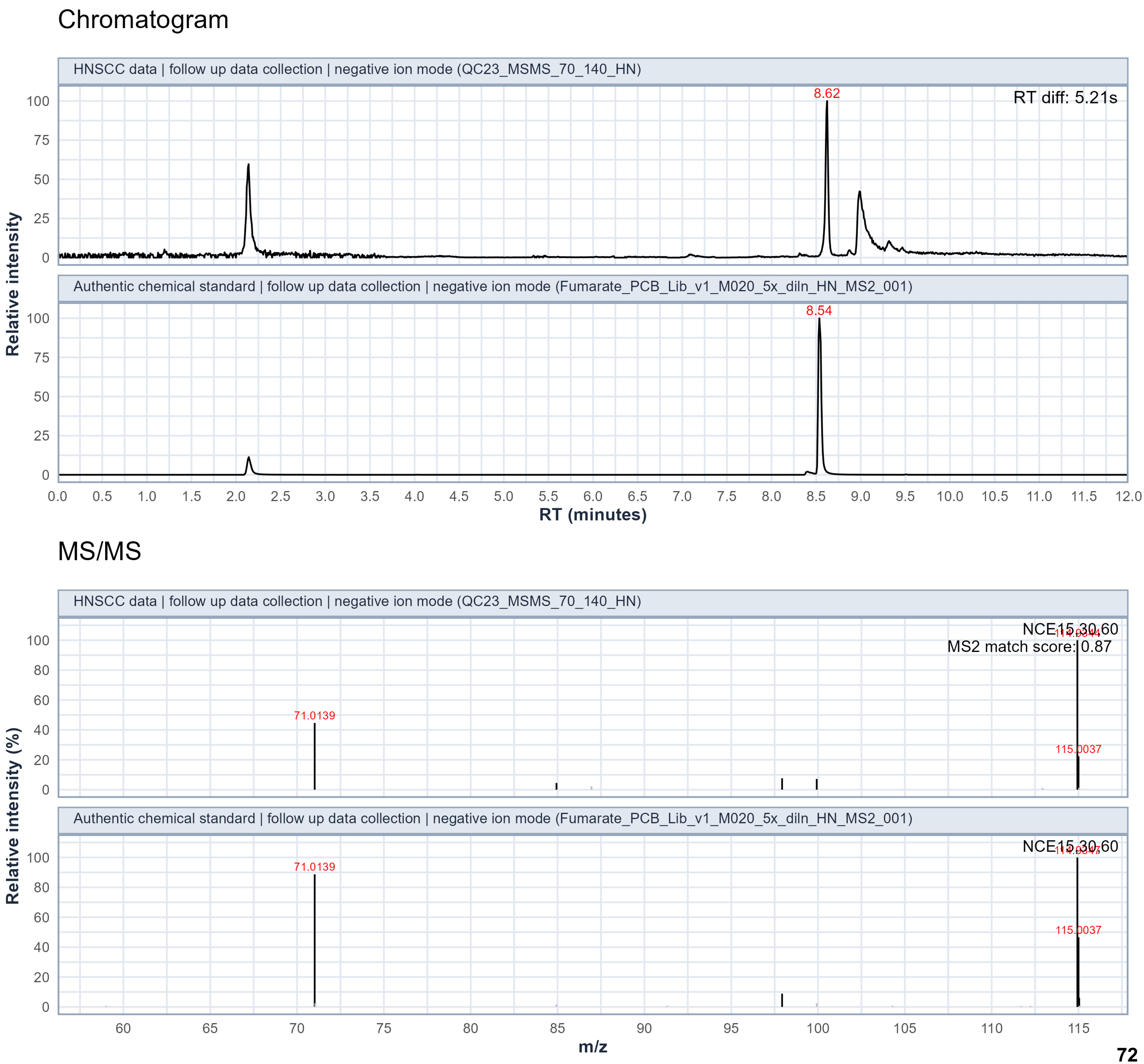

# Pyruvic acid [M-H]- | HMDB0000243

Negative ion mode: 87.0088 m/z | Instrument: Orbitrap Exploris 120

## Chromatogram

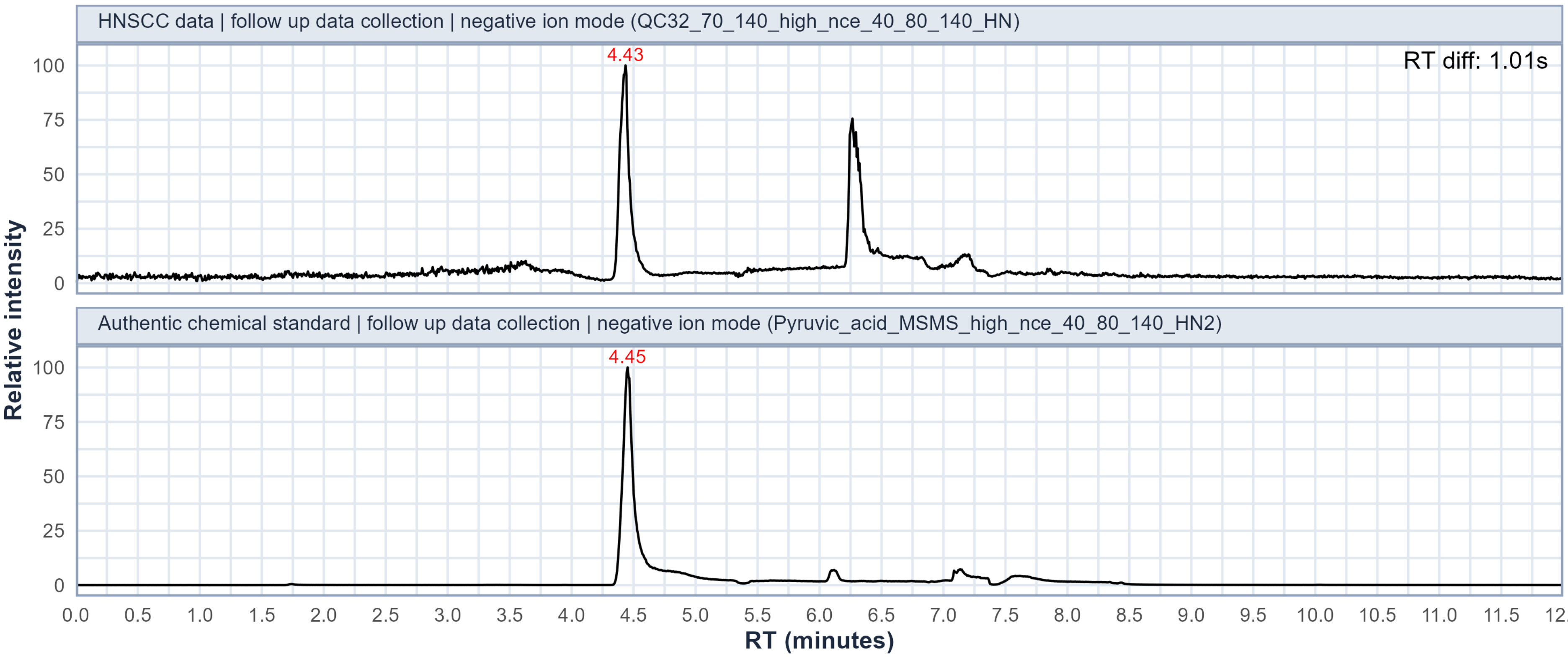


## MS/MS

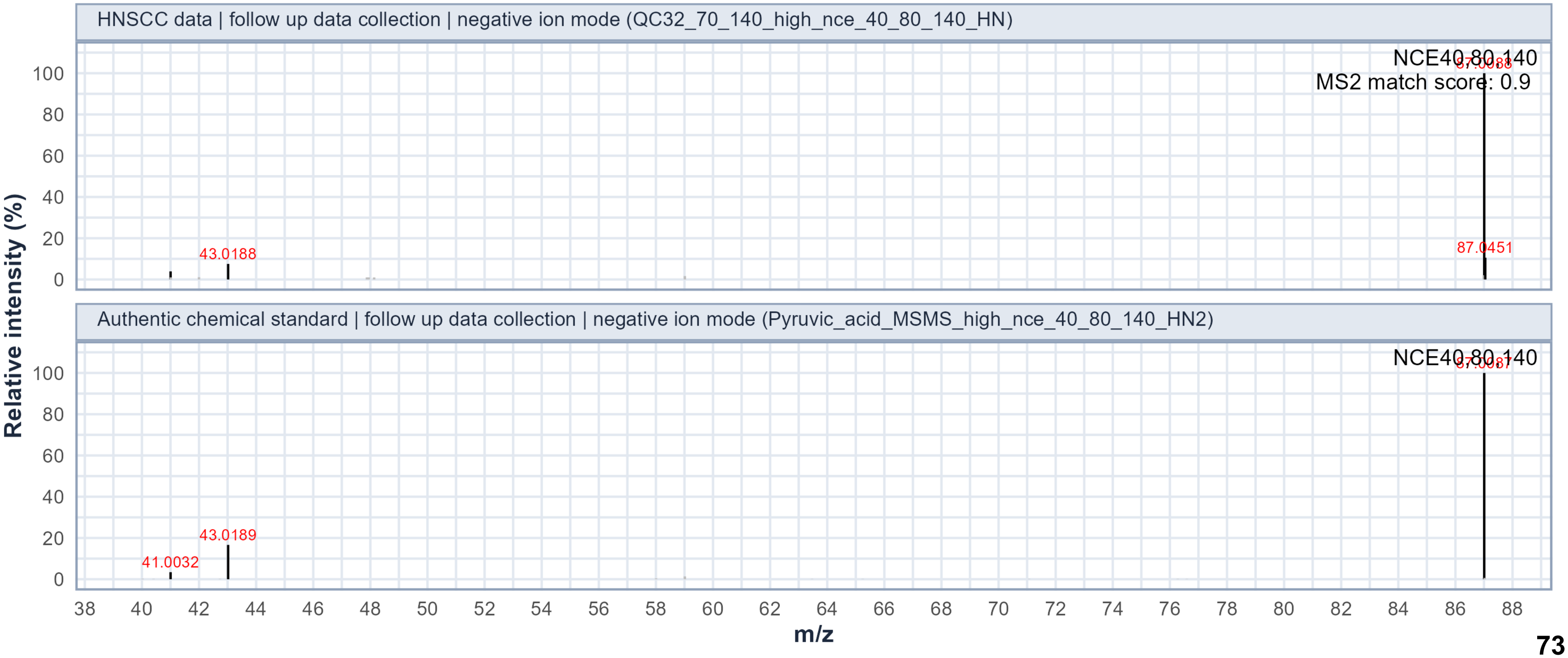

# Malic acid [M-H]- | HMDB0000744

Negative ion mode: 133.0143 m/z | Instrument: Orbitrap Exploris 120

## Chromatogram

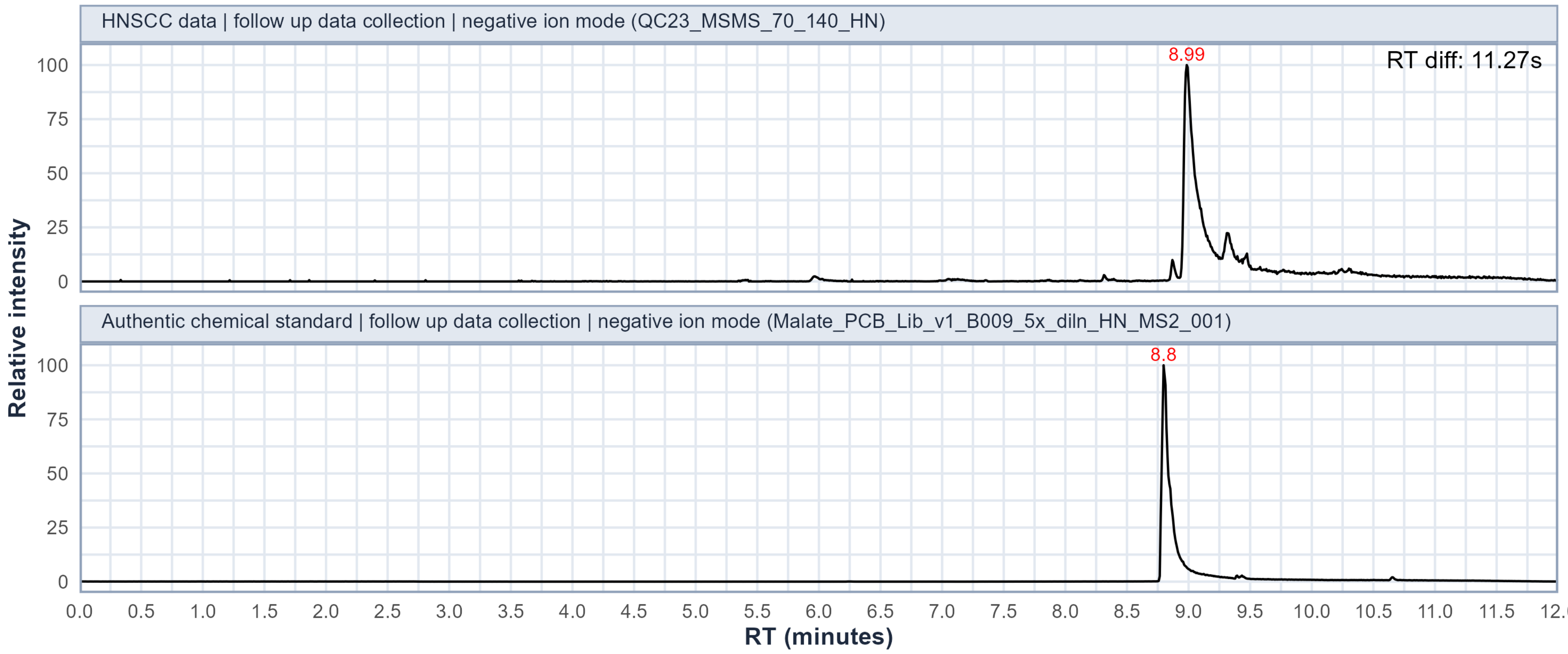


## MS/MS

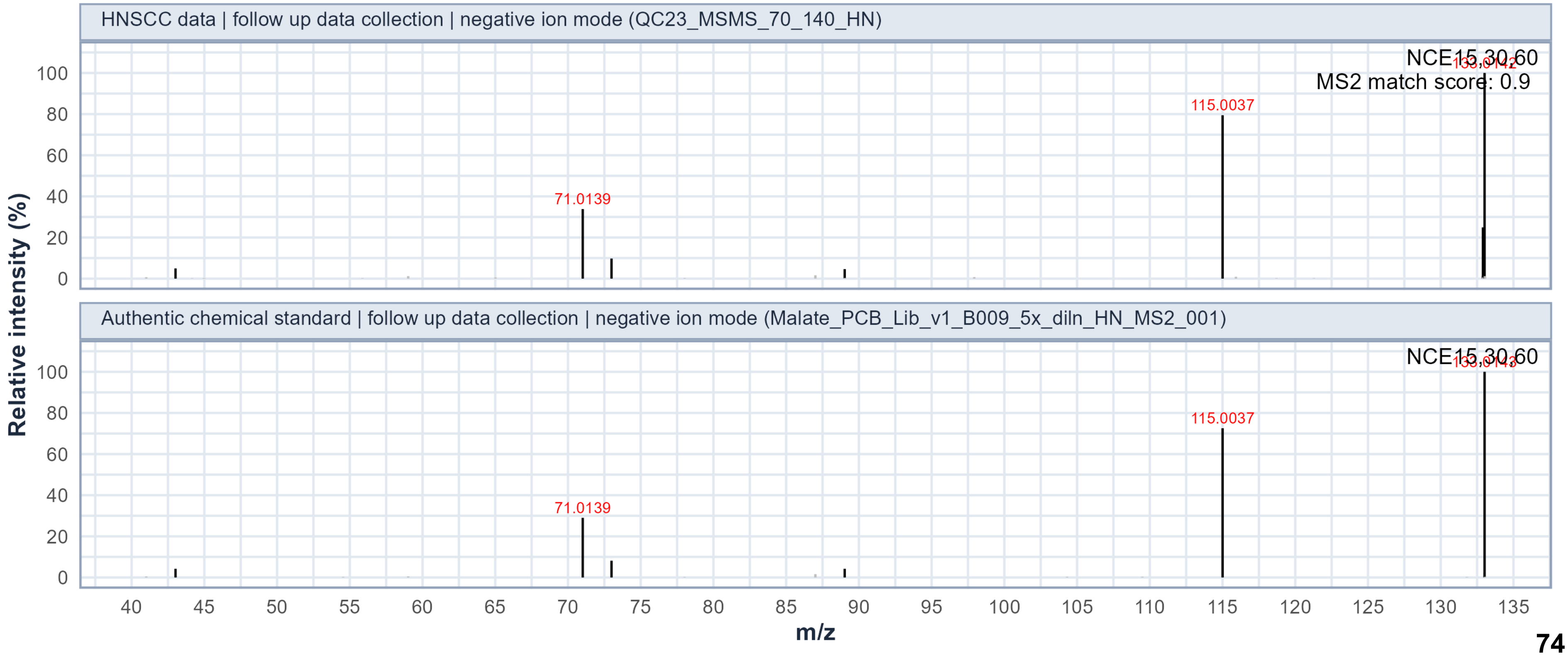

Supplement: Supplementary file 2 — Supplementary Figure S1 [file 41416_2026_3410_MOESM2_ESM.pdf]
