# Supplementary material for: Regional HNSCC metabolomics reveals widespread changes to one-carbon metabolism and S-adenosylmethionine metabolism across tumour core, tumour edge and adjacent non-tumour tissues
Source: Br J Cancer. 2026 Apr 29;135(3):372–81. doi: 10.1038/s41416-026-03410-4 (PMC13372809; doi:10.1038/s41416-026-03410-4)

Supplementary Figure S2: Spearman's rank correlation analysis of UHPLC-MS metabolite response data for metabolites related to one-carbon, polyamine and cysteine metabolism (Tables S3 and S4). Note that correlations were calculated within, not across, classes (non-tumour [N], edge tumour [E], and core tumour [C] tissues from HNSCC patients). CDP = cytidine diphosphate. *q<0.05, **q<0.01, ***q<0.001 (FDR corrected).

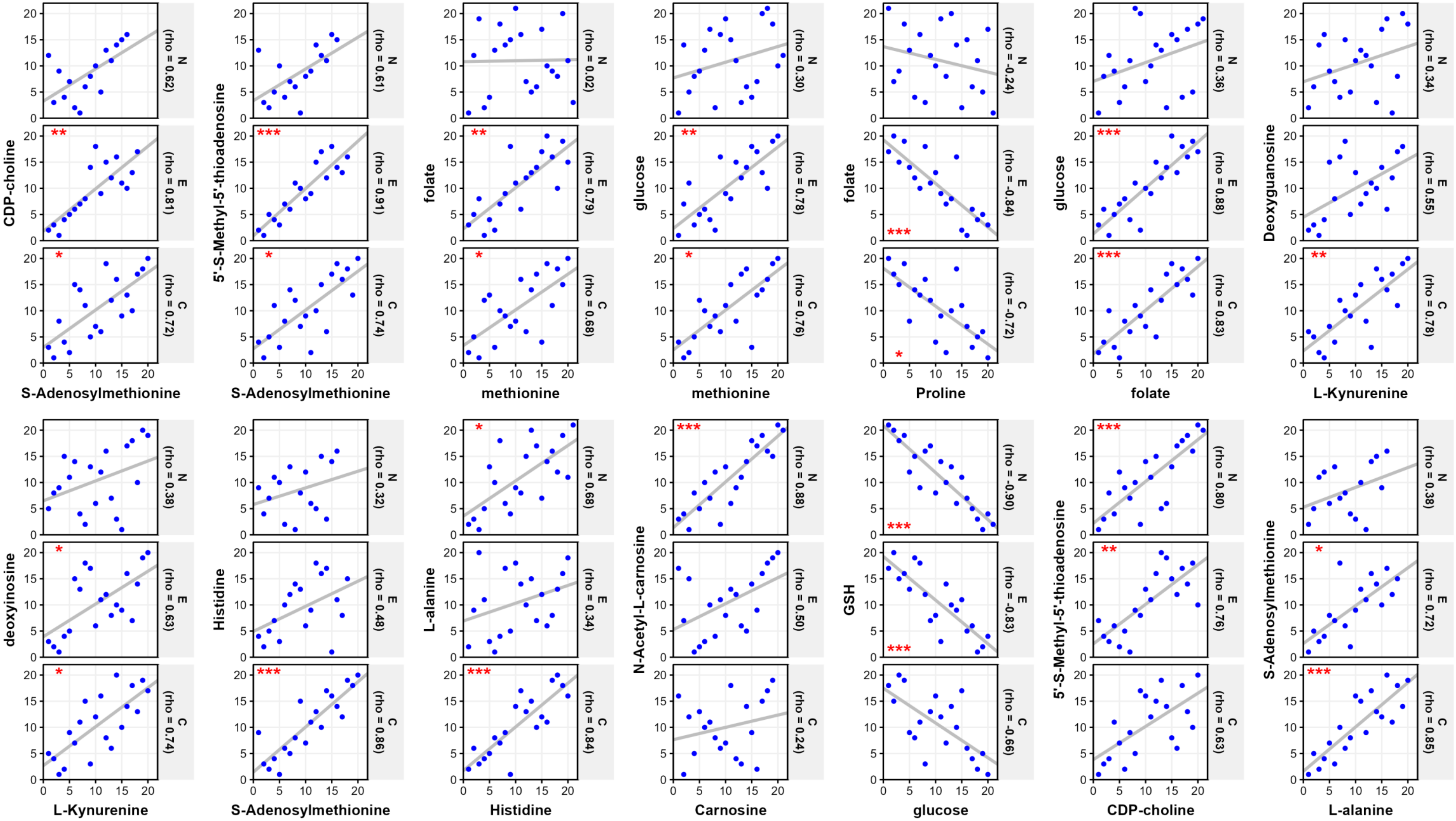

Supplement: Supplementary file 3 — Supplementary Figure S2 [file 41416_2026_3410_MOESM3_ESM.pdf]
